# Supplementary material for: Experiences of participants in a clinical trial of a novel radioactive treatment for advanced prostate cancer: A nested, qualitative longitudinal study
Source: PLoS One. 2022 Nov 9;17(11):e0276063. doi: 10.1371/journal.pone.0276063 (PMC9645653; doi:10.1371/journal.pone.0276063)
Supplement: S1 File — (DOCX) [file pone.0276063.s001.docx]

| 6/10/2022 8:14 AM | | | | | | | | | | | | | | | | | |
| --- | --- | --- | --- | --- | --- | --- | --- | --- | --- | --- | --- | --- | --- | --- | --- | --- | --- |
| Coding Summary by File | | | | | | | | | | | | | | | | | |
| Coding Interview 2 | | | | | | | | | | | | | | | | | |
| 6/10/2022 8:14 AM | | | | | | | | | | | | | | | | | |
|  | | | **Classification** |  | **Aggregate** |  | **Coverage** |  | **Number Of Coding References** |  | | **Reference Number** |  | **Coded By Initials** |  | **Modified On** |  |
| **Document** | | | | | | | | | | | | | | | | |  |
|  | **Files\\ID001.2 Removed from Trial** | | | | | | | | | | | | | | | |  |
|  | | **Code** | | | | | | | | | | | | | | |  |
|  | | | **Codes\\x - removed from trial\additional support** | | | | | | | | | | | | | |  |
|  |  |  |  |  | No |  | 0.0871 |  | 4 |  | | | | | | |  |
|  | | |  |  |  |  |  |  |  |  | | | | | | | |
|  | | | | | | | | | | | | 1 |  | BV |  | 25/03/2020 9:04 AM |  |
|  | | | I don’t know who to turn to. | | | | | | | | | | | | | |  |
|  | | |  | | | | | | | | | | | | | |  |
|  | | | | | | | | | | | | 2 |  | BV |  | 25/03/2020 9:04 AM |  |
|  | | | Don’t know where I'm going to get help from, I mean I was sent to North Lakes originally from The Prince Charles when I saw them a few years ago, they sent me over there and it’s convenient to go there, but nothing’s gone right you know. | | | | | | | | | | | | | |  |
|  | | |  | | | | | | | | | | | | | |  |
|  | | | | | | | | | | | | 3 |  | BV |  | 25/03/2020 9:05 AM |  |
|  | | | I don’t know what other trials there is. As I say, I'm in the dark, I know nothing about anything, I don’t get told anything and I don’t know who to see – I mean I can't just go to GP and ask him, he wouldn’t have a clue. | | | | | | | | | | | | | |  |
|  | | |  | | | | | | | | | | | | | |  |
|  | | | | | | | | | | | | 4 |  | BV |  | 25/03/2020 9:05 AM |  |
|  | | | I mean this guy’s a specialist, he’s the one that’s supposed to be telling me, he’s the oncologist | | | | | | | | | | | | | |  |
|  | | |  | | | | | | | | | | | | | |  |
|  | | | **Codes\\x - removed from trial\additional support\support from doctors required** | | | | | | | | | | | | | |  |
|  |  |  |  |  | No |  | 0.1767 |  | 6 |  | | | | | | |  |
|  | | |  |  |  |  |  |  |  |  | | | | | | | |
|  | | | | | | | | | | | | 1 |  | BV |  | 25/03/2020 9:06 AM |  |
|  | | | I don’t know who to turn to. | | | | | | | | | | | | | |  |
|  | | |  | | | | | | | | | | | | | |  |
|  | | | | | | | | | | | | 2 |  | BV |  | 25/03/2020 9:06 AM |  |
|  | | | Don’t know where I'm going to get help from, I mean I was sent to North Lakes originally from The Prince Charles when I saw them a few years ago, they sent me over there and it’s convenient to go there, but nothing’s gone right you know. | | | | | | | | | | | | | |  |
|  | | |  | | | | | | | | | | | | | |  |
|  | | | | | | | | | | | | 3 |  | BV |  | 25/03/2020 9:06 AM |  |
|  | | | I don’t know what other trials there is. As I say, I'm in the dark, I know nothing about anything, I don’t get told anything and I don’t know who to see – I mean I can't just go to GP and ask him, he wouldn’t have a clue. | | | | | | | | | | | | | |  |
|  | | |  | | | | | | | | | | | | | |  |
| Formatted Reports\\Coding Summary by File Formatted Report | | | | | | | | | | | Page 1 of 143 | | | | | | |
| 6/10/2022 8:14 AM | | | | | | | | | | | | | | | | | |
|  | | | **Classification** |  | **Aggregate** |  | **Coverage** |  | **Number Of Coding References** |  | | **Reference Number** |  | **Coded By Initials** |  | **Modified On** |  |
|  | | | | | | | | | | | | | | | | | |
|  | | | | | | | | | | | | 4 |  | BV |  | 25/03/2020 9:06 AM |  |
|  | | | I mean this guy’s a specialist, he’s the one that’s supposed to be telling me, he’s the oncologist | | | | | | | | | | | | | |  |
|  | | |  | | | | | | | | | | | | | |  |
|  | | | | | | | | | | | | 5 |  | BV |  | 25/03/2020 9:05 AM |  |
|  | | | I mean no one’s – I mean he’s the oncologist, he should be telling me these things; no one’s told me anything. | | | | | | | | | | | | | |  |
|  | | |  | | | | | | | | | | | | | |  |
|  | | | | | | | | | | | | 6 |  | BV |  | 25/03/2020 9:06 AM |  |
|  | | | No. I see him before every chemo, every three weeks I see him for ten minutes or whatever it is, not even it’s probably about five minutes and then I have my treatment which I won't be having this next time, I won't be having the treatment. I also get a needle in the stomach because I've got a tumour on the spine and so I see him for about ten minutes if it’s that and then I have my treatment. But I’ll have a talk to him on the 5th and see what options there is, that’s all I can do. | | | | | | | | | | | | | |  |
|  | | |  | | | | | | | | | | | | | |  |
|  | | | **Codes\\x - removed from trial\mutual agreement doctor and patient** | | | | | | | | | | | | | |  |
|  |  |  |  |  | No |  | 0.0428 |  | 2 |  | | | | | | |  |
|  | | |  |  |  |  |  |  |  |  | | | | | | | |
|  | | | | | | | | | | | | 1 |  | BV |  | 25/03/2020 9:01 AM |  |
|  | | | I don’t know what my future is now because I can't have chemo and I mean even when I had to go to hospital, I was so crook I went to hospital and they said I was so dehydrated my kidneys were about to fail. | | | | | | | | | | | | | |  |
|  | | |  | | | | | | | | | | | | | |  |
|  | | | | | | | | | | | | 2 |  | BV |  | 25/03/2020 9:01 AM |  |
|  | | | Well he just said I was not eligible for the trial now, I had to go back on chemo. | | | | | | | | | | | | | |  |
|  | | |  | | | | | | | | | | | | | |  |
|  | | | **Codes\\x - removed from trial\mutual agreement doctor and patient\removed due to kidney failure** | | | | | | | | | | | | | |  |
|  |  |  |  |  | No |  | 0.0306 |  | 1 |  | | | | | | |  |
|  | | |  |  |  |  |  |  |  |  | | | | | | | |
|  | | | | | | | | | | | | 1 |  | BV |  | 25/03/2020 9:01 AM |  |
|  | | | I don’t know what my future is now because I can't have chemo and I mean even when I had to go to hospital, I was so crook I went to hospital and they said I was so dehydrated my kidneys were about to fail. | | | | | | | | | | | | | |  |
|  | | |  | | | | | | | | | | | | | |  |
|  | | | **Codes\\x - removed from trial\sceptical** | | | | | | | | | | | | | |  |
|  |  |  |  |  | No |  | 0.0466 |  | 1 |  | | | | | | |  |
|  | | |  |  |  |  |  |  |  |  | | | | | | | |
|  | | | | | | | | | | | | 1 |  | BV |  | 25/03/2020 9:05 AM |  |
|  | | | No, he said to me it’s Russian roulette, when you go in there you don’t know what you’re going to have. They pick certain people and they’ll say you’re having chemo; you’re having this injection and you don’t know until you get what you’re going to get; I got nothing, I was just told I was no longer eligible. | | | | | | | | | | | | | |  |
|  | | |  | | | | | | | | | | | | | |  |
|  | | | | | | | | | | | | | | | | | |
| Formatted Reports\\Coding Summary by File Formatted Report | | | | | | | | | | | Page 2 of 143 | | | | | | |
| 6/10/2022 8:14 AM | | | | | | | | | | | | | | | | | |
|  | | | **Classification** |  | **Aggregate** |  | **Coverage** |  | **Number Of Coding References** |  | | **Reference Number** |  | **Coded By Initials** |  | **Modified On** |  |
|  | | | **Codes\\x - removed from trial\sceptical\randomisation** | | | | | | | | | | | | | |  |
|  |  |  |  |  | No |  | 0.0466 |  | 1 |  | | | | | | |  |
|  | | |  |  |  |  |  |  |  |  | | | | | | | |
|  | | | | | | | | | | | | 1 |  | BV |  | 25/03/2020 9:04 AM |  |
|  | | | No, he said to me it’s Russian roulette, when you go in there you don’t know what you’re going to have. They pick certain people and they’ll say you’re having chemo; you’re having this injection and you don’t know until you get what you’re going to get; I got nothing, I was just told I was no longer eligible. | | | | | | | | | | | | | |  |
|  | | |  | | | | | | | | | | | | | |  |
|  | | | **Codes\\x - removed from trial\side effects** | | | | | | | | | | | | | |  |
|  |  |  |  |  | No |  | 0.0302 |  | 2 |  | | | | | | |  |
|  | | |  |  |  |  |  |  |  |  | | | | | | | |
|  | | | | | | | | | | | | 1 |  | BV |  | 25/03/2020 9:04 AM |  |
|  | | | It’s marvellous, I'm fit and healthy all the time until I have treatment and then I'm sick. | | | | | | | | | | | | | |  |
|  | | |  | | | | | | | | | | | | | |  |
|  | | | | | | | | | | | | 2 |  | BV |  | 25/03/2020 9:05 AM |  |
|  | | | So the other one was this needle, I don’t know anything about it because I didn’t get it, I didn’t get anything. | | | | | | | | | | | | | |  |
|  | | |  | | | | | | | | | | | | | |  |
|  | | | **Codes\\x - removed from trial\uncertain about the future** | | | | | | | | | | | | | |  |
|  |  |  |  |  | No |  | 0.1553 |  | 6 |  | | | | | | |  |
|  | | |  |  |  |  |  |  |  |  | | | | | | | |
|  | | | | | | | | | | | | 1 |  | BV |  | 25/03/2020 9:01 AM |  |
|  | | | So I honestly can't tell you anything, I don’t know what the future is. He’d say to me if you can't have chemo we’ve got other options, but I’ve never been told any, so I honestly don’t know what’s happening. | | | | | | | | | | | | | |  |
|  | | |  | | | | | | | | | | | | | |  |
|  | | | | | | | | | | | | 2 |  | BV |  | 25/03/2020 9:01 AM |  |
|  | | | Well he just said I was not eligible for the trial now, I had to go back on chemo. | | | | | | | | | | | | | |  |
|  | | |  | | | | | | | | | | | | | |  |
|  | | | | | | | | | | | | 3 |  | BV |  | 25/03/2020 9:01 AM |  |
|  | | | Well I’d like to know what options I’ve got and well I’d like something to happen, as I say I don’t know what my future is now. | | | | | | | | | | | | | |  |
|  | | |  | | | | | | | | | | | | | |  |
|  | | | | | | | | | | | | 4 |  | BV |  | 25/03/2020 9:04 AM |  |
|  | | | Don’t know where I'm going to get help from, I mean I was sent to North Lakes originally from The Prince Charles when I saw them a few years ago, they sent me over there and it’s convenient to go there, but nothing’s gone right you know. | | | | | | | | | | | | | |  |
|  | | |  | | | | | | | | | | | | | |  |
|  | | | | | | | | | | | | 5 |  | BV |  | 25/03/2020 9:04 AM |  |
|  | | | Obviously I can't have chemo, but what are the other options? He’ll say there’s other options then I won't see him for months and then he’ll say back on the chemo. | | | | | | | | | | | | | |  |
|  | | |  | | | | | | | | | | | | | |  |
|  | | | | | | | | | | | | 6 |  | BV |  | 25/03/2020 9:05 AM |  |
|  | | | I don’t know what other trials there is. As I say, I'm in the dark, I know nothing about anything, I don’t get told anything and I don’t know who to see – I mean I can't just go to GP and ask him, he wouldn’t have a clue. | | | | | | | | | | | | | |  |
|  | | |  | | | | | | | | | | | | | |  |
| Formatted Reports\\Coding Summary by File Formatted Report | | | | | | | | | | | Page 3 of 143 | | | | | | |
| 6/10/2022 8:14 AM | | | | | | | | | | | | | | | | | |
|  | | | **Classification** |  | **Aggregate** |  | **Coverage** |  | **Number Of Coding References** |  | | **Reference Number** |  | **Coded By Initials** |  | **Modified On** |  |
|  | | | **Codes\\x - removed from trial\uncertain about the future\no plan b** | | | | | | | | | | | | | |  |
|  |  |  |  |  | No |  | 0.0244 |  | 1 |  | | | | | | |  |
|  | | |  |  |  |  |  |  |  |  | | | | | | | |
|  | | | | | | | | | | | | 1 |  | BV |  | 25/03/2020 9:04 AM |  |
|  | | | Obviously I can't have chemo, but what are the other options? He’ll say there’s other options then I won't see him for months and then he’ll say back on the chemo. | | | | | | | | | | | | | |  |
|  | | |  | | | | | | | | | | | | | |  |
|  | **Files\\ID004.2 Removed from Trial** | | | | | | | | | | | | | | | |  |
|  | | **Code** | | | | | | | | | | | | | | |  |
|  | | | **Codes\\x - removed from trial\choosing alternative treatment rather than TheraP** | | | | | | | | | | | | | |  |
|  |  |  |  |  | No |  | 0.0831 |  | 2 |  | | | | | | |  |
|  | | |  |  |  |  |  |  |  |  | | | | | | | |
|  | | | | | | | | | | | | 1 |  | BV |  | 25/03/2020 8:49 AM |  |
|  | | | So I didn’t really have much option and you know talked about the Lupin and they were going to be recruiting new participants, but at that stage it wasn’t functioning. So anyway fortunately Shahine Sander who was the oncologist Lupin and they sort of made the process fairly quickly. So in fact I was approved before they received results of final PEP scans. So went up there and signed on and then was treated the same day. | | | | | | | | | | | | | |  |
|  | | |  | | | | | | | | | | | | | |  |
|  | | | | | | | | | | | | 2 |  | BV |  | 25/03/2020 8:50 AM |  |
|  | | | Really as far as I could see there was really no alternative but to persist with it until I could get the Lupin in place. | | | | | | | | | | | | | |  |
|  | | |  | | | | | | | | | | | | | |  |
|  | | | **Codes\\x - removed from trial\initial motivation for enrolment** | | | | | | | | | | | | | |  |
|  |  |  |  |  | No |  | 0.1934 |  | 1 |  | | | | | | |  |
|  | | |  |  |  |  |  |  |  |  | | | | | | | |
|  | | | | | | | | | | | | 1 |  | BV |  | 25/03/2020 8:51 AM |  |
|  | | | Oh yes well I mean my experience throughout the whole story of my disease has been that I have to be vigilant informed. I make my own analyses of test results. They have led to me drawing attention to things which have not been mentioned by radiologists for example and led to further investigations or treatment. I mean getting onto TheraP was really my initiative because my regular oncologist that okay the next stage is Cabazitaxel and I said oh well TheraP as Cabazitaxel or what lots of people think I would be a good candidate for Lutetium, so he referred me to Monash for some reason because I am a 20 minute walk from Peter Mac. So essentially I contacted Peter Mac and they were able to let me do TheraP even more quickly than Monash could have. So I would have to say the whole I would have to say the whole history has required me to be fairly active and you know challenging but once into TheraP there well the process all went smoothly and it really just was that issue of me feeling like it was unlikely it was doing any good. Shahine insisting that there was a chance of it turning around before the final one I was a 20% chance. Well she knows more about it than I do but I was sceptical about that, but then she certainly helped me to get into Lupin. | | | | | | | | | | | | | |  |
|  |  |  |  |  |  |  |  |  |  |  |  |  |  |  |  |  |  |
|  | | |  | | | | | | | | | | | | | |  |
|  | | | **Codes\\x - removed from trial\mutual agreement doctor and patient** | | | | | | | | | | | | | |  |
|  |  |  |  |  | No |  | 0.2035 |  | 2 |  | | | | | | |  |
|  | | |  |  |  |  |  |  |  |  | | | | | | | |
|  | | | | | | | | | | | | 1 |  | BV |  | 25/03/2020 8:50 AM |  |
|  | | | Well that was mutually agreed that TheraP wasn’t doing it for me. | | | | | | | | | | | | | |  |
|  | | |  | | | | | | | | | | | | | |  |
| Formatted Reports\\Coding Summary by File Formatted Report | | | | | | | | | | | Page 4 of 143 | | | | | | |
| 6/10/2022 8:14 AM | | | | | | | | | | | | | | | | | |
|  | | | **Classification** |  | **Aggregate** |  | **Coverage** |  | **Number Of Coding References** |  | | **Reference Number** |  | **Coded By Initials** |  | **Modified On** |  |
|  | | | | | | | | | | | | | | | | | |
|  | | | | | | | | | | | | 2 |  | BV |  | 25/03/2020 8:51 AM |  |
|  | | | Oh yes well I mean my experience throughout the whole story of my disease has been that I have to be vigilant informed. I make my own analyses of test results. They have led to me drawing attention to things which have not been mentioned by radiologists for example and led to further investigations or treatment. I mean getting onto TheraP was really my initiative because my regular oncologist that okay the next stage is Cabazitaxel and I said oh well TheraP as Cabazitaxel or what lots of people think I would be a good candidate for Lutetium, so he referred me to Monash for some reason because I am a 20 minute walk from Peter Mac. So essentially I contacted Peter Mac and they were able to let me do TheraP even more quickly than Monash could have. So I would have to say the whole I would have to say the whole history has required me to be fairly active and you know challenging but once into TheraP there well the process all went smoothly and it really just was that issue of me feeling like it was unlikely it was doing any good. Shahine insisting that there was a chance of it turning around before the final one I was a 20% chance. Well she knows more about it than I do but I was sceptical about that, but then she certainly helped me to get into Lupin. | | | | | | | | | | | | | |  |
|  |  |  |  |  |  |  |  |  |  |  |  |  |  |  |  |  |  |
|  | | |  | | | | | | | | | | | | | |  |
|  | | | **Codes\\x - removed from trial\partner** | | | | | | | | | | | | | |  |
|  |  |  |  |  | No |  | 0.0521 |  | 1 |  | | | | | | |  |
|  | | |  |  |  |  |  |  |  |  | | | | | | | |
|  | | | | | | | | | | | | 1 |  | BV |  | 25/03/2020 8:52 AM |  |
|  | | | Well she finds it – in fact she has just written a little letter to one of her friends sort of describing the experience and how kind of difficult not being able to make plans and being rather short term, and so I think she’s finding it quite difficult, but she is a great support to me and she’s terrific but publicly it is very hard for her. | | | | | | | | | | | | | |  |
|  | | |  | | | | | | | | | | | | | |  |
|  | | | **Codes\\x - removed from trial\quality of trialists** | | | | | | | | | | | | | |  |
|  |  |  |  |  | No |  | 0.0556 |  | 1 |  | | | | | | |  |
|  | | |  |  |  |  |  |  |  |  | | | | | | | |
|  | | | | | | | | | | | | 1 |  | BV |  | 25/03/2020 8:51 AM |  |
|  | | | Oh no, I mean I was glad to be able to get into it. Peter Mac was an easier process to get in, much quicker than I had expected and the whole system seemed to work. You know it’s a very busy public hospital so I often had to wait and so on but that’s to be expected, but no I think it all worked as it was supposed to and it was unfortunate it was not effective. | | | | | | | | | | | | | |  |
|  | | |  | | | | | | | | | | | | | |  |
|  | | | **Codes\\x - removed from trial\sceptical** | | | | | | | | | | | | | |  |
|  |  |  |  |  | No |  | 0.1468 |  | 4 |  | | | | | | |  |
|  | | |  |  |  |  |  |  |  |  | | | | | | | |
|  | | | | | | | | | | | | 1 |  | BV |  | 25/03/2020 8:48 AM |  |
|  | | | Well I guess my concern was that immediate, like that PSA test a couple of weeks after initial treatment showed a great increase and the trial organiser was pragmatic about that saying oh you know it can well increase but eventually come down, but seeing that it increased about – I can’t remember the exact figures now but it’s something like that maybe 30%-50% from the baseline, and I would say if it’s going to come down below 50% of baseline it’s got a long way to come down. | | | | | | | | | | | | | |  |
|  | | |  | | | | | | | | | | | | | |  |
|  | | | | | | | | | | | | 2 |  | BV |  | 25/03/2020 8:48 AM |  |
|  | | | Anyway she still felt that it was important to continue, and really there was no Plan B. | | | | | | | | | | | | | |  |
|  | | |  | | | | | | | | | | | | | |  |
|  | | | | | | | | | | | | 3 |  | BV |  | 25/03/2020 8:49 AM |  |
|  | | | in terms of TheraP yeah I mean I was highly sceptical about the turnaround because the PSA just kept going on and on, and then when I did have the final PEP scans there was a fairly massive increase in what was already a very high disease burden, but that’s the breaks I guess. | | | | | | | | | | | | | |  |
|  | | |  | | | | | | | | | | | | | |  |
|  | | | | | | | | | | | | 4 |  | BV |  | 25/03/2020 8:50 AM |  |
|  | | | Really as far as I could see there was really no alternative but to persist with it until I could get the Lupin in place. | | | | | | | | | | | | | |  |
|  | | |  | | | | | | | | | | | | | |  |
| Formatted Reports\\Coding Summary by File Formatted Report | | | | | | | | | | | Page 5 of 143 | | | | | | |
| 6/10/2022 8:14 AM | | | | | | | | | | | | | | | | | |
|  | | | **Classification** |  | **Aggregate** |  | **Coverage** |  | **Number Of Coding References** |  | | **Reference Number** |  | **Coded By Initials** |  | **Modified On** |  |
|  | | | **Codes\\x - removed from trial\sceptical\TheraP not effective** | | | | | | | | | | | | | |  |
|  |  |  |  |  | No |  | 0.0556 |  | 1 |  | | | | | | |  |
|  | | |  |  |  |  |  |  |  |  | | | | | | | |
|  | | | | | | | | | | | | 1 |  | BV |  | 25/03/2020 8:51 AM |  |
|  | | | Oh no, I mean I was glad to be able to get into it. Peter Mac was an easier process to get in, much quicker than I had expected and the whole system seemed to work. You know it’s a very busy public hospital so I often had to wait and so on but that’s to be expected, but no I think it all worked as it was supposed to and it was unfortunate it was not effective. | | | | | | | | | | | | | |  |
|  | | |  | | | | | | | | | | | | | |  |
|  | | | **Codes\\x - removed from trial\side effects\quality of life changes - cabazitaxel** | | | | | | | | | | | | | |  |
|  |  |  |  |  | No |  | 0.0449 |  | 1 |  | | | | | | |  |
|  | | |  |  |  |  |  |  |  |  | | | | | | | |
|  | | | | | | | | | | | | 1 |  | BV |  | 25/03/2020 8:45 AM |  |
|  | | | I certainly felt quite unwell and fatigued, and I have now finished taking the supplemental drug as I’m hoping things might improve at bit and I am certainly better than I was but I still don’t feel terribly well but I’m functional. I might have a lie down sometime during the day but maybe not. | | | | | | | | | | | | | |  |
|  | | |  | | | | | | | | | | | | | |  |
|  | | | **Codes\\x - removed from trial\side effects\side effects - cabazitaxel** | | | | | | | | | | | | | |  |
|  |  |  |  |  | No |  | 0.2034 |  | 2 |  | | | | | | |  |
|  | | |  |  |  |  |  |  |  |  | | | | | | | |
|  | | | | | | | | | | | | 1 |  | BV |  | 25/03/2020 8:44 AM |  |
|  | | | Well I’m suffering some side effects from my current treatment which was about 10-11 days ago. So there is the radio nuclear treatment plus Dox 66 is the experimental drug which is meant to enhance the effects of the radiation, and both of those have advertised side effect and fatigue. I don’t have nausea, but my sort of feeling is on that spectrum kind of uncomfortable, and the first probably three days or so after the treatment I felt okay and I was in Sydney at the time, and did quite a lot of walking and by the time I got home it started to hit me and I certainly felt quite unwell and fatigued, and I have now finished taking the supplemental drug as I’m hoping things might improve at bit and I am certainly better than I was but I still don’t feel terribly well but I’m functional. I might have a lie down sometime during the day but maybe not. | | | | | | | | | | | | | |  |
|  |  |  |  |  |  |  |  |  |  |  |  |  |  |  |  |  |  |
|  | | |  | | | | | | | | | | | | | |  |
|  | | | | | | | | | | | | 2 |  | BV |  | 25/03/2020 8:48 AM |  |
|  | | | Well I guess my concern was that immediate, like that PSA test a couple of weeks after initial treatment showed a great increase and the trial organiser was pragmatic about that saying oh you know it can well increase but eventually come down, but seeing that it increased about – I can’t remember the exact figures now but it’s something like that maybe 30%-50% from the baseline, and I would say if it’s going to come down below 50% of baseline it’s got a long way to come down. | | | | | | | | | | | | | |  |
|  | | |  | | | | | | | | | | | | | |  |
|  | | | **Codes\\x - removed from trial\uncertain about the future\no plan b** | | | | | | | | | | | | | |  |
|  |  |  |  |  | No |  | 0.0319 |  | 2 |  | | | | | | |  |
|  | | |  |  |  |  |  |  |  |  | | | | | | | |
|  | | | | | | | | | | | | 1 |  | BV |  | 25/03/2020 8:48 AM |  |
|  | | | Anyway she still felt that it was important to continue, and really there was no Plan B. | | | | | | | | | | | | | |  |
|  | | |  | | | | | | | | | | | | | |  |
|  | | | | | | | | | | | | 2 |  | BV |  | 25/03/2020 8:50 AM |  |
|  | | | Really as far as I could see there was really no alternative but to persist with it until I could get the Lupin in place. | | | | | | | | | | | | | |  |
|  | | |  | | | | | | | | | | | | | |  |
|  | | | | | | | | | | | | | | | | | |
| Formatted Reports\\Coding Summary by File Formatted Report | | | | | | | | | | | Page 6 of 143 | | | | | | |
| 6/10/2022 8:14 AM | | | | | | | | | | | | | | | | | |
|  | | | **Classification** |  | **Aggregate** |  | **Coverage** |  | **Number Of Coding References** |  | | **Reference Number** |  | **Coded By Initials** |  | **Modified On** |  |
|  | **Files\\ID005.2 Removed from Trial** | | | | | | | | | | | | | | | |  |
|  | | **Code** | | | | | | | | | | | | | | |  |
|  | | | **Codes\\x - removed from trial\additional support** | | | | | | | | | | | | | |  |
|  |  |  |  |  | No |  | 0.1076 |  | 1 |  | | | | | | |  |
|  | | |  |  |  |  |  |  |  |  | | | | | | | |
|  | | | | | | | | | | | | 1 |  | BV |  | 25/03/2020 8:56 AM |  |
|  | | | Well no I did – I did get some care in. I got – oh gosh, gosh Auscare to come in and to clean my house once every 2 weeks but then that’s no great drama because I – I’m the only that lives in it. It’s a very clean little modern townhouse, 4 years old. So that’s all done, every 2 weeks. I have Anglicare that will take me to the doctor or the hospital or wherever I’ve got to go and that’s no problem. My biggest problem now is that I’ve come in here, is to whether I go home or not and I’d like to try to go home to see how I can cope and that’s the next big trial. If I can’t cope then if I – how do I cope if I get extra people in to come in and help me cook meals. I’ve been living on pre-cooked meals now for some 4 or 5 months and I can’t cook myself anymore. But that’s okay. I’ve been able to – but now it’s – I don’t know how I’m going to go when I go home this time and that’s why everything is very much on the table. No decisions are being made until I – I trial everything. I go home and see how I go; I’ll have to come back in here. And I must admit I’m – I’m really incredibly pleased with the things they do here at Prince Charles. | | | | | | | | | | | | | |  |
|  |  |  |  |  |  |  |  |  |  |  |  |  |  |  |  |  |  |
|  | | |  | | | | | | | | | | | | | |  |
|  | | | **Codes\\x - removed from trial\additional support\support from doctors required** | | | | | | | | | | | | | |  |
|  |  |  |  |  | No |  | 0.1076 |  | 1 |  | | | | | | |  |
|  | | |  |  |  |  |  |  |  |  | | | | | | | |
|  | | | | | | | | | | | | 1 |  | BV |  | 25/03/2020 9:06 AM |  |
|  | | | Well no I did – I did get some care in. I got – oh gosh, gosh Auscare to come in and to clean my house once every 2 weeks but then that’s no great drama because I – I’m the only that lives in it. It’s a very clean little modern townhouse, 4 years old. So that’s all done, every 2 weeks. I have Anglicare that will take me to the doctor or the hospital or wherever I’ve got to go and that’s no problem. My biggest problem now is that I’ve come in here, is to whether I go home or not and I’d like to try to go home to see how I can cope and that’s the next big trial. If I can’t cope then if I – how do I cope if I get extra people in to come in and help me cook meals. I’ve been living on pre-cooked meals now for some 4 or 5 months and I can’t cook myself anymore. But that’s okay. I’ve been able to – but now it’s – I don’t know how I’m going to go when I go home this time and that’s why everything is very much on the table. No decisions are being made until I – I trial everything. I go home and see how I go; I’ll have to come back in here. And I must admit I’m – I’m really incredibly pleased with the things they do here at Prince Charles. | | | | | | | | | | | | | |  |
|  |  |  |  |  |  |  |  |  |  |  |  |  |  |  |  |  |  |
|  | | |  | | | | | | | | | | | | | |  |
|  | | | **Codes\\x - removed from trial\additional support\support of family** | | | | | | | | | | | | | |  |
|  |  |  |  |  | No |  | 0.0596 |  | 1 |  | | | | | | |  |
|  | | |  |  |  |  |  |  |  |  | | | | | | | |
|  | | | | | | | | | | | | 1 |  | BV |  | 25/03/2020 8:55 AM |  |
|  | | | certainly did. I make all my decisions by myself. I don’t have a partner. My – my ex-wife is still a live. She questions me a lot of course still. My kids are – you know putting me a third degree ringer and that’s okay. I’m used to being in charge. That’s the probably the best way. I’ve – I’ve always run my own companies and run my own – well I’ve got a wonderful family … strong family life and kids without any mental problems. I’ve got to the end of my life and I’ve – and we’ve all thought – we’ve had long discussions about this. Got to the end of my life saying well it’s been a very interesting one and it’s time to go. | | | | | | | | | | | | | |  |
|  | | |  | | | | | | | | | | | | | |  |
|  | | | | | | | | | | | | | | | | | |
|  | | | | | | | | | | | | | | | | | |
| Formatted Reports\\Coding Summary by File Formatted Report | | | | | | | | | | | Page 7 of 143 | | | | | | |
| 6/10/2022 8:14 AM | | | | | | | | | | | | | | | | | |
|  | | | **Classification** |  | **Aggregate** |  | **Coverage** |  | **Number Of Coding References** |  | | **Reference Number** |  | **Coded By Initials** |  | **Modified On** |  |
|  | | | **Codes\\x - removed from trial\disappointed** | | | | | | | | | | | | | |  |
|  |  |  |  |  | No |  | 0.0957 |  | 1 |  | | | | | | |  |
|  | | |  |  |  |  |  |  |  |  | | | | | | | |
|  | | | | | | | | | | | | 1 |  | BV |  | 25/03/2020 8:57 AM |  |
|  | | | No, no not at all. No well I’ve been – if you can stop me from being sick yes I would have loved that but there’s no way that it could be done by pills and if you could have given me all my energy back that – I would have kept going but no nothing – there was nothing that could keep me back on the trial. I mean I must admit I was disappointed when I had to say to Doctor Goh that I’m sorry mate I can’t keep going on this, my quality of life is – is down the gurgler. And if there was – there was basically nothing – he tried a couple of things but I can’t remember what they were for pain relief and but it’s the – it’s – instead of getting out of bed at 7 o’clock I was having trouble pushing myself out of bed at 8:30 or 9 o’clock apart from someone ringing me and saying I’m coming around to see you but - or I had to give away all my – the activities that I loved, my volunteer activities purely for sheer lack of stamina and I couldn’t see – oh sorry he couldn’t find anyway where I could get all my stamina back again. | | | | | | | | | | | | | |  |
|  |  |  |  |  |  |  |  |  |  |  |  |  |  |  |  |  |  |
|  | | |  | | | | | | | | | | | | | |  |
|  | | | **Codes\\x - removed from trial\facing mortality** | | | | | | | | | | | | | |  |
|  |  |  |  |  | No |  | 0.3591 |  | 6 |  | | | | | | |  |
|  | | |  |  |  |  |  |  |  |  | | | | | | | |
|  | | | | | | | | | | | | 1 |  | BV |  | 25/03/2020 8:54 AM |  |
|  | | | Oh look – look don’t – no – thank you very much I appreciate that. People keep saying that to me and I keep saying I’m sorry too but I’m – I’m 81 years of age. I’ve had a pretty, brilliant, incredible life and now it’s time to pass on and leave my children in peace and you know - it won’t be a long while but that’s – that’s where I am. The only thing I – the only thing I’ve got no other choice in my own head. If you can come up with a choice like a cure that would be terrific but I know that’s not going to happen that’s why I’ve made this weird – although to me it’s not weird- | | | | | | | | | | | | | |  |
|  | | |  | | | | | | | | | | | | | |  |
|  | | | | | | | | | | | | 2 |  | BV |  | 25/03/2020 8:55 AM |  |
|  | | | certainly did. I make all my decisions by myself. I don’t have a partner. My – my ex-wife is still a live. She questions me a lot of course still. My kids are – you know putting me a third degree ringer and that’s okay. I’m used to being in charge. That’s the probably the best way. I’ve – I’ve always run my own companies and run my own – well I’ve got a wonderful family … strong family life and kids without any mental problems. I’ve got to the end of my life and I’ve – and we’ve all thought – we’ve had long discussions about this. Got to the end of my life saying well it’s been a very interesting one and it’s time to go. | | | | | | | | | | | | | |  |
|  | | |  | | | | | | | | | | | | | |  |
|  | | | | | | | | | | | | 3 |  | BV |  | 25/03/2020 8:55 AM |  |
|  | | | I – well in terms of deterioration of health and deterioration of being able to do anything, extreme tiredness, pain and just not being able to be myself. | | | | | | | | | | | | | |  |
|  | | |  | | | | | | | | | | | | | |  |
|  | | | | | | | | | | | | 4 |  | BV |  | 25/03/2020 8:56 AM |  |
|  | | | I – I’m – since I retired when I was 72 that was 9 years ago, when I sold my company I did my world trips, I did all these things, I’ve – in the last 8 years of that I was an ambassador at the Brisbane airport which I was very, very active there. I gave that away a year ago because I just don’t have the – the stamina any longer and the fortitude and that was the love of my life. I – I’ve been a singer for some 40 years in the Brisbane chorale and the Queensland choir and I had to give that away because my voice was going, I haven’t got the energy for that. I – in the last 4 years I was the chairman of the body corporate that I … in which I enjoyed immensely and because of the stop in – drop in stamina, energy I’ve had to give that away so the 3 things that kept me going I’ve had to give away and great joy I might add. I – now it – yeah I can’t do anything, I can’t do a thing. | | | | | | | | | | | | | |  |
|  |  |  |  |  |  |  |  |  |  |  |  |  |  |  |  |  |  |
|  | | |  | | | | | | | | | | | | | |  |
|  | | | | | | | | | | | | 5 |  | BV |  | 25/03/2020 8:56 AM |  |
|  | | | No I can’t say that no. The trial was very good. No I – look I don’t have a lot of money. I’ve got enough to keep me going. I’ve started to give a little bit of money away. I don’t have a little … to give away. It’s all basically planned when my kids – when I die my kids will only have to sell the house and I’ll have a bank account. I had a very small share trading account, that’s all been liquidated, it’s all been cashed up, very little for the solicitor to do except to … them to do. So it’s all – tried to control everything before the grave. | | | | | | | | | | | | | |  |
|  | | |  | | | | | | | | | | | | | |  |
|  | | | | | | | | | | | | | | | | | |
| Formatted Reports\\Coding Summary by File Formatted Report | | | | | | | | | | | Page 8 of 143 | | | | | | |
| 6/10/2022 8:14 AM | | | | | | | | | | | | | | | | | |
|  | | | **Classification** |  | **Aggregate** |  | **Coverage** |  | **Number Of Coding References** |  | | **Reference Number** |  | **Coded By Initials** |  | **Modified On** |  |
|  | | | | | | | | | | | | | | | | | |
|  | | | | | | | | | | | | 6 |  | BV |  | 25/03/2020 8:57 AM |  |
|  | | | No, no not at all. No well I’ve been – if you can stop me from being sick yes I would have loved that but there’s no way that it could be done by pills and if you could have given me all my energy back that – I would have kept going but no nothing – there was nothing that could keep me back on the trial. I mean I must admit I was disappointed when I had to say to Doctor Goh that I’m sorry mate I can’t keep going on this, my quality of life is – is down the gurgler. And if there was – there was basically nothing – he tried a couple of things but I can’t remember what they were for pain relief and but it’s the – it’s – instead of getting out of bed at 7 o’clock I was having trouble pushing myself out of bed at 8:30 or 9 o’clock apart from someone ringing me and saying I’m coming around to see you but - or I had to give away all my – the activities that I loved, my volunteer activities purely for sheer lack of stamina and I couldn’t see – oh sorry he couldn’t find anyway where I could get all my stamina back again. | | | | | | | | | | | | | |  |
|  |  |  |  |  |  |  |  |  |  |  |  |  |  |  |  |  |  |
|  | | |  | | | | | | | | | | | | | |  |
|  | | | **Codes\\x - removed from trial\facing mortality\lived a good life** | | | | | | | | | | | | | |  |
|  |  |  |  |  | No |  | 0.1374 |  | 2 |  | | | | | | |  |
|  | | |  |  |  |  |  |  |  |  | | | | | | | |
|  | | | | | | | | | | | | 1 |  | BV |  | 25/03/2020 8:54 AM |  |
|  | | | Oh look – look don’t – no – thank you very much I appreciate that. People keep saying that to me and I keep saying I’m sorry too but I’m – I’m 81 years of age. I’ve had a pretty, brilliant, incredible life and now it’s time to pass on and leave my children in peace and you know - it won’t be a long while but that’s – that’s where I am. The only thing I – the only thing I’ve got no other choice in my own head. If you can come up with a choice like a cure that would be terrific but I know that’s not going to happen that’s why I’ve made this weird – although to me it’s not weird- | | | | | | | | | | | | | |  |
|  | | |  | | | | | | | | | | | | | |  |
|  | | | | | | | | | | | | 2 |  | BV |  | 25/03/2020 8:56 AM |  |
|  | | | I – I’m – since I retired when I was 72 that was 9 years ago, when I sold my company I did my world trips, I did all these things, I’ve – in the last 8 years of that I was an ambassador at the Brisbane airport which I was very, very active there. I gave that away a year ago because I just don’t have the – the stamina any longer and the fortitude and that was the love of my life. I – I’ve been a singer for some 40 years in the Brisbane chorale and the Queensland choir and I had to give that away because my voice was going, I haven’t got the energy for that. I – in the last 4 years I was the chairman of the body corporate that I … in which I enjoyed immensely and because of the stop in – drop in stamina, energy I’ve had to give that away so the 3 things that kept me going I’ve had to give away and great joy I might add. I – now it – yeah I can’t do anything, I can’t do a thing. | | | | | | | | | | | | | |  |
|  |  |  |  |  |  |  |  |  |  |  |  |  |  |  |  |  |  |
|  | | |  | | | | | | | | | | | | | |  |
|  | | | **Codes\\x - removed from trial\facing mortality\placed self in palliative care** | | | | | | | | | | | | | |  |
|  |  |  |  |  | No |  | 0.4051 |  | 6 |  | | | | | | |  |
|  | | |  |  |  |  |  |  |  |  | | | | | | | |
|  | | | | | | | | | | | | 1 |  | BV |  | 25/03/2020 8:53 AM |  |
|  | | | I’ve actually been doing this for about – how long, some many, many years. I started with chemo, then with radiotherapy, then I started on clinical trials and then I started on other things like Zytiga and oh gosh I can’t remember. So many trials and I’ve had a very, very negative reaction basically to everything and in the end I’ve lost 18 kilos out of a 72 kilo body, that my body is been that way for the last 80 years and so I’ve lost so much weight and I just – I – mate I just said to Doctor Jeffrey Goh my oncologist, my body can’t take anymore. So I’ve just said please take me off all trials, all – everything, chemo every – everything and I just want to go into palliative care and die and because my quality of life is almost non-existent. So a week and a bit ago I booked myself into – after a few – lots of investigation by Prince Charles palliative care unit and they’ve booked me into here. I’ve been here now a week and a half and they’re trying to control my pain and my nausea and my eating and that’s where I am at the moment. | | | | | | | | | | | | | |  |
|  |  |  |  |  |  |  |  |  |  |  |  |  |  |  |  |  |  |
|  | | |  | | | | | | | | | | | | | |  |
|  | | | | | | | | | | | | 2 |  | BV |  | 25/03/2020 8:54 AM |  |
|  | | | Oh look – look don’t – no – thank you very much I appreciate that. People keep saying that to me and I keep saying I’m sorry too but I’m – I’m 81 years of age. I’ve had a pretty, brilliant, incredible life and now it’s time to pass on and leave my children in peace and you know - it won’t be a long while but that’s – that’s where I am. The only thing I – the only thing I’ve got no other choice in my own head. If you can come up with a choice like a cure that would be terrific but I know that’s not going to happen that’s why I’ve made this weird – although to me it’s not weird- | | | | | | | | | | | | | |  |
|  | | |  | | | | | | | | | | | | | |  |
|  | | | | | | | | | | | | 3 |  | BV |  | 25/03/2020 8:55 AM |  |
|  | | | certainly did. I make all my decisions by myself. I don’t have a partner. My – my ex-wife is still a live. She questions me a lot of course still. My kids are – you know putting me a third degree ringer and that’s okay. I’m used to being in charge. That’s the probably the best way. I’ve – I’ve always run my own companies and run my own – well I’ve got a wonderful family … strong family life and kids without any mental problems. I’ve got to the end of my life and I’ve – and we’ve all thought – we’ve had long discussions about this. Got to the end of my life saying well it’s been a very interesting one and it’s time to go. | | | | | | | | | | | | | |  |
|  | | |  | | | | | | | | | | | | | |  |
| Formatted Reports\\Coding Summary by File Formatted Report | | | | | | | | | | | Page 9 of 143 | | | | | | |
| 6/10/2022 8:14 AM | | | | | | | | | | | | | | | | | |
|  | | | **Classification** |  | **Aggregate** |  | **Coverage** |  | **Number Of Coding References** |  | | **Reference Number** |  | **Coded By Initials** |  | **Modified On** |  |
|  | | | | | | | | | | | | | | | | | |
|  | | | | | | | | | | | | 4 |  | BV |  | 25/03/2020 8:55 AM |  |
|  | | | I – well in terms of deterioration of health and deterioration of being able to do anything, extreme tiredness, pain and just not being able to be myself. | | | | | | | | | | | | | |  |
|  | | |  | | | | | | | | | | | | | |  |
|  | | | | | | | | | | | | 5 |  | BV |  | 25/03/2020 8:56 AM |  |
|  | | | I – I’m – since I retired when I was 72 that was 9 years ago, when I sold my company I did my world trips, I did all these things, I’ve – in the last 8 years of that I was an ambassador at the Brisbane airport which I was very, very active there. I gave that away a year ago because I just don’t have the – the stamina any longer and the fortitude and that was the love of my life. I – I’ve been a singer for some 40 years in the Brisbane chorale and the Queensland choir and I had to give that away because my voice was going, I haven’t got the energy for that. I – in the last 4 years I was the chairman of the body corporate that I … in which I enjoyed immensely and because of the stop in – drop in stamina, energy I’ve had to give that away so the 3 things that kept me going I’ve had to give away and great joy I might add. I – now it – yeah I can’t do anything, I can’t do a thing. | | | | | | | | | | | | | |  |
|  |  |  |  |  |  |  |  |  |  |  |  |  |  |  |  |  |  |
|  | | |  | | | | | | | | | | | | | |  |
|  | | | | | | | | | | | | 6 |  | BV |  | 25/03/2020 8:57 AM |  |
|  | | | No, no not at all. No well I’ve been – if you can stop me from being sick yes I would have loved that but there’s no way that it could be done by pills and if you could have given me all my energy back that – I would have kept going but no nothing – there was nothing that could keep me back on the trial. I mean I must admit I was disappointed when I had to say to Doctor Goh that I’m sorry mate I can’t keep going on this, my quality of life is – is down the gurgler. And if there was – there was basically nothing – he tried a couple of things but I can’t remember what they were for pain relief and but it’s the – it’s – instead of getting out of bed at 7 o’clock I was having trouble pushing myself out of bed at 8:30 or 9 o’clock apart from someone ringing me and saying I’m coming around to see you but - or I had to give away all my – the activities that I loved, my volunteer activities purely for sheer lack of stamina and I couldn’t see – oh sorry he couldn’t find anyway where I could get all my stamina back again. | | | | | | | | | | | | | |  |
|  |  |  |  |  |  |  |  |  |  |  |  |  |  |  |  |  |  |
|  | | |  | | | | | | | | | | | | | |  |
|  | | | **Codes\\x - removed from trial\financial impact** | | | | | | | | | | | | | |  |
|  |  |  |  |  | No |  | 0.0520 |  | 1 |  | | | | | | |  |
|  | | |  |  |  |  |  |  |  |  | | | | | | | |
|  | | | | | | | | | | | | 1 |  | BV |  | 25/03/2020 8:56 AM |  |
|  | | | No I can’t say that no. The trial was very good. No I – look I don’t have a lot of money. I’ve got enough to keep me going. I’ve started to give a little bit of money away. I don’t have a little … to give away. It’s all basically planned when my kids – when I die my kids will only have to sell the house and I’ll have a bank account. I had a very small share trading account, that’s all been liquidated, it’s all been cashed up, very little for the solicitor to do except to … them to do. So it’s all – tried to control everything before the grave. | | | | | | | | | | | | | |  |
|  | | |  | | | | | | | | | | | | | |  |
|  | | | **Codes\\x - removed from trial\quality of trialists** | | | | | | | | | | | | | |  |
|  |  |  |  |  | No |  | 0.0091 |  | 1 |  | | | | | | |  |
|  | | |  |  |  |  |  |  |  |  | | | | | | | |
|  | | | | | | | | | | | | 1 |  | BV |  | 25/03/2020 8:57 AM |  |
|  | | | I must admit I’m – I’m really incredibly pleased with the things they do here at Prince Charles. | | | | | | | | | | | | | |  |
|  | | |  | | | | | | | | | | | | | |  |
|  | | | **Codes\\x - removed from trial\sceptical** | | | | | | | | | | | | | |  |
|  |  |  |  |  | No |  | 0.1127 |  | 2 |  | | | | | | |  |
|  | | |  |  |  |  |  |  |  |  | | | | | | | |
|  | | | | | | | | | | | | 1 |  | BV |  | 25/03/2020 8:58 AM |  |
|  | | | No well I’ve been – if you can stop me from being sick yes I would have loved that but there’s no way that it could be done by pills and if you could have given me all my energy back that – I would have kept going but no nothing – there was nothing that could keep me back on the trial. | | | | | | | | | | | | | |  |
|  | | |  | | | | | | | | | | | | | |  |
|  | | | | | | | | | | | | | | | | | |
| Formatted Reports\\Coding Summary by File Formatted Report | | | | | | | | | | | Page 10 of 143 | | | | | | |
| 6/10/2022 8:14 AM | | | | | | | | | | | | | | | | | |
|  | | | **Classification** |  | **Aggregate** |  | **Coverage** |  | **Number Of Coding References** |  | | **Reference Number** |  | **Coded By Initials** |  | **Modified On** |  |
|  | | | | | | | | | | | | | | | | | |
|  | | | | | | | | | | | | 2 |  | BV |  | 25/03/2020 8:58 AM |  |
|  | | | But then I was – he said righto well I’ll put you on a choice of Lutetium or this other one but unfortunately the computer makes the decision and I said well I … go on now that I’ve read all the documents because as you know are quite extensive. I read everything incidentally. I read everything that’s given to me and I saw this and I went so one is a choice of 2 … that could go wrong and the other thing is a choice of everything that I’ve been going wrong with me now plus even more and when the – when the computer came up and said no, you’ve been chosen on the second one I said no, no, no that’s not on. I – my body physically cannot take anymore. I’m not talking about mentally, I’m talking about physically. I’d be – I’d have a nurse in my room at my bedside all day long and I did not want that. Have you had a look at the trial – well you’d know the side effects of both of those particular trials no doubt? | | | | | | | | | | | | | |  |
|  |  |  |  |  |  |  |  |  |  |  |  |  |  |  |  |  |  |
|  | | |  | | | | | | | | | | | | | |  |
|  | | | **Codes\\x - removed from trial\sceptical\randomisation** | | | | | | | | | | | | | |  |
|  |  |  |  |  | No |  | 0.0861 |  | 1 |  | | | | | | |  |
|  | | |  |  |  |  |  |  |  |  | | | | | | | |
|  | | | | | | | | | | | | 1 |  | BV |  | 25/03/2020 8:58 AM |  |
|  | | | But then I was – he said righto well I’ll put you on a choice of Lutetium or this other one but unfortunately the computer makes the decision and I said well I … go on now that I’ve read all the documents because as you know are quite extensive. I read everything incidentally. I read everything that’s given to me and I saw this and I went so one is a choice of 2 … that could go wrong and the other thing is a choice of everything that I’ve been going wrong with me now plus even more and when the – when the computer came up and said no, you’ve been chosen on the second one I said no, no, no that’s not on. I – my body physically cannot take anymore. I’m not talking about mentally, I’m talking about physically. I’d be – I’d have a nurse in my room at my bedside all day long and I did not want that. Have you had a look at the trial – well you’d know the side effects of both of those particular trials no doubt? | | | | | | | | | | | | | |  |
|  |  |  |  |  |  |  |  |  |  |  |  |  |  |  |  |  |  |
|  | | |  | | | | | | | | | | | | | |  |
|  | | | **Codes\\x - removed from trial\side effects\side effects - cabazitaxel** | | | | | | | | | | | | | |  |
|  |  |  |  |  | No |  | 0.1291 |  | 4 |  | | | | | | |  |
|  | | |  |  |  |  |  |  |  |  | | | | | | | |
|  | | | | | | | | | | | | 1 |  | BV |  | 25/03/2020 8:54 AM |  |
|  | | | Very, very tired. I was very nauseated and in and out of pain but that’s where I am. | | | | | | | | | | | | | |  |
|  | | |  | | | | | | | | | | | | | |  |
|  | | | | | | | | | | | | 2 |  | BV |  | 25/03/2020 8:56 AM |  |
|  | | | I – well in terms of deterioration of health and deterioration of being able to do anything, extreme tiredness, pain and just not being able to be myself. | | | | | | | | | | | | | |  |
|  | | |  | | | | | | | | | | | | | |  |
|  | | | | | | | | | | | | 3 |  | BV |  | 25/03/2020 8:58 AM |  |
|  | | | one had side effects of about 4 things and the other one had side effects of about 54 things and when I was chosen for the second one which had the multiple side effects my heart plummeted and I went no, no I can’t do this. | | | | | | | | | | | | | |  |
|  | | |  | | | | | | | | | | | | | |  |
|  | | | | | | | | | | | | 4 |  | BV |  | 25/03/2020 8:58 AM |  |
|  | | | But then I was – he said righto well I’ll put you on a choice of Lutetium or this other one but unfortunately the computer makes the decision and I said well I … go on now that I’ve read all the documents because as you know are quite extensive. I read everything incidentally. I read everything that’s given to me and I saw this and I went so one is a choice of 2 … that could go wrong and the other thing is a choice of everything that I’ve been going wrong with me now plus even more and when the – when the computer came up and said no, you’ve been chosen on the second one I said no, no, no that’s not on. I – my body physically cannot take anymore. I’m not talking about mentally, I’m talking about physically. I’d be – I’d have a nurse in my room at my bedside all day long and I did not want that. Have you had a look at the trial – well you’d know the side effects of both of those particular trials no doubt? | | | | | | | | | | | | | |  |
|  |  |  |  |  |  |  |  |  |  |  |  |  |  |  |  |  |  |
|  | | |  | | | | | | | | | | | | | |  |
|  | | | | | | | | | | | | | | | | | |
| Formatted Reports\\Coding Summary by File Formatted Report | | | | | | | | | | | Page 11 of 143 | | | | | | |
| 6/10/2022 8:14 AM | | | | | | | | | | | | | | | | | |
|  | | | **Classification** |  | **Aggregate** |  | **Coverage** |  | **Number Of Coding References** |  | | **Reference Number** |  | **Coded By Initials** |  | **Modified On** |  |
|  | | | **Codes\\x - removed from trial\struggling with treatment** | | | | | | | | | | | | | |  |
|  |  |  |  |  | No |  | 0.2989 |  | 5 |  | | | | | | |  |
|  | | |  |  |  |  |  |  |  |  | | | | | | | |
|  | | | | | | | | | | | | 1 |  | BV |  | 25/03/2020 8:53 AM |  |
|  | | | I’ve actually been doing this for about – how long, some many, many years. I started with chemo, then with radiotherapy, then I started on clinical trials and then I started on other things like Zytiga and oh gosh I can’t remember. So many trials and I’ve had a very, very negative reaction basically to everything and in the end I’ve lost 18 kilos out of a 72 kilo body, that my body is been that way for the last 80 years and so I’ve lost so much weight and I just – I – mate I just said to Doctor Jeffrey Goh my oncologist, my body can’t take anymore. So I’ve just said please take me off all trials, all – everything, chemo every – everything and I just want to go into palliative care and die and because my quality of life is almost non-existent. So a week and a bit ago I booked myself into – after a few – lots of investigation by Prince Charles palliative care unit and they’ve booked me into here. I’ve been here now a week and a half and they’re trying to control my pain and my nausea and my eating and that’s where I am at the moment. | | | | | | | | | | | | | |  |
|  |  |  |  |  |  |  |  |  |  |  |  |  |  |  |  |  |  |
|  | | |  | | | | | | | | | | | | | |  |
|  | | | | | | | | | | | | 2 |  | BV |  | 25/03/2020 8:54 AM |  |
|  | | | Very, very tired. I was very nauseated and in and out of pain but that’s where I am. | | | | | | | | | | | | | |  |
|  | | |  | | | | | | | | | | | | | |  |
|  | | | | | | | | | | | | 3 |  | BV |  | 25/03/2020 8:55 AM |  |
|  | | | I – well in terms of deterioration of health and deterioration of being able to do anything, extreme tiredness, pain and just not being able to be myself. | | | | | | | | | | | | | |  |
|  | | |  | | | | | | | | | | | | | |  |
|  | | | | | | | | | | | | 4 |  | BV |  | 25/03/2020 8:56 AM |  |
|  | | | I – I’m – since I retired when I was 72 that was 9 years ago, when I sold my company I did my world trips, I did all these things, I’ve – in the last 8 years of that I was an ambassador at the Brisbane airport which I was very, very active there. I gave that away a year ago because I just don’t have the – the stamina any longer and the fortitude and that was the love of my life. I – I’ve been a singer for some 40 years in the Brisbane chorale and the Queensland choir and I had to give that away because my voice was going, I haven’t got the energy for that. I – in the last 4 years I was the chairman of the body corporate that I … in which I enjoyed immensely and because of the stop in – drop in stamina, energy I’ve had to give that away so the 3 things that kept me going I’ve had to give away and great joy I might add. I – now it – yeah I can’t do anything, I can’t do a thing. | | | | | | | | | | | | | |  |
|  |  |  |  |  |  |  |  |  |  |  |  |  |  |  |  |  |  |
|  | | |  | | | | | | | | | | | | | |  |
|  | | | | | | | | | | | | 5 |  | BV |  | 25/03/2020 8:57 AM |  |
|  | | | No, no not at all. No well I’ve been – if you can stop me from being sick yes I would have loved that but there’s no way that it could be done by pills and if you could have given me all my energy back that – I would have kept going but no nothing – there was nothing that could keep me back on the trial. I mean I must admit I was disappointed when I had to say to Doctor Goh that I’m sorry mate I can’t keep going on this, my quality of life is – is down the gurgler. And if there was – there was basically nothing – he tried a couple of things but I can’t remember what they were for pain relief and but it’s the – it’s – instead of getting out of bed at 7 o’clock I was having trouble pushing myself out of bed at 8:30 or 9 o’clock apart from someone ringing me and saying I’m coming around to see you but - or I had to give away all my – the activities that I loved, my volunteer activities purely for sheer lack of stamina and I couldn’t see – oh sorry he couldn’t find anyway where I could get all my stamina back again. | | | | | | | | | | | | | |  |
|  |  |  |  |  |  |  |  |  |  |  |  |  |  |  |  |  |  |
|  | | |  | | | | | | | | | | | | | |  |
|  | | | **Codes\\x - removed from trial\struggling with treatment\stopped all treatment and trials** | | | | | | | | | | | | | |  |
|  |  |  |  |  | No |  | 0.1993 |  | 3 |  | | | | | | |  |
|  | | |  |  |  |  |  |  |  |  | | | | | | | |
|  | | | | | | | | | | | | 1 |  | BV |  | 25/03/2020 8:53 AM |  |
|  | | | No I’ve actually stopped the trial, I’ve stopped all trials. | | | | | | | | | | | | | |  |
|  | | |  | | | | | | | | | | | | | |  |
|  | | | | | | | | | | | | 2 |  | BV |  | 25/03/2020 8:53 AM |  |
|  | | | I’ve actually been doing this for about – how long, some many, many years. I started with chemo, then with radiotherapy, then I started on clinical trials and then I started on other things like Zytiga and oh gosh I can’t remember. So many trials and I’ve had a very, very negative reaction basically to everything and in the end I’ve lost 18 kilos out of a 72 kilo body, that my body is been that way for the last 80 years and so I’ve lost so much weight and I just – I – mate I just said to Doctor Jeffrey Goh my oncologist, my body can’t take anymore. So I’ve just said please take me off all trials, all – everything, chemo every – everything and I just want to go into palliative care and die and because my quality of life is almost non-existent. So a week and a bit ago I booked myself into – after a few – lots of investigation by Prince Charles palliative care unit and they’ve booked me into here. I’ve been here now a week and a half and they’re trying to control my pain and my nausea and my eating and that’s where I am at the moment. | | | | | | | | | | | | | |  |
|  |  |  |  |  |  |  |  |  |  |  |  |  |  |  |  |  |  |
|  | | |  | | | | | | | | | | | | | |  |
| Formatted Reports\\Coding Summary by File Formatted Report | | | | | | | | | | | Page 12 of 143 | | | | | | |
| 6/10/2022 8:14 AM | | | | | | | | | | | | | | | | | |
|  | | | **Classification** |  | **Aggregate** |  | **Coverage** |  | **Number Of Coding References** |  | | **Reference Number** |  | **Coded By Initials** |  | **Modified On** |  |
|  | | | | | | | | | | | | | | | | | |
|  | | | | | | | | | | | | 3 |  | BV |  | 25/03/2020 8:57 AM |  |
|  | | | No, no not at all. No well I’ve been – if you can stop me from being sick yes I would have loved that but there’s no way that it could be done by pills and if you could have given me all my energy back that – I would have kept going but no nothing – there was nothing that could keep me back on the trial. I mean I must admit I was disappointed when I had to say to Doctor Goh that I’m sorry mate I can’t keep going on this, my quality of life is – is down the gurgler. And if there was – there was basically nothing – he tried a couple of things but I can’t remember what they were for pain relief and but it’s the – it’s – instead of getting out of bed at 7 o’clock I was having trouble pushing myself out of bed at 8:30 or 9 o’clock apart from someone ringing me and saying I’m coming around to see you but - or I had to give away all my – the activities that I loved, my volunteer activities purely for sheer lack of stamina and I couldn’t see – oh sorry he couldn’t find anyway where I could get all my stamina back again. | | | | | | | | | | | | | |  |
|  |  |  |  |  |  |  |  |  |  |  |  |  |  |  |  |  |  |
|  | | |  | | | | | | | | | | | | | |  |
|  | **Files\\Transcription 2_002** | | | | | | | | | | | | | | | |  |
|  | | **Code** | | | | | | | | | | | | | | |  |
|  | | | **Codes\\coping strategies\coping okay** | | | | | | | | | | | | | |  |
|  |  |  |  |  | No |  | 0.0023 |  | 1 |  | | | | | | |  |
|  | | |  |  |  |  |  |  |  |  | | | | | | | |
|  | | | | | | | | | | | | 1 |  | BV |  | 10/01/2020 10:18 AM |  |
|  | | | I’m coping okay. | | | | | | | | | | | | | |  |
|  | | |  | | | | | | | | | | | | | |  |
|  | | | **Codes\\financial impact\no affect to finances** | | | | | | | | | | | | | |  |
|  |  |  |  |  | No |  | 0.0070 |  | 1 |  | | | | | | |  |
|  | | |  |  |  |  |  |  |  |  | | | | | | | |
|  | | | | | | | | | | | | 1 |  | BV |  | 10/01/2020 10:18 AM |  |
|  | | | No – no I don’t have any problems with finances | | | | | | | | | | | | | |  |
|  | | |  | | | | | | | | | | | | | |  |
|  | | | **Codes\\going back and forth to the hospital** | | | | | | | | | | | | | |  |
|  |  |  |  |  | No |  | 0.0153 |  | 1 |  | | | | | | |  |
|  | | |  |  |  |  |  |  |  |  | | | | | | | |
|  | | | | | | | | | | | | 1 |  | BV |  | 10/01/2020 10:06 AM |  |
|  | | | Yeah no, no it’s – well it’s going back and forward to the hospitals every 3 weeks is – is not much fun | | | | | | | | | | | | | |  |
|  | | |  | | | | | | | | | | | | | |  |
|  | | | **Codes\\initial motivation for participation** | | | | | | | | | | | | | |  |
|  |  |  |  |  | No |  | 0.0872 |  | 3 |  | | | | | | |  |
|  | | |  |  |  |  |  |  |  |  | | | | | | | |
|  | | | | | | | | | | | | 1 |  | BV |  | 10/01/2020 10:20 AM |  |
|  | | | Q: What about – alright let’s have a look at. When you were – thinking back on what your initial motivations were for participating in this trial. Has anything changed?  A: No certainly not – no. | | | | | | | | | | | | | |  |
|  | | |  | | | | | | | | | | | | | |  |
| Formatted Reports\\Coding Summary by File Formatted Report | | | | | | | | | | | Page 13 of 143 | | | | | | |
| 6/10/2022 8:14 AM | | | | | | | | | | | | | | | | | |
|  | | | **Classification** |  | **Aggregate** |  | **Coverage** |  | **Number Of Coding References** |  | | **Reference Number** |  | **Coded By Initials** |  | **Modified On** |  |
|  | | | | | | | | | | | | | | | | | |
|  | | | | | | | | | | | | 2 |  | BV |  | 10/01/2020 10:19 AM |  |
|  | | | Well sort of running out of options. | | | | | | | | | | | | | |  |
|  | | |  | | | | | | | | | | | | | |  |
|  | | | | | | | | | | | | 3 |  | BV |  | 10/01/2020 10:19 AM |  |
|  | | | I have had – had chemotherapy, then I’ve had another – another drug. And they work for a little a while, but they don’t work anymore. So it was either – the oncologist told me that probably the next step would have been Cabazitaxel. There were really nothing after that. So I seen that trial and thought well I’ll do it again – that’s all. | | | | | | | | | | | | | |  |
|  | | |  | | | | | | | | | | | | | |  |
|  | | | **Codes\\initial motivation for participation\changes to initial motivation for participation** | | | | | | | | | | | | | |  |
|  |  |  |  |  | No |  | 0.0298 |  | 1 |  | | | | | | |  |
|  | | |  |  |  |  |  |  |  |  | | | | | | | |
|  | | | | | | | | | | | | 1 |  | BV |  | 10/01/2020 10:20 AM |  |
|  | | | Q: What about – alright let’s have a look at. When you were – thinking back on what your initial motivations were for participating in this trial. Has anything changed?  A: No certainly not – no. | | | | | | | | | | | | | |  |
|  | | |  | | | | | | | | | | | | | |  |
|  | | | **Codes\\quality of trialists** | | | | | | | | | | | | | |  |
|  |  |  |  |  | No |  | 0.0257 |  | 2 |  | | | | | | |  |
|  | | |  |  |  |  |  |  |  |  | | | | | | | |
|  | | | | | | | | | | | | 1 |  | BV |  | 10/01/2020 10:06 AM |  |
|  | | | People have – people are all very nice and yeah I can’t complain about it at all. It’s – it’s quite … put it that way – yeah it’s good. | | | | | | | | | | | | | |  |
|  | | |  | | | | | | | | | | | | | |  |
|  | | | | | | | | | | | | 2 |  | BV |  | 10/01/2020 10:12 AM |  |
|  | | | Everybody seems to be helpful there. | | | | | | | | | | | | | |  |
|  | | |  | | | | | | | | | | | | | |  |
|  | | | **Codes\\side effects** | | | | | | | | | | | | | |  |
|  |  |  |  |  | No |  | 0.0179 |  | 1 |  | | | | | | |  |
|  | | |  |  |  |  |  |  |  |  | | | | | | | |
|  | | | | | | | | | | | | 1 |  | BV |  | 25/03/2020 8:26 AM |  |
|  | | | It’s – doesn’t seem to have hardly any side effects, just really – just a dry mouth and a little bit of fatigue I guess. | | | | | | | | | | | | | |  |
|  | | |  | | | | | | | | | | | | | |  |
|  | | | **Codes\\side effects\side effects - lutetium** | | | | | | | | | | | | | |  |
|  |  |  |  |  | No |  | 0.0179 |  | 1 |  | | | | | | |  |
|  | | |  |  |  |  |  |  |  |  | | | | | | | |
|  | | | | | | | | | | | | 1 |  | BV |  | 10/01/2020 10:07 AM |  |
|  | | | It’s – doesn’t seem to have hardly any side effects, just really – just a dry mouth and a little bit of fatigue I guess. | | | | | | | | | | | | | |  |
|  | | |  | | | | | | | | | | | | | |  |
| Formatted Reports\\Coding Summary by File Formatted Report | | | | | | | | | | | Page 14 of 143 | | | | | | |
| 6/10/2022 8:14 AM | | | | | | | | | | | | | | | | | |
|  | | | **Classification** |  | **Aggregate** |  | **Coverage** |  | **Number Of Coding References** |  | | **Reference Number** |  | **Coded By Initials** |  | **Modified On** |  |
|  | | | **Codes\\treatments been good** | | | | | | | | | | | | | |  |
|  |  |  |  |  | No |  | 0.0313 |  | 3 |  | | | | | | |  |
|  | | |  |  |  |  |  |  |  |  | | | | | | | |
|  | | | | | | | | | | | | 1 |  | BV |  | 10/01/2020 10:07 AM |  |
|  | | | It’s – it’s quite … put it that way – yeah it’s good. | | | | | | | | | | | | | |  |
|  | | |  | | | | | | | | | | | | | |  |
|  | | | | | | | | | | | | 2 |  | BV |  | 10/01/2020 10:07 AM |  |
|  | | | Seems to be working pretty good. | | | | | | | | | | | | | |  |
|  | | |  | | | | | | | | | | | | | |  |
|  | | | | | | | | | | | | 3 |  | BV |  | 10/01/2020 10:19 AM |  |
|  | | | I couldn’t just - everything seems to be going according to …. It’s – it’s good, it’s working and - no it’s great. | | | | | | | | | | | | | |  |
|  | | |  | | | | | | | | | | | | | |  |
|  | **Files\\Transcription 2_003** | | | | | | | | | | | | | | | |  |
|  | | **Code** | | | | | | | | | | | | | | |  |
|  | | | **Codes\\emotional impact** | | | | | | | | | | | | | |  |
|  |  |  |  |  | No |  | 0.0949 |  | 2 |  | | | | | | |  |
|  | | |  |  |  |  |  |  |  |  | | | | | | | |
|  | | | | | | | | | | | | 1 |  | BV |  | 15/01/2020 5:24 AM |  |
|  | | | I: Umm, has anything, your participation in the trial affected you physically or psychologically?  P: No. No. Not at all. Umm, umm, I suppose the only thing that you do think about is, it is a radioactive tracer for the first couple of days you know, you cant really, sort of, spend a lot of time with the quality members, cos I have a granddaughter, you know, they say, you know, just don’t stay with them for long periods of time but a part from that no, it’s really affected me no other way. It’s been really good. | | | | | | | | | | | | | |  |
|  | | |  | | | | | | | | | | | | | |  |
|  | | | | | | | | | | | | 2 |  | BV |  | 15/01/2020 4:53 AM |  |
|  | | | I think it’s a case of, like anything, you know, you have your days when you’re a little bit down and you have days where your back up, you know, but you’ve got to stay strong and fight all the way through otherwise, it means giving up. | | | | | | | | | | | | | |  |
|  | | |  | | | | | | | | | | | | | |  |
|  | | | **Codes\\financial impact\no affect to finances** | | | | | | | | | | | | | |  |
|  |  |  |  |  | No |  | 0.0047 |  | 1 |  | | | | | | |  |
|  | | |  |  |  |  |  |  |  |  | | | | | | | |
|  | | | | | | | | | | | | 1 |  | BV |  | 14/01/2020 10:50 AM |  |
|  | | | No finances. That’s always been good. | | | | | | | | | | | | | |  |
|  | | |  | | | | | | | | | | | | | |  |
|  | | | | | | | | | | | | | | | | | |
| Formatted Reports\\Coding Summary by File Formatted Report | | | | | | | | | | | Page 15 of 143 | | | | | | |
| 6/10/2022 8:14 AM | | | | | | | | | | | | | | | | | |
|  | | | **Classification** |  | **Aggregate** |  | **Coverage** |  | **Number Of Coding References** |  | | **Reference Number** |  | **Coded By Initials** |  | **Modified On** |  |
|  | | | **Codes\\happy about participating** | | | | | | | | | | | | | |  |
|  |  |  |  |  | No |  | 0.0132 |  | 1 |  | | | | | | |  |
|  | | |  |  |  |  |  |  |  |  | | | | | | | |
|  | | | | | | | | | | | | 1 |  | BV |  | 14/01/2020 10:59 AM |  |
|  | | | I: So, your quite happy that you’re on the trial and you’re receiving the new treatment?  P: Definitely. | | | | | | | | | | | | | |  |
|  | | |  | | | | | | | | | | | | | |  |
|  | | | **Codes\\initial motivation for participation** | | | | | | | | | | | | | |  |
|  |  |  |  |  | No |  | 0.1016 |  | 2 |  | | | | | | |  |
|  | | |  |  |  |  |  |  |  |  | | | | | | | |
|  | | | | | | | | | | | | 1 |  | BV |  | 14/01/2020 10:57 AM |  |
|  | | | Umm, well it wasn’t my motivation, it was umm, my, the lady who was looking after me at the hospital | | | | | | | | | | | | | |  |
|  | | |  | | | | | | | | | | | | | |  |
|  | | | | | | | | | | | | 2 |  | BV |  | 14/01/2020 10:59 AM |  |
|  | | | my PSA started to rise again and I was, a couple of years ago, I had a dose of ….taxel and that seemed to cool me down for a couple of years and then my PSA started to jump again and they tried the Docetaxel umm and it wasn’t working umm and umm she said to me look I’m not going to give you the next lot, which is Cabazitaxel, give you that, there’s a trial at the Royal Hospital that will exclude you because their doing a blind trial on Cabazitaxel and the Lu- PSMA. So, umm she was the one that actually referred me to it so yeah, she is obviously up with all of the cancer things, so she was fantastic.  I: Ok. So, did you initially enrol because your doctor made that suggestion?  P: Yes definitely. | | | | | | | | | | | | | |  |
|  |  |  |  |  |  |  |  |  |  |  |  |  |  |  |  |  |  |
|  | | |  | | | | | | | | | | | | | |  |
|  | | | **Codes\\initial motivation for participation\doctors recommendation** | | | | | | | | | | | | | |  |
|  |  |  |  |  | No |  | 0.0766 |  | 3 |  | | | | | | |  |
|  | | |  |  |  |  |  |  |  |  | | | | | | | |
|  | | | | | | | | | | | | 1 |  | BV |  | 14/01/2020 10:57 AM |  |
|  | | | Umm, well it wasn’t my motivation, it was umm, my, the lady who was looking after me at the hospital | | | | | | | | | | | | | |  |
|  | | |  | | | | | | | | | | | | | |  |
|  | | | | | | | | | | | | 2 |  | BV |  | 14/01/2020 10:59 AM |  |
|  | | | I: Ok. So, did you initially enrol because your doctor made that suggestion?  P: Yes definitely. | | | | | | | | | | | | | |  |
|  | | |  | | | | | | | | | | | | | |  |
|  | | | | | | | | | | | | 3 |  | BV |  | 14/01/2020 10:59 AM |  |
|  | | | No well, umm, she recommended it because she said, if you get up to the Lutieum stage, its very good, it’s umm, had some good results even though it’s only, their only doing it on a trial over there it’s had some good results so yeah, she said its in my best interests not to have the treatment now, let me PSA get up over 20 and get over there and get onto it and do that trial first before we do anything else. | | | | | | | | | | | | | |  |
|  | | |  | | | | | | | | | | | | | |  |
|  | | | **Codes\\initial motivation for participation\failure in previous treatment** | | | | | | | | | | | | | |  |
|  |  |  |  |  | No |  | 0.1110 |  | 2 |  | | | | | | |  |
|  | | |  |  |  |  |  |  |  |  | | | | | | | |
|  | | | | | | | | | | | | 1 |  | BV |  | 14/01/2020 10:36 AM |  |
|  | | | Umm from I suppose, the big difference I noticed is the, umm, its not as intrusive as some other chemo’s so I was very lucky with chemo, I wasn’t sick and all those sorts of things but I, I, my hair did start to fall out so you know, it was coming out in clumps you know. | | | | | | | | | | | | | |  |
|  | | |  | | | | | | | | | | | | | |  |
| Formatted Reports\\Coding Summary by File Formatted Report | | | | | | | | | | | Page 16 of 143 | | | | | | |
| 6/10/2022 8:14 AM | | | | | | | | | | | | | | | | | |
|  | | | **Classification** |  | **Aggregate** |  | **Coverage** |  | **Number Of Coding References** |  | | **Reference Number** |  | **Coded By Initials** |  | **Modified On** |  |
|  | | | | | | | | | | | | | | | | | |
|  | | | | | | | | | | | | 2 |  | BV |  | 14/01/2020 10:59 AM |  |
|  | | | my PSA started to rise again and I was, a couple of years ago, I had a dose of ….taxel and that seemed to cool me down for a couple of years and then my PSA started to jump again and they tried the Docetaxel umm and it wasn’t working umm and umm she said to me look I’m not going to give you the next lot, which is Cabazitaxel, give you that, there’s a trial at the Royal Hospital that will exclude you because their doing a blind trial on Cabazitaxel and the Lu- PSMA. So, umm she was the one that actually referred me to it so yeah, she is obviously up with all of the cancer things, so she was fantastic. | | | | | | | | | | | | | |  |
|  | | |  | | | | | | | | | | | | | |  |
|  | | | **Codes\\keep battling** | | | | | | | | | | | | | |  |
|  |  |  |  |  | No |  | 0.0367 |  | 2 |  | | | | | | |  |
|  | | |  |  |  |  |  |  |  |  | | | | | | | |
|  | | | | | | | | | | | | 1 |  | BV |  | 15/01/2020 4:53 AM |  |
|  | | | I think it’s a case of, like anything, you know, you have your days when you’re a little bit down and you have days where your back up, you know, but you’ve got to stay strong and fight all the way through otherwise, it means giving up. | | | | | | | | | | | | | |  |
|  | | |  | | | | | | | | | | | | | |  |
|  | | | | | | | | | | | | 2 |  | BV |  | 14/01/2020 10:50 AM |  |
|  | | | You’ve got to keep battling so I’m pretty solid in that. | | | | | | | | | | | | | |  |
|  | | |  | | | | | | | | | | | | | |  |
|  | | | **Codes\\needing better communication from doctor** | | | | | | | | | | | | | |  |
|  |  |  |  |  | No |  | 0.0544 |  | 2 |  | | | | | | |  |
|  | | |  |  |  |  |  |  |  |  | | | | | | | |
|  | | | | | | | | | | | | 1 |  | BV |  | 14/01/2020 10:46 AM |  |
|  | | | you know, look I understand there are a lot of people there and it’s very busy but yeah sometimes it’s just a case of saying “oh look, it’s going to be an hour or an hour and a half” and they just sort of make you sit there sort of thing, you know what I mean? | | | | | | | | | | | | | |  |
|  | | |  | | | | | | | | | | | | | |  |
|  | | | | | | | | | | | | 2 |  | BV |  | 14/01/2020 10:52 AM |  |
|  | | | I suppose the only thing, sometimes the doctors talk in their own jargon umm, and they get pretty, sometimes you’ve got to slow them down, talk to them for a bit, umm yeah. | | | | | | | | | | | | | |  |
|  | | |  | | | | | | | | | | | | | |  |
|  | | | **Codes\\not as intrusive as chemo** | | | | | | | | | | | | | |  |
|  |  |  |  |  | No |  | 0.0747 |  | 3 |  | | | | | | |  |
|  | | |  |  |  |  |  |  |  |  | | | | | | | |
|  | | | | | | | | | | | | 1 |  | BV |  | 15/01/2020 4:57 AM |  |
|  | | | Umm from I suppose, the big difference I noticed is the, umm, its not as intrusive as some other chemo’s so I was very lucky with chemo, I wasn’t sick and all those sorts of things but I, I, my hair did start to fall out so you know, it was coming out in clumps you know. Whereas with the Lutieum, I’ve had none of that. | | | | | | | | | | | | | |  |
|  | | |  | | | | | | | | | | | | | |  |
|  | | | | | | | | | | | | 2 |  | BV |  | 14/01/2020 10:37 AM |  |
|  | | | My appetite’s good, you know, everything is really good about it and umm, its umm, a much quicker process than going through chemo so… | | | | | | | | | | | | | |  |
|  | | |  | | | | | | | | | | | | | |  |
|  | | | | | | | | | | | | 3 |  | BV |  | 14/01/2020 10:37 AM |  |
|  | | | Its umm, yeah, its quite umm, I shouldn’t say pleasant but its umm, a hell of a lot better than the standard chemo process that’s for sure | | | | | | | | | | | | | |  |
|  | | |  | | | | | | | | | | | | | |  |
| Formatted Reports\\Coding Summary by File Formatted Report | | | | | | | | | | | Page 17 of 143 | | | | | | |
| 6/10/2022 8:14 AM | | | | | | | | | | | | | | | | | |
|  | | | **Classification** |  | **Aggregate** |  | **Coverage** |  | **Number Of Coding References** |  | | **Reference Number** |  | **Coded By Initials** |  | **Modified On** |  |
|  | | | **Codes\\quality of life changes** | | | | | | | | | | | | | |  |
|  |  |  |  |  | No |  | 0.0051 |  | 1 |  | | | | | | |  |
|  | | |  |  |  |  |  |  |  |  | | | | | | | |
|  | | | | | | | | | | | | 1 |  | BV |  | 25/03/2020 8:25 AM |  |
|  | | | No quality of life issues, definitely no. | | | | | | | | | | | | | |  |
|  | | |  | | | | | | | | | | | | | |  |
|  | | | **Codes\\quality of life changes\quality of life changes - lutetium** | | | | | | | | | | | | | |  |
|  |  |  |  |  | No |  | 0.0051 |  | 1 |  | | | | | | |  |
|  | | |  |  |  |  |  |  |  |  | | | | | | | |
|  | | | | | | | | | | | | 1 |  | BV |  | 14/01/2020 10:48 AM |  |
|  | | | No quality of life issues, definitely no. | | | | | | | | | | | | | |  |
|  | | |  | | | | | | | | | | | | | |  |
|  | | | **Codes\\quality of trialists** | | | | | | | | | | | | | |  |
|  |  |  |  |  | No |  | 0.0963 |  | 4 |  | | | | | | |  |
|  | | |  |  |  |  |  |  |  |  | | | | | | | |
|  | | | | | | | | | | | | 1 |  | BV |  | 14/01/2020 10:38 AM |  |
|  | | | Umm, I don’t know about helpful but umm, I must admit, like I said, when I was at the PA, the people were fantastic and umm, everyone here at the Royal has been just as good. | | | | | | | | | | | | | |  |
|  | | |  | | | | | | | | | | | | | |  |
|  | | | | | | | | | | | | 2 |  | BV |  | 14/01/2020 10:38 AM |  |
|  | | | The actual level of care is just amazing, and people are just fantastic at what they do you know umm, that’s the big thing that sticks out to me is that it seems no matter where you go the people are just amazing you know. | | | | | | | | | | | | | |  |
|  | | |  | | | | | | | | | | | | | |  |
|  | | | | | | | | | | | | 3 |  | BV |  | 14/01/2020 10:38 AM |  |
|  | | | They would do anything to just help you... are you hungry? Do you want a drink? Something to eat? You know, just, they look after you. Are you comfortable? Tell us this, tell us that. So, they have been fantastic. The whole lot. | | | | | | | | | | | | | |  |
|  | | |  | | | | | | | | | | | | | |  |
|  | | | | | | | | | | | | 4 |  | BV |  | 14/01/2020 10:52 AM |  |
|  | | | Umm, to be honest with you no. It’s been pretty good. Like I said, the umm, the doctors have been good, umm, the nurses have been fantastic | | | | | | | | | | | | | |  |
|  | | |  | | | | | | | | | | | | | |  |
|  | | | **Codes\\side effects** | | | | | | | | | | | | | |  |
|  |  |  |  |  | No |  | 0.1400 |  | 4 |  | | | | | | |  |
|  | | |  |  |  |  |  |  |  |  | | | | | | | |
|  | | | | | | | | | | | | 1 |  | BV |  | 25/03/2020 8:26 AM |  |
|  | | | Umm from I suppose, the big difference I noticed is the, umm, its not as intrusive as some other chemo’s so I was very lucky with chemo, I wasn’t sick and all those sorts of things but I, I, my hair did start to fall out so you know, it was coming out in clumps you know. Whereas with the Lutieum, I’ve had none of that. | | | | | | | | | | | | | |  |
|  | | |  | | | | | | | | | | | | | |  |
|  | | | | | | | | | | | | 2 |  | BV |  | 25/03/2020 8:26 AM |  |
|  | | | My appetite’s good, you know, everything is really good about it and umm, its umm, a much quicker process than going through chemo so… | | | | | | | | | | | | | |  |
|  | | |  | | | | | | | | | | | | | |  |
| Formatted Reports\\Coding Summary by File Formatted Report | | | | | | | | | | | Page 18 of 143 | | | | | | |
| 6/10/2022 8:14 AM | | | | | | | | | | | | | | | | | |
|  | | | **Classification** |  | **Aggregate** |  | **Coverage** |  | **Number Of Coding References** |  | | **Reference Number** |  | **Coded By Initials** |  | **Modified On** |  |
|  | | | | | | | | | | | | | | | | | |
|  | | | | | | | | | | | | 3 |  | BV |  | 25/03/2020 8:26 AM |  |
|  | | | Its umm, yeah, its quite umm, I shouldn’t say pleasant but its umm, a hell of a lot better than the standard chemo process that’s for sure | | | | | | | | | | | | | |  |
|  | | |  | | | | | | | | | | | | | |  |
|  | | | | | | | | | | | | 4 |  | BV |  | 25/03/2020 8:26 AM |  |
|  | | | I: Umm, has anything, your participation in the trial affected you physically or psychologically?  P: No. No. Not at all. Umm, umm, I suppose the only thing that you do think about is, it is a radioactive tracer for the first couple of days you know, you cant really, sort of, spend a lot of time with the quality members, cos I have a granddaughter, you know, they say, you know, just don’t stay with them for long periods of time but a part from that no, it’s really affected me no other way. It’s been really good. | | | | | | | | | | | | | |  |
|  | | |  | | | | | | | | | | | | | |  |
|  | | | **Codes\\side effects\side effects - lutetium** | | | | | | | | | | | | | |  |
|  |  |  |  |  | No |  | 0.1400 |  | 4 |  | | | | | | |  |
|  | | |  |  |  |  |  |  |  |  | | | | | | | |
|  | | | | | | | | | | | | 1 |  | BV |  | 14/01/2020 10:36 AM |  |
|  | | | Umm from I suppose, the big difference I noticed is the, umm, its not as intrusive as some other chemo’s so I was very lucky with chemo, I wasn’t sick and all those sorts of things but I, I, my hair did start to fall out so you know, it was coming out in clumps you know. Whereas with the Lutieum, I’ve had none of that. | | | | | | | | | | | | | |  |
|  | | |  | | | | | | | | | | | | | |  |
|  | | | | | | | | | | | | 2 |  | BV |  | 14/01/2020 10:37 AM |  |
|  | | | My appetite’s good, you know, everything is really good about it and umm, its umm, a much quicker process than going through chemo so… | | | | | | | | | | | | | |  |
|  | | |  | | | | | | | | | | | | | |  |
|  | | | | | | | | | | | | 3 |  | BV |  | 14/01/2020 10:37 AM |  |
|  | | | Its umm, yeah, its quite umm, I shouldn’t say pleasant but its umm, a hell of a lot better than the standard chemo process that’s for sure | | | | | | | | | | | | | |  |
|  | | |  | | | | | | | | | | | | | |  |
|  | | | | | | | | | | | | 4 |  | BV |  | 15/01/2020 5:24 AM |  |
|  | | | I: Umm, has anything, your participation in the trial affected you physically or psychologically?  P: No. No. Not at all. Umm, umm, I suppose the only thing that you do think about is, it is a radioactive tracer for the first couple of days you know, you cant really, sort of, spend a lot of time with the quality members, cos I have a granddaughter, you know, they say, you know, just don’t stay with them for long periods of time but a part from that no, it’s really affected me no other way. It’s been really good. | | | | | | | | | | | | | |  |
|  | | |  | | | | | | | | | | | | | |  |
|  | | | **Codes\\staying positive** | | | | | | | | | | | | | |  |
|  |  |  |  |  | No |  | 0.0656 |  | 4 |  | | | | | | |  |
|  | | |  |  |  |  |  |  |  |  | | | | | | | |
|  | | | | | | | | | | | | 1 |  | BV |  | 14/01/2020 10:49 AM |  |
|  | | | Umm, yeah, no, I mean I’m pretty, It’s probably nearly four years since I was diagnosed and I’ve been pretty positive the whole time | | | | | | | | | | | | | |  |
|  | | |  | | | | | | | | | | | | | |  |
|  | | | | | | | | | | | | 2 |  | BV |  | 15/01/2020 4:53 AM |  |
|  | | | I think it’s a case of, like anything, you know, you have your days when you’re a little bit down and you have days where your back up, you know, but you’ve got to stay strong and fight all the way through otherwise, it means giving up. | | | | | | | | | | | | | |  |
|  | | |  | | | | | | | | | | | | | |  |
|  | | | | | | | | | | | | 3 |  | BV |  | 14/01/2020 10:49 AM |  |
|  | | | You’ve got to keep battling so I’m pretty solid in that. | | | | | | | | | | | | | |  |
|  | | |  | | | | | | | | | | | | | |  |
| Formatted Reports\\Coding Summary by File Formatted Report | | | | | | | | | | | Page 19 of 143 | | | | | | |
| 6/10/2022 8:14 AM | | | | | | | | | | | | | | | | | |
|  | | | **Classification** |  | **Aggregate** |  | **Coverage** |  | **Number Of Coding References** |  | | **Reference Number** |  | **Coded By Initials** |  | **Modified On** |  |
|  | | | | | | | | | | | | | | | | | |
|  | | | | | | | | | | | | 4 |  | BV |  | 14/01/2020 10:50 AM |  |
|  | | | I’ve got a pretty good support basis as well, you know, they keep your chin up all the time so… | | | | | | | | | | | | | |  |
|  | | |  | | | | | | | | | | | | | |  |
|  | | | **Codes\\support from family** | | | | | | | | | | | | | |  |
|  |  |  |  |  | No |  | 0.0844 |  | 3 |  | | | | | | |  |
|  | | |  |  |  |  |  |  |  |  | | | | | | | |
|  | | | | | | | | | | | | 1 |  | BV |  | 15/01/2020 5:24 AM |  |
|  | | | I: Umm, has anything, your participation in the trial affected you physically or psychologically?  P: No. No. Not at all. Umm, umm, I suppose the only thing that you do think about is, it is a radioactive tracer for the first couple of days you know, you cant really, sort of, spend a lot of time with the quality members, cos I have a granddaughter, you know, they say, you know, just don’t stay with them for long periods of time but a part from that no, it’s really affected me no other way. It’s been really good. | | | | | | | | | | | | | |  |
|  | | |  | | | | | | | | | | | | | |  |
|  | | | | | | | | | | | | 2 |  | BV |  | 14/01/2020 10:50 AM |  |
|  | | | I’ve got a pretty good support basis as well, you know, they keep your chin up all the time so… | | | | | | | | | | | | | |  |
|  | | |  | | | | | | | | | | | | | |  |
|  | | | | | | | | | | | | 3 |  | BV |  | 14/01/2020 10:52 AM |  |
|  | | | I’ve got a good support base at home and at work here. | | | | | | | | | | | | | |  |
|  | | |  | | | | | | | | | | | | | |  |
|  | | | **Codes\\support from others** | | | | | | | | | | | | | |  |
|  |  |  |  |  | No |  | 0.0616 |  | 3 |  | | | | | | |  |
|  | | |  |  |  |  |  |  |  |  | | | | | | | |
|  | | | | | | | | | | | | 1 |  | BV |  | 14/01/2020 10:50 AM |  |
|  | | | I’ve got a pretty good support basis as well, you know, they keep your chin up all the time so… | | | | | | | | | | | | | |  |
|  | | |  | | | | | | | | | | | | | |  |
|  | | | | | | | | | | | | 2 |  | BV |  | 14/01/2020 10:50 AM |  |
|  | | | No. Not at all. Nah, works been fantastic. They’ve supported me the whole time. All the way through. Umm, yeah, I’ve been with the company for many years and I’ve got a lot of sick time up, but you know, even then, there’s been times where I don’t worry about that, they’ve said don’t worry about it, keep going so that been fantastic. | | | | | | | | | | | | | |  |
|  | | |  | | | | | | | | | | | | | |  |
|  | | | | | | | | | | | | 3 |  | BV |  | 14/01/2020 10:52 AM |  |
|  | | | I’ve got a good support base at home and at work here. | | | | | | | | | | | | | |  |
|  | | |  | | | | | | | | | | | | | |  |
|  | | | **Codes\\support from work** | | | | | | | | | | | | | |  |
|  |  |  |  |  | No |  | 0.0494 |  | 2 |  | | | | | | |  |
|  | | |  |  |  |  |  |  |  |  | | | | | | | |
|  | | | | | | | | | | | | 1 |  | BV |  | 14/01/2020 10:50 AM |  |
|  | | | No. Not at all. Nah, works been fantastic. They’ve supported me the whole time. All the way through. Umm, yeah, I’ve been with the company for many years and I’ve got a lot of sick time up, but you know, even then, there’s been times where I don’t worry about that, they’ve said don’t worry about it, keep going so that been fantastic. | | | | | | | | | | | | | |  |
|  | | |  | | | | | | | | | | | | | |  |
| Formatted Reports\\Coding Summary by File Formatted Report | | | | | | | | | | | Page 20 of 143 | | | | | | |
| 6/10/2022 8:14 AM | | | | | | | | | | | | | | | | | |
|  | | | **Classification** |  | **Aggregate** |  | **Coverage** |  | **Number Of Coding References** |  | | **Reference Number** |  | **Coded By Initials** |  | **Modified On** |  |
|  | | | | | | | | | | | | | | | | | |
|  | | | | | | | | | | | | 2 |  | BV |  | 14/01/2020 10:52 AM |  |
|  | | | I’ve got a good support base at home and at work here. | | | | | | | | | | | | | |  |
|  | | |  | | | | | | | | | | | | | |  |
|  | | | **Codes\\treatments been good** | | | | | | | | | | | | | |  |
|  |  |  |  |  | No |  | 0.0411 |  | 2 |  | | | | | | |  |
|  | | |  |  |  |  |  |  |  |  | | | | | | | |
|  | | | | | | | | | | | | 1 |  | BV |  | 14/01/2020 10:37 AM |  |
|  | | | My appetite’s good, you know, everything is really good about it and umm, its umm, a much quicker process than going through chemo so… | | | | | | | | | | | | | |  |
|  | | |  | | | | | | | | | | | | | |  |
|  | | | | | | | | | | | | 2 |  | BV |  | 14/01/2020 10:38 AM |  |
|  | | | ... are you hungry? Do you want a drink? Something to eat? You know, just, they look after you. Are you comfortable? Tell us this, tell us that. So, they have been fantastic. The whole lot. | | | | | | | | | | | | | |  |
|  | | |  | | | | | | | | | | | | | |  |
|  | | | **Codes\\waiting for doctors** | | | | | | | | | | | | | |  |
|  |  |  |  |  | No |  | 0.1261 |  | 3 |  | | | | | | |  |
|  | | |  |  |  |  |  |  |  |  | | | | | | | |
|  | | | | | | | | | | | | 1 |  | BV |  | 14/01/2020 10:39 AM |  |
|  | | | The only, the only bad experience I’ve had is sometimes you have to wait a bit, but I think that’s just the public health hospital system so yeah. Apart from that. | | | | | | | | | | | | | |  |
|  | | |  | | | | | | | | | | | | | |  |
|  | | | | | | | | | | | | 2 |  | BV |  | 14/01/2020 10:46 AM |  |
|  | | | Yeah. Yeah look well we got to the hospital on Tuesday umm and I have a bone needle every, every six weeks, it’s for bone density… I can remember the name of it. I think it has fifteen letters on it umm and after seeing the doctor about, about 10 o’clock or just after 10 o’clock, it took an hour and a half for him to release the order to go to one part of the section, to go to another section, to go to pharmacy, to then dispense it and get it back to me. So I, I was sitting there probably just after 12 o’clock before I got it, so you know umm yeah … those sorts of things | | | | | | | | | | | | | |  |
|  | | |  | | | | | | | | | | | | | |  |
|  | | | | | | | | | | | | 3 |  | BV |  | 14/01/2020 10:46 AM |  |
|  | | | you know, look I understand there are a lot of people there and it’s very busy but yeah sometimes it’s just a case of saying “oh look, it’s going to be an hour or an hour and a half” and they just sort of make you sit there sort of thing, you know what I mean? | | | | | | | | | | | | | |  |
|  | | |  | | | | | | | | | | | | | |  |
|  | | | | | | | | | | | | | | | | | |
|  | | | | | | | | | | | | | | | | | |
|  | | | | | | | | | | | | | | | | | |
| Formatted Reports\\Coding Summary by File Formatted Report | | | | | | | | | | | Page 21 of 143 | | | | | | |
| 6/10/2022 8:14 AM | | | | | | | | | | | | | | | | | |
|  | | | **Classification** |  | **Aggregate** |  | **Coverage** |  | **Number Of Coding References** |  | | **Reference Number** |  | **Coded By Initials** |  | **Modified On** |  |
|  | **Files\\Transcription 2_006** | | | | | | | | | | | | | | | |  |
|  | | **Code** | | | | | | | | | | | | | | |  |
|  | | | **Codes\\financial impact** | | | | | | | | | | | | | |  |
|  |  |  |  |  | No |  | 0.0842 |  | 1 |  | | | | | | |  |
|  | | |  |  |  |  |  |  |  |  | | | | | | | |
|  | | | | | | | | | | | | 1 |  | BV |  | 14/01/2020 11:05 AM |  |
|  | | | P: Well I mean, it’s a bit annoying I suppose, you’ve got, you know, parking fees aren’t umm, aren’t umm small but I mean it costs about $30, $35 a day to do it so umm yeah but that’s the only thing but again that’s nothing, it’s nothing. It’s umm annoying to think you have to pay that much to, to, go and park for a day but that’s the only thing. A very mild, a very mild annoyance. That’s all.  I: Ok.  P: But other than that, financial, no I can’t see that there’s, umm, compared to the umm, alternative, that’s pain, itself, there’s no financial burden whatsoever. | | | | | | | | | | | | | |  |
|  | | |  | | | | | | | | | | | | | |  |
|  | | | **Codes\\financial impact\hospital parking** | | | | | | | | | | | | | |  |
|  |  |  |  |  | No |  | 0.0584 |  | 1 |  | | | | | | |  |
|  | | |  |  |  |  |  |  |  |  | | | | | | | |
|  | | | | | | | | | | | | 1 |  | BV |  | 15/01/2020 5:08 AM |  |
|  | | | Well I mean, it’s a bit annoying I suppose, you’ve got, you know, parking fees aren’t umm, aren’t umm small but I mean it costs about $30, $35 a day to do it so umm yeah but that’s the only thing but again that’s nothing, it’s nothing. It’s umm annoying to think you have to pay that much to, to, go and park for a day but that’s the only thing. A very mild, a very mild annoyance. That’s all. | | | | | | | | | | | | | |  |
|  | | |  | | | | | | | | | | | | | |  |
|  | | | **Codes\\getting good results** | | | | | | | | | | | | | |  |
|  |  |  |  |  | No |  | 0.0238 |  | 1 |  | | | | | | |  |
|  | | |  |  |  |  |  |  |  |  | | | | | | | |
|  | | | | | | | | | | | | 1 |  | BV |  | 15/01/2020 5:11 AM |  |
|  | | | It is going well. From what I can gather, we are getting good results from the trial so that’s all I can hope for and hopefully it just keeps going for some time. | | | | | | | | | | | | | |  |
|  | | |  | | | | | | | | | | | | | |  |
|  | | | **Codes\\happy about participating** | | | | | | | | | | | | | |  |
|  |  |  |  |  | No |  | 0.0238 |  | 1 |  | | | | | | |  |
|  | | |  |  |  |  |  |  |  |  | | | | | | | |
|  | | | | | | | | | | | | 1 |  | BV |  | 14/01/2020 11:15 AM |  |
|  | | | It is going well. From what I can gather, we are getting good results from the trial so that’s all I can hope for and hopefully it just keeps going for some time. | | | | | | | | | | | | | |  |
|  | | |  | | | | | | | | | | | | | |  |
|  | | | **Codes\\hope** | | | | | | | | | | | | | |  |
|  |  |  |  |  | No |  | 0.0238 |  | 1 |  | | | | | | |  |
|  | | |  |  |  |  |  |  |  |  | | | | | | | |
|  | | | | | | | | | | | | 1 |  | BV |  | 14/01/2020 11:15 AM |  |
|  | | | It is going well. From what I can gather, we are getting good results from the trial so that’s all I can hope for and hopefully it just keeps going for some time. | | | | | | | | | | | | | |  |
|  | | |  | | | | | | | | | | | | | |  |
| Formatted Reports\\Coding Summary by File Formatted Report | | | | | | | | | | | Page 22 of 143 | | | | | | |
| 6/10/2022 8:14 AM | | | | | | | | | | | | | | | | | |
|  | | | **Classification** |  | **Aggregate** |  | **Coverage** |  | **Number Of Coding References** |  | | **Reference Number** |  | **Coded By Initials** |  | **Modified On** |  |
|  | | | **Codes\\hope\hoping treatment lasts** | | | | | | | | | | | | | |  |
|  |  |  |  |  | No |  | 0.0238 |  | 1 |  | | | | | | |  |
|  | | |  |  |  |  |  |  |  |  | | | | | | | |
|  | | | | | | | | | | | | 1 |  | BV |  | 15/01/2020 9:54 AM |  |
|  | | | It is going well. From what I can gather, we are getting good results from the trial so that’s all I can hope for and hopefully it just keeps going for some time. | | | | | | | | | | | | | |  |
|  | | |  | | | | | | | | | | | | | |  |
|  | | | **Codes\\initial motivation for participation** | | | | | | | | | | | | | |  |
|  |  |  |  |  | No |  | 0.0113 |  | 1 |  | | | | | | |  |
|  | | |  |  |  |  |  |  |  |  | | | | | | | |
|  | | | | | | | | | | | | 1 |  | BV |  | 14/01/2020 11:10 AM |  |
|  | | | My motivations ... I think extension of life was the, probably the best one. | | | | | | | | | | | | | |  |
|  | | |  | | | | | | | | | | | | | |  |
|  | | | **Codes\\initial motivation for participation\changes to initial motivation for participation** | | | | | | | | | | | | | |  |
|  |  |  |  |  | No |  | 0.0210 |  | 1 |  | | | | | | |  |
|  | | |  |  |  |  |  |  |  |  | | | | | | | |
|  | | | | | | | | | | | | 1 |  | BV |  | 14/01/2020 11:10 AM |  |
|  | | | Well as I said, things are a lot easier to, umm, to do now so not, not experiencing near the pain I was experiencing so everything’s better. | | | | | | | | | | | | | |  |
|  | | |  | | | | | | | | | | | | | |  |
|  | | | **Codes\\needing more support\no additional support required** | | | | | | | | | | | | | |  |
|  |  |  |  |  | No |  | 0.0122 |  | 1 |  | | | | | | |  |
|  | | |  |  |  |  |  |  |  |  | | | | | | | |
|  | | | | | | | | | | | | 1 |  | BV |  | 14/01/2020 11:09 AM |  |
|  | | | I don’t think so. I can’t think how, how I could have been supported more really. | | | | | | | | | | | | | |  |
|  | | |  | | | | | | | | | | | | | |  |
|  | | | **Codes\\not as intrusive as chemo** | | | | | | | | | | | | | |  |
|  |  |  |  |  | No |  | 0.0122 |  | 1 |  | | | | | | |  |
|  | | |  |  |  |  |  |  |  |  | | | | | | | |
|  | | | | | | | | | | | | 1 |  | BV |  | 14/01/2020 11:01 AM |  |
|  | | | Far easier than some of the other treatments I had umm you know, chemo and things. | | | | | | | | | | | | | |  |
|  | | |  | | | | | | | | | | | | | |  |
|  | | | | | | | | | | | | | | | | | |
| Formatted Reports\\Coding Summary by File Formatted Report | | | | | | | | | | | Page 23 of 143 | | | | | | |
| 6/10/2022 8:14 AM | | | | | | | | | | | | | | | | | |
|  | | | **Classification** |  | **Aggregate** |  | **Coverage** |  | **Number Of Coding References** |  | | **Reference Number** |  | **Coded By Initials** |  | **Modified On** |  |
|  | | | **Codes\\not as intrusive as chemo\nothing to compare Lutetium to** | | | | | | | | | | | | | |  |
|  |  |  |  |  | No |  | 0.0160 |  | 1 |  | | | | | | |  |
|  | | |  |  |  |  |  |  |  |  | | | | | | | |
|  | | | | | | | | | | | | 1 |  | BV |  | 14/01/2020 11:01 AM |  |
|  | | | Well, its fine I suppose. There’s not much to compare it too. It’s umm I guess it’s umm fairly easy at times. | | | | | | | | | | | | | |  |
|  | | |  | | | | | | | | | | | | | |  |
|  | | | **Codes\\quality of life changes** | | | | | | | | | | | | | |  |
|  |  |  |  |  | No |  | 0.0443 |  | 1 |  | | | | | | |  |
|  | | |  |  |  |  |  |  |  |  | | | | | | | |
|  | | | | | | | | | | | | 1 |  | BV |  | 25/03/2020 8:25 AM |  |
|  | | | No, well the only quality of life changes is I can do a lot more I was doing prior to the trial. Umm, the pain was a bit of a barrier to doing certain things, but I found that I’m not physically, as physically, impaired to doing whatever I want to do. Umm, so yeah, so it’s all been good up till now. | | | | | | | | | | | | | |  |
|  | | |  | | | | | | | | | | | | | |  |
|  | | | **Codes\\quality of life changes\quality of life changes - lutetium** | | | | | | | | | | | | | |  |
|  |  |  |  |  | No |  | 0.0446 |  | 1 |  | | | | | | |  |
|  | | |  |  |  |  |  |  |  |  | | | | | | | |
|  | | | | | | | | | | | | 1 |  | BV |  | 14/01/2020 11:03 AM |  |
|  | | | No, well the only quality of life changes is I can do a lot more I was doing prior to the trial. Umm, the pain was a bit of a barrier to doing certain things, but I found that I’m not physically, as physically, impaired to doing whatever I want to do. Umm, so yeah, so it’s all been good up till now. | | | | | | | | | | | | | |  |
|  | | |  | | | | | | | | | | | | | |  |
|  | | | **Codes\\quality of trialists** | | | | | | | | | | | | | |  |
|  |  |  |  |  | No |  | 0.1284 |  | 3 |  | | | | | | |  |
|  | | |  |  |  |  |  |  |  |  | | | | | | | |
|  | | | | | | | | | | | | 1 |  | BV |  | 14/01/2020 11:09 AM |  |
|  | | | Everyone’s been really nice and their all, the people involved in the trial are all very, very easy to talk to and quite happy to umm, to umm, tell you whatever, whatever you want to know umm yeah so no, I think it’s all, as I say, all pretty positive. | | | | | | | | | | | | | |  |
|  | | |  | | | | | | | | | | | | | |  |
|  | | | | | | | | | | | | 2 |  | BV |  | 14/01/2020 11:09 AM |  |
|  | | | No, as I say, everyone’s pretty easy to talk to and pretty easy to umm talk to and to, like if there’s any questions or any queries, they’re all really straight forward and umm, in saying what’s happening and what’s going on so no, it’s all been pretty good. | | | | | | | | | | | | | |  |
|  | | |  | | | | | | | | | | | | | |  |
|  | | | | | | | | | | | | 3 |  | BV |  | 14/01/2020 11:14 AM |  |
|  | | | As I said, we haven’t been left in the dark, with any aspects of the trial umm you know, if we have any queries people are happy to answer the queries we come up with and very straight forward and to let us know what’s happening and what’s been going on so umm no I can’t think of anything that is lacking as far as communication with anyone is concerned. | | | | | | | | | | | | | |  |
|  | | |  | | | | | | | | | | | | | |  |
|  | | | **Codes\\side effects** | | | | | | | | | | | | | |  |
|  |  |  |  |  | No |  | 0.1626 |  | 4 |  | | | | | | |  |
|  | | |  |  |  |  |  |  |  |  | | | | | | | |
|  | | | | | | | | | | | | 1 |  | BV |  | 25/03/2020 8:26 AM |  |
|  | | | I haven’t had any adverse effects that I can really think of too much. Umm, its fairly easy. | | | | | | | | | | | | | |  |
|  | | |  | | | | | | | | | | | | | |  |
| Formatted Reports\\Coding Summary by File Formatted Report | | | | | | | | | | | Page 24 of 143 | | | | | | |
| 6/10/2022 8:14 AM | | | | | | | | | | | | | | | | | |
|  | | | **Classification** |  | **Aggregate** |  | **Coverage** |  | **Number Of Coding References** |  | | **Reference Number** |  | **Coded By Initials** |  | **Modified On** |  |
|  | | | | | | | | | | | | | | | | | |
|  | | | | | | | | | | | | 2 |  | BV |  | 25/03/2020 8:26 AM |  |
|  | | | It’s fine, its, I guess, the day after I guess, is the worst one, where you have to have the scan and sort of lay still for an hour and a half or so or two hours. That’s a bit umm, umm, a bit hard to do I guess but if that’s the worst thing that can happen then there’s no great problem. | | | | | | | | | | | | | |  |
|  | | |  | | | | | | | | | | | | | |  |
|  | | | | | | | | | | | | 3 |  | BV |  | 25/03/2020 8:26 AM |  |
|  | | | Well, not, certainly not psychologically but umm physically, the only thing that’s really, I’ve noticed, is a dry mouth. A very dry mouth. But apart from that it’s been all positive, I guess. I was experiencing quite a bit of pain prior to the trial. It was like someone through a switch when I had the first treatment. The pain almost, not totally, but almost disappeared and so yeah. Quite a, quite startling, the difference, prior to the trial and after the trial.  I: Wow so, reduced pain. That sounds excellent.  P: Yes, it was. Quite extensive, yeah reduction in pain, yes. | | | | | | | | | | | | | |  |
|  | | |  | | | | | | | | | | | | | |  |
|  | | | | | | | | | | | | 4 |  | BV |  | 25/03/2020 8:26 AM |  |
|  | | | Well as I said, things are a lot easier to, umm, to do now so not, not experiencing near the pain I was experiencing so everything’s better. | | | | | | | | | | | | | |  |
|  | | |  | | | | | | | | | | | | | |  |
|  | | | **Codes\\side effects\side effects - lutetium** | | | | | | | | | | | | | |  |
|  |  |  |  |  | No |  | 0.1626 |  | 4 |  | | | | | | |  |
|  | | |  |  |  |  |  |  |  |  | | | | | | | |
|  | | | | | | | | | | | | 1 |  | BV |  | 14/01/2020 11:01 AM |  |
|  | | | I haven’t had any adverse effects that I can really think of too much. Umm, its fairly easy. | | | | | | | | | | | | | |  |
|  | | |  | | | | | | | | | | | | | |  |
|  | | | | | | | | | | | | 2 |  | BV |  | 14/01/2020 11:02 AM |  |
|  | | | It’s fine, its, I guess, the day after I guess, is the worst one, where you have to have the scan and sort of lay still for an hour and a half or so or two hours. That’s a bit umm, umm, a bit hard to do I guess but if that’s the worst thing that can happen then there’s no great problem. | | | | | | | | | | | | | |  |
|  | | |  | | | | | | | | | | | | | |  |
|  | | | | | | | | | | | | 3 |  | BV |  | 15/01/2020 5:24 AM |  |
|  | | | Well, not, certainly not psychologically but umm physically, the only thing that’s really, I’ve noticed, is a dry mouth. A very dry mouth. But apart from that it’s been all positive, I guess. I was experiencing quite a bit of pain prior to the trial. It was like someone through a switch when I had the first treatment. The pain almost, not totally, but almost disappeared and so yeah. Quite a, quite startling, the difference, prior to the trial and after the trial.  I: Wow so, reduced pain. That sounds excellent.  P: Yes, it was. Quite extensive, yeah reduction in pain, yes. | | | | | | | | | | | | | |  |
|  | | |  | | | | | | | | | | | | | |  |
|  | | | | | | | | | | | | 4 |  | BV |  | 15/01/2020 5:24 AM |  |
|  | | | Well as I said, things are a lot easier to, umm, to do now so not, not experiencing near the pain I was experiencing so everything’s better. | | | | | | | | | | | | | |  |
|  | | |  | | | | | | | | | | | | | |  |
|  | | | **Codes\\side effects\side effects - lutetium\reduction in pain** | | | | | | | | | | | | | |  |
|  |  |  |  |  | No |  | 0.0786 |  | 2 |  | | | | | | |  |
|  | | |  |  |  |  |  |  |  |  | | | | | | | |
|  | | | | | | | | | | | | 1 |  | BV |  | 14/01/2020 11:03 AM |  |
|  | | | I was experiencing quite a bit of pain prior to the trial. It was like someone through a switch when I had the first treatment. The pain almost, not totally, but almost disappeared and so yeah. Quite a, quite startling, the difference, prior to the trial and after the trial.  I: Wow so, reduced pain. That sounds excellent.  P: Yes, it was. Quite extensive, yeah reduction in pain, yes. | | | | | | | | | | | | | |  |
|  | | |  | | | | | | | | | | | | | |  |
| Formatted Reports\\Coding Summary by File Formatted Report | | | | | | | | | | | Page 25 of 143 | | | | | | |
| 6/10/2022 8:14 AM | | | | | | | | | | | | | | | | | |
|  | | | **Classification** |  | **Aggregate** |  | **Coverage** |  | **Number Of Coding References** |  | | **Reference Number** |  | **Coded By Initials** |  | **Modified On** |  |
|  | | | | | | | | | | | | | | | | | |
|  | | | | | | | | | | | | 2 |  | BV |  | 14/01/2020 11:10 AM |  |
|  | | | Well as I said, things are a lot easier to, umm, to do now so not, not experiencing near the pain I was experiencing so everything’s better. | | | | | | | | | | | | | |  |
|  | | |  | | | | | | | | | | | | | |  |
|  | | | **Codes\\staying positive** | | | | | | | | | | | | | |  |
|  |  |  |  |  | No |  | 0.0415 |  | 2 |  | | | | | | |  |
|  | | |  |  |  |  |  |  |  |  | | | | | | | |
|  | | | | | | | | | | | | 1 |  | BV |  | 14/01/2020 11:03 AM |  |
|  | | | I don’t think so. As I said, I think it’s more positive than negative. Umm, the trial, I mean, dry mouth is about the only thing that I can think of and if that’s the worst thing that’s going to happen, its nothing to worry about. | | | | | | | | | | | | | |  |
|  | | |  | | | | | | | | | | | | | |  |
|  | | | | | | | | | | | | 2 |  | BV |  | 14/01/2020 11:09 AM |  |
|  | | | I think it’s all, as I say, all pretty positive. | | | | | | | | | | | | | |  |
|  | | |  | | | | | | | | | | | | | |  |
|  | | | **Codes\\struggling with treatment** | | | | | | | | | | | | | |  |
|  |  |  |  |  | No |  | 0.0425 |  | 1 |  | | | | | | |  |
|  | | |  |  |  |  |  |  |  |  | | | | | | | |
|  | | | | | | | | | | | | 1 |  | BV |  | 14/01/2020 11:02 AM |  |
|  | | | It’s fine, its, I guess, the day after I guess, is the worst one, where you have to have the scan and sort of lay still for an hour and a half or so or two hours. That’s a bit umm, umm, a bit hard to do I guess but if that’s the worst thing that can happen then there’s no great problem. | | | | | | | | | | | | | |  |
|  | | |  | | | | | | | | | | | | | |  |
|  | | | **Codes\\struggling with treatment\struggling with post treatment scans** | | | | | | | | | | | | | |  |
|  |  |  |  |  | No |  | 0.0425 |  | 1 |  | | | | | | |  |
|  | | |  |  |  |  |  |  |  |  | | | | | | | |
|  | | | | | | | | | | | | 1 |  | BV |  | 15/01/2020 5:29 AM |  |
|  | | | It’s fine, its, I guess, the day after I guess, is the worst one, where you have to have the scan and sort of lay still for an hour and a half or so or two hours. That’s a bit umm, umm, a bit hard to do I guess but if that’s the worst thing that can happen then there’s no great problem. | | | | | | | | | | | | | |  |
|  | | |  | | | | | | | | | | | | | |  |
|  | | | **Codes\\treatments been good** | | | | | | | | | | | | | |  |
|  |  |  |  |  | No |  | 0.0814 |  | 2 |  | | | | | | |  |
|  | | |  |  |  |  |  |  |  |  | | | | | | | |
|  | | | | | | | | | | | | 1 |  | BV |  | 14/01/2020 11:03 AM |  |
|  | | | I was experiencing quite a bit of pain prior to the trial. It was like someone through a switch when I had the first treatment. The pain almost, not totally, but almost disappeared and so yeah. Quite a, quite startling, the difference, prior to the trial and after the trial.  I: Wow so, reduced pain. That sounds excellent.  P: Yes, it was. Quite extensive, yeah reduction in pain, yes. | | | | | | | | | | | | | |  |
|  | | |  | | | | | | | | | | | | | |  |
|  | | | | | | | | | | | | | | | | | |
| Formatted Reports\\Coding Summary by File Formatted Report | | | | | | | | | | | Page 26 of 143 | | | | | | |
| 6/10/2022 8:14 AM | | | | | | | | | | | | | | | | | |
|  | | | **Classification** |  | **Aggregate** |  | **Coverage** |  | **Number Of Coding References** |  | | **Reference Number** |  | **Coded By Initials** |  | **Modified On** |  |
|  | | | | | | | | | | | | | | | | | |
|  | | | | | | | | | | | | 2 |  | BV |  | 15/01/2020 5:09 AM |  |
|  | | | It is going well. From what I can gather, we are getting good results from the trial so that’s all I can hope for and hopefully it just keeps going for some time. | | | | | | | | | | | | | |  |
|  | | |  | | | | | | | | | | | | | |  |
|  | **Files\\Transcription 2_008** | | | | | | | | | | | | | | | |  |
|  | | **Code** | | | | | | | | | | | | | | |  |
|  | | | **Codes\\coping strategies** | | | | | | | | | | | | | |  |
|  |  |  |  |  | No |  | 0.0400 |  | 2 |  | | | | | | |  |
|  | | |  |  |  |  |  |  |  |  | | | | | | | |
|  | | | | | | | | | | | | 1 |  | BV |  | 11/01/2020 10:14 AM |  |
|  | | | I: Any… what about emotionally, has the treatment trial affected you emotionally from a coping point of view?  P: I try to block it out as much as I can. Get my mind on other things. Things that interest me. Yeah. So…  I: What sort of things interest you?  P: Sport  I: That’s a good one.  P: It’s a very good one. I do tutoring.  I: Okay. What tutoring?  P: English. | | | | | | | | | | | | | |  |
|  |  |  |  |  |  |  |  |  |  |  |  |  |  |  |  |  |  |
|  | | |  | | | | | | | | | | | | | |  |
|  | | | | | | | | | | | | 2 |  | BV |  | 11/01/2020 10:20 AM |  |
|  | | | I try to keep my mind busy, yeah. | | | | | | | | | | | | | |  |
|  | | |  | | | | | | | | | | | | | |  |
|  | | | **Codes\\financial impact\no affect to finances** | | | | | | | | | | | | | |  |
|  |  |  |  |  | No |  | 0.0163 |  | 1 |  | | | | | | |  |
|  | | |  |  |  |  |  |  |  |  | | | | | | | |
|  | | | | | | | | | | | | 1 |  | BV |  | 11/01/2020 10:21 AM |  |
|  | | | What about, has anything affected you on the trial, such as any finances, or any practical aspects, of your life been affected by the trial?  P: Not at this stage. | | | | | | | | | | | | | |  |
|  | | |  | | | | | | | | | | | | | |  |
|  | | | **Codes\\happy about participating** | | | | | | | | | | | | | |  |
|  |  |  |  |  | No |  | 0.0022 |  | 1 |  | | | | | | |  |
|  | | |  |  |  |  |  |  |  |  | | | | | | | |
|  | | | | | | | | | | | | 1 |  | BV |  | 11/01/2020 10:24 AM |  |
|  | | | Oh yes, yes, yes, sure. | | | | | | | | | | | | | |  |
|  | | |  | | | | | | | | | | | | | |  |
|  | | | | | | | | | | | | | | | | | |
| Formatted Reports\\Coding Summary by File Formatted Report | | | | | | | | | | | Page 27 of 143 | | | | | | |
| 6/10/2022 8:14 AM | | | | | | | | | | | | | | | | | |
|  | | | **Classification** |  | **Aggregate** |  | **Coverage** |  | **Number Of Coding References** |  | | **Reference Number** |  | **Coded By Initials** |  | **Modified On** |  |
|  | | | **Codes\\initial motivation for participation** | | | | | | | | | | | | | |  |
|  |  |  |  |  | No |  | 0.0132 |  | 1 |  | | | | | | |  |
|  | | |  |  |  |  |  |  |  |  | | | | | | | |
|  | | | | | | | | | | | | 1 |  | BV |  | 11/01/2020 10:24 AM |  |
|  | | | I: Thinking back, what were your initial motivations or reasons for participating in the trial?  P: Oh, for my health to be restored. | | | | | | | | | | | | | |  |
|  | | |  | | | | | | | | | | | | | |  |
|  | | | **Codes\\initial motivation for participation\changes to initial motivation for participation** | | | | | | | | | | | | | |  |
|  |  |  |  |  | No |  | 0.0122 |  | 1 |  | | | | | | |  |
|  | | |  |  |  |  |  |  |  |  | | | | | | | |
|  | | | | | | | | | | | | 1 |  | BV |  | 11/01/2020 10:24 AM |  |
|  | | | I: And do you feel like that motivation has changed, still being part of the trial for the same reason?  P: Yeah. Yes, yes. | | | | | | | | | | | | | |  |
|  | | |  | | | | | | | | | | | | | |  |
|  | | | **Codes\\initial motivation for participation\doctors recommendation** | | | | | | | | | | | | | |  |
|  |  |  |  |  | No |  | 0.0142 |  | 1 |  | | | | | | |  |
|  | | |  |  |  |  |  |  |  |  | | | | | | | |
|  | | | | | | | | | | | | 1 |  | BV |  | 11/01/2020 10:08 AM |  |
|  | | | Well, I guess everything is doing me good. I can’t deny that. I wouldn’t be, I wouldn’t have been prescribed this if it wasn’t doing me good. | | | | | | | | | | | | | |  |
|  | | |  | | | | | | | | | | | | | |  |
|  | | | **Codes\\needing more support** | | | | | | | | | | | | | |  |
|  |  |  |  |  | No |  | 0.0539 |  | 2 |  | | | | | | |  |
|  | | |  |  |  |  |  |  |  |  | | | | | | | |
|  | | | | | | | | | | | | 1 |  | BV |  | 11/01/2020 10:22 AM |  |
|  | | | That’s a hard one. You see, next week I’ve got a blood test to do on the Monday, I’ve got a ???? readiness for the chemo on the Wednesday. I just hope I’m going to be strong enough to walk out, to walk out of here, hop on the bus, then hop on the tram and get off at the Peter Mac. Rather than have to call on someone again, to help me with transport. I don’t like doing it to the same person over and over. | | | | | | | | | | | | | |  |
|  | | |  | | | | | | | | | | | | | |  |
|  | | | | | | | | | | | | 2 |  | BV |  | 11/01/2020 10:23 AM |  |
|  | | | I’m going to get some home help, someone’s coming in to clean up and do some messages. I’m having a physio in on Thursday. Yes, yeah. | | | | | | | | | | | | | |  |
|  | | |  | | | | | | | | | | | | | |  |
|  | | | **Codes\\no family support** | | | | | | | | | | | | | |  |
|  |  |  |  |  | No |  | 0.0025 |  | 1 |  | | | | | | |  |
|  | | |  |  |  |  |  |  |  |  | | | | | | | |
|  | | | | | | | | | | | | 1 |  | BV |  | 11/01/2020 10:20 AM |  |
|  | | | No, I don’t have family. | | | | | | | | | | | | | |  |
|  | | |  | | | | | | | | | | | | | |  |
| Formatted Reports\\Coding Summary by File Formatted Report | | | | | | | | | | | Page 28 of 143 | | | | | | |
| 6/10/2022 8:14 AM | | | | | | | | | | | | | | | | | |
|  | | | **Classification** |  | **Aggregate** |  | **Coverage** |  | **Number Of Coding References** |  | | **Reference Number** |  | **Coded By Initials** |  | **Modified On** |  |
|  | | | **Codes\\quality of life changes** | | | | | | | | | | | | | |  |
|  |  |  |  |  | No |  | 0.0344 |  | 2 |  | | | | | | |  |
|  | | |  |  |  |  |  |  |  |  | | | | | | | |
|  | | | | | | | | | | | | 1 |  | BV |  | 25/03/2020 8:25 AM |  |
|  | | | It’s, it’s been alright, but I’ve been feeling weak for some weeks on and off, maybe after breakfast some mornings, I have to sit down. I’ve really done nothing I’ve really got to sit down and relax and wait until I feel better and up and about. | | | | | | | | | | | | | |  |
|  | | |  | | | | | | | | | | | | | |  |
|  | | | | | | | | | | | | 2 |  | BV |  | 25/03/2020 8:25 AM |  |
|  | | | I feel too weak at the moment, to even drive. I’m not prepared to drive the car. So, that is on hold. | | | | | | | | | | | | | |  |
|  | | |  | | | | | | | | | | | | | |  |
|  | | | **Codes\\quality of life changes\quality of life changes - cabazitaxel** | | | | | | | | | | | | | |  |
|  |  |  |  |  | No |  | 0.0344 |  | 2 |  | | | | | | |  |
|  | | |  |  |  |  |  |  |  |  | | | | | | | |
|  | | | | | | | | | | | | 1 |  | BV |  | 15/01/2020 5:26 AM |  |
|  | | | It’s, it’s been alright, but I’ve been feeling weak for some weeks on and off, maybe after breakfast some mornings, I have to sit down. I’ve really done nothing I’ve really got to sit down and relax and wait until I feel better and up and about. | | | | | | | | | | | | | |  |
|  | | |  | | | | | | | | | | | | | |  |
|  | | | | | | | | | | | | 2 |  | BV |  | 15/01/2020 5:38 AM |  |
|  | | | I feel too weak at the moment, to even drive. I’m not prepared to drive the car. So, that is on hold. | | | | | | | | | | | | | |  |
|  | | |  | | | | | | | | | | | | | |  |
|  | | | **Codes\\quality of trialists** | | | | | | | | | | | | | |  |
|  |  |  |  |  | No |  | 0.0123 |  | 1 |  | | | | | | |  |
|  | | |  |  |  |  |  |  |  |  | | | | | | | |
|  | | | | | | | | | | | | 1 |  | BV |  | 11/01/2020 10:23 AM |  |
|  | | | Oh yes, yes. Very helpful. They’re wonderful people, they really are. They’re so caring, so obliging, so understanding, yes. | | | | | | | | | | | | | |  |
|  | | |  | | | | | | | | | | | | | |  |
|  | | | **Codes\\side effects** | | | | | | | | | | | | | |  |
|  |  |  |  |  | No |  | 0.2198 |  | 5 |  | | | | | | |  |
|  | | |  |  |  |  |  |  |  |  | | | | | | | |
|  | | | | | | | | | | | | 1 |  | BV |  | 25/03/2020 8:26 AM |  |
|  | | | It’s, it’s been alright, but I’ve been feeling weak for some weeks on and off, maybe after breakfast some mornings, I have to sit down. I’ve really done nothing I’ve really got to sit down and relax and wait until I feel better and up and about. | | | | | | | | | | | | | |  |
|  | | |  | | | | | | | | | | | | | |  |
|  | | | | | | | | | | | | 2 |  | BV |  | 25/03/2020 8:26 AM |  |
|  | | | Things have slowed since last week, when I had the fall and then I went to the podiatrist and I was so weak that they got the ambulance, so that changed. I don’t know what the cause of that was, was it the blood pressure tablets… See, with the blood pressure tablets, they’ve changed, the dose has been reduced. I don’t know if that is significant, after what happened last week, but the whole business of blood pressure tablets has been chopping and changing for a good deal of time. | | | | | | | | | | | | | |  |
|  | | |  | | | | | | | | | | | | | |  |
|  | | | | | | | | | | | | | | | | | |
| Formatted Reports\\Coding Summary by File Formatted Report | | | | | | | | | | | Page 29 of 143 | | | | | | |
| 6/10/2022 8:14 AM | | | | | | | | | | | | | | | | | |
|  | | | **Classification** |  | **Aggregate** |  | **Coverage** |  | **Number Of Coding References** |  | | **Reference Number** |  | **Coded By Initials** |  | **Modified On** |  |
|  | | | | | | | | | | | | | | | | | |
|  | | | | | | | | | | | | 3 |  | BV |  | 25/03/2020 8:26 AM |  |
|  | | | Well, if you go by the PSA as to what happened, since I’ve started this second time, last time I started I was on chemo until I developed swollen legs and they took me off the chemo about December. Now I’ve started again, and I’ve had 5 sessions, but the PSA has dropped alarmingly. I can tell you off the top of my head what’s happened. First time, the PSA was 2000 plus, next time was 1000 plus, the next time was 440 plus, the next time was 111. It’s come down. And that’s made me feel a lot better.  I: That’s good.  P: It’s very good, but I’m still experiencing trouble with going to the toilet of a night time so many times and having to wear pads.  I: Oh yeah, okay, I can understand.  P: That is an ongoing problem. I did have a procedure at the Royal Melbourne Hospital on the 31st of July and then I wore a catheter for a week, but since that catheter has come out, I’m still having trouble with urinating and still having to wear pads a number of times a day. | | | | | | | | | | | | | |  |
|  |  |  |  |  |  |  |  |  |  |  |  |  |  |  |  |  |  |
|  | | |  | | | | | | | | | | | | | |  |
|  | | | | | | | | | | | | 4 |  | BV |  | 25/03/2020 8:26 AM |  |
|  | | | I feel too weak at the moment, to even drive. I’m not prepared to drive the car. So, that is on hold. | | | | | | | | | | | | | |  |
|  | | |  | | | | | | | | | | | | | |  |
|  | | | | | | | | | | | | 5 |  | BV |  | 25/03/2020 8:26 AM |  |
|  | | | That’s a hard one. You see, next week I’ve got a blood test to do on the Monday, I’ve got a ???? readiness for the chemo on the Wednesday. I just hope I’m going to be strong enough to walk out, to walk out of here, hop on the bus, then hop on the tram and get off at the Peter Mac. Rather than have to call on someone again, to help me with transport. I don’t like doing it to the same person over and over. | | | | | | | | | | | | | |  |
|  | | |  | | | | | | | | | | | | | |  |
|  | | | **Codes\\side effects\side effects - cabazitaxel** | | | | | | | | | | | | | |  |
|  |  |  |  |  | No |  | 0.2198 |  | 5 |  | | | | | | |  |
|  | | |  |  |  |  |  |  |  |  | | | | | | | |
|  | | | | | | | | | | | | 1 |  | BV |  | 15/01/2020 5:26 AM |  |
|  | | | It’s, it’s been alright, but I’ve been feeling weak for some weeks on and off, maybe after breakfast some mornings, I have to sit down. I’ve really done nothing I’ve really got to sit down and relax and wait until I feel better and up and about. | | | | | | | | | | | | | |  |
|  | | |  | | | | | | | | | | | | | |  |
|  | | | | | | | | | | | | 2 |  | BV |  | 15/01/2020 5:27 AM |  |
|  | | | Things have slowed since last week, when I had the fall and then I went to the podiatrist and I was so weak that they got the ambulance, so that changed. I don’t know what the cause of that was, was it the blood pressure tablets… See, with the blood pressure tablets, they’ve changed, the dose has been reduced. I don’t know if that is significant, after what happened last week, but the whole business of blood pressure tablets has been chopping and changing for a good deal of time. | | | | | | | | | | | | | |  |
|  | | |  | | | | | | | | | | | | | |  |
|  | | | | | | | | | | | | 3 |  | BV |  | 15/01/2020 5:27 AM |  |
|  | | | Well, if you go by the PSA as to what happened, since I’ve started this second time, last time I started I was on chemo until I developed swollen legs and they took me off the chemo about December. Now I’ve started again, and I’ve had 5 sessions, but the PSA has dropped alarmingly. I can tell you off the top of my head what’s happened. First time, the PSA was 2000 plus, next time was 1000 plus, the next time was 440 plus, the next time was 111. It’s come down. And that’s made me feel a lot better.  I: That’s good.  P: It’s very good, but I’m still experiencing trouble with going to the toilet of a night time so many times and having to wear pads.  I: Oh yeah, okay, I can understand.  P: That is an ongoing problem. I did have a procedure at the Royal Melbourne Hospital on the 31st of July and then I wore a catheter for a week, but since that catheter has come out, I’m still having trouble with urinating and still having to wear pads a number of times a day. | | | | | | | | | | | | | |  |
|  |  |  |  |  |  |  |  |  |  |  |  |  |  |  |  |  |  |
|  | | |  | | | | | | | | | | | | | |  |
|  | | | | | | | | | | | | 4 |  | BV |  | 15/01/2020 5:28 AM |  |
|  | | | I feel too weak at the moment, to even drive. I’m not prepared to drive the car. So, that is on hold. | | | | | | | | | | | | | |  |
|  | | |  | | | | | | | | | | | | | |  |
|  | | | | | | | | | | | | 5 |  | BV |  | 15/01/2020 5:28 AM |  |
|  | | | That’s a hard one. You see, next week I’ve got a blood test to do on the Monday, I’ve got a ???? readiness for the chemo on the Wednesday. I just hope I’m going to be strong enough to walk out, to walk out of here, hop on the bus, then hop on the tram and get off at the Peter Mac. Rather than have to call on someone again, to help me with transport. I don’t like doing it to the same person over and over. | | | | | | | | | | | | | |  |
|  | | |  | | | | | | | | | | | | | |  |
| Formatted Reports\\Coding Summary by File Formatted Report | | | | | | | | | | | Page 30 of 143 | | | | | | |
| 6/10/2022 8:14 AM | | | | | | | | | | | | | | | | | |
|  | | | **Classification** |  | **Aggregate** |  | **Coverage** |  | **Number Of Coding References** |  | | **Reference Number** |  | **Coded By Initials** |  | **Modified On** |  |
|  | | | **Codes\\side effects\side effects - lutetium** | | | | | | | | | | | | | |  |
|  |  |  |  |  | No |  | 0.0245 |  | 1 |  | | | | | | |  |
|  | | |  |  |  |  |  |  |  |  | | | | | | | |
|  | | | | | | | | | | | | 1 |  | BV |  | 11/01/2020 10:07 AM |  |
|  | | | It’s, it’s been alright, but I’ve been feeling weak for some weeks on and off, maybe after breakfast some mornings, I have to sit down. I’ve really done nothing I’ve really got to sit down and relax and wait until I feel better and up and about. | | | | | | | | | | | | | |  |
|  | | |  | | | | | | | | | | | | | |  |
|  | | | **Codes\\struggling with treatment\struggling with additional health issues** | | | | | | | | | | | | | |  |
|  |  |  |  |  | No |  | 0.1796 |  | 4 |  | | | | | | |  |
|  | | |  |  |  |  |  |  |  |  | | | | | | | |
|  | | | | | | | | | | | | 1 |  | BV |  | 15/01/2020 5:26 AM |  |
|  | | | Things have slowed since last week, when I had the fall and then I went to the podiatrist and I was so weak that they got the ambulance, so that changed. I don’t know what the cause of that was, was it the blood pressure tablets… See, with the blood pressure tablets, they’ve changed, the dose has been reduced. I don’t know if that is significant, after what happened last week, but the whole business of blood pressure tablets has been chopping and changing for a good deal of time. | | | | | | | | | | | | | |  |
|  | | |  | | | | | | | | | | | | | |  |
|  | | | | | | | | | | | | 2 |  | BV |  | 11/01/2020 10:09 AM |  |
|  | | | Well then, then at the Royal Melbourne Hospital, they were looking at things like heart trouble and I think I’m going to have my heart checked and they were asking me about family history, which I told them. My mother died of heart trouble; she was 55. My father died in 1943, which is along time ago, I don’t know exactly how old he was, but I don’t think he could have been much older than 50. At that time, I was, I think I was 7, and my big brother was 9, so that will give you some idea. But I’ve never had any heart trouble at all. | | | | | | | | | | | | | |  |
|  | | |  | | | | | | | | | | | | | |  |
|  | | | | | | | | | | | | 3 |  | BV |  | 11/01/2020 10:10 AM |  |
|  | | | I: So, when are you going to have your heart checked?  P: Well, I’m waiting on a letter.  I: Oh, okay.  P: I did have it checked last year after I had a fall, but they, I, I went to a place in Niddrie called One Heart, One Heart and they put the monitor on me and it was found that there was nothing, nothing wrong. | | | | | | | | | | | | | |  |
|  | | |  | | | | | | | | | | | | | |  |
|  | | | | | | | | | | | | 4 |  | BV |  | 15/01/2020 5:27 AM |  |
|  | | | I: That’s good.  P: It’s very good, but I’m still experiencing trouble with going to the toilet of a night time so many times and having to wear pads.  I: Oh yeah, okay, I can understand.  P: That is an ongoing problem. I did have a procedure at the Royal Melbourne Hospital on the 31st of July and then I wore a catheter for a week, but since that catheter has come out, I’m still having trouble with urinating and still having to wear pads a number of times a day. | | | | | | | | | | | | | |  |
|  | | |  | | | | | | | | | | | | | |  |
|  | | | **Codes\\struggling with treatment\symptom fluctuations** | | | | | | | | | | | | | |  |
|  |  |  |  |  | No |  | 0.0725 |  | 2 |  | | | | | | |  |
|  | | |  |  |  |  |  |  |  |  | | | | | | | |
|  | | | | | | | | | | | | 1 |  | BV |  | 15/01/2020 5:27 AM |  |
|  | | | It’s, it’s been alright, but I’ve been feeling weak for some weeks on and off, maybe after breakfast some mornings, I have to sit down. I’ve really done nothing I’ve really got to sit down and relax and wait until I feel better and up and about. | | | | | | | | | | | | | |  |
|  | | |  | | | | | | | | | | | | | |  |
|  | | | | | | | | | | | | 2 |  | BV |  | 15/01/2020 5:27 AM |  |
|  | | | Things have slowed since last week, when I had the fall and then I went to the podiatrist and I was so weak that they got the ambulance, so that changed. I don’t know what the cause of that was, was it the blood pressure tablets… See, with the blood pressure tablets, they’ve changed, the dose has been reduced. I don’t know if that is significant, after what happened last week, but the whole business of blood pressure tablets has been chopping and changing for a good deal of time. | | | | | | | | | | | | | |  |
|  | | |  | | | | | | | | | | | | | |  |
|  | | | | | | | | | | | | | | | | | |
| Formatted Reports\\Coding Summary by File Formatted Report | | | | | | | | | | | Page 31 of 143 | | | | | | |
| 6/10/2022 8:14 AM | | | | | | | | | | | | | | | | | |
|  | | | **Classification** |  | **Aggregate** |  | **Coverage** |  | **Number Of Coding References** |  | | **Reference Number** |  | **Coded By Initials** |  | **Modified On** |  |
|  | | | **Codes\\support from others** | | | | | | | | | | | | | |  |
|  |  |  |  |  | No |  | 0.0016 |  | 1 |  | | | | | | |  |
|  | | |  |  |  |  |  |  |  |  | | | | | | | |
|  | | | | | | | | | | | | 1 |  | BV |  | 11/01/2020 10:21 AM |  |
|  | | | I’ve got friends. | | | | | | | | | | | | | |  |
|  | | |  | | | | | | | | | | | | | |  |
|  | | | **Codes\\travelling to treatment** | | | | | | | | | | | | | |  |
|  |  |  |  |  | No |  | 0.0431 |  | 1 |  | | | | | | |  |
|  | | |  |  |  |  |  |  |  |  | | | | | | | |
|  | | | | | | | | | | | | 1 |  | BV |  | 11/01/2020 10:22 AM |  |
|  | | | For the cancer, I take public transport. I take a bus, that’s not far from my door. I go to Sydney Road in Coburg, and hop on the tram, and go down Sydney Road to the Peter Mac. All up, it’s around about 40 minutes, from the time I leave here.  I: And that doesn’t bother you?  P: That doesn’t bother me, no.  I: Does it cost for you to travel?  P: No, because I’m a pensioner and I’ll use a Myki card, that’s, that’s a great saving. | | | | | | | | | | | | | |  |
|  | | |  | | | | | | | | | | | | | |  |
|  | | | **Codes\\travelling to treatment\travelling to treatment challenging** | | | | | | | | | | | | | |  |
|  |  |  |  |  | No |  | 0.0405 |  | 1 |  | | | | | | |  |
|  | | |  |  |  |  |  |  |  |  | | | | | | | |
|  | | | | | | | | | | | | 1 |  | BV |  | 15/01/2020 5:28 AM |  |
|  | | | That’s a hard one. You see, next week I’ve got a blood test to do on the Monday, I’ve got a ???? readiness for the chemo on the Wednesday. I just hope I’m going to be strong enough to walk out, to walk out of here, hop on the bus, then hop on the tram and get off at the Peter Mac. Rather than have to call on someone again, to help me with transport. I don’t like doing it to the same person over and over. | | | | | | | | | | | | | |  |
|  | | |  | | | | | | | | | | | | | |  |
|  | | | **Codes\\treatments been good** | | | | | | | | | | | | | |  |
|  |  |  |  |  | No |  | 0.0645 |  | 2 |  | | | | | | |  |
|  | | |  |  |  |  |  |  |  |  | | | | | | | |
|  | | | | | | | | | | | | 1 |  | BV |  | 11/01/2020 10:08 AM |  |
|  | | | Well, I guess everything is doing me good. I can’t deny that. I wouldn’t be, I wouldn’t have been prescribed this if it wasn’t doing me good. | | | | | | | | | | | | | |  |
|  | | |  | | | | | | | | | | | | | |  |
|  | | | | | | | | | | | | 2 |  | BV |  | 11/01/2020 10:12 AM |  |
|  | | | Well, if you go by the PSA as to what happened, since I’ve started this second time, last time I started I was on chemo until I developed swollen legs and they took me off the chemo about December. Now I’ve started again, and I’ve had 5 sessions, but the PSA has dropped alarmingly. I can tell you off the top of my head what’s happened. First time, the PSA was 2000 plus, next time was 1000 plus, the next time was 440 plus, the next time was 111. It’s come down. And that’s made me feel a lot better. | | | | | | | | | | | | | |  |
|  | | |  | | | | | | | | | | | | | |  |
|  | | | | | | | | | | | | | | | | | |
|  | | | | | | | | | | | | | | | | | |
| Formatted Reports\\Coding Summary by File Formatted Report | | | | | | | | | | | Page 32 of 143 | | | | | | |
| 6/10/2022 8:14 AM | | | | | | | | | | | | | | | | | |
|  | | | **Classification** |  | **Aggregate** |  | **Coverage** |  | **Number Of Coding References** |  | | **Reference Number** |  | **Coded By Initials** |  | **Modified On** |  |
|  | | | **Codes\\waiting for doctors** | | | | | | | | | | | | | |  |
|  |  |  |  |  | No |  | 0.0065 |  | 1 |  | | | | | | |  |
|  | | |  |  |  |  |  |  |  |  | | | | | | | |
|  | | | | | | | | | | | | 1 |  | BV |  | 11/01/2020 10:23 AM |  |
|  | | | Well, that, that’s part of hospital treatment, isn’t it? You wait. | | | | | | | | | | | | | |  |
|  | | |  | | | | | | | | | | | | | |  |
|  | **Files\\Transcription 2_009** | | | | | | | | | | | | | | | |  |
|  | | **Code** | | | | | | | | | | | | | | |  |
|  | | | **Codes\\coping strategies** | | | | | | | | | | | | | |  |
|  |  |  |  |  | No |  | 0.0130 |  | 1 |  | | | | | | |  |
|  | | |  |  |  |  |  |  |  |  | | | | | | | |
|  | | | | | | | | | | | | 1 |  | BV |  | 11/01/2020 10:14 AM |  |
|  | | | Oh no. I always try to keep a, I try to keep a, I don’t normally like to make my life too complicated. So, I try to keep my life uncomplicated. No, apart from that no, none. I think I’ve been fine. | | | | | | | | | | | | | |  |
|  | | |  | | | | | | | | | | | | | |  |
|  | | | **Codes\\doctor happy with progress** | | | | | | | | | | | | | |  |
|  |  |  |  |  | No |  | 0.0255 |  | 2 |  | | | | | | |  |
|  | | |  |  |  |  |  |  |  |  | | | | | | | |
|  | | | | | | | | | | | | 1 |  | BV |  | 10/01/2020 10:46 AM |  |
|  | | | Yes, I do believe it is, yeah… He was in, they all watched me get it last, well, 2 weeks ago. I had to, they took my scans and then I had to wait around you know... They all talked to me, sort of thing. Actually, he was very, very happy with my progress, yeah. | | | | | | | | | | | | | |  |
|  | | |  | | | | | | | | | | | | | |  |
|  | | | | | | | | | | | | 2 |  | BV |  | 10/01/2020 10:59 AM |  |
|  | | | So, you’ve got, he was, he was really, that’s what I said to him, I said well mate if you’re happy, so am I, so am I, yeah. | | | | | | | | | | | | | |  |
|  | | |  | | | | | | | | | | | | | |  |
|  | | | **Codes\\feeling lucky** | | | | | | | | | | | | | |  |
|  |  |  |  |  | No |  | 0.0223 |  | 3 |  | | | | | | |  |
|  | | |  |  |  |  |  |  |  |  | | | | | | | |
|  | | | | | | | | | | | | 1 |  | BV |  | 10/01/2020 10:55 AM |  |
|  | | | So, I’m actually hoping, I mean when I went, when I applied for the trial, you either got gold standard chemo, or Lutetium, and I was hoping for the Lutetium and I was lucky enough to get on it, so… I actually thought I was pretty lucky. | | | | | | | | | | | | | |  |
|  | | |  | | | | | | | | | | | | | |  |
|  | | | | | | | | | | | | 2 |  | BV |  | 15/01/2020 5:03 AM |  |
|  | | | So, I’m actually hoping, I mean when I went, when I applied for the trial, you either got gold standard chemo, or Lutetium, and I was hoping for the Lutetium and I was lucky enough to get on it, so… I actually thought I was pretty lucky. | | | | | | | | | | | | | |  |
|  | | |  | | | | | | | | | | | | | |  |
| Formatted Reports\\Coding Summary by File Formatted Report | | | | | | | | | | | Page 33 of 143 | | | | | | |
| 6/10/2022 8:14 AM | | | | | | | | | | | | | | | | | |
|  | | | **Classification** |  | **Aggregate** |  | **Coverage** |  | **Number Of Coding References** |  | | **Reference Number** |  | **Coded By Initials** |  | **Modified On** |  |
|  | | | | | | | | | | | | | | | | | |
|  | | | | | | | | | | | | 3 |  | BV |  | 10/01/2020 10:56 AM |  |
|  | | | I still think I’m lucky, but I don’t know, you don’t know what’s down the track… we don’t know. | | | | | | | | | | | | | |  |
|  | | |  | | | | | | | | | | | | | |  |
|  | | | **Codes\\financial impact\no affect to finances** | | | | | | | | | | | | | |  |
|  |  |  |  |  | No |  | 0.0181 |  | 1 |  | | | | | | |  |
|  | | |  |  |  |  |  |  |  |  | | | | | | | |
|  | | | | | | | | | | | | 1 |  | BV |  | 10/01/2020 10:44 AM |  |
|  | | | Oh no. I always try to keep a, I try to keep a, I don’t normally like to make my life too complicated. So, I try to keep my life uncomplicated. No, apart from that no, none. I think I’ve been fine.  I: Okay. Any financial or practical impact so far?  P1: No, not really no. | | | | | | | | | | | | | |  |
|  | | |  | | | | | | | | | | | | | |  |
|  | | | **Codes\\happy about participating** | | | | | | | | | | | | | |  |
|  |  |  |  |  | No |  | 0.0319 |  | 2 |  | | | | | | |  |
|  | | |  |  |  |  |  |  |  |  | | | | | | | |
|  | | | | | | | | | | | | 1 |  | BV |  | 10/01/2020 10:52 AM |  |
|  | | | I applied and I went and did all the scans and everything, and I was suitable. I was a suitable applicant and I got offered the trial, which I was very happy.  I: That’s good.  P1: I’m still happy, I’m still happy being on the trial, yeah. | | | | | | | | | | | | | |  |
|  | | |  | | | | | | | | | | | | | |  |
|  | | | | | | | | | | | | 2 |  | BV |  | 10/01/2020 10:55 AM |  |
|  | | | So, I’m actually hoping, I mean when I went, when I applied for the trial, you either got gold standard chemo, or Lutetium, and I was hoping for the Lutetium and I was lucky enough to get on it, so… I actually thought I was pretty lucky. | | | | | | | | | | | | | |  |
|  | | |  | | | | | | | | | | | | | |  |
|  | | | **Codes\\hope** | | | | | | | | | | | | | |  |
|  |  |  |  |  | No |  | 0.0157 |  | 1 |  | | | | | | |  |
|  | | |  |  |  |  |  |  |  |  | | | | | | | |
|  | | | | | | | | | | | | 1 |  | BV |  | 15/01/2020 5:03 AM |  |
|  | | | So, I’m actually hoping, I mean when I went, when I applied for the trial, you either got gold standard chemo, or Lutetium, and I was hoping for the Lutetium and I was lucky enough to get on it, so… I actually thought I was pretty lucky. | | | | | | | | | | | | | |  |
|  | | |  | | | | | | | | | | | | | |  |
|  | | | **Codes\\initial motivation for participation** | | | | | | | | | | | | | |  |
|  |  |  |  |  | No |  | 0.1998 |  | 4 |  | | | | | | |  |
|  | | |  |  |  |  |  |  |  |  | | | | | | | |
|  | | | | | | | | | | | | 1 |  | BV |  | 10/01/2020 10:51 AM |  |
|  | | | I was always, it was actually our local doctor, actually we went there for our flu shots you know, and then he was talking about how he knew, or was a friend, or he knew somebody, because you can actually pay for this drug if you want to, and I think it was about, I think if you had the 6, the 6 things, the 6 doses, it was about $60,000, or something like that… and he was telling me that, the bloke that he knew went to, you couldn’t get it done, you couldn’t pay for it in Melbourne, and you could get it in Perth, a private hospital in Sydney and also somewhere in Brisbane. Obviously where maybe where you work, I don’t know… | | | | | | | | | | | | | |  |
|  | | |  | | | | | | | | | | | | | |  |
|  | | | | | | | | | | | | | | | | | |
| Formatted Reports\\Coding Summary by File Formatted Report | | | | | | | | | | | Page 34 of 143 | | | | | | |
| 6/10/2022 8:14 AM | | | | | | | | | | | | | | | | | |
|  | | | **Classification** |  | **Aggregate** |  | **Coverage** |  | **Number Of Coding References** |  | | **Reference Number** |  | **Coded By Initials** |  | **Modified On** |  |
|  | | | | | | | | | | | | | | | | | |
|  | | | | | | | | | | | | 2 |  | BV |  | 10/01/2020 10:52 AM |  |
|  | | | So, that was the 3 places, and we heard that in one of the hospitals, I’m not too sure which hospital in Melbourne, was going to also start doing it later on in the year. So we, or (name withheld) did then, she was looking up and she was ringing head office in Perth and we were looking at, to, I could of actually done it in Sydney, but the whole thing is, because my daughter lives pretty close to the one in Sydney, but the whole point is, because you’re radioactive for a couple of days, she’s got a couple of young boys and I really didn’t want to go anywhere them. So, I then applied for the trial, for this, trial came along you know, so we, I think it might have been our local doctor that said, because I used to go to Peter Mac, I had my operation, you know originally in Peter Mac, and I went to Peter Mac for quite a while. It’s only the last 3 years I’ve been to Andrew Love and that was again because I started doing chemo, I had to every, it was quite, I don’t know, every 3 weeks, and the traffic, that was in the old hospital, and the traffic was murder. So, we transferred to Andrew Love and so, that’s the reason why I ended up in Andrew Love in Geelong, and the trial, it wasn’t in Andrew Love so, the trial was down at Peter Mac so, I applied and I went and did all the scans and everything, and I was suitable. I was a suitable applicant and I got offered the trial, which I was very happy.  I: That’s good.  P1: I’m still happy, I’m still happy being on the trial, yeah. | | | | | | | | | | | | | |  |
|  |  |  |  |  |  |  |  |  |  |  |  |  |  |  |  |  |  |
|  | | |  | | | | | | | | | | | | | |  |
|  | | | | | | | | | | | | 3 |  | BV |  | 10/01/2020 10:56 AM |  |
|  | | | Well I think, well, I don’t really know how long after you stop taking the Lutetium you’re going to be well for, I don’t really know, and I dare say everybody’s different, but like when (name withheld) and I did chemo you go through, I think you have 7 shots of chemo, or something. I could have had 10 but I wouldn’t have lasted, I don’t think. They said I was sort of going down hill too quickly or something. So, but then, when I stopped taking it, when I stopped doing the chemo about 3 weeks, much longer, 3 or 4 weeks anyway, all of a sudden, my PSA just started going back up again. So, I went through all the crap for sort of nothing. So, I’m actually hoping, I mean when I went, when I applied for the trial, you either got gold standard chemo, or Lutetium, and I was hoping for the Lutetium and I was lucky enough to get on it, so… I actually thought I was pretty lucky. | | | | | | | | | | | | | |  |
|  |  |  |  |  |  |  |  |  |  |  |  |  |  |  |  |  |  |
|  | | |  | | | | | | | | | | | | | |  |
|  | | | | | | | | | | | | 4 |  | BV |  | 15/01/2020 5:03 AM |  |
|  | | | So, I’m actually hoping, I mean when I went, when I applied for the trial, you either got gold standard chemo, or Lutetium, and I was hoping for the Lutetium and I was lucky enough to get on it, so… I actually thought I was pretty lucky. | | | | | | | | | | | | | |  |
|  | | |  | | | | | | | | | | | | | |  |
|  | | | **Codes\\initial motivation for participation\changes to initial motivation for participation** | | | | | | | | | | | | | |  |
|  |  |  |  |  | No |  | 0.0096 |  | 1 |  | | | | | | |  |
|  | | |  |  |  |  |  |  |  |  | | | | | | | |
|  | | | | | | | | | | | | 1 |  | BV |  | 10/01/2020 10:49 AM |  |
|  | | | I: Thinking back on what your initial motivations were for participating in this trial, do you think these motivations have changed?  P1: No, no… | | | | | | | | | | | | | |  |
|  | | |  | | | | | | | | | | | | | |  |
|  | | | **Codes\\initial motivation for participation\failure in previous treatment** | | | | | | | | | | | | | |  |
|  |  |  |  |  | No |  | 0.0671 |  | 3 |  | | | | | | |  |
|  | | |  |  |  |  |  |  |  |  | | | | | | | |
|  | | | | | | | | | | | | 1 |  | BV |  | 10/01/2020 10:22 AM |  |
|  | | | Well, I was actually pretty, pretty ordinary by that stage, because I hadn’t had any treatment for my cancer. I was on another trial drug and it sort of failed and we kept it on, they kept me on it for another 3 or 4 months, and then it took about another 3 months for me to get onto this trial, you know? | | | | | | | | | | | | | |  |
|  | | |  | | | | | | | | | | | | | |  |
|  | | | | | | | | | | | | 2 |  | BV |  | 10/01/2020 10:23 AM |  |
|  | | | So, by that stage I wasn’t really feeling very flash, anyway. | | | | | | | | | | | | | |  |
|  | | |  | | | | | | | | | | | | | |  |
|  | | | | | | | | | | | | | | | | | |
| Formatted Reports\\Coding Summary by File Formatted Report | | | | | | | | | | | Page 35 of 143 | | | | | | |
| 6/10/2022 8:14 AM | | | | | | | | | | | | | | | | | |
|  | | | **Classification** |  | **Aggregate** |  | **Coverage** |  | **Number Of Coding References** |  | | **Reference Number** |  | **Coded By Initials** |  | **Modified On** |  |
|  | | | | | | | | | | | | | | | | | |
|  | | | | | | | | | | | | 3 |  | BV |  | 10/01/2020 10:55 AM |  |
|  | | | Well I think, well, I don’t really know how long after you stop taking the Lutetium you’re going to be well for, I don’t really know, and I dare say everybody’s different, but like when (name withheld) and I did chemo you go through, I think you have 7 shots of chemo, or something. I could have had 10 but I wouldn’t have lasted, I don’t think. They said I was sort of going down hill too quickly or something. So, but then, when I stopped taking it, when I stopped doing the chemo about 3 weeks, much longer, 3 or 4 weeks anyway, all of a sudden, my PSA just started going back up again. So, I went through all the crap for sort of nothing. | | | | | | | | | | | | | |  |
|  | | |  | | | | | | | | | | | | | |  |
|  | | | **Codes\\needing more support** | | | | | | | | | | | | | |  |
|  |  |  |  |  | No |  | 0.0415 |  | 2 |  | | | | | | |  |
|  | | |  |  |  |  |  |  |  |  | | | | | | | |
|  | | | | | | | | | | | | 1 |  | BV |  | 10/01/2020 10:56 AM |  |
|  | | | I: Do you feel like there’s any other support that you might need going forward?  P1: No, not really no. Somebody can come and do the ironing. | | | | | | | | | | | | | |  |
|  | | |  | | | | | | | | | | | | | |  |
|  | | | | | | | | | | | | 2 |  | BV |  | 10/01/2020 10:57 AM |  |
|  | | | So, this is it you know, so there’s few jobs you know, well cooking is one, I never learnt to cook. I’m this old school, because I’m 72, we never sort of cooked, our wives always did all that. They did the ironing and the cooking and all that kind of… Maybe the new modern man is a lot different, but…  I: You’re learning new things by the sounds of it?  P1: But… that’s about it, so somebody, you can send somebody down to do the ironing and all that, that’d be greatly appreciated. | | | | | | | | | | | | | |  |
|  | | |  | | | | | | | | | | | | | |  |
|  | | | **Codes\\quality of life changes** | | | | | | | | | | | | | |  |
|  |  |  |  |  | No |  | 0.0440 |  | 2 |  | | | | | | |  |
|  | | |  |  |  |  |  |  |  |  | | | | | | | |
|  | | | | | | | | | | | | 1 |  | BV |  | 25/03/2020 8:25 AM |  |
|  | | | Well, our quality of life here, has been shot down because my wife just got diagnosed with a melanoma a couple of months ago. | | | | | | | | | | | | | |  |
|  | | |  | | | | | | | | | | | | | |  |
|  | | | | | | | | | | | | 2 |  | BV |  | 25/03/2020 8:25 AM |  |
|  | | | So, you know, so what was going to be, soon as we got a bit better we were going to do something because we’ve been sort trapped in the house, well, our winter down here was absolutely shocking, so, and I wasn’t really feeling flash up to June and of course all of a sudden I’m coming good and (name withheld) was good, and I was crook and now I’m coming good and (name withheld) is crook.  I: Having a bit of a tough time for you both…  P1: Yeah it has, it has, so, sort of really, it really, it sort of, knocks the wind out of your sails | | | | | | | | | | | | | |  |
|  | | |  | | | | | | | | | | | | | |  |
|  | | | **Codes\\quality of life changes\quality of life changes - lutetium** | | | | | | | | | | | | | |  |
|  |  |  |  |  | No |  | 0.0440 |  | 2 |  | | | | | | |  |
|  | | |  |  |  |  |  |  |  |  | | | | | | | |
|  | | | | | | | | | | | | 1 |  | BV |  | 10/01/2020 10:25 AM |  |
|  | | | Well, our quality of life here, has been shot down because my wife just got diagnosed with a melanoma a couple of months ago. | | | | | | | | | | | | | |  |
|  | | |  | | | | | | | | | | | | | |  |
|  | | | | | | | | | | | | 2 |  | BV |  | 10/01/2020 10:27 AM |  |
|  | | | So, you know, so what was going to be, soon as we got a bit better we were going to do something because we’ve been sort trapped in the house, well, our winter down here was absolutely shocking, so, and I wasn’t really feeling flash up to June and of course all of a sudden I’m coming good and (name withheld) was good, and I was crook and now I’m coming good and (name withheld) is crook.  I: Having a bit of a tough time for you both…  P1: Yeah it has, it has, so, sort of really, it really, it sort of, knocks the wind out of your sails | | | | | | | | | | | | | |  |
|  | | |  | | | | | | | | | | | | | |  |
| Formatted Reports\\Coding Summary by File Formatted Report | | | | | | | | | | | Page 36 of 143 | | | | | | |
| 6/10/2022 8:14 AM | | | | | | | | | | | | | | | | | |
|  | | | **Classification** |  | **Aggregate** |  | **Coverage** |  | **Number Of Coding References** |  | | **Reference Number** |  | **Coded By Initials** |  | **Modified On** |  |
|  | | | **Codes\\quality of trialists** | | | | | | | | | | | | | |  |
|  |  |  |  |  | No |  | 0.0251 |  | 2 |  | | | | | | |  |
|  | | |  |  |  |  |  |  |  |  | | | | | | | |
|  | | | | | | | | | | | | 1 |  | BV |  | 10/01/2020 10:46 AM |  |
|  | | | Yes, I do believe it is, yeah… He was in, they all watched me get it last, well, 2 weeks ago. I had to, they took my scans and then I had to wait around you know... They all talked to me, sort of thing. Actually, he was very, very happy with my progress, yeah. | | | | | | | | | | | | | |  |
|  | | |  | | | | | | | | | | | | | |  |
|  | | | | | | | | | | | | 2 |  | BV |  | 10/01/2020 10:57 AM |  |
|  | | | I find all the people are doing the trial very good, you know, I always know that when you have a doctors appointment | | | | | | | | | | | | | |  |
|  | | |  | | | | | | | | | | | | | |  |
|  | | | **Codes\\side effects** | | | | | | | | | | | | | |  |
|  |  |  |  |  | No |  | 0.0886 |  | 5 |  | | | | | | |  |
|  | | |  |  |  |  |  |  |  |  | | | | | | | |
|  | | | | | | | | | | | | 1 |  | BV |  | 25/03/2020 8:26 AM |  |
|  | | | So, when I had it, it sorted had I think, I had a lot of side effects, side effects. I think the steroids and all that, you do get sort of constipated and then sometimes you might have diarrhea, you do have sort of aches and pains in your legs but my left leg especially, I’ve had a lot of aches and pains in my left leg. But, after a couple of weeks everything came fine, and I was really good. | | | | | | | | | | | | | |  |
|  | | |  | | | | | | | | | | | | | |  |
|  | | | | | | | | | | | | 2 |  | BV |  | 25/03/2020 8:26 AM |  |
|  | | | And I did, I did, my platelets were down, instead of having it the next 6 weeks, after 6 weeks, I had my next lot after 8 weeks but apart from that, I feel really good and then of course I’ve had it 2 weeks ago now, or roughly 2 weeks anyway, and I’m feeling really good. | | | | | | | | | | | | | |  |
|  | | |  | | | | | | | | | | | | | |  |
|  | | | | | | | | | | | | 3 |  | BV |  | 25/03/2020 8:26 AM |  |
|  | | | I: Having a bit of a tough time for you both…  P1: Yeah it has, it has, so, sort of really, it really, it sort of, knocks the wind out of your sails | | | | | | | | | | | | | |  |
|  | | |  | | | | | | | | | | | | | |  |
|  | | | | | | | | | | | | 4 |  | BV |  | 25/03/2020 8:26 AM |  |
|  | | | All I know is, the first time I first had it, I got sort of constipated, then of course I then went and got laxatives, and then I overdosed on laxatives, and then went the other way so, I think I probably panic a bit that way. | | | | | | | | | | | | | |  |
|  | | |  | | | | | | | | | | | | | |  |
|  | | | | | | | | | | | | 5 |  | BV |  | 25/03/2020 8:26 AM |  |
|  | | | This time everything is fine, because they give you nausea, nausea pills and, and whatever those other damn things are? … steroids… give you, you take steroids and nausea for 3 days you know? And they upset your bowel a bit, you know, so… But no, I think I’ve got through everything pretty good… | | | | | | | | | | | | | |  |
|  | | |  | | | | | | | | | | | | | |  |
|  | | | **Codes\\side effects\side effects - lutetium** | | | | | | | | | | | | | |  |
|  |  |  |  |  | No |  | 0.0887 |  | 5 |  | | | | | | |  |
|  | | |  |  |  |  |  |  |  |  | | | | | | | |
|  | | | | | | | | | | | | 1 |  | BV |  | 10/01/2020 10:24 AM |  |
|  | | | So, when I had it, it sorted had I think, I had a lot of side effects, side effects. I think the steroids and all that, you do get sort of constipated and then sometimes you might have diarrhea, you do have sort of aches and pains in your legs but my left leg especially, I’ve had a lot of aches and pains in my left leg. But, after a couple of weeks everything came fine, and I was really good. | | | | | | | | | | | | | |  |
|  | | |  | | | | | | | | | | | | | |  |
|  | | | | | | | | | | | | 2 |  | BV |  | 10/01/2020 10:24 AM |  |
|  | | | And I did, I did, my platelets were down, instead of having it the next 6 weeks, after 6 weeks, I had my next lot after 8 weeks but apart from that, I feel really good and then of course I’ve had it 2 weeks ago now, or roughly 2 weeks anyway, and I’m feeling really good. | | | | | | | | | | | | | |  |
|  | | |  | | | | | | | | | | | | | |  |
| Formatted Reports\\Coding Summary by File Formatted Report | | | | | | | | | | | Page 37 of 143 | | | | | | |
| 6/10/2022 8:14 AM | | | | | | | | | | | | | | | | | |
|  | | | **Classification** |  | **Aggregate** |  | **Coverage** |  | **Number Of Coding References** |  | | **Reference Number** |  | **Coded By Initials** |  | **Modified On** |  |
|  | | | | | | | | | | | | | | | | | |
|  | | | | | | | | | | | | 3 |  | BV |  | 10/01/2020 10:27 AM |  |
|  | | | I: Having a bit of a tough time for you both…  P1: Yeah it has, it has, so, sort of really, it really, it sort of, knocks the wind out of your sails | | | | | | | | | | | | | |  |
|  | | |  | | | | | | | | | | | | | |  |
|  | | | | | | | | | | | | 4 |  | BV |  | 10/01/2020 10:45 AM |  |
|  | | | All I know is, the first time I first had it, I got sort of constipated, then of course I then went and got laxatives, and then I overdosed on laxatives, and then went the other way so, I think I probably panic a bit that way. | | | | | | | | | | | | | |  |
|  | | |  | | | | | | | | | | | | | |  |
|  | | | | | | | | | | | | 5 |  | BV |  | 10/01/2020 10:45 AM |  |
|  | | | This time everything is fine, because they give you nausea, nausea pills and, and whatever those other damn things are? … steroids… give you, you take steroids and nausea for 3 days you know? And they upset your bowel a bit, you know, so… But no, I think I’ve got through everything pretty good… | | | | | | | | | | | | | |  |
|  | | |  | | | | | | | | | | | | | |  |
|  | | | **Codes\\struggling with treatment** | | | | | | | | | | | | | |  |
|  |  |  |  |  | No |  | 0.0168 |  | 1 |  | | | | | | |  |
|  | | |  |  |  |  |  |  |  |  | | | | | | | |
|  | | | | | | | | | | | | 1 |  | BV |  | 10/01/2020 10:49 AM |  |
|  | | | Well it probably hasn’t affected me, but I am a bit tired. I do believe, not only because, I think it’s also, you do get a bit stressed or something you know, or a bit worried or whatever it is you know, and I think that takes a bit out of you as well. | | | | | | | | | | | | | |  |
|  | | |  | | | | | | | | | | | | | |  |
|  | | | **Codes\\struggling with treatment\symptom fluctuations** | | | | | | | | | | | | | |  |
|  |  |  |  |  | No |  | 0.0357 |  | 1 |  | | | | | | |  |
|  | | |  |  |  |  |  |  |  |  | | | | | | | |
|  | | | | | | | | | | | | 1 |  | BV |  | 10/01/2020 10:27 AM |  |
|  | | | So, you know, so what was going to be, soon as we got a bit better we were going to do something because we’ve been sort trapped in the house, well, our winter down here was absolutely shocking, so, and I wasn’t really feeling flash up to June and of course all of a sudden I’m coming good and (name withheld) was good, and I was crook and now I’m coming good and (name withheld) is crook.  I: Having a bit of a tough time for you both…  P1: Yeah it has, it has, so, sort of really, it really, it sort of, knocks the wind out of your sails | | | | | | | | | | | | | |  |
|  | | |  | | | | | | | | | | | | | |  |
|  | | | **Codes\\support from family** | | | | | | | | | | | | | |  |
|  |  |  |  |  | No |  | 0.0094 |  | 1 |  | | | | | | |  |
|  | | |  |  |  |  |  |  |  |  | | | | | | | |
|  | | | | | | | | | | | | 1 |  | BV |  | 10/01/2020 10:48 AM |  |
|  | | | I got, well, I did have, a lot of support here, and I have to give my support now to (name withheld) because she, she’s so fatigued you know? | | | | | | | | | | | | | |  |
|  | | |  | | | | | | | | | | | | | |  |
|  | | | **Codes\\support from partner\supporting partner** | | | | | | | | | | | | | |  |
|  |  |  |  |  | No |  | 0.0260 |  | 1 |  | | | | | | |  |
|  | | |  |  |  |  |  |  |  |  | | | | | | | |
|  | | | | | | | | | | | | 1 |  | BV |  | 10/01/2020 10:48 AM |  |
|  | | | I got, well, I did have, a lot of support here, and I have to give my support now to (name withheld) because she, she’s so fatigued you know? So, she sleeps most of the time so, I’ve had to take on some house, some house duties now, which is absolutely foreign to me. I never used to do, I never used to do shopping, or anything like that. So, now I’ve had to take on some of that as well. | | | | | | | | | | | | | |  |
|  | | |  | | | | | | | | | | | | | |  |
| Formatted Reports\\Coding Summary by File Formatted Report | | | | | | | | | | | Page 38 of 143 | | | | | | |
| 6/10/2022 8:14 AM | | | | | | | | | | | | | | | | | |
|  | | | **Classification** |  | **Aggregate** |  | **Coverage** |  | **Number Of Coding References** |  | | **Reference Number** |  | **Coded By Initials** |  | **Modified On** |  |
|  | | | **Codes\\travelling to treatment\travelling to treatment challenging** | | | | | | | | | | | | | |  |
|  |  |  |  |  | No |  | 0.0342 |  | 3 |  | | | | | | |  |
|  | | |  |  |  |  |  |  |  |  | | | | | | | |
|  | | | | | | | | | | | | 1 |  | BV |  | 10/01/2020 10:46 AM |  |
|  | | | Well, the only thing I can tell you, is the traffic. | | | | | | | | | | | | | |  |
|  | | |  | | | | | | | | | | | | | |  |
|  | | | | | | | | | | | | 2 |  | BV |  | 10/01/2020 10:47 AM |  |
|  | | | The traffic between Lara and Melbourne, whenever I go. So that’s it, that’s the worst part right, getting there | | | | | | | | | | | | | |  |
|  | | |  | | | | | | | | | | | | | |  |
|  | | | | | | | | | | | | 3 |  | BV |  | 10/01/2020 10:47 AM |  |
|  | | | If you have it really early in the morning, you really have to give yourself 2 hours but, if you have it say, round that lunchtime or whatever appointment, even 10 o’clock or 11 o’clock, it only takes roughly an hour, you know, so? I know that’s nothing to do with the medical or anything like that, but that’s the only thing I find that’s really bad. | | | | | | | | | | | | | |  |
|  | | |  | | | | | | | | | | | | | |  |
|  | | | **Codes\\treatments been good** | | | | | | | | | | | | | |  |
|  |  |  |  |  | No |  | 0.0355 |  | 2 |  | | | | | | |  |
|  | | |  |  |  |  |  |  |  |  | | | | | | | |
|  | | | | | | | | | | | | 1 |  | BV |  | 10/01/2020 10:24 AM |  |
|  | | | And I did, I did, my platelets were down, instead of having it the next 6 weeks, after 6 weeks, I had my next lot after 8 weeks but apart from that, I feel really good and then of course I’ve had it 2 weeks ago now, or roughly 2 weeks anyway, and I’m feeling really good. | | | | | | | | | | | | | |  |
|  | | |  | | | | | | | | | | | | | |  |
|  | | | | | | | | | | | | 2 |  | BV |  | 10/01/2020 10:46 AM |  |
|  | | | Yes, I do believe it is, yeah… He was in, they all watched me get it last, well, 2 weeks ago. I had to, they took my scans and then I had to wait around you know... They all talked to me, sort of thing. Actually, he was very, very happy with my progress, yeah. | | | | | | | | | | | | | |  |
|  | | |  | | | | | | | | | | | | | |  |
|  | | | **Codes\\treatments left** | | | | | | | | | | | | | |  |
|  |  |  |  |  | No |  | 0.0643 |  | 2 |  | | | | | | |  |
|  | | |  |  |  |  |  |  |  |  | | | | | | | |
|  | | | | | | | | | | | | 1 |  | BV |  | 10/01/2020 10:59 AM |  |
|  | | | He said last, you know, well I think it’s not quite 2 weeks, must be getting very close to 2 weeks, but I think probably 2 weeks, on Thursday. He said that even, and I’m thinking, I thought this was rather funny, well, this is not really my field, even though he was the boss of the whole trial, he said, this is not my field but I think you’ll probably only have one more, one more of this and, but that’s up to, her name’s (name withheld) she’s a doctor I always see on Tuesday. She’s the one that tells you whether, yes, you can have another go at it, or not, you know… and that’s what he said so, I will, I do believe I’ll be going there next Tuesday, and that’s when I’ll find out if I’m having another one 3 weeks after that, or that could be it, I don’t know. | | | | | | | | | | | | | |  |
|  | | |  | | | | | | | | | | | | | |  |
|  | | | | | | | | | | | | 2 |  | BV |  | 10/01/2020 11:00 AM |  |
|  | | | So, yeah, I could be having another go, or maybe not, I don’t know, but he felt that yes, because it must get to a certain point where your side effects probably outweigh your benefits, you know, maybe. | | | | | | | | | | | | | |  |
|  | | |  | | | | | | | | | | | | | |  |
|  | | | | | | | | | | | | | | | | | |
| Formatted Reports\\Coding Summary by File Formatted Report | | | | | | | | | | | Page 39 of 143 | | | | | | |
| 6/10/2022 8:14 AM | | | | | | | | | | | | | | | | | |
|  | | | **Classification** |  | **Aggregate** |  | **Coverage** |  | **Number Of Coding References** |  | | **Reference Number** |  | **Coded By Initials** |  | **Modified On** |  |
|  | | | **Codes\\trying to keep life uncomplicated** | | | | | | | | | | | | | |  |
|  |  |  |  |  | No |  | 0.0132 |  | 1 |  | | | | | | |  |
|  | | |  |  |  |  |  |  |  |  | | | | | | | |
|  | | | | | | | | | | | | 1 |  | BV |  | 10/01/2020 10:28 AM |  |
|  | | | Oh no. I always try to keep a, I try to keep a, I don’t normally like to make my life too complicated. So, I try to keep my life uncomplicated. No, apart from that no, none. I think I’ve been fine. | | | | | | | | | | | | | |  |
|  | | |  | | | | | | | | | | | | | |  |
|  | | | **Codes\\uncertainty of the future** | | | | | | | | | | | | | |  |
|  |  |  |  |  | No |  | 0.0064 |  | 2 |  | | | | | | |  |
|  | | |  |  |  |  |  |  |  |  | | | | | | | |
|  | | | | | | | | | | | | 1 |  | BV |  | 10/01/2020 10:56 AM |  |
|  | | | I still think I’m lucky, but I don’t know, you don’t know what’s down the track… we don’t know. | | | | | | | | | | | | | |  |
|  | | |  | | | | | | | | | | | | | |  |
|  | | | | | | | | | | | | 2 |  | BV |  | 15/01/2020 5:03 AM |  |
|  | | | I still think I’m lucky, but I don’t know, you don’t know what’s down the track… we don’t know. | | | | | | | | | | | | | |  |
|  | | |  | | | | | | | | | | | | | |  |
|  | | | **Codes\\unsure of how long treatment effects will last** | | | | | | | | | | | | | |  |
|  |  |  |  |  | No |  | 0.0426 |  | 2 |  | | | | | | |  |
|  | | |  |  |  |  |  |  |  |  | | | | | | | |
|  | | | | | | | | | | | | 1 |  | BV |  | 10/01/2020 10:55 AM |  |
|  | | | Well I think, well, I don’t really know how long after you stop taking the Lutetium you’re going to be well for, I don’t really know, and I dare say everybody’s different, but like when (name withheld) and I did chemo you go through, I think you have 7 shots of chemo, or something. I could have had 10 but I wouldn’t have lasted, I don’t think. They said I was sort of going down hill too quickly or something. | | | | | | | | | | | | | |  |
|  | | |  | | | | | | | | | | | | | |  |
|  | | | | | | | | | | | | 2 |  | BV |  | 10/01/2020 10:55 AM |  |
|  | | | So, but then, when I stopped taking it, when I stopped doing the chemo about 3 weeks, much longer, 3 or 4 weeks anyway, all of a sudden, my PSA just started going back up again. So, I went through all the crap for sort of nothing. | | | | | | | | | | | | | |  |
|  | | |  | | | | | | | | | | | | | |  |
|  | | | **Codes\\waiting for doctors** | | | | | | | | | | | | | |  |
|  |  |  |  |  | No |  | 0.0238 |  | 1 |  | | | | | | |  |
|  | | |  |  |  |  |  |  |  |  | | | | | | | |
|  | | | | | | | | | | | | 1 |  | BV |  | 10/01/2020 10:58 AM |  |
|  | | | I always know that when you have a doctors appointment, normally it takes an hour to get to see them, that’s it, but doesn’t matter doctors are always, doctors are the same everywhere. Some days are quicker. I always give myself an hour. If your appointment is 10 o’clock, you get there at 10 o’clock and it’s probably an hour before you get to see the doctor. | | | | | | | | | | | | | |  |
|  | | |  | | | | | | | | | | | | | |  |
|  | | | | | | | | | | | | | | | | | |
| Formatted Reports\\Coding Summary by File Formatted Report | | | | | | | | | | | Page 40 of 143 | | | | | | |
| 6/10/2022 8:14 AM | | | | | | | | | | | | | | | | | |
|  | | | **Classification** |  | **Aggregate** |  | **Coverage** |  | **Number Of Coding References** |  | | **Reference Number** |  | **Coded By Initials** |  | **Modified On** |  |
|  | **Files\\Transcription 2_011** | | | | | | | | | | | | | | | |  |
|  | | **Code** | | | | | | | | | | | | | | |  |
|  | | | **Codes\\comparing progress to others** | | | | | | | | | | | | | |  |
|  |  |  |  |  | No |  | 0.0126 |  | 1 |  | | | | | | |  |
|  | | |  |  |  |  |  |  |  |  | | | | | | | |
|  | | | | | | | | | | | | 1 |  | BV |  | 15/01/2020 4:47 AM |  |
|  | | | That’s correct yeah, yeah like this half way through, and then yeah, you know, I suppose as long as they’re happy with the decrease in activity of the cancers, then I’ll continue on but I know I was in with another fellow and he’s nearly done, completely clear and I’m still quite significant. | | | | | | | | | | | | | |  |
|  | | |  | | | | | | | | | | | | | |  |
|  | | | **Codes\\coping strategies** | | | | | | | | | | | | | |  |
|  |  |  |  |  | No |  | 0.0116 |  | 1 |  | | | | | | |  |
|  | | |  |  |  |  |  |  |  |  | | | | | | | |
|  | | | | | | | | | | | | 1 |  | BV |  | 14/01/2020 11:46 AM |  |
|  | | | I honestly, I don’t know where, what’s happening to me. So, as far as I, I’m concerned, I’m not going backward, as far as my general day to day health and everything, I’m quite well. It’s just the numbers aren’t matching up to me. I’m a bit of an anomaly, sort of thing. | | | | | | | | | | | | | |  |
|  | | |  | | | | | | | | | | | | | |  |
|  | | | **Codes\\coping strategies\coping okay** | | | | | | | | | | | | | |  |
|  |  |  |  |  | No |  | 0.0018 |  | 1 |  | | | | | | |  |
|  | | |  |  |  |  |  |  |  |  | | | | | | | |
|  | | | | | | | | | | | | 1 |  | BV |  | 14/01/2020 11:25 AM |  |
|  | | | I think I’m coping quite well with it all. | | | | | | | | | | | | | |  |
|  | | |  | | | | | | | | | | | | | |  |
|  | | | **Codes\\doctor happy with progress** | | | | | | | | | | | | | |  |
|  |  |  |  |  | No |  | 0.0126 |  | 1 |  | | | | | | |  |
|  | | |  |  |  |  |  |  |  |  | | | | | | | |
|  | | | | | | | | | | | | 1 |  | BV |  | 15/01/2020 4:48 AM |  |
|  | | | That’s correct yeah, yeah like this half way through, and then yeah, you know, I suppose as long as they’re happy with the decrease in activity of the cancers, then I’ll continue on but I know I was in with another fellow and he’s nearly done, completely clear and I’m still quite significant. | | | | | | | | | | | | | |  |
|  | | |  | | | | | | | | | | | | | |  |
|  | | | **Codes\\fear of the unknown** | | | | | | | | | | | | | |  |
|  |  |  |  |  | No |  | 0.0123 |  | 1 |  | | | | | | |  |
|  | | |  |  |  |  |  |  |  |  | | | | | | | |
|  | | | | | | | | | | | | 1 |  | BV |  | 15/01/2020 5:32 AM |  |
|  | | | I think I’m coping quite well with it all. I mean, the little bit of unknown that we’re going through now, and the uncertainty of why my PSA isn’t dropping, is a little bit of a concern and that causes me a little bit of worry, because you know, you always worry why isn’t it going down. | | | | | | | | | | | | | |  |
|  | | |  | | | | | | | | | | | | | |  |
|  | | | | | | | | | | | | | | | | | |
| Formatted Reports\\Coding Summary by File Formatted Report | | | | | | | | | | | Page 41 of 143 | | | | | | |
| 6/10/2022 8:14 AM | | | | | | | | | | | | | | | | | |
|  | | | **Classification** |  | **Aggregate** |  | **Coverage** |  | **Number Of Coding References** |  | | **Reference Number** |  | **Coded By Initials** |  | **Modified On** |  |
|  | | | **Codes\\feeling like an anomaly** | | | | | | | | | | | | | |  |
|  |  |  |  |  | No |  | 0.0442 |  | 5 |  | | | | | | |  |
|  | | |  |  |  |  |  |  |  |  | | | | | | | |
|  | | | | | | | | | | | | 1 |  | BV |  | 15/01/2020 5:17 AM |  |
|  | | | Oh yeah. It’s just, it’s purely… They’ve got to have time to formulate some sort of a read on me, and even I can’t get one on me and I’m pretty good. | | | | | | | | | | | | | |  |
|  | | |  | | | | | | | | | | | | | |  |
|  | | | | | | | | | | | | 2 |  | BV |  | 15/01/2020 4:49 AM |  |
|  | | | I honestly, I don’t know where, what’s happening to me. So, as far as I, I’m concerned, I’m not going backward, as far as my general day to day health and everything, I’m quite well. It’s just the numbers aren’t matching up to me. I’m a bit of an anomaly, sort of thing. | | | | | | | | | | | | | |  |
|  | | |  | | | | | | | | | | | | | |  |
|  | | | | | | | | | | | | 3 |  | BV |  | 14/01/2020 11:46 AM |  |
|  | | | And I’ve been an anomaly all the way through. If there’s a different way, or an odd way to do or be something, it seems to find me. | | | | | | | | | | | | | |  |
|  | | |  | | | | | | | | | | | | | |  |
|  | | | | | | | | | | | | 4 |  | BV |  | 14/01/2020 11:46 AM |  |
|  | | | I: You’re unique.  P: Yeah, that’s probably a very good word. I’m very unique. | | | | | | | | | | | | | |  |
|  | | |  | | | | | | | | | | | | | |  |
|  | | | | | | | | | | | | 5 |  | BV |  | 14/01/2020 12:12 PM |  |
|  | | | Yeah, so to get back off that, and I’ve had a reduction in my pain pills as well, and I haven’t had to go back up, or anything at this stage, so you know, so, as far as all that goes, and that’s why I’m a bit of an anomaly because I’m, there’s so many things saying I’m heading in the right direction, it’s just my PSA saying I’m not, but all my other bloods and everything are really quite good. | | | | | | | | | | | | | |  |
|  | | |  | | | | | | | | | | | | | |  |
|  | | | **Codes\\feeling lucky** | | | | | | | | | | | | | |  |
|  |  |  |  |  | No |  | 0.0094 |  | 1 |  | | | | | | |  |
|  | | |  |  |  |  |  |  |  |  | | | | | | | |
|  | | | | | | | | | | | | 1 |  | BV |  | 15/01/2020 5:00 AM |  |
|  | | | Every time you have a treatment, you just thank your lucky stars, that you’ve got this available, even if it does make you crook and I’m just so thankful that other people have paved the way for me to have this treatment | | | | | | | | | | | | | |  |
|  | | |  | | | | | | | | | | | | | |  |
|  | | | **Codes\\financial impact** | | | | | | | | | | | | | |  |
|  |  |  |  |  | No |  | 0.1092 |  | 6 |  | | | | | | |  |
|  | | |  |  |  |  |  |  |  |  | | | | | | | |
|  | | | | | | | | | | | | 1 |  | BV |  | 14/01/2020 11:26 AM |  |
|  | | | Well, yeah, I mean, because I’m not working, we’re starting to you know, we’ve been twelve months now in the new house, so we haven’t had financial aid from my super with the income protection insurance, that sort of thing so, we’ve sort of been going by ourselves for a year now and we got rid of all our debt. It absolutely took a lot of stress and everything away, but we’re just starting to realise, we just don’t make enough money to make ends meet, year to year. | | | | | | | | | | | | | |  |
|  | | |  | | | | | | | | | | | | | |  |
|  | | | | | | | | | | | | 2 |  | BV |  | 14/01/2020 11:27 AM |  |
|  | | | So, we’re having to dip into my life insurance, just to cover day to day costs, which we were hoping we wouldn’t have to do. And then of course, that means extra trips down to Melbourne and staying over, and all that sort of stuff all adds to the financial pressures, for sure. But I mean, we just have to do it, you know? If I want to be here, we have to take the hit somewhere. | | | | | | | | | | | | | |  |
|  | | |  | | | | | | | | | | | | | |  |
|  | | | | | | | | | | | | 3 |  | BV |  | 14/01/2020 11:28 AM |  |
|  | | | So, I go down on the Tuesday to see the girls, like the first clinical visit by myself, because it’s just basically an interview like this, so, and that just means (name withheld) doesn’t lose a days pay which it helps, and then she, because we have Thursday, Friday she’ll lose a days pay on the Thursday, and then she doesn’t work on a Friday. So yeah, so it’s, we’re just trying to arrange it. | | | | | | | | | | | | | |  |
|  | | |  | | | | | | | | | | | | | |  |
| Formatted Reports\\Coding Summary by File Formatted Report | | | | | | | | | | | Page 42 of 143 | | | | | | |
| 6/10/2022 8:14 AM | | | | | | | | | | | | | | | | | |
|  | | | **Classification** |  | **Aggregate** |  | **Coverage** |  | **Number Of Coding References** |  | | **Reference Number** |  | **Coded By Initials** |  | **Modified On** |  |
|  | | | | | | | | | | | | | | | | | |
|  | | | | | | | | | | | | 4 |  | BV |  | 15/01/2020 5:14 AM |  |
|  | | | I mean, we have to stay over the night because I’m radioactive, you know, it’s probably just a, and it’s easier to stay, then have to go home and then get up first thing in the morning and then come back down and deal with the traffic and all that sort of stuff.  I: Exactly. Do you stay close by or?  P: Yeah, directly opposite the Peter Mac.  I: Great. So, you’re not paying for parking, or anything like that?  P: Ah no, that’s part of the, part of the accommodation.  I: Great. No, I was asking if you were…  P: It’s twenty dollars.  I: Yep. I see, so you still kind of have that as well…  P Yeah, it’s $270 for the night, with parking. | | | | | | | | | | | | | |  |
|  |  |  |  |  |  |  |  |  |  |  |  |  |  |  |  |  |  |
|  | | |  | | | | | | | | | | | | | |  |
|  | | | | | | | | | | | | 5 |  | BV |  | 14/01/2020 11:32 AM |  |
|  | | | That, well, that and just the fuel and everything you know, and getting you know, you just putting the k’s on the car and you get the car serviced and yeah, you just notice it a little, but I mean that’s what it is you know, we’re used to travelling anyway because we live out of a big city so, if you need to do something, or want to go see something, most of the time you have to pack up and go to Melbourne anyway. So, it’s not uncommon to us, but just because I’m not working, you just can’t, it’s getting a bit harder, to cover all these costs. | | | | | | | | | | | | | |  |
|  | | |  | | | | | | | | | | | | | |  |
|  | | | | | | | | | | | | 6 |  | BV |  | 14/01/2020 11:33 AM |  |
|  | | | P: Oh…and we’ve lost our healthcare card…  I: Oh, okay.  P: …as well. So, that really hurt too, last month. | | | | | | | | | | | | | |  |
|  | | |  | | | | | | | | | | | | | |  |
|  | | | **Codes\\financial impact\hospital parking** | | | | | | | | | | | | | |  |
|  |  |  |  |  | No |  | 0.1142 |  | 3 |  | | | | | | |  |
|  | | |  |  |  |  |  |  |  |  | | | | | | | |
|  | | | | | | | | | | | | 1 |  | BV |  | 15/01/2020 5:15 AM |  |
|  | | | I mean, we have to stay over the night because I’m radioactive, you know, it’s probably just a, and it’s easier to stay, then have to go home and then get up first thing in the morning and then come back down and deal with the traffic and all that sort of stuff.  I: Exactly. Do you stay close by or?  P: Yeah, directly opposite the Peter Mac.  I: Great. So, you’re not paying for parking, or anything like that?  P: Ah no, that’s part of the, part of the accommodation.  I: Great. No, I was asking if you were…  P: It’s twenty dollars.  I: Yep. I see, so you still kind of have that as well…  P Yeah, it’s $270 for the night, with parking. | | | | | | | | | | | | | |  |
|  |  |  |  |  |  |  |  |  |  |  |  |  |  |  |  |  |  |
|  | | |  | | | | | | | | | | | | | |  |
|  | | | | | | | | | | | | 2 |  | BV |  | 14/01/2020 12:25 PM |  |
|  | | | Now, that sort of information would be absolutely invaluable at the start, you know, like as part of the pack they give you, the information about the Lutetium and so forth to take home and read, but if part of that, it would have been really nice, to have some information about that sort of thing, you know. People who do travel over the hundred k’s or whatever it’s meant to be, and have to stay down, because we didn’t realise we’d have to stay down as often as we have, as far as, you know, we just thought, we’ll just go up and back but then once they explained the Lutetium, how you know, it’s really quite radioactive for 24 hours and that you know, I need to keep my space away from people. Or they may think that we should just be able to walk over to an apartment, and I sit on one couch and (name withheld) sits on the other, and you know, we keep away from, sort of, people just for that 24 hours. Yeah, you just didn’t realise what that entailed, as far as having to stay over and find accommodation, parking, all that kind of stuff. It would be nice if, and even just to travel down for the clinical visits and stuff like that, yeah just things like that, and I know when we, when we go to Geelong to my oncologist, the oncology girls give us a parking ticket so that we can validate our parking in their carpark and it’s all free.  I: That’s useful.  P: Yeah, it would be fantastic, rather than having to try and find cheap parking, you know, somewhere. So, yeah, so, that sort of, little things like that. I don’t know how that would work in your situation, as in Peter Mac, because it’s such a large hospital and it’s all dedicated to you know, there’s just so much oncology, it’s the whole hospital, it’s not just one little part of it. | | | | | | | | | | | | | |  |
|  |  |  |  |  |  |  |  |  |  |  |  |  |  |  |  |  |  |
|  |  |  |  |  |  |  |  |  |  |  |  |  |  |  |  |  |  |
|  | | |  | | | | | | | | | | | | | |  |
|  | | | | | | | | | | | | 3 |  | BV |  | 14/01/2020 12:25 PM |  |
|  | | | Yeah, that would be, that would be good, especially for country people because honestly, we had no idea about, you know, how hard it will be to park and, you know, we just worry about having to leave four hours for appointments, just in case we hit traffic. | | | | | | | | | | | | | |  |
|  | | |  | | | | | | | | | | | | | |  |
| Formatted Reports\\Coding Summary by File Formatted Report | | | | | | | | | | | Page 43 of 143 | | | | | | |
| 6/10/2022 8:14 AM | | | | | | | | | | | | | | | | | |
|  | | | **Classification** |  | **Aggregate** |  | **Coverage** |  | **Number Of Coding References** |  | | **Reference Number** |  | **Coded By Initials** |  | **Modified On** |  |
|  | | | **Codes\\financial impact\needing accomodation** | | | | | | | | | | | | | |  |
|  |  |  |  |  | No |  | 0.1287 |  | 2 |  | | | | | | |  |
|  | | |  |  |  |  |  |  |  |  | | | | | | | |
|  | | | | | | | | | | | | 1 |  | BV |  | 14/01/2020 11:32 AM |  |
|  | | | I mean, we have to stay over the night because I’m radioactive, you know, it’s probably just a, and it’s easier to stay, then have to go home and then get up first thing in the morning and then come back down and deal with the traffic and all that sort of stuff.  I: Exactly. Do you stay close by or?  P: Yeah, directly opposite the Peter Mac.  I: Great. So, you’re not paying for parking, or anything like that?  P: Ah no, that’s part of the, part of the accommodation.  I: Great. No, I was asking if you were…  P: It’s twenty dollars.  I: Yep. I see, so you still kind of have that as well…  P Yeah, it’s $270 for the night, with parking. | | | | | | | | | | | | | |  |
|  |  |  |  |  |  |  |  |  |  |  |  |  |  |  |  |  |  |
|  | | |  | | | | | | | | | | | | | |  |
|  | | | | | | | | | | | | 2 |  | BV |  | 14/01/2020 12:25 PM |  |
|  | | | Well, possibly. We were talking to someone, I can’t remember who actually mentioned it to us, but they did mention, that there is someone at the hospital that can help with accommodation. Because we were talking about this to someone and they said, oh now I think the hospital has got access to, or is part of the Medicare, or something you know, to do with the travel or accommodation, that we can claim it somewhere along the line. Which, we only just got that information and because I’ve been a bit crook, we haven’t followed up on it just yet, but we got a name of someone from the hospital. Now, that sort of information would be absolutely invaluable at the start, you know, like as part of the pack they give you, the information about the Lutetium and so forth to take home and read, but if part of that, it would have been really nice, to have some information about that sort of thing, you know. People who do travel over the hundred k’s or whatever it’s meant to be, and have to stay down, because we didn’t realise we’d have to stay down as often as we have, as far as, you know, we just thought, we’ll just go up and back but then once they explained the Lutetium, how you know, it’s really quite radioactive for 24 hours and that you know, I need to keep my space away from people. Or they may think that we should just be able to walk over to an apartment, and I sit on one couch and (name withheld) sits on the other, and you know, we keep away from, sort of, people just for that 24 hours. Yeah, you just didn’t realise what that entailed, as far as having to stay over and find accommodation, parking, all that kind of stuff. It would be nice if, and even just to travel down for the clinical visits and stuff like that, yeah just things like that, and I know when we, when we go to Geelong to my oncologist, the oncology girls give us a parking ticket so that we can validate our parking in their carpark and it’s all free.  I: That’s useful.  P: Yeah, it would be fantastic, rather than having to try and find cheap parking, you know, somewhere. So, yeah, so, that sort of, little things like that. I don’t know how that would work in your situation, as in Peter Mac, because it’s such a large hospital and it’s all dedicated to you know, there’s just so much oncology, it’s the whole hospital, it’s not just one little part of it. | | | | | | | | | | | | | |  |
|  |  |  |  |  |  |  |  |  |  |  |  |  |  |  |  |  |  |
|  |  |  |  |  |  |  |  |  |  |  |  |  |  |  |  |  |  |
|  | | |  | | | | | | | | | | | | | |  |
|  | | | **Codes\\happy about participating** | | | | | | | | | | | | | |  |
|  |  |  |  |  | No |  | 0.0010 |  | 1 |  | | | | | | |  |
|  | | |  |  |  |  |  |  |  |  | | | | | | | |
|  | | | | | | | | | | | | 1 |  | BV |  | 14/01/2020 12:19 PM |  |
|  | | | Yes. One hundred percent | | | | | | | | | | | | | |  |
|  | | |  | | | | | | | | | | | | | |  |
|  | | | **Codes\\hope** | | | | | | | | | | | | | |  |
|  |  |  |  |  | No |  | 0.0452 |  | 4 |  | | | | | | |  |
|  | | |  |  |  |  |  |  |  |  | | | | | | | |
|  | | | | | | | | | | | | 1 |  | BV |  | 14/01/2020 11:26 AM |  |
|  | | | I mean, the little bit of unknown that we’re going through now, and the uncertainty of why my PSA isn’t dropping, is a little bit of a concern and that causes me a little bit of worry, because you know, you always worry why isn’t it going down. But they’re saying it can, you know, stay up for a while before it goes down so, we’re hoping we’ll see a change in this blood test, coming up in a couple of weeks. | | | | | | | | | | | | | |  |
|  | | |  | | | | | | | | | | | | | |  |
|  | | | | | | | | | | | | 2 |  | BV |  | 14/01/2020 11:27 AM |  |
|  | | | So, we’re having to dip into my life insurance, just to cover day to day costs, which we were hoping we wouldn’t have to do. | | | | | | | | | | | | | |  |
|  | | |  | | | | | | | | | | | | | |  |
| Formatted Reports\\Coding Summary by File Formatted Report | | | | | | | | | | | Page 44 of 143 | | | | | | |
| 6/10/2022 8:14 AM | | | | | | | | | | | | | | | | | |
|  | | | **Classification** |  | **Aggregate** |  | **Coverage** |  | **Number Of Coding References** |  | | **Reference Number** |  | **Coded By Initials** |  | **Modified On** |  |
|  | | | | | | | | | | | | | | | | | |
|  | | | | | | | | | | | | 3 |  | BV |  | 14/01/2020 12:13 PM |  |
|  | | | Yeah, I don’t know. It’ll be interesting to see what sort of scan they want me to have if required. I’m just hoping that my bloods finally decide to turn and start heading down and then that would just, that would make everything fit nicely then. | | | | | | | | | | | | | |  |
|  | | |  | | | | | | | | | | | | | |  |
|  | | | | | | | | | | | | 4 |  | BV |  | 14/01/2020 12:28 PM |  |
|  | | | Yeah, yeah, I’m hoping. I’m hoping we get, you know, they did say to us that they would prefer it to happen slowly rather than quickly because that’s when you get the longevity of the treatment so, yeah, we’re still hanging in there, that everything’s going really well. | | | | | | | | | | | | | |  |
|  | | |  | | | | | | | | | | | | | |  |
|  | | | **Codes\\hope\hoping blood count improves** | | | | | | | | | | | | | |  |
|  |  |  |  |  | No |  | 0.0126 |  | 2 |  | | | | | | |  |
|  | | |  |  |  |  |  |  |  |  | | | | | | | |
|  | | | | | | | | | | | | 1 |  | BV |  | 15/01/2020 9:54 AM |  |
|  | | | we’re hoping we’ll see a change in this blood test | | | | | | | | | | | | | |  |
|  | | |  | | | | | | | | | | | | | |  |
|  | | | | | | | | | | | | 2 |  | BV |  | 15/01/2020 9:55 AM |  |
|  | | | Yeah, I don’t know. It’ll be interesting to see what sort of scan they want me to have if required. I’m just hoping that my bloods finally decide to turn and start heading down and then that would just, that would make everything fit nicely then | | | | | | | | | | | | | |  |
|  | | |  | | | | | | | | | | | | | |  |
|  | | | **Codes\\hope\hoping PSA dropped further** | | | | | | | | | | | | | |  |
|  |  |  |  |  | No |  | 0.0116 |  | 1 |  | | | | | | |  |
|  | | |  |  |  |  |  |  |  |  | | | | | | | |
|  | | | | | | | | | | | | 1 |  | BV |  | 15/01/2020 9:55 AM |  |
|  | | | Yeah, yeah, I’m hoping. I’m hoping we get, you know, they did say to us that they would prefer it to happen slowly rather than quickly because that’s when you get the longevity of the treatment so, yeah, we’re still hanging in there, that everything’s going really well. | | | | | | | | | | | | | |  |
|  | | |  | | | | | | | | | | | | | |  |
|  | | | **Codes\\hope\hoping to keep finances down** | | | | | | | | | | | | | |  |
|  |  |  |  |  | No |  | 0.0053 |  | 1 |  | | | | | | |  |
|  | | |  |  |  |  |  |  |  |  | | | | | | | |
|  | | | | | | | | | | | | 1 |  | BV |  | 15/01/2020 9:54 AM |  |
|  | | | So, we’re having to dip into my life insurance, just to cover day to day costs, which we were hoping we wouldn’t have to do. | | | | | | | | | | | | | |  |
|  | | |  | | | | | | | | | | | | | |  |
|  | | | **Codes\\initial motivation for participation\failure in previous treatment** | | | | | | | | | | | | | |  |
|  |  |  |  |  | No |  | 0.0121 |  | 1 |  | | | | | | |  |
|  | | |  |  |  |  |  |  |  |  | | | | | | | |
|  | | | | | | | | | | | | 1 |  | BV |  | 14/01/2020 11:36 AM |  |
|  | | | In the fact that I’m not having to do chemo. I’m not getting knocked around like I was with chemo. It’s completely different, as far as the side effects and that go. It’s so much gentler on your body. So, as far as all that goes, I’m so thankful and that’s a huge positive, you know | | | | | | | | | | | | | |  |
|  | | |  | | | | | | | | | | | | | |  |
| Formatted Reports\\Coding Summary by File Formatted Report | | | | | | | | | | | Page 45 of 143 | | | | | | |
| 6/10/2022 8:14 AM | | | | | | | | | | | | | | | | | |
|  | | | **Classification** |  | **Aggregate** |  | **Coverage** |  | **Number Of Coding References** |  | | **Reference Number** |  | **Coded By Initials** |  | **Modified On** |  |
|  | | | **Codes\\needing more information** | | | | | | | | | | | | | |  |
|  |  |  |  |  | No |  | 0.1116 |  | 2 |  | | | | | | |  |
|  | | |  |  |  |  |  |  |  |  | | | | | | | |
|  | | | | | | | | | | | | 1 |  | BV |  | 14/01/2020 12:25 PM |  |
|  | | | We were talking to someone, I can’t remember who actually mentioned it to us, but they did mention, that there is someone at the hospital that can help with accommodation. Because we were talking about this to someone and they said, oh now I think the hospital has got access to, or is part of the Medicare, or something you know, to do with the travel or accommodation, that we can claim it somewhere along the line. Which, we only just got that information and because I’ve been a bit crook, we haven’t followed up on it just yet, but we got a name of someone from the hospital. Now, that sort of information would be absolutely invaluable at the start, you know, like as part of the pack they give you, the information about the Lutetium and so forth to take home and read, but if part of that, it would have been really nice, to have some information about that sort of thing, you know. People who do travel over the hundred k’s or whatever it’s meant to be, and have to stay down, because we didn’t realise we’d have to stay down as often as we have, as far as, you know, we just thought, we’ll just go up and back but then once they explained the Lutetium, how you know, it’s really quite radioactive for 24 hours and that you know, I need to keep my space away from people. Or they may think that we should just be able to walk over to an apartment, and I sit on one couch and (name withheld) sits on the other, and you know, we keep away from, sort of, people just for that 24 hours. Yeah, you just didn’t realise what that entailed, as far as having to stay over and find accommodation, parking, all that kind of stuff. It would be nice if, and even just to travel down for the clinical visits and stuff like that, yeah just things like that, and I know when we, when we go to Geelong to my oncologist, the oncology girls give us a parking ticket so that we can validate our parking in their carpark and it’s all free.  I: That’s useful.  P: Yeah, it would be fantastic, rather than having to try and find cheap parking, you know, somewhere. So, yeah, so, that sort of, little things like that. I don’t know how that would work in your situation, as in Peter Mac, because it’s such a large hospital and it’s all dedicated to you know, there’s just so much oncology, it’s the whole hospital, it’s not just one little part of it. | | | | | | | | | | | | | |  |
|  |  |  |  |  |  |  |  |  |  |  |  |  |  |  |  |  |  |
|  |  |  |  |  |  |  |  |  |  |  |  |  |  |  |  |  |  |
|  | | |  | | | | | | | | | | | | | |  |
|  | | | | | | | | | | | | 2 |  | BV |  | 14/01/2020 12:25 PM |  |
|  | | | Yeah, that would be, that would be good, especially for country people because honestly, we had no idea about, you know, how hard it will be to park and, you know, we just worry about having to leave four hours for appointments, just in case we hit traffic. | | | | | | | | | | | | | |  |
|  | | |  | | | | | | | | | | | | | |  |
|  | | | **Codes\\needing more support** | | | | | | | | | | | | | |  |
|  |  |  |  |  | No |  | 0.1004 |  | 1 |  | | | | | | |  |
|  | | |  |  |  |  |  |  |  |  | | | | | | | |
|  | | | | | | | | | | | | 1 |  | BV |  | 14/01/2020 12:25 PM |  |
|  | | | We were talking to someone, I can’t remember who actually mentioned it to us, but they did mention, that there is someone at the hospital that can help with accommodation. Because we were talking about this to someone and they said, oh now I think the hospital has got access to, or is part of the Medicare, or something you know, to do with the travel or accommodation, that we can claim it somewhere along the line. Which, we only just got that information and because I’ve been a bit crook, we haven’t followed up on it just yet, but we got a name of someone from the hospital. Now, that sort of information would be absolutely invaluable at the start, you know, like as part of the pack they give you, the information about the Lutetium and so forth to take home and read, but if part of that, it would have been really nice, to have some information about that sort of thing, you know. People who do travel over the hundred k’s or whatever it’s meant to be, and have to stay down, because we didn’t realise we’d have to stay down as often as we have, as far as, you know, we just thought, we’ll just go up and back but then once they explained the Lutetium, how you know, it’s really quite radioactive for 24 hours and that you know, I need to keep my space away from people. Or they may think that we should just be able to walk over to an apartment, and I sit on one couch and (name withheld) sits on the other, and you know, we keep away from, sort of, people just for that 24 hours. Yeah, you just didn’t realise what that entailed, as far as having to stay over and find accommodation, parking, all that kind of stuff. It would be nice if, and even just to travel down for the clinical visits and stuff like that, yeah just things like that, and I know when we, when we go to Geelong to my oncologist, the oncology girls give us a parking ticket so that we can validate our parking in their carpark and it’s all free.  I: That’s useful.  P: Yeah, it would be fantastic, rather than having to try and find cheap parking, you know, somewhere. So, yeah, so, that sort of, little things like that. I don’t know how that would work in your situation, as in Peter Mac, because it’s such a large hospital and it’s all dedicated to you know, there’s just so much oncology, it’s the whole hospital, it’s not just one little part of it. | | | | | | | | | | | | | |  |
|  |  |  |  |  |  |  |  |  |  |  |  |  |  |  |  |  |  |
|  |  |  |  |  |  |  |  |  |  |  |  |  |  |  |  |  |  |
|  | | |  | | | | | | | | | | | | | |  |
|  | | | | | | | | | | | | | | | | | |
| Formatted Reports\\Coding Summary by File Formatted Report | | | | | | | | | | | Page 46 of 143 | | | | | | |
| 6/10/2022 8:14 AM | | | | | | | | | | | | | | | | | |
|  | | | **Classification** |  | **Aggregate** |  | **Coverage** |  | **Number Of Coding References** |  | | **Reference Number** |  | **Coded By Initials** |  | **Modified On** |  |
|  | | | **Codes\\not as intrusive as chemo** | | | | | | | | | | | | | |  |
|  |  |  |  |  | No |  | 0.0171 |  | 1 |  | | | | | | |  |
|  | | |  |  |  |  |  |  |  |  | | | | | | | |
|  | | | | | | | | | | | | 1 |  | BV |  | 15/01/2020 4:57 AM |  |
|  | | | P: Oh, I’ve been, it’s very helpful. You mean the actual Lutetium treatment itself?  I: (acknowledgement)  P: Yes… In the fact that I’m not having to do chemo. I’m not getting knocked around like I was with chemo. It’s completely different, as far as the side effects and that go. It’s so much gentler on your body. So, as far as all that goes, I’m so thankful and that’s a huge positive, you know | | | | | | | | | | | | | |  |
|  | | |  | | | | | | | | | | | | | |  |
|  | | | **Codes\\PSA numbers not going down** | | | | | | | | | | | | | |  |
|  |  |  |  |  | No |  | 0.1191 |  | 10 |  | | | | | | |  |
|  | | |  |  |  |  |  |  |  |  | | | | | | | |
|  | | | | | | | | | | | | 1 |  | BV |  | 14/01/2020 11:20 AM |  |
|  | | | But the Lutetium itself has been, well I mean, other than my numbers aren’t coming down | | | | | | | | | | | | | |  |
|  | | |  | | | | | | | | | | | | | |  |
|  | | | | | | | | | | | | 2 |  | BV |  | 15/01/2020 5:13 AM |  |
|  | | | I think I’m coping quite well with it all. I mean, the little bit of unknown that we’re going through now, and the uncertainty of why my PSA isn’t dropping, is a little bit of a concern and that causes me a little bit of worry, because you know, you always worry why isn’t it going down. But they’re saying it can, you know, stay up for a while before it goes down so, we’re hoping we’ll see a change in this blood test, coming up in a couple of weeks. | | | | | | | | | | | | | |  |
|  | | |  | | | | | | | | | | | | | |  |
|  | | | | | | | | | | | | 3 |  | BV |  | 14/01/2020 11:36 AM |  |
|  | | | I don’t know how long we’ll stay on Lutetium for because they’ve said if my markers are up, they’re going to start looking at other things, to find out why, and yeah, we’ll have one more go of it and they’ll have another look, because my cancer hasn’t reacted quite as fast as what they thought it might. | | | | | | | | | | | | | |  |
|  | | |  | | | | | | | | | | | | | |  |
|  | | | | | | | | | | | | 4 |  | BV |  | 14/01/2020 11:41 AM |  |
|  | | | They’ve just talked about trying to chase up other scans to find out why my numbers aren’t coming down. | | | | | | | | | | | | | |  |
|  | | |  | | | | | | | | | | | | | |  |
|  | | | | | | | | | | | | 5 |  | BV |  | 14/01/2020 11:41 AM |  |
|  | | | Like my PSA is still up over 30, it’s actually been going up, not down. So, they don’t know why, whether it’s a secondary type of cancer I’ve got that Lutetium’s treating, the prostate cancer, or whether, I don’t know, I don’t know. We’re not sure and that’s a little bit of the worry that comes with it all, is the uncertainty as to what’s happening. | | | | | | | | | | | | | |  |
|  | | |  | | | | | | | | | | | | | |  |
|  | | | | | | | | | | | | 6 |  | BV |  | 14/01/2020 11:45 AM |  |
|  | | | I honestly, I don’t know where, what’s happening to me. So, as far as I, I’m concerned, I’m not going backward, as far as my general day to day health and everything, I’m quite well. It’s just the numbers aren’t matching up to me. I’m a bit of an anomaly, sort of thing. | | | | | | | | | | | | | |  |
|  | | |  | | | | | | | | | | | | | |  |
|  | | | | | | | | | | | | 7 |  | BV |  | 14/01/2020 11:47 AM |  |
|  | | | You know, like you don’t even panic, you don’t even worry about much normally, you know, it’s just, it’s just yeah, just don’t know why my numbers aren’t coming down. That’s probably the only uncertainty. If they were coming down, then I’d be absolutely rapt with the whole system. | | | | | | | | | | | | | |  |
|  | | |  | | | | | | | | | | | | | |  |
|  | | | | | | | | | | | | 8 |  | BV |  | 14/01/2020 12:12 PM |  |
|  | | | Yeah, so to get back off that, and I’ve had a reduction in my pain pills as well, and I haven’t had to go back up, or anything at this stage, so you know, so, as far as all that goes, and that’s why I’m a bit of an anomaly because I’m, there’s so many things saying I’m heading in the right direction, it’s just my PSA saying I’m not, but all my other bloods and everything are really quite good. | | | | | | | | | | | | | |  |
|  | | |  | | | | | | | | | | | | | |  |
|  | | | | | | | | | | | | 9 |  | BV |  | 14/01/2020 12:13 PM |  |
|  | | | Yeah, I don’t know. It’ll be interesting to see what sort of scan they want me to have if required. I’m just hoping that my bloods finally decide to turn and start heading down and then that would just, that would make everything fit nicely then. | | | | | | | | | | | | | |  |
|  | | |  | | | | | | | | | | | | | |  |
| Formatted Reports\\Coding Summary by File Formatted Report | | | | | | | | | | | Page 47 of 143 | | | | | | |
| 6/10/2022 8:14 AM | | | | | | | | | | | | | | | | | |
|  | | | **Classification** |  | **Aggregate** |  | **Coverage** |  | **Number Of Coding References** |  | | **Reference Number** |  | **Coded By Initials** |  | **Modified On** |  |
|  | | | | | | | | | | | | | | | | | |
|  | | | | | | | | | | | | 10 |  | BV |  | 14/01/2020 12:28 PM |  |
|  | | | Yeah, yeah, I’m hoping. I’m hoping we get, you know, they did say to us that they would prefer it to happen slowly rather than quickly because that’s when you get the longevity of the treatment so, yeah, we’re still hanging in there, that everything’s going really well. | | | | | | | | | | | | | |  |
|  | | |  | | | | | | | | | | | | | |  |
|  | | | **Codes\\PSA numbers not going down\PSA numbers not going down - lutetium** | | | | | | | | | | | | | |  |
|  |  |  |  |  | No |  | 0.1187 |  | 10 |  | | | | | | |  |
|  | | |  |  |  |  |  |  |  |  | | | | | | | |
|  | | | | | | | | | | | | 1 |  | BV |  | 25/03/2020 8:20 AM |  |
|  | | | But the Lutetium itself has been, well I mean, other than my numbers aren’t coming down | | | | | | | | | | | | | |  |
|  | | |  | | | | | | | | | | | | | |  |
|  | | | | | | | | | | | | 2 |  | BV |  | 25/03/2020 8:20 AM |  |
|  | | | I think I’m coping quite well with it all. I mean, the little bit of unknown that we’re going through now, and the uncertainty of why my PSA isn’t dropping, is a little bit of a concern and that causes me a little bit of worry, because you know, you always worry why isn’t it going down. But they’re saying it can, you know, stay up for a while before it goes down so, we’re hoping we’ll see a change in this blood test, coming up in a couple of weeks. | | | | | | | | | | | | | |  |
|  | | |  | | | | | | | | | | | | | |  |
|  | | | | | | | | | | | | 3 |  | BV |  | 25/03/2020 8:20 AM |  |
|  | | | I don’t know how long we’ll stay on Lutetium for because they’ve said if my markers are up, they’re going to start looking at other things, to find out why, and yeah, we’ll have one more go of it and they’ll have another look, because my cancer hasn’t reacted quite as fast as what they thought it might. | | | | | | | | | | | | | |  |
|  | | |  | | | | | | | | | | | | | |  |
|  | | | | | | | | | | | | 4 |  | BV |  | 25/03/2020 8:20 AM |  |
|  | | | They’ve just talked about trying to chase up other scans to find out why my numbers aren’t coming down. | | | | | | | | | | | | | |  |
|  | | |  | | | | | | | | | | | | | |  |
|  | | | | | | | | | | | | 5 |  | BV |  | 25/03/2020 8:20 AM |  |
|  | | | Like my PSA is still up over 30, it’s actually been going up, not down. So, they don’t know why, whether it’s a secondary type of cancer I’ve got that Lutetium’s treating, the prostate cancer, or whether, I don’t know, I don’t know. We’re not sure and that’s a little bit of the worry that comes with it all, is the uncertainty as to what’s happening. | | | | | | | | | | | | | |  |
|  | | |  | | | | | | | | | | | | | |  |
|  | | | | | | | | | | | | 6 |  | BV |  | 25/03/2020 8:20 AM |  |
|  | | | I honestly, I don’t know where, what’s happening to me. So, as far as I, I’m concerned, I’m not going backward, as far as my general day to day health and everything, I’m quite well. It’s just the numbers aren’t matching up to me. I’m a bit of an anomaly, sort of thing. | | | | | | | | | | | | | |  |
|  | | |  | | | | | | | | | | | | | |  |
|  | | | | | | | | | | | | 7 |  | BV |  | 25/03/2020 8:20 AM |  |
|  | | | You know, like you don’t even panic, you don’t even worry about much normally, you know, it’s just, it’s just yeah, just don’t know why my numbers aren’t coming down. That’s probably the only uncertainty. If they were coming down, then I’d be absolutely rapt with the whole system. | | | | | | | | | | | | | |  |
|  | | |  | | | | | | | | | | | | | |  |
|  | | | | | | | | | | | | 8 |  | BV |  | 25/03/2020 8:20 AM |  |
|  | | | Yeah, so to get back off that, and I’ve had a reduction in my pain pills as well, and I haven’t had to go back up, or anything at this stage, so you know, so, as far as all that goes, and that’s why I’m a bit of an anomaly because I’m, there’s so many things saying I’m heading in the right direction, it’s just my PSA saying I’m not, but all my other bloods and everything are really quite good. | | | | | | | | | | | | | |  |
|  | | |  | | | | | | | | | | | | | |  |
|  | | | | | | | | | | | | 9 |  | BV |  | 25/03/2020 8:20 AM |  |
|  | | | Yeah, I don’t know. It’ll be interesting to see what sort of scan they want me to have if required. I’m just hoping that my bloods finally decide to turn and start heading down and then that would just, that would make everything fit nicely then. | | | | | | | | | | | | | |  |
|  | | |  | | | | | | | | | | | | | |  |
| Formatted Reports\\Coding Summary by File Formatted Report | | | | | | | | | | | Page 48 of 143 | | | | | | |
| 6/10/2022 8:14 AM | | | | | | | | | | | | | | | | | |
|  | | | **Classification** |  | **Aggregate** |  | **Coverage** |  | **Number Of Coding References** |  | | **Reference Number** |  | **Coded By Initials** |  | **Modified On** |  |
|  | | | | | | | | | | | | | | | | | |
|  | | | | | | | | | | | | 10 |  | BV |  | 25/03/2020 8:20 AM |  |
|  | | | Yeah, yeah, I’m hoping. I’m hoping we get, you know, they did say to us that they would prefer it to happen slowly rather than quickly because that’s when you get the longevity of the treatment so, yeah, we’re still hanging in there, that everything’s going really well. | | | | | | | | | | | | | |  |
|  | | |  | | | | | | | | | | | | | |  |
|  | | | **Codes\\quality of life changes** | | | | | | | | | | | | | |  |
|  |  |  |  |  | No |  | 0.0486 |  | 2 |  | | | | | | |  |
|  | | |  |  |  |  |  |  |  |  | | | | | | | |
|  | | | | | | | | | | | | 1 |  | BV |  | 25/03/2020 8:25 AM |  |
|  | | | P: Well, I don’t feel bad as in, yeah like, I’m functioning quite well. It’s just the fatigue. I haven’t got my bone pain, or anything like that, which is really good.  I: Good. That’s good.  P: So, I’m assuming that’s part of the Lutetium, and that’s why we took me off the steroids, because I was just ravenous and just staying up all night. So, and there was no bone pain, so, we thought just give that a go, and no bone pain came back, and I lost the, like I’m sleeping all through now, straight through the night, yeah. I’m just not starving hungry all the time, which is fantastic too. | | | | | | | | | | | | | |  |
|  | | |  | | | | | | | | | | | | | |  |
|  | | | | | | | | | | | | 2 |  | BV |  | 25/03/2020 8:25 AM |  |
|  | | | No, not really like, I’m reasonably positive you know, like I’m functioning socially and everything, I’m going really well, because the (indistinguishable) knocked me around a bit and slowed me up, as far as getting out and about, but that’s only because I’m tired, I only just want to lie on the couch and have a rest. It’s not as though I don’t want to go out, it’s just, I’m too tired to. So, but when I’m out in a social gathering, or whatever the case may be, I’m really well and everyone’s incredibly surprised at how well I’m doing. | | | | | | | | | | | | | |  |
|  | | |  | | | | | | | | | | | | | |  |
|  | | | **Codes\\quality of life changes\quality of life changes - lutetium** | | | | | | | | | | | | | |  |
|  |  |  |  |  | No |  | 0.0487 |  | 2 |  | | | | | | |  |
|  | | |  |  |  |  |  |  |  |  | | | | | | | |
|  | | | | | | | | | | | | 1 |  | BV |  | 14/01/2020 11:23 AM |  |
|  | | | P: Well, I don’t feel bad as in, yeah like, I’m functioning quite well. It’s just the fatigue. I haven’t got my bone pain, or anything like that, which is really good.  I: Good. That’s good.  P: So, I’m assuming that’s part of the Lutetium, and that’s why we took me off the steroids, because I was just ravenous and just staying up all night. So, and there was no bone pain, so, we thought just give that a go, and no bone pain came back, and I lost the, like I’m sleeping all through now, straight through the night, yeah. I’m just not starving hungry all the time, which is fantastic too. | | | | | | | | | | | | | |  |
|  | | |  | | | | | | | | | | | | | |  |
|  | | | | | | | | | | | | 2 |  | BV |  | 15/01/2020 5:29 AM |  |
|  | | | No, not really like, I’m reasonably positive you know, like I’m functioning socially and everything, I’m going really well, because the (indistinguishable) knocked me around a bit and slowed me up, as far as getting out and about, but that’s only because I’m tired, I only just want to lie on the couch and have a rest. It’s not as though I don’t want to go out, it’s just, I’m too tired to. So, but when I’m out in a social gathering, or whatever the case may be, I’m really well and everyone’s incredibly surprised at how well I’m doing. | | | | | | | | | | | | | |  |
|  | | |  | | | | | | | | | | | | | |  |
|  | | | **Codes\\quality of trialists** | | | | | | | | | | | | | |  |
|  |  |  |  |  | No |  | 0.2063 |  | 11 |  | | | | | | |  |
|  | | |  |  |  |  |  |  |  |  | | | | | | | |
|  | | | | | | | | | | | | 1 |  | BV |  | 15/01/2020 5:16 AM |  |
|  | | | I: Have you found anything else helpful?  P: Oh well, just rest. I mean, look I’ve, I must admit after you go down there, you do feel comforted and you feel relieved. Just by visiting the staff and that down there. | | | | | | | | | | | | | |  |
|  | | |  | | | | | | | | | | | | | |  |
|  | | | | | | | | | | | | 2 |  | BV |  | 14/01/2020 11:47 AM |  |
|  | | | Purely, because you know, you get a new read on what you’re doing and it mightn’t be what you’re expecting, but you know they give you the confidence to not worry, or like panic, or not worry too much you know, any more than you, sort of would normally. | | | | | | | | | | | | | |  |
|  | | |  | | | | | | | | | | | | | |  |
| Formatted Reports\\Coding Summary by File Formatted Report | | | | | | | | | | | Page 49 of 143 | | | | | | |
| 6/10/2022 8:14 AM | | | | | | | | | | | | | | | | | |
|  | | | **Classification** |  | **Aggregate** |  | **Coverage** |  | **Number Of Coding References** |  | | **Reference Number** |  | **Coded By Initials** |  | **Modified On** |  |
|  | | | | | | | | | | | | | | | | | |
|  | | | | | | | | | | | | 3 |  | BV |  | 14/01/2020 11:48 AM |  |
|  | | | You know, like you don’t even panic, you don’t even worry about much normally, you know, it’s just, it’s just yeah, just don’t know why my numbers aren’t coming down. That’s probably the only uncertainty. If they were coming down, then I’d be absolutely rapt with the whole system. | | | | | | | | | | | | | |  |
|  | | |  | | | | | | | | | | | | | |  |
|  | | | | | | | | | | | | 4 |  | BV |  | 14/01/2020 12:16 PM |  |
|  | | | There’s just been a couple of little things, probably somewhat uncontrollable and some are. My last treatment scan, treatment was fine but the next day when they take you in for scans I was booked in at 10, and the other fellow who did the treatment with me was booked in at 11, and I went in just before him, and then they left me on the bed with all the scan stuff really close to me, so, and they forgot to turn the machine on. They forgot to start the scan. So, I sat there for probably 15 – 20 minutes and I started to get quite claustrophobic. | | | | | | | | | | | | | |  |
|  | | |  | | | | | | | | | | | | | |  |
|  | | | | | | | | | | | | 5 |  | BV |  | 14/01/2020 12:16 PM |  |
|  | | | P: … Because of the heat and everything coming off, and I had my arm strapped and I was starting to sweat, and I couldn’t wipe anything and it all got quite uncomfortable, and possibly that’s why the last scans weren’t as good as they could have been, because I was quite fidgety through the scan after it, like once it started because it went for probably half an hour longer than it should have, and I’d got quite uncomfortable by that stage. So, that was a bit of a, and then of course he had his scans all done and finished then he went in and he started an hour behind me, and he was finished before I even got to go in and have my review. | | | | | | | | | | | | | |  |
|  | | |  | | | | | | | | | | | | | |  |
|  | | | | | | | | | | | | 6 |  | BV |  | 14/01/2020 12:17 PM |  |
|  | | | You sort of think, oh that’s you know, we’ve got to travel a lot further than he did, and you know, it would’ve been nice to have stayed on plan, and it was you know, just a little bit of neglect, I suppose, caused it to go off the rails. | | | | | | | | | | | | | |  |
|  | | |  | | | | | | | | | | | | | |  |
|  | | | | | | | | | | | | 7 |  | BV |  | 14/01/2020 12:17 PM |  |
|  | | | It was definitely human error. I sort of, turned around and said to them, and they said, don’t move, don’t move and I said, but it’s not going. And then they said to me afterwards, oh actually, you were right, we hadn’t pushed the start button. | | | | | | | | | | | | | |  |
|  | | |  | | | | | | | | | | | | | |  |
|  | | | | | | | | | | | | 8 |  | BV |  | 14/01/2020 12:17 PM |  |
|  | | | So, that was a bit disappointing, but you know, and the fact that I got uncomfortable then and fidgety in the scan, because they said, oh we’re not sure, there’s a couple of your spots that look bigger this time, than last time, but if you’ve moved that could possibly be the reason, and I sort of thought well, I know I was moving, and I was very uncomfortable because I’d been in there for so long. So, you know, that’s probably the downside, is that sort of thing happening, you get a bit frustrated with that, but other than that no, they’ve been, they’ve been, really good. You know, the people themselves are fantastic, it’s just you know when something like that happens and it’s just purely, it’s just, it’s just oversight, but you know, and you put up with it, because you have to, but you don’t necessarily appreciate it. | | | | | | | | | | | | | |  |
|  | | |  | | | | | | | | | | | | | |  |
|  | | | | | | | | | | | | 9 |  | BV |  | 14/01/2020 12:18 PM |  |
|  | | | Yeah, yeah like normally it wouldn’t worry me, but when you’re just laying there, and I was all strapped up for that long, and with the heat of the magnets and everything yeah, it got, it did, I started to get really hot and uncomfortable, and then of course I couldn’t settle down like I would normally do for the actual scan. I was itchy and sort of, you know, you were just sort of, just trying to scratch everywhere because you couldn’t move anyway because you’re that strapped up.  I: And that’s the last thing you want, is to be uncomfortable.  P: Yeah and that did, I just couldn’t settle myself down, to be as still as I would have liked in my scans because I wanted them to be as good, as they wanted them to be good, yeah. | | | | | | | | | | | | | |  |
|  |  |  |  |  |  |  |  |  |  |  |  |  |  |  |  |  |  |
|  | | |  | | | | | | | | | | | | | |  |
|  | | | | | | | | | | | | 10 |  | BV |  | 14/01/2020 12:18 PM |  |
|  | | | They’re really good. They’re really good. All the people you talk to are wonderful. All the staff that you encounter, I haven’t had any bad people yet. | | | | | | | | | | | | | |  |
|  | | |  | | | | | | | | | | | | | |  |
|  | | | | | | | | | | | | 11 |  | BV |  | 14/01/2020 12:19 PM |  |
|  | | | You, that’s across the board, everyone really is on the journey with you, you know, that’s what it feels like. So, as far as that goes, the empathy and compassion that they show is first class, you know, they do make you feel pretty good, and that’s why when something goes wrong, you really are taken aback, because it is so much out of the norm. So, yeah other than that little incident, no, it’s been pretty good. I had a couple of things, I think I told you about the doctor, all my results, and everything that weren’t handed in from the very start, so we’ve had couple of things, but other than that, everything, everyone’s been fantastic. | | | | | | | | | | | | | |  |
|  | | |  | | | | | | | | | | | | | |  |
|  | | | | | | | | | | | | | | | | | |
| Formatted Reports\\Coding Summary by File Formatted Report | | | | | | | | | | | Page 50 of 143 | | | | | | |
| 6/10/2022 8:14 AM | | | | | | | | | | | | | | | | | |
|  | | | **Classification** |  | **Aggregate** |  | **Coverage** |  | **Number Of Coding References** |  | | **Reference Number** |  | **Coded By Initials** |  | **Modified On** |  |
|  | | | **Codes\\side effects** | | | | | | | | | | | | | |  |
|  |  |  |  |  | No |  | 0.1212 |  | 9 |  | | | | | | |  |
|  | | |  |  |  |  |  |  |  |  | | | | | | | |
|  | | | | | | | | | | | | 1 |  | BV |  | 25/03/2020 8:26 AM |  |
|  | | | It’s been really good. Except for the last couple of weeks, but I don’t know whether that’s the Lutetium or coming off steroids. I finally got a kick back from them, I’m just not sure, I’ll find out in a couple of weeks, I suppose. | | | | | | | | | | | | | |  |
|  | | |  | | | | | | | | | | | | | |  |
|  | | | | | | | | | | | | 2 |  | BV |  | 25/03/2020 8:26 AM |  |
|  | | | But the Lutetium itself has been, well I mean, other than my numbers aren’t coming down, no side effects or anything, which is really good. | | | | | | | | | | | | | |  |
|  | | |  | | | | | | | | | | | | | |  |
|  | | | | | | | | | | | | 3 |  | BV |  | 25/03/2020 8:26 AM |  |
|  | | | But the last couple of weeks I’ve been very fatigued and nauseous but we just don’t know, like I came off my Dexamethasone so, I’m not sure whether it’s just a delayed, sort of, a hit of fatigue, which is normal, you get when you come off it, and I sort of, didn’t get it when I came off it straight away. Also, my lack of appetite, might be, well, it seems to be the cause of the nausea, as far as I can tell, because if I eat something, the nausea goes away. | | | | | | | | | | | | | |  |
|  | | |  | | | | | | | | | | | | | |  |
|  | | | | | | | | | | | | 4 |  | BV |  | 25/03/2020 8:26 AM |  |
|  | | | Yeah, and because I’m off the steroid with my, because I had a huge appetite, and I reckon my body’s just got used to, used to having heaps of food go in to it, and then when I stopped and the hunger stopped, I’m just not eating anywhere near as much as I used too. | | | | | | | | | | | | | |  |
|  | | |  | | | | | | | | | | | | | |  |
|  | | | | | | | | | | | | 5 |  | BV |  | 25/03/2020 8:26 AM |  |
|  | | | P: Well, I don’t feel bad as in, yeah like, I’m functioning quite well. It’s just the fatigue. I haven’t got my bone pain, or anything like that, which is really good.  I: Good. That’s good.  P: So, I’m assuming that’s part of the Lutetium, and that’s why we took me off the steroids, because I was just ravenous and just staying up all night. So, and there was no bone pain, so, we thought just give that a go, and no bone pain came back, and I lost the, like I’m sleeping all through now, straight through the night, yeah. I’m just not starving hungry all the time, which is fantastic too. | | | | | | | | | | | | | |  |
|  | | |  | | | | | | | | | | | | | |  |
|  | | | | | | | | | | | | 6 |  | BV |  | 25/03/2020 8:26 AM |  |
|  | | | I: Have you found anything else helpful?  P: Oh well, just rest. I mean, look I’ve, I must admit after you go down there, you do feel comforted and you feel relieved. Just by visiting the staff and that down there. | | | | | | | | | | | | | |  |
|  | | |  | | | | | | | | | | | | | |  |
|  | | | | | | | | | | | | 7 |  | BV |  | 25/03/2020 8:26 AM |  |
|  | | | No, not really like, I’m reasonably positive you know, like I’m functioning socially and everything, I’m going really well, because the (indistinguishable) knocked me around a bit and slowed me up, as far as getting out and about, but that’s only because I’m tired, I only just want to lie on the couch and have a rest. It’s not as though I don’t want to go out, it’s just, I’m too tired to. So, but when I’m out in a social gathering, or whatever the case may be, I’m really well and everyone’s incredibly surprised at how well I’m doing. | | | | | | | | | | | | | |  |
|  | | |  | | | | | | | | | | | | | |  |
|  | | | | | | | | | | | | 8 |  | BV |  | 25/03/2020 8:26 AM |  |
|  | | | Oh… yeah. It’s brilliant, that is fantastic because when we first got to you guys, I did have a lot of bone pain. It was quite significant and that’s why they put me back on the Dexamethasone, but I’ve never been on it everyday, like I was. | | | | | | | | | | | | | |  |
|  | | |  | | | | | | | | | | | | | |  |
|  | | | | | | | | | | | | 9 |  | BV |  | 25/03/2020 8:26 AM |  |
|  | | | Yeah, so to get back off that, and I’ve had a reduction in my pain pills as well, and I haven’t had to go back up, or anything at this stage | | | | | | | | | | | | | |  |
|  | | |  | | | | | | | | | | | | | |  |
|  | | | | | | | | | | | | | | | | | |
| Formatted Reports\\Coding Summary by File Formatted Report | | | | | | | | | | | Page 51 of 143 | | | | | | |
| 6/10/2022 8:14 AM | | | | | | | | | | | | | | | | | |
|  | | | **Classification** |  | **Aggregate** |  | **Coverage** |  | **Number Of Coding References** |  | | **Reference Number** |  | **Coded By Initials** |  | **Modified On** |  |
|  | | | **Codes\\side effects\side effects - lutetium** | | | | | | | | | | | | | |  |
|  |  |  |  |  | No |  | 0.1216 |  | 9 |  | | | | | | |  |
|  | | |  |  |  |  |  |  |  |  | | | | | | | |
|  | | | | | | | | | | | | 1 |  | BV |  | 14/01/2020 11:20 AM |  |
|  | | | It’s been really good. Except for the last couple of weeks, but I don’t know whether that’s the Lutetium or coming off steroids. I finally got a kick back from them, I’m just not sure, I’ll find out in a couple of weeks, I suppose. | | | | | | | | | | | | | |  |
|  | | |  | | | | | | | | | | | | | |  |
|  | | | | | | | | | | | | 2 |  | BV |  | 14/01/2020 11:20 AM |  |
|  | | | But the Lutetium itself has been, well I mean, other than my numbers aren’t coming down, no side effects or anything, which is really good. | | | | | | | | | | | | | |  |
|  | | |  | | | | | | | | | | | | | |  |
|  | | | | | | | | | | | | 3 |  | BV |  | 14/01/2020 11:21 AM |  |
|  | | | But the last couple of weeks I’ve been very fatigued and nauseous but we just don’t know, like I came off my Dexamethasone so, I’m not sure whether it’s just a delayed, sort of, a hit of fatigue, which is normal, you get when you come off it, and I sort of, didn’t get it when I came off it straight away. Also, my lack of appetite, might be, well, it seems to be the cause of the nausea, as far as I can tell, because if I eat something, the nausea goes away. | | | | | | | | | | | | | |  |
|  | | |  | | | | | | | | | | | | | |  |
|  | | | | | | | | | | | | 4 |  | BV |  | 14/01/2020 11:21 AM |  |
|  | | | Yeah, and because I’m off the steroid with my, because I had a huge appetite, and I reckon my body’s just got used to, used to having heaps of food go in to it, and then when I stopped and the hunger stopped, I’m just not eating anywhere near as much as I used too. | | | | | | | | | | | | | |  |
|  | | |  | | | | | | | | | | | | | |  |
|  | | | | | | | | | | | | 5 |  | BV |  | 15/01/2020 5:25 AM |  |
|  | | | P: Well, I don’t feel bad as in, yeah like, I’m functioning quite well. It’s just the fatigue. I haven’t got my bone pain, or anything like that, which is really good.  I: Good. That’s good.  P: So, I’m assuming that’s part of the Lutetium, and that’s why we took me off the steroids, because I was just ravenous and just staying up all night. So, and there was no bone pain, so, we thought just give that a go, and no bone pain came back, and I lost the, like I’m sleeping all through now, straight through the night, yeah. I’m just not starving hungry all the time, which is fantastic too. | | | | | | | | | | | | | |  |
|  | | |  | | | | | | | | | | | | | |  |
|  | | | | | | | | | | | | 6 |  | BV |  | 14/01/2020 11:46 AM |  |
|  | | | I: Have you found anything else helpful?  P: Oh well, just rest. I mean, look I’ve, I must admit after you go down there, you do feel comforted and you feel relieved. Just by visiting the staff and that down there. | | | | | | | | | | | | | |  |
|  | | |  | | | | | | | | | | | | | |  |
|  | | | | | | | | | | | | 7 |  | BV |  | 14/01/2020 11:49 AM |  |
|  | | | No, not really like, I’m reasonably positive you know, like I’m functioning socially and everything, I’m going really well, because the (indistinguishable) knocked me around a bit and slowed me up, as far as getting out and about, but that’s only because I’m tired, I only just want to lie on the couch and have a rest. It’s not as though I don’t want to go out, it’s just, I’m too tired to. So, but when I’m out in a social gathering, or whatever the case may be, I’m really well and everyone’s incredibly surprised at how well I’m doing. | | | | | | | | | | | | | |  |
|  | | |  | | | | | | | | | | | | | |  |
|  | | | | | | | | | | | | 8 |  | BV |  | 15/01/2020 5:25 AM |  |
|  | | | Oh… yeah. It’s brilliant, that is fantastic because when we first got to you guys, I did have a lot of bone pain. It was quite significant and that’s why they put me back on the Dexamethasone, but I’ve never been on it everyday, like I was. | | | | | | | | | | | | | |  |
|  | | |  | | | | | | | | | | | | | |  |
|  | | | | | | | | | | | | 9 |  | BV |  | 15/01/2020 5:25 AM |  |
|  | | | Yeah, so to get back off that, and I’ve had a reduction in my pain pills as well, and I haven’t had to go back up, or anything at this stage | | | | | | | | | | | | | |  |
|  | | |  | | | | | | | | | | | | | |  |
|  | | | | | | | | | | | | | | | | | |
| Formatted Reports\\Coding Summary by File Formatted Report | | | | | | | | | | | Page 52 of 143 | | | | | | |
| 6/10/2022 8:14 AM | | | | | | | | | | | | | | | | | |
|  | | | **Classification** |  | **Aggregate** |  | **Coverage** |  | **Number Of Coding References** |  | | **Reference Number** |  | **Coded By Initials** |  | **Modified On** |  |
|  | | | **Codes\\side effects\side effects - lutetium\reduction in pain** | | | | | | | | | | | | | |  |
|  |  |  |  |  | No |  | 0.0419 |  | 3 |  | | | | | | |  |
|  | | |  |  |  |  |  |  |  |  | | | | | | | |
|  | | | | | | | | | | | | 1 |  | BV |  | 14/01/2020 11:23 AM |  |
|  | | | P: Well, I don’t feel bad as in, yeah like, I’m functioning quite well. It’s just the fatigue. I haven’t got my bone pain, or anything like that, which is really good.  I: Good. That’s good.  P: So, I’m assuming that’s part of the Lutetium, and that’s why we took me off the steroids, because I was just ravenous and just staying up all night. So, and there was no bone pain, so, we thought just give that a go, and no bone pain came back, and I lost the, like I’m sleeping all through now, straight through the night, yeah. I’m just not starving hungry all the time, which is fantastic too. | | | | | | | | | | | | | |  |
|  | | |  | | | | | | | | | | | | | |  |
|  | | | | | | | | | | | | 2 |  | BV |  | 14/01/2020 11:49 AM |  |
|  | | | Oh… yeah. It’s brilliant, that is fantastic because when we first got to you guys, I did have a lot of bone pain. It was quite significant and that’s why they put me back on the Dexamethasone, but I’ve never been on it everyday, like I was. | | | | | | | | | | | | | |  |
|  | | |  | | | | | | | | | | | | | |  |
|  | | | | | | | | | | | | 3 |  | BV |  | 14/01/2020 12:13 PM |  |
|  | | | Yeah, so to get back off that, and I’ve had a reduction in my pain pills as well, and I haven’t had to go back up, or anything at this stage | | | | | | | | | | | | | |  |
|  | | |  | | | | | | | | | | | | | |  |
|  | | | **Codes\\staying positive** | | | | | | | | | | | | | |  |
|  |  |  |  |  | No |  | 0.0290 |  | 3 |  | | | | | | |  |
|  | | |  |  |  |  |  |  |  |  | | | | | | | |
|  | | | | | | | | | | | | 1 |  | BV |  | 14/01/2020 11:36 AM |  |
|  | | | In the fact that I’m not having to do chemo. I’m not getting knocked around like I was with chemo. It’s completely different, as far as the side effects and that go. It’s so much gentler on your body. So, as far as all that goes, I’m so thankful and that’s a huge positive, you know | | | | | | | | | | | | | |  |
|  | | |  | | | | | | | | | | | | | |  |
|  | | | | | | | | | | | | 2 |  | BV |  | 15/01/2020 4:49 AM |  |
|  | | | I honestly, I don’t know where, what’s happening to me. So, as far as I, I’m concerned, I’m not going backward, as far as my general day to day health and everything, I’m quite well. It’s just the numbers aren’t matching up to me. I’m a bit of an anomaly, sort of thing. | | | | | | | | | | | | | |  |
|  | | |  | | | | | | | | | | | | | |  |
|  | | | | | | | | | | | | 3 |  | BV |  | 14/01/2020 11:49 AM |  |
|  | | | No, not really like, I’m reasonably positive you know, like I’m functioning socially and everything, I’m going really well | | | | | | | | | | | | | |  |
|  | | |  | | | | | | | | | | | | | |  |
|  | | | **Codes\\struggling with treatment** | | | | | | | | | | | | | |  |
|  |  |  |  |  | No |  | 0.1526 |  | 6 |  | | | | | | |  |
|  | | |  |  |  |  |  |  |  |  | | | | | | | |
|  | | | | | | | | | | | | 1 |  | BV |  | 14/01/2020 11:49 AM |  |
|  | | | No, not really like, I’m reasonably positive you know, like I’m functioning socially and everything, I’m going really well, because the (indistinguishable) knocked me around a bit and slowed me up, as far as getting out and about, but that’s only because I’m tired, I only just want to lie on the couch and have a rest. It’s not as though I don’t want to go out, it’s just, I’m too tired to. So, but when I’m out in a social gathering, or whatever the case may be, I’m really well and everyone’s incredibly surprised at how well I’m doing. | | | | | | | | | | | | | |  |
|  | | |  | | | | | | | | | | | | | |  |
|  | | | | | | | | | | | | 2 |  | BV |  | 14/01/2020 12:16 PM |  |
|  | | | There’s just been a couple of little things, probably somewhat uncontrollable and some are. My last treatment scan, treatment was fine but the next day when they take you in for scans I was booked in at 10, and the other fellow who did the treatment with me was booked in at 11, and I went in just before him, and then they left me on the bed with all the scan stuff really close to me, so, and they forgot to turn the machine on. They forgot to start the scan. So, I sat there for probably 15 – 20 minutes and I started to get quite claustrophobic. | | | | | | | | | | | | | |  |
|  | | |  | | | | | | | | | | | | | |  |
| Formatted Reports\\Coding Summary by File Formatted Report | | | | | | | | | | | Page 53 of 143 | | | | | | |
| 6/10/2022 8:14 AM | | | | | | | | | | | | | | | | | |
|  | | | **Classification** |  | **Aggregate** |  | **Coverage** |  | **Number Of Coding References** |  | | **Reference Number** |  | **Coded By Initials** |  | **Modified On** |  |
|  | | | | | | | | | | | | | | | | | |
|  | | | | | | | | | | | | 3 |  | BV |  | 14/01/2020 12:16 PM |  |
|  | | | P: … Because of the heat and everything coming off, and I had my arm strapped and I was starting to sweat, and I couldn’t wipe anything and it all got quite uncomfortable, and possibly that’s why the last scans weren’t as good as they could have been, because I was quite fidgety through the scan after it, like once it started because it went for probably half an hour longer than it should have, and I’d got quite uncomfortable by that stage. So, that was a bit of a, and then of course he had his scans all done and finished then he went in and he started an hour behind me, and he was finished before I even got to go in and have my review. | | | | | | | | | | | | | |  |
|  | | |  | | | | | | | | | | | | | |  |
|  | | | | | | | | | | | | 4 |  | BV |  | 14/01/2020 12:17 PM |  |
|  | | | It was definitely human error. I sort of, turned around and said to them, and they said, don’t move, don’t move and I said, but it’s not going. And then they said to me afterwards, oh actually, you were right, we hadn’t pushed the start button. | | | | | | | | | | | | | |  |
|  | | |  | | | | | | | | | | | | | |  |
|  | | | | | | | | | | | | 5 |  | BV |  | 14/01/2020 12:17 PM |  |
|  | | | So, that was a bit disappointing, but you know, and the fact that I got uncomfortable then and fidgety in the scan, because they said, oh we’re not sure, there’s a couple of your spots that look bigger this time, than last time, but if you’ve moved that could possibly be the reason, and I sort of thought well, I know I was moving, and I was very uncomfortable because I’d been in there for so long. So, you know, that’s probably the downside, is that sort of thing happening, you get a bit frustrated with that, but other than that no, they’ve been, they’ve been, really good. You know, the people themselves are fantastic, it’s just you know when something like that happens and it’s just purely, it’s just, it’s just oversight, but you know, and you put up with it, because you have to, but you don’t necessarily appreciate it. | | | | | | | | | | | | | |  |
|  | | |  | | | | | | | | | | | | | |  |
|  | | | | | | | | | | | | 6 |  | BV |  | 14/01/2020 12:18 PM |  |
|  | | | Yeah, yeah like normally it wouldn’t worry me, but when you’re just laying there, and I was all strapped up for that long, and with the heat of the magnets and everything yeah, it got, it did, I started to get really hot and uncomfortable, and then of course I couldn’t settle down like I would normally do for the actual scan. I was itchy and sort of, you know, you were just sort of, just trying to scratch everywhere because you couldn’t move anyway because you’re that strapped up.  I: And that’s the last thing you want, is to be uncomfortable.  P: Yeah and that did, I just couldn’t settle myself down, to be as still as I would have liked in my scans because I wanted them to be as good, as they wanted them to be good, yeah. | | | | | | | | | | | | | |  |
|  |  |  |  |  |  |  |  |  |  |  |  |  |  |  |  |  |  |
|  | | |  | | | | | | | | | | | | | |  |
|  | | | **Codes\\struggling with treatment\struggling with post treatment scans** | | | | | | | | | | | | | |  |
|  |  |  |  |  | No |  | 0.1293 |  | 5 |  | | | | | | |  |
|  | | |  |  |  |  |  |  |  |  | | | | | | | |
|  | | | | | | | | | | | | 1 |  | BV |  | 15/01/2020 5:29 AM |  |
|  | | | There’s just been a couple of little things, probably somewhat uncontrollable and some are. My last treatment scan, treatment was fine but the next day when they take you in for scans I was booked in at 10, and the other fellow who did the treatment with me was booked in at 11, and I went in just before him, and then they left me on the bed with all the scan stuff really close to me, so, and they forgot to turn the machine on. They forgot to start the scan. So, I sat there for probably 15 – 20 minutes and I started to get quite claustrophobic. | | | | | | | | | | | | | |  |
|  | | |  | | | | | | | | | | | | | |  |
|  | | | | | | | | | | | | 2 |  | BV |  | 15/01/2020 5:29 AM |  |
|  | | | P: … Because of the heat and everything coming off, and I had my arm strapped and I was starting to sweat, and I couldn’t wipe anything and it all got quite uncomfortable, and possibly that’s why the last scans weren’t as good as they could have been, because I was quite fidgety through the scan after it, like once it started because it went for probably half an hour longer than it should have, and I’d got quite uncomfortable by that stage. So, that was a bit of a, and then of course he had his scans all done and finished then he went in and he started an hour behind me, and he was finished before I even got to go in and have my review. | | | | | | | | | | | | | |  |
|  | | |  | | | | | | | | | | | | | |  |
|  | | | | | | | | | | | | 3 |  | BV |  | 15/01/2020 5:30 AM |  |
|  | | | It was definitely human error. I sort of, turned around and said to them, and they said, don’t move, don’t move and I said, but it’s not going. And then they said to me afterwards, oh actually, you were right, we hadn’t pushed the start button. | | | | | | | | | | | | | |  |
|  | | |  | | | | | | | | | | | | | |  |
|  | | | | | | | | | | | | 4 |  | BV |  | 15/01/2020 5:30 AM |  |
|  | | | So, that was a bit disappointing, but you know, and the fact that I got uncomfortable then and fidgety in the scan, because they said, oh we’re not sure, there’s a couple of your spots that look bigger this time, than last time, but if you’ve moved that could possibly be the reason, and I sort of thought well, I know I was moving, and I was very uncomfortable because I’d been in there for so long. So, you know, that’s probably the downside, is that sort of thing happening, you get a bit frustrated with that, but other than that no, they’ve been, they’ve been, really good. You know, the people themselves are fantastic, it’s just you know when something like that happens and it’s just purely, it’s just, it’s just oversight, but you know, and you put up with it, because you have to, but you don’t necessarily appreciate it. | | | | | | | | | | | | | |  |
|  | | |  | | | | | | | | | | | | | |  |
| Formatted Reports\\Coding Summary by File Formatted Report | | | | | | | | | | | Page 54 of 143 | | | | | | |
| 6/10/2022 8:14 AM | | | | | | | | | | | | | | | | | |
|  | | | **Classification** |  | **Aggregate** |  | **Coverage** |  | **Number Of Coding References** |  | | **Reference Number** |  | **Coded By Initials** |  | **Modified On** |  |
|  | | | | | | | | | | | | | | | | | |
|  | | | | | | | | | | | | 5 |  | BV |  | 15/01/2020 5:30 AM |  |
|  | | | Yeah, yeah like normally it wouldn’t worry me, but when you’re just laying there, and I was all strapped up for that long, and with the heat of the magnets and everything yeah, it got, it did, I started to get really hot and uncomfortable, and then of course I couldn’t settle down like I would normally do for the actual scan. I was itchy and sort of, you know, you were just sort of, just trying to scratch everywhere because you couldn’t move anyway because you’re that strapped up.  I: And that’s the last thing you want, is to be uncomfortable.  P: Yeah and that did, I just couldn’t settle myself down, to be as still as I would have liked in my scans because I wanted them to be as good, as they wanted them to be good, yeah. | | | | | | | | | | | | | |  |
|  |  |  |  |  |  |  |  |  |  |  |  |  |  |  |  |  |  |
|  | | |  | | | | | | | | | | | | | |  |
|  | | | **Codes\\struggling with treatment\symptom fluctuations** | | | | | | | | | | | | | |  |
|  |  |  |  |  | No |  | 0.0123 |  | 1 |  | | | | | | |  |
|  | | |  |  |  |  |  |  |  |  | | | | | | | |
|  | | | | | | | | | | | | 1 |  | BV |  | 14/01/2020 11:25 AM |  |
|  | | | I think I’m coping quite well with it all. I mean, the little bit of unknown that we’re going through now, and the uncertainty of why my PSA isn’t dropping, is a little bit of a concern and that causes me a little bit of worry, because you know, you always worry why isn’t it going down. | | | | | | | | | | | | | |  |
|  | | |  | | | | | | | | | | | | | |  |
|  | | | **Codes\\thankful for others sacrifice** | | | | | | | | | | | | | |  |
|  |  |  |  |  | No |  | 0.0422 |  | 3 |  | | | | | | |  |
|  | | |  |  |  |  |  |  |  |  | | | | | | | |
|  | | | | | | | | | | | | 1 |  | BV |  | 15/01/2020 5:00 AM |  |
|  | | | I’m so thankful that people have done this you know, 10 years ago or whatever, to give all the drugs I’ve had up to this point, you know, someone would have done a trial somewhere along the way, for those drugs to be available to me and they are actually, a godsend. Every time you have a treatment, you just thank your lucky stars, that you’ve got this available, even if it does make you crook and I’m just so thankful that other people have paved the way for me to have this treatment | | | | | | | | | | | | | |  |
|  | | |  | | | | | | | | | | | | | |  |
|  | | | | | | | | | | | | 2 |  | BV |  | 14/01/2020 12:20 PM |  |
|  | | | that’s all I want to do, is pay it forward or pay it back, whatever you call it, because I want the next lot of people to, you know, to be thankful, and I’m sure they will be because of their situation, they’ll be as thankful that someone like me, has done what I’ve done, you know. And it’s not about any, purely, you know you’re in the line and you need to step up and do what everyone else has done, so you know, if you feel you can. | | | | | | | | | | | | | |  |
|  | | |  | | | | | | | | | | | | | |  |
|  | | | | | | | | | | | | 3 |  | BV |  | 14/01/2020 12:22 PM |  |
|  | | | Oh no, you’ve got to thank a lot more people than just me. | | | | | | | | | | | | | |  |
|  | | |  | | | | | | | | | | | | | |  |
|  | | | **Codes\\travelling to treatment** | | | | | | | | | | | | | |  |
|  |  |  |  |  | No |  | 0.0163 |  | 2 |  | | | | | | |  |
|  | | |  |  |  |  |  |  |  |  | | | | | | | |
|  | | | | | | | | | | | | 1 |  | BV |  | 14/01/2020 11:27 AM |  |
|  | | | I: Are you travelling down yourself, to the treatments?  P: Yes. Yes, well yeah (name withheld) and I. My wife and I. | | | | | | | | | | | | | |  |
|  | | |  | | | | | | | | | | | | | |  |
|  | | | | | | | | | | | | 2 |  | BV |  | 14/01/2020 11:31 AM |  |
|  | | | I mean, we have to stay over the night because I’m radioactive, you know, it’s probably just a, and it’s easier to stay, then have to go home and then get up first thing in the morning and then come back down and deal with the traffic and all that sort of stuff. | | | | | | | | | | | | | |  |
|  | | |  | | | | | | | | | | | | | |  |
| Formatted Reports\\Coding Summary by File Formatted Report | | | | | | | | | | | Page 55 of 143 | | | | | | |
| 6/10/2022 8:14 AM | | | | | | | | | | | | | | | | | |
|  | | | **Classification** |  | **Aggregate** |  | **Coverage** |  | **Number Of Coding References** |  | | **Reference Number** |  | **Coded By Initials** |  | **Modified On** |  |
|  | | | **Codes\\travelling to treatment\travelling to treatment challenging** | | | | | | | | | | | | | |  |
|  |  |  |  |  | No |  | 0.1815 |  | 8 |  | | | | | | |  |
|  | | |  |  |  |  |  |  |  |  | | | | | | | |
|  | | | | | | | | | | | | 1 |  | BV |  | 14/01/2020 11:27 AM |  |
|  | | | So, we’re having to dip into my life insurance, just to cover day to day costs, which we were hoping we wouldn’t have to do. And then of course, that means extra trips down to Melbourne and staying over, and all that sort of stuff all adds to the financial pressures, for sure. But I mean, we just have to do it, you know? If I want to be here, we have to take the hit somewhere. | | | | | | | | | | | | | |  |
|  | | |  | | | | | | | | | | | | | |  |
|  | | | | | | | | | | | | 2 |  | BV |  | 15/01/2020 4:43 AM |  |
|  | | | I mean, we have to stay over the night because I’m radioactive, you know, it’s probably just a, and it’s easier to stay, then have to go home and then get up first thing in the morning and then come back down and deal with the traffic and all that sort of stuff. | | | | | | | | | | | | | |  |
|  | | |  | | | | | | | | | | | | | |  |
|  | | | | | | | | | | | | 3 |  | BV |  | 14/01/2020 11:32 AM |  |
|  | | | That, well, that and just the fuel and everything you know, and getting you know, you just putting the k’s on the car and you get the car serviced and yeah, you just notice it a little, but I mean that’s what it is you know, we’re used to travelling anyway because we live out of a big city so, if you need to do something, or want to go see something, most of the time you have to pack up and go to Melbourne anyway. So, it’s not uncommon to us, but just because I’m not working, you just can’t, it’s getting a bit harder, to cover all these costs. | | | | | | | | | | | | | |  |
|  | | |  | | | | | | | | | | | | | |  |
|  | | | | | | | | | | | | 4 |  | BV |  | 14/01/2020 12:25 PM |  |
|  | | | We were talking to someone, I can’t remember who actually mentioned it to us, but they did mention, that there is someone at the hospital that can help with accommodation. Because we were talking about this to someone and they said, oh now I think the hospital has got access to, or is part of the Medicare, or something you know, to do with the travel or accommodation, that we can claim it somewhere along the line. Which, we only just got that information and because I’ve been a bit crook, we haven’t followed up on it just yet, but we got a name of someone from the hospital. Now, that sort of information would be absolutely invaluable at the start, you know, like as part of the pack they give you, the information about the Lutetium and so forth to take home and read, but if part of that, it would have been really nice, to have some information about that sort of thing, you know. People who do travel over the hundred k’s or whatever it’s meant to be, and have to stay down, because we didn’t realise we’d have to stay down as often as we have, as far as, you know, we just thought, we’ll just go up and back but then once they explained the Lutetium, how you know, it’s really quite radioactive for 24 hours and that you know, I need to keep my space away from people. Or they may think that we should just be able to walk over to an apartment, and I sit on one couch and (name withheld) sits on the other, and you know, we keep away from, sort of, people just for that 24 hours. Yeah, you just didn’t realise what that entailed, as far as having to stay over and find accommodation, parking, all that kind of stuff. It would be nice if, and even just to travel down for the clinical visits and stuff like that, yeah just things like that, and I know when we, when we go to Geelong to my oncologist, the oncology girls give us a parking ticket so that we can validate our parking in their carpark and it’s all free.  I: That’s useful.  P: Yeah, it would be fantastic, rather than having to try and find cheap parking, you know, somewhere. So, yeah, so, that sort of, little things like that. I don’t know how that would work in your situation, as in Peter Mac, because it’s such a large hospital and it’s all dedicated to you know, there’s just so much oncology, it’s the whole hospital, it’s not just one little part of it. | | | | | | | | | | | | | |  |
|  |  |  |  |  |  |  |  |  |  |  |  |  |  |  |  |  |  |
|  |  |  |  |  |  |  |  |  |  |  |  |  |  |  |  |  |  |
|  | | |  | | | | | | | | | | | | | |  |
|  | | | | | | | | | | | | 5 |  | BV |  | 14/01/2020 12:25 PM |  |
|  | | | Yeah, that would be, that would be good, especially for country people because honestly, we had no idea about, you know, how hard it will be to park and, you know, we just worry about having to leave four hours for appointments, just in case we hit traffic. | | | | | | | | | | | | | |  |
|  | | |  | | | | | | | | | | | | | |  |
|  | | | | | | | | | | | | 6 |  | BV |  | 14/01/2020 12:26 PM |  |
|  | | | And that’s why, if the scan’s early, which it normally is, you don’t want to have to ring them and say, oh I’m sorry we’re caught in traffic. I used to end up getting there at 6 in the morning and sit around until 9 o’clock for your appointment. | | | | | | | | | | | | | |  |
|  | | |  | | | | | | | | | | | | | |  |
|  | | | | | | | | | | | | 7 |  | BV |  | 14/01/2020 12:26 PM |  |
|  | | | That’s just what we do, you know, we just think it’s going to be a horrible traffic day, every day and we end up going four hours before we should. | | | | | | | | | | | | | |  |
|  | | |  | | | | | | | | | | | | | |  |
|  | | | | | | | | | | | | 8 |  | BV |  | 14/01/2020 12:26 PM |  |
|  | | | We tend to be too early though, you know. | | | | | | | | | | | | | |  |
|  | | |  | | | | | | | | | | | | | |  |
| Formatted Reports\\Coding Summary by File Formatted Report | | | | | | | | | | | Page 56 of 143 | | | | | | |
| 6/10/2022 8:14 AM | | | | | | | | | | | | | | | | | |
|  | | | **Classification** |  | **Aggregate** |  | **Coverage** |  | **Number Of Coding References** |  | | **Reference Number** |  | **Coded By Initials** |  | **Modified On** |  |
|  | | | **Codes\\treatments been good** | | | | | | | | | | | | | |  |
|  |  |  |  |  | No |  | 0.0009 |  | 1 |  | | | | | | |  |
|  | | |  |  |  |  |  |  |  |  | | | | | | | |
|  | | | | | | | | | | | | 1 |  | BV |  | 14/01/2020 11:20 AM |  |
|  | | | It’s been really good. | | | | | | | | | | | | | |  |
|  | | |  | | | | | | | | | | | | | |  |
|  | | | **Codes\\treatments left** | | | | | | | | | | | | | |  |
|  |  |  |  |  | No |  | 0.0181 |  | 2 |  | | | | | | |  |
|  | | |  |  |  |  |  |  |  |  | | | | | | | |
|  | | | | | | | | | | | | 1 |  | BV |  | 14/01/2020 11:22 AM |  |
|  | | | I think, I’ve had 2 and I’m nearly due for my third. I’ve got my third in a couple of weeks. Oh, actually, two weeks today. | | | | | | | | | | | | | |  |
|  | | |  | | | | | | | | | | | | | |  |
|  | | | | | | | | | | | | 2 |  | BV |  | 14/01/2020 11:41 AM |  |
|  | | | That’s correct yeah, yeah like this half way through, and then yeah, you know, I suppose as long as they’re happy with the decrease in activity of the cancers, then I’ll continue on but I know I was in with another fellow and he’s nearly done, completely clear and I’m still quite significant. | | | | | | | | | | | | | |  |
|  | | |  | | | | | | | | | | | | | |  |
|  | | | **Codes\\uncertainty of the future** | | | | | | | | | | | | | |  |
|  |  |  |  |  | No |  | 0.0785 |  | 6 |  | | | | | | |  |
|  | | |  |  |  |  |  |  |  |  | | | | | | | |
|  | | | | | | | | | | | | 1 |  | BV |  | 15/01/2020 5:32 AM |  |
|  | | | I think I’m coping quite well with it all. I mean, the little bit of unknown that we’re going through now, and the uncertainty of why my PSA isn’t dropping, is a little bit of a concern and that causes me a little bit of worry, because you know, you always worry why isn’t it going down. But they’re saying it can, you know, stay up for a while before it goes down so, we’re hoping we’ll see a change in this blood test, coming up in a couple of weeks. | | | | | | | | | | | | | |  |
|  | | |  | | | | | | | | | | | | | |  |
|  | | | | | | | | | | | | 2 |  | BV |  | 14/01/2020 11:36 AM |  |
|  | | | I don’t know how long we’ll stay on Lutetium for because they’ve said if my markers are up, they’re going to start looking at other things, to find out why, and yeah, we’ll have one more go of it and they’ll have another look, because my cancer hasn’t reacted quite as fast as what they thought it might. | | | | | | | | | | | | | |  |
|  | | |  | | | | | | | | | | | | | |  |
|  | | | | | | | | | | | | 3 |  | BV |  | 14/01/2020 11:40 AM |  |
|  | | | That’s correct yeah, yeah like this half way through, and then yeah, you know, I suppose as long as they’re happy with the decrease in activity of the cancers, then I’ll continue on but I know I was in with another fellow and he’s nearly done, completely clear and I’m still quite significant. | | | | | | | | | | | | | |  |
|  | | |  | | | | | | | | | | | | | |  |
|  | | | | | | | | | | | | 4 |  | BV |  | 14/01/2020 11:41 AM |  |
|  | | | Like my PSA is still up over 30, it’s actually been going up, not down. So, they don’t know why, whether it’s a secondary type of cancer I’ve got that Lutetium’s treating, the prostate cancer, or whether, I don’t know, I don’t know. We’re not sure and that’s a little bit of the worry that comes with it all, is the uncertainty as to what’s happening. | | | | | | | | | | | | | |  |
|  | | |  | | | | | | | | | | | | | |  |
|  | | | | | | | | | | | | 5 |  | BV |  | 14/01/2020 11:45 AM |  |
|  | | | Oh yeah. It’s just, it’s purely… They’ve got to have time to formulate some sort of a read on me, and even I can’t get one on me and I’m pretty good. | | | | | | | | | | | | | |  |
|  | | |  | | | | | | | | | | | | | |  |
|  | | | | | | | | | | | | | | | | | |
| Formatted Reports\\Coding Summary by File Formatted Report | | | | | | | | | | | Page 57 of 143 | | | | | | |
| 6/10/2022 8:14 AM | | | | | | | | | | | | | | | | | |
|  | | | **Classification** |  | **Aggregate** |  | **Coverage** |  | **Number Of Coding References** |  | | **Reference Number** |  | **Coded By Initials** |  | **Modified On** |  |
|  | | | | | | | | | | | | | | | | | |
|  | | | | | | | | | | | | 6 |  | BV |  | 15/01/2020 4:49 AM |  |
|  | | | I honestly, I don’t know where, what’s happening to me. So, as far as I, I’m concerned, I’m not going backward, as far as my general day to day health and everything, I’m quite well. It’s just the numbers aren’t matching up to me. I’m a bit of an anomaly, sort of thing. | | | | | | | | | | | | | |  |
|  | | |  | | | | | | | | | | | | | |  |
|  | | | **Codes\\unsure of how long treatment effects will last** | | | | | | | | | | | | | |  |
|  |  |  |  |  | No |  | 0.0130 |  | 1 |  | | | | | | |  |
|  | | |  |  |  |  |  |  |  |  | | | | | | | |
|  | | | | | | | | | | | | 1 |  | BV |  | 14/01/2020 11:36 AM |  |
|  | | | I don’t know how long we’ll stay on Lutetium for because they’ve said if my markers are up, they’re going to start looking at other things, to find out why, and yeah, we’ll have one more go of it and they’ll have another look, because my cancer hasn’t reacted quite as fast as what they thought it might. | | | | | | | | | | | | | |  |
|  | | |  | | | | | | | | | | | | | |  |
|  | | | **Codes\\wanting to contribute to research** | | | | | | | | | | | | | |  |
|  |  |  |  |  | No |  | 0.0188 |  | 1 |  | | | | | | |  |
|  | | |  |  |  |  |  |  |  |  | | | | | | | |
|  | | | | | | | | | | | | 1 |  | BV |  | 14/01/2020 12:21 PM |  |
|  | | | that’s all I want to do, is pay it forward or pay it back, whatever you call it, because I want the next lot of people to, you know, to be thankful, and I’m sure they will be because of their situation, they’ll be as thankful that someone like me, has done what I’ve done, you know. And it’s not about any, purely, you know you’re in the line and you need to step up and do what everyone else has done, so you know, if you feel you can. | | | | | | | | | | | | | |  |
|  | | |  | | | | | | | | | | | | | |  |
|  | | | **Codes\\worry** | | | | | | | | | | | | | |  |
|  |  |  |  |  | No |  | 0.0455 |  | 3 |  | | | | | | |  |
|  | | |  |  |  |  |  |  |  |  | | | | | | | |
|  | | | | | | | | | | | | 1 |  | BV |  | 15/01/2020 5:33 AM |  |
|  | | | I think I’m coping quite well with it all. I mean, the little bit of unknown that we’re going through now, and the uncertainty of why my PSA isn’t dropping, is a little bit of a concern and that causes me a little bit of worry, because you know, you always worry why isn’t it going down. But they’re saying it can, you know, stay up for a while before it goes down so, we’re hoping we’ll see a change in this blood test, coming up in a couple of weeks. | | | | | | | | | | | | | |  |
|  | | |  | | | | | | | | | | | | | |  |
|  | | | | | | | | | | | | 2 |  | BV |  | 14/01/2020 11:41 AM |  |
|  | | | Like my PSA is still up over 30, it’s actually been going up, not down. So, they don’t know why, whether it’s a secondary type of cancer I’ve got that Lutetium’s treating, the prostate cancer, or whether, I don’t know, I don’t know. We’re not sure and that’s a little bit of the worry that comes with it all, is the uncertainty as to what’s happening. | | | | | | | | | | | | | |  |
|  | | |  | | | | | | | | | | | | | |  |
|  | | | | | | | | | | | | 3 |  | BV |  | 14/01/2020 11:46 AM |  |
|  | | | Purely, because you know, you get a new read on what you’re doing and it mightn’t be what you’re expecting, but you know they give you the confidence to not worry, or like panic, or not worry too much you know, any more than you, sort of would normally. | | | | | | | | | | | | | |  |
|  | | |  | | | | | | | | | | | | | |  |
|  | | | | | | | | | | | | | | | | | |
|  | | | | | | | | | | | | | | | | | |
| Formatted Reports\\Coding Summary by File Formatted Report | | | | | | | | | | | Page 58 of 143 | | | | | | |
| 6/10/2022 8:14 AM | | | | | | | | | | | | | | | | | |
|  | | | **Classification** |  | **Aggregate** |  | **Coverage** |  | **Number Of Coding References** |  | | **Reference Number** |  | **Coded By Initials** |  | **Modified On** |  |
|  | **Files\\Transcription 2_012** | | | | | | | | | | | | | | | |  |
|  | | **Code** | | | | | | | | | | | | | | |  |
|  | | | **Codes\\cure is worse than the disease** | | | | | | | | | | | | | |  |
|  |  |  |  |  | No |  | 0.0118 |  | 1 |  | | | | | | |  |
|  | | |  |  |  |  |  |  |  |  | | | | | | | |
|  | | | | | | | | | | | | 1 |  | BV |  | 14/01/2020 12:37 PM |  |
|  | | | Oh, not too bad lovey, not too bad. Not too bad, I don’t know whether the cure is worse than the disease. | | | | | | | | | | | | | |  |
|  | | |  | | | | | | | | | | | | | |  |
|  | | | **Codes\\doctor happy with progress** | | | | | | | | | | | | | |  |
|  |  |  |  |  | No |  | 0.0285 |  | 1 |  | | | | | | |  |
|  | | |  |  |  |  |  |  |  |  | | | | | | | |
|  | | | | | | | | | | | | 1 |  | BV |  | 15/01/2020 5:10 AM |  |
|  | | | I’ll have a talk to (name withheld) at the doctors and he sits me down and shows what it was and how it is you know, the last scan and this scan, and he said he can see that the cancer cells are fading out, they’re fading out, so it must be successful | | | | | | | | | | | | | |  |
|  | | |  | | | | | | | | | | | | | |  |
|  | | | **Codes\\facing mortality** | | | | | | | | | | | | | |  |
|  |  |  |  |  | No |  | 0.0439 |  | 1 |  | | | | | | |  |
|  | | |  |  |  |  |  |  |  |  | | | | | | | |
|  | | | | | | | | | | | | 1 |  | BV |  | 14/01/2020 12:47 PM |  |
|  | | | Just that (name withheld) said that this could be, this could be beneficial for you, and the PSA was rising you see. So, he was going to have to, some different sort of treatment, otherwise if the PSA keeps rising that’s the end of you. So, he’s given me this other one and there’s a few people on it he said, and you would probably benefit from this so, I’m in the process of benefitting. | | | | | | | | | | | | | |  |
|  | | |  | | | | | | | | | | | | | |  |
|  | | | **Codes\\financial impact\hospital parking** | | | | | | | | | | | | | |  |
|  |  |  |  |  | No |  | 0.0114 |  | 1 |  | | | | | | |  |
|  | | |  |  |  |  |  |  |  |  | | | | | | | |
|  | | | | | | | | | | | | 1 |  | BV |  | 14/01/2020 12:45 PM |  |
|  | | | All we’d like is just cheaper rates at the parking station at the hospital, but we can’t have that. | | | | | | | | | | | | | |  |
|  | | |  | | | | | | | | | | | | | |  |
|  | | | **Codes\\financial impact\no affect to finances** | | | | | | | | | | | | | |  |
|  |  |  |  |  | No |  | 0.0159 |  | 1 |  | | | | | | |  |
|  | | |  |  |  |  |  |  |  |  | | | | | | | |
|  | | | | | | | | | | | | 1 |  | BV |  | 14/01/2020 12:43 PM |  |
|  | | | The finances no, no, no, no, no. The finances are no great drama. My superannuation it comes every second Thursday, whether I need it or not. | | | | | | | | | | | | | |  |
|  | | |  | | | | | | | | | | | | | |  |
|  | | | | | | | | | | | | | | | | | |
| Formatted Reports\\Coding Summary by File Formatted Report | | | | | | | | | | | Page 59 of 143 | | | | | | |
| 6/10/2022 8:14 AM | | | | | | | | | | | | | | | | | |
|  | | | **Classification** |  | **Aggregate** |  | **Coverage** |  | **Number Of Coding References** |  | | **Reference Number** |  | **Coded By Initials** |  | **Modified On** |  |
|  | | | **Codes\\getting good results** | | | | | | | | | | | | | |  |
|  |  |  |  |  | No |  | 0.0285 |  | 1 |  | | | | | | |  |
|  | | |  |  |  |  |  |  |  |  | | | | | | | |
|  | | | | | | | | | | | | 1 |  | BV |  | 14/01/2020 12:41 PM |  |
|  | | | I’ll have a talk to (name withheld) at the doctors and he sits me down and shows what it was and how it is you know, the last scan and this scan, and he said he can see that the cancer cells are fading out, they’re fading out, so it must be successful | | | | | | | | | | | | | |  |
|  | | |  | | | | | | | | | | | | | |  |
|  | | | **Codes\\happy about participating** | | | | | | | | | | | | | |  |
|  |  |  |  |  | No |  | 0.0145 |  | 1 |  | | | | | | |  |
|  | | |  |  |  |  |  |  |  |  | | | | | | | |
|  | | | | | | | | | | | | 1 |  | BV |  | 14/01/2020 12:47 PM |  |
|  | | | I: Are you still happy that you’ve decided to enrol in the trial?  P: Oh yes, yes, yes, yes. I’ll persevere until the bitter end. | | | | | | | | | | | | | |  |
|  | | |  | | | | | | | | | | | | | |  |
|  | | | **Codes\\hope** | | | | | | | | | | | | | |  |
|  |  |  |  |  | No |  | 0.0568 |  | 3 |  | | | | | | |  |
|  | | |  |  |  |  |  |  |  |  | | | | | | | |
|  | | | | | | | | | | | | 1 |  | BV |  | 14/01/2020 12:38 PM |  |
|  | | | I hope so. I hope I’ll still be here talking to you yeah, but no, he doesn’t seem to be really concerned about any of these side effects, but one thing, the PSA has gone down so that’s something I dare say to be thankful for. | | | | | | | | | | | | | |  |
|  | | |  | | | | | | | | | | | | | |  |
|  | | | | | | | | | | | | 2 |  | BV |  | 15/01/2020 5:15 AM |  |
|  | | | Well, only I think there might be light at the end of the tunnel. I’ve had 2 of these treatments now and I believe I have to have 6, so there’s another 4 to go. | | | | | | | | | | | | | |  |
|  | | |  | | | | | | | | | | | | | |  |
|  | | | | | | | | | | | | 3 |  | BV |  | 14/01/2020 12:38 PM |  |
|  | | | Which is you know, rather terrifying. I mean hopefully at the end of this (indistinguishable) for ever and a day. | | | | | | | | | | | | | |  |
|  | | |  | | | | | | | | | | | | | |  |
|  | | | **Codes\\hope\hoping treatment lasts** | | | | | | | | | | | | | |  |
|  |  |  |  |  | No |  | 0.0255 |  | 1 |  | | | | | | |  |
|  | | |  |  |  |  |  |  |  |  | | | | | | | |
|  | | | | | | | | | | | | 1 |  | BV |  | 15/01/2020 9:55 AM |  |
|  | | | I hope so. I hope I’ll still be here talking to you yeah, but no, he doesn’t seem to be really concerned about any of these side effects, but one thing, the PSA has gone down so that’s something I dare say to be thankful for. | | | | | | | | | | | | | |  |
|  | | |  | | | | | | | | | | | | | |  |
|  | | | | | | | | | | | | | | | | | |
| Formatted Reports\\Coding Summary by File Formatted Report | | | | | | | | | | | Page 60 of 143 | | | | | | |
| 6/10/2022 8:14 AM | | | | | | | | | | | | | | | | | |
|  | | | **Classification** |  | **Aggregate** |  | **Coverage** |  | **Number Of Coding References** |  | | **Reference Number** |  | **Coded By Initials** |  | **Modified On** |  |
|  | | | **Codes\\initial motivation for participation** | | | | | | | | | | | | | |  |
|  |  |  |  |  | No |  | 0.0439 |  | 1 |  | | | | | | |  |
|  | | |  |  |  |  |  |  |  |  | | | | | | | |
|  | | | | | | | | | | | | 1 |  | BV |  | 15/01/2020 4:54 AM |  |
|  | | | Just that (name withheld) said that this could be, this could be beneficial for you, and the PSA was rising you see. So, he was going to have to, some different sort of treatment, otherwise if the PSA keeps rising that’s the end of you. So, he’s given me this other one and there’s a few people on it he said, and you would probably benefit from this so, I’m in the process of benefitting. | | | | | | | | | | | | | |  |
|  | | |  | | | | | | | | | | | | | |  |
|  | | | **Codes\\initial motivation for participation\doctors recommendation** | | | | | | | | | | | | | |  |
|  |  |  |  |  | No |  | 0.0439 |  | 1 |  | | | | | | |  |
|  | | |  |  |  |  |  |  |  |  | | | | | | | |
|  | | | | | | | | | | | | 1 |  | BV |  | 14/01/2020 12:47 PM |  |
|  | | | Just that (name withheld) said that this could be, this could be beneficial for you, and the PSA was rising you see. So, he was going to have to, some different sort of treatment, otherwise if the PSA keeps rising that’s the end of you. So, he’s given me this other one and there’s a few people on it he said, and you would probably benefit from this so, I’m in the process of benefitting. | | | | | | | | | | | | | |  |
|  | | |  | | | | | | | | | | | | | |  |
|  | | | **Codes\\keep battling** | | | | | | | | | | | | | |  |
|  |  |  |  |  | No |  | 0.0162 |  | 2 |  | | | | | | |  |
|  | | |  |  |  |  |  |  |  |  | | | | | | | |
|  | | | | | | | | | | | | 1 |  | BV |  | 15/01/2020 5:21 AM |  |
|  | | | I: Are you still happy that you’ve decided to enrol in the trial?  P: Oh yes, yes, yes, yes. I’ll persevere until the bitter end. | | | | | | | | | | | | | |  |
|  | | |  | | | | | | | | | | | | | |  |
|  | | | | | | | | | | | | 2 |  | BV |  | 15/01/2020 5:21 AM |  |
|  | | | I’ll persevere. | | | | | | | | | | | | | |  |
|  | | |  | | | | | | | | | | | | | |  |
|  | | | **Codes\\light at the end of the tunnel** | | | | | | | | | | | | | |  |
|  |  |  |  |  | No |  | 0.0183 |  | 1 |  | | | | | | |  |
|  | | |  |  |  |  |  |  |  |  | | | | | | | |
|  | | | | | | | | | | | | 1 |  | BV |  | 14/01/2020 12:38 PM |  |
|  | | | Well, only I think there might be light at the end of the tunnel. I’ve had 2 of these treatments now and I believe I have to have 6, so there’s another 4 to go. | | | | | | | | | | | | | |  |
|  | | |  | | | | | | | | | | | | | |  |
|  | | | **Codes\\no variations** | | | | | | | | | | | | | |  |
|  |  |  |  |  | No |  | 0.0629 |  | 3 |  | | | | | | |  |
|  | | |  |  |  |  |  |  |  |  | | | | | | | |
|  | | | | | | | | | | | | 1 |  | BV |  | 14/01/2020 12:48 PM |  |
|  | | | Well, see the thing is, this nuclear stuff comes up from Sydney on a certain day and it’s ready at a certain time, so they can’t entertain any variations at all, even if I was off to see the Queen of England I’d have to say, sorry X, but I have to go to the hospital. | | | | | | | | | | | | | |  |
|  | | |  | | | | | | | | | | | | | |  |
|  | | | | | | | | | | | | 2 |  | BV |  | 14/01/2020 12:48 PM |  |
|  | | | Ah yeah, go to the hospital, yeah leave the Queen on her own but that’s how it is, there can be no variation. Once I miss a treatment of course, well the whole process is in doubt. | | | | | | | | | | | | | |  |
|  | | |  | | | | | | | | | | | | | |  |
| Formatted Reports\\Coding Summary by File Formatted Report | | | | | | | | | | | Page 61 of 143 | | | | | | |
| 6/10/2022 8:14 AM | | | | | | | | | | | | | | | | | |
|  | | | **Classification** |  | **Aggregate** |  | **Coverage** |  | **Number Of Coding References** |  | | **Reference Number** |  | **Coded By Initials** |  | **Modified On** |  |
|  | | | | | | | | | | | | | | | | | |
|  | | | | | | | | | | | | 3 |  | BV |  | 14/01/2020 12:48 PM |  |
|  | | | I: It’s best to try and stick to your appointments, as best as you can.  P: In this case I have to yeah. Yeah. | | | | | | | | | | | | | |  |
|  | | |  | | | | | | | | | | | | | |  |
|  | | | **Codes\\PSA numbers going down** | | | | | | | | | | | | | |  |
|  |  |  |  |  | No |  | 0.0257 |  | 1 |  | | | | | | |  |
|  | | |  |  |  |  |  |  |  |  | | | | | | | |
|  | | | | | | | | | | | | 1 |  | BV |  | 14/01/2020 12:38 PM |  |
|  | | | I hope so. I hope I’ll still be here talking to you yeah, but no, he doesn’t seem to be really concerned about any of these side effects, but one thing, the PSA has gone down so that’s something I dare say to be thankful for. | | | | | | | | | | | | | |  |
|  | | |  | | | | | | | | | | | | | |  |
|  | | | **Codes\\PSA numbers going down\PSA numbers going down - lutetium** | | | | | | | | | | | | | |  |
|  |  |  |  |  | No |  | 0.0255 |  | 1 |  | | | | | | |  |
|  | | |  |  |  |  |  |  |  |  | | | | | | | |
|  | | | | | | | | | | | | 1 |  | BV |  | 25/03/2020 8:22 AM |  |
|  | | | I hope so. I hope I’ll still be here talking to you yeah, but no, he doesn’t seem to be really concerned about any of these side effects, but one thing, the PSA has gone down so that’s something I dare say to be thankful for. | | | | | | | | | | | | | |  |
|  | | |  | | | | | | | | | | | | | |  |
|  | | | **Codes\\quality of trialists** | | | | | | | | | | | | | |  |
|  |  |  |  |  | No |  | 0.0066 |  | 1 |  | | | | | | |  |
|  | | |  |  |  |  |  |  |  |  | | | | | | | |
|  | | | | | | | | | | | | 1 |  | BV |  | 14/01/2020 12:45 PM |  |
|  | | | They’re very, very good. Can’t speak highly enough of them. | | | | | | | | | | | | | |  |
|  | | |  | | | | | | | | | | | | | |  |
|  | | | **Codes\\side effects** | | | | | | | | | | | | | |  |
|  |  |  |  |  | No |  | 0.1082 |  | 5 |  | | | | | | |  |
|  | | |  |  |  |  |  |  |  |  | | | | | | | |
|  | | | | | | | | | | | | 1 |  | BV |  | 25/03/2020 8:26 AM |  |
|  | | | P: I’m starting to get some dizzy spells now.  I: Okay.  P: Now, I don’t know whether it’s because of the treatment or not, I don’t know. I’ve got to be careful when I walk along and turn my head very slowly otherwise, I think I’m going to fall over.  I: Have you been speaking to the trialists about this?  P: No, I don’t, it only just happened since the last lot, which was on Tuesday and Wednesday last week. | | | | | | | | | | | | | |  |
|  | | |  | | | | | | | | | | | | | |  |
|  | | | | | | | | | | | | 2 |  | BV |  | 25/03/2020 8:26 AM |  |
|  | | | I hope so. I hope I’ll still be here talking to you yeah, but no, he doesn’t seem to be really concerned about any of these side effects, but one thing, the PSA has gone down so that’s something I dare say to be thankful for. | | | | | | | | | | | | | |  |
|  | | |  | | | | | | | | | | | | | |  |
|  | | | | | | | | | | | | 3 |  | BV |  | 25/03/2020 8:26 AM |  |
|  | | | I do a lot of sleeping now, I do a lot of sleeping. | | | | | | | | | | | | | |  |
|  | | |  | | | | | | | | | | | | | |  |
| Formatted Reports\\Coding Summary by File Formatted Report | | | | | | | | | | | Page 62 of 143 | | | | | | |
| 6/10/2022 8:14 AM | | | | | | | | | | | | | | | | | |
|  | | | **Classification** |  | **Aggregate** |  | **Coverage** |  | **Number Of Coding References** |  | | **Reference Number** |  | **Coded By Initials** |  | **Modified On** |  |
|  | | | | | | | | | | | | | | | | | |
|  | | | | | | | | | | | | 4 |  | BV |  | 25/03/2020 8:26 AM |  |
|  | | | As I said, I get very tired, because I have to get up maybe 6 or 7 times a night to go for a comfort stop. Now whether that’s because I’m drinking a lot of coffee, or water, or whatever, I don’t know. | | | | | | | | | | | | | |  |
|  | | |  | | | | | | | | | | | | | |  |
|  | | | | | | | | | | | | 5 |  | BV |  | 25/03/2020 8:26 AM |  |
|  | | | I just get the feeling that the bladder might have shrunk a bit more. | | | | | | | | | | | | | |  |
|  | | |  | | | | | | | | | | | | | |  |
|  | | | **Codes\\side effects\side effects - lutetium** | | | | | | | | | | | | | |  |
|  |  |  |  |  | No |  | 0.1087 |  | 5 |  | | | | | | |  |
|  | | |  |  |  |  |  |  |  |  | | | | | | | |
|  | | | | | | | | | | | | 1 |  | BV |  | 14/01/2020 12:37 PM |  |
|  | | | P: I’m starting to get some dizzy spells now.  I: Okay.  P: Now, I don’t know whether it’s because of the treatment or not, I don’t know. I’ve got to be careful when I walk along and turn my head very slowly otherwise, I think I’m going to fall over.  I: Have you been speaking to the trialists about this?  P: No, I don’t, it only just happened since the last lot, which was on Tuesday and Wednesday last week. | | | | | | | | | | | | | |  |
|  | | |  | | | | | | | | | | | | | |  |
|  | | | | | | | | | | | | 2 |  | BV |  | 14/01/2020 12:38 PM |  |
|  | | | I hope so. I hope I’ll still be here talking to you yeah, but no, he doesn’t seem to be really concerned about any of these side effects, but one thing, the PSA has gone down so that’s something I dare say to be thankful for. | | | | | | | | | | | | | |  |
|  | | |  | | | | | | | | | | | | | |  |
|  | | | | | | | | | | | | 3 |  | BV |  | 14/01/2020 12:42 PM |  |
|  | | | I do a lot of sleeping now, I do a lot of sleeping. | | | | | | | | | | | | | |  |
|  | | |  | | | | | | | | | | | | | |  |
|  | | | | | | | | | | | | 4 |  | BV |  | 14/01/2020 12:43 PM |  |
|  | | | As I said, I get very tired, because I have to get up maybe 6 or 7 times a night to go for a comfort stop. Now whether that’s because I’m drinking a lot of coffee, or water, or whatever, I don’t know. | | | | | | | | | | | | | |  |
|  | | |  | | | | | | | | | | | | | |  |
|  | | | | | | | | | | | | 5 |  | BV |  | 14/01/2020 12:43 PM |  |
|  | | | I just get the feeling that the bladder might have shrunk a bit more. | | | | | | | | | | | | | |  |
|  | | |  | | | | | | | | | | | | | |  |
|  | | | **Codes\\struggling with treatment** | | | | | | | | | | | | | |  |
|  |  |  |  |  | No |  | 0.0656 |  | 2 |  | | | | | | |  |
|  | | |  |  |  |  |  |  |  |  | | | | | | | |
|  | | | | | | | | | | | | 1 |  | BV |  | 14/01/2020 12:40 PM |  |
|  | | | Well, when I go for the x-rays I have to, you know, those scans, they have to stop halfway through and go for a comfort stop, because my bladder capacity isn’t, doesn’t seem to be what it once was. | | | | | | | | | | | | | |  |
|  | | |  | | | | | | | | | | | | | |  |
|  | | | | | | | | | | | | 2 |  | BV |  | 14/01/2020 12:42 PM |  |
|  | | | P: Yeah, yeah. I mean, I don’t have a very, a very high pain threshold or anything like that.  I: So, you’ve, you’ve found it difficult.  P: Well, a little bit hard yeah, to sit on the… Well, the main thing is, I only get halfway through and then I got to go for a leak and then they say, we got to start again, and I think oh my godfather, must we, must we? Well, we have to, yeah | | | | | | | | | | | | | |  |
|  | | |  | | | | | | | | | | | | | |  |
| Formatted Reports\\Coding Summary by File Formatted Report | | | | | | | | | | | Page 63 of 143 | | | | | | |
| 6/10/2022 8:14 AM | | | | | | | | | | | | | | | | | |
|  | | | **Classification** |  | **Aggregate** |  | **Coverage** |  | **Number Of Coding References** |  | | **Reference Number** |  | **Coded By Initials** |  | **Modified On** |  |
|  | | | **Codes\\struggling with treatment\struggling with additional health issues** | | | | | | | | | | | | | |  |
|  |  |  |  |  | No |  | 0.0909 |  | 4 |  | | | | | | |  |
|  | | |  |  |  |  |  |  |  |  | | | | | | | |
|  | | | | | | | | | | | | 1 |  | BV |  | 14/01/2020 12:45 PM |  |
|  | | | That’s just me practising me cough. I’ve had the flu since about February, thank heavens I had the flu shot, otherwise I would have been worse. | | | | | | | | | | | | | |  |
|  | | |  | | | | | | | | | | | | | |  |
|  | | | | | | | | | | | | 2 |  | BV |  | 14/01/2020 12:45 PM |  |
|  | | | No, no, I said I’ve had the flu, the flu since February. | | | | | | | | | | | | | |  |
|  | | |  | | | | | | | | | | | | | |  |
|  | | | | | | | | | | | | 3 |  | BV |  | 14/01/2020 12:46 PM |  |
|  | | | I guess it is good that you did get a flu shot anyway.  P: Yeah, yeah, yeah, or it either, if you’re over 75 well, you’d better have one. Nothing hard in that, nothing hard. I’ve got a pulled tendon in my left arm. I think I might have told you; I fell over on the back veranda here about 6 weeks ago. | | | | | | | | | | | | | |  |
|  | | |  | | | | | | | | | | | | | |  |
|  | | | | | | | | | | | | 4 |  | BV |  | 14/01/2020 12:46 PM |  |
|  | | | Yeah, yeah. Fell over on the back veranda and I got a pulled tendon in my left arm, and I go out to see the physiotherapist at Allied Court so he’s trying to get it back again. It’ll only go as high as my shoulder now, my right arm goes all the way up, my left arm doesn’t, so trying to cure that as well. | | | | | | | | | | | | | |  |
|  | | |  | | | | | | | | | | | | | |  |
|  | | | **Codes\\struggling with treatment\struggling with post treatment scans** | | | | | | | | | | | | | |  |
|  |  |  |  |  | No |  | 0.0770 |  | 2 |  | | | | | | |  |
|  | | |  |  |  |  |  |  |  |  | | | | | | | |
|  | | | | | | | | | | | | 1 |  | BV |  | 15/01/2020 5:30 AM |  |
|  | | | P: Well, when I go for the x-rays I have to, you know, those scans, they have to stop halfway through and go for a comfort stop, because my bladder capacity isn’t, doesn’t seem to be what it once was.  I: Okay.  P: So, you know, that means they’ve got to stop the program, and do it again in 2 halves | | | | | | | | | | | | | |  |
|  | | |  | | | | | | | | | | | | | |  |
|  | | | | | | | | | | | | 2 |  | BV |  | 15/01/2020 5:30 AM |  |
|  | | | P: Yeah, yeah. I mean, I don’t have a very, a very high pain threshold or anything like that.  I: So, you’ve, you’ve found it difficult.  P: Well, a little bit hard yeah, to sit on the… Well, the main thing is, I only get halfway through and then I got to go for a leak and then they say, we got to start again, and I think oh my godfather, must we, must we? Well, we have to, yeah | | | | | | | | | | | | | |  |
|  | | |  | | | | | | | | | | | | | |  |
|  | | | **Codes\\support from others** | | | | | | | | | | | | | |  |
|  |  |  |  |  | No |  | 0.0222 |  | 1 |  | | | | | | |  |
|  | | |  |  |  |  |  |  |  |  | | | | | | | |
|  | | | | | | | | | | | | 1 |  | BV |  | 14/01/2020 12:46 PM |  |
|  | | | No, not really, not really. (name withheld) gets support from the Anglicare people, a cleaner comes once a fortnight to sweep the floor and do a bit of this and that. In fact, she’s due tomorrow. | | | | | | | | | | | | | |  |
|  | | |  | | | | | | | | | | | | | |  |
|  | | | | | | | | | | | | | | | | | |
| Formatted Reports\\Coding Summary by File Formatted Report | | | | | | | | | | | Page 64 of 143 | | | | | | |
| 6/10/2022 8:14 AM | | | | | | | | | | | | | | | | | |
|  | | | **Classification** |  | **Aggregate** |  | **Coverage** |  | **Number Of Coding References** |  | | **Reference Number** |  | **Coded By Initials** |  | **Modified On** |  |
|  | | | **Codes\\treatments been good** | | | | | | | | | | | | | |  |
|  |  |  |  |  | No |  | 0.0285 |  | 1 |  | | | | | | |  |
|  | | |  |  |  |  |  |  |  |  | | | | | | | |
|  | | | | | | | | | | | | 1 |  | BV |  | 15/01/2020 5:09 AM |  |
|  | | | I’ll have a talk to (name withheld) at the doctors and he sits me down and shows what it was and how it is you know, the last scan and this scan, and he said he can see that the cancer cells are fading out, they’re fading out, so it must be successful | | | | | | | | | | | | | |  |
|  | | |  | | | | | | | | | | | | | |  |
|  | | | **Codes\\treatments left** | | | | | | | | | | | | | |  |
|  |  |  |  |  | No |  | 0.0183 |  | 1 |  | | | | | | |  |
|  | | |  |  |  |  |  |  |  |  | | | | | | | |
|  | | | | | | | | | | | | 1 |  | BV |  | 15/01/2020 5:14 AM |  |
|  | | | Well, only I think there might be light at the end of the tunnel. I’ve had 2 of these treatments now and I believe I have to have 6, so there’s another 4 to go. | | | | | | | | | | | | | |  |
|  | | |  | | | | | | | | | | | | | |  |
|  | **Files\\Transcription 2_013** | | | | | | | | | | | | | | | |  |
|  | | **Code** | | | | | | | | | | | | | | |  |
|  | | | **Codes\\fear of the unknown** | | | | | | | | | | | | | |  |
|  |  |  |  |  | No |  | 0.0091 |  | 1 |  | | | | | | |  |
|  | | |  |  |  |  |  |  |  |  | | | | | | | |
|  | | | | | | | | | | | | 1 |  | BV |  | 14/01/2020 12:59 PM |  |
|  | | | So you know there’s a bit of that you know, fear of the unknown. | | | | | | | | | | | | | |  |
|  | | |  | | | | | | | | | | | | | |  |
|  | | | **Codes\\financial impact** | | | | | | | | | | | | | |  |
|  |  |  |  |  | No |  | 0.0141 |  | 1 |  | | | | | | |  |
|  | | |  |  |  |  |  |  |  |  | | | | | | | |
|  | | | | | | | | | | | | 1 |  | BV |  | 14/01/2020 12:59 PM |  |
|  | | | Oh a bit you know just you know you have to make sure you're careful with your finances you know. | | | | | | | | | | | | | |  |
|  | | |  | | | | | | | | | | | | | |  |
|  | | | **Codes\\financial impact\hospital parking** | | | | | | | | | | | | | |  |
|  |  |  |  |  | No |  | 0.0163 |  | 1 |  | | | | | | |  |
|  | | |  |  |  |  |  |  |  |  | | | | | | | |
|  | | | | | | | | | | | | 1 |  | BV |  | 14/01/2020 1:00 PM |  |
|  | | | Yeah well where we park is certainly, it's pretty tight if there’s a lot of people there on the day at … you know. | | | | | | | | | | | | | |  |
|  | | |  | | | | | | | | | | | | | |  |
| Formatted Reports\\Coding Summary by File Formatted Report | | | | | | | | | | | Page 65 of 143 | | | | | | |
| 6/10/2022 8:14 AM | | | | | | | | | | | | | | | | | |
|  | | | **Classification** |  | **Aggregate** |  | **Coverage** |  | **Number Of Coding References** |  | | **Reference Number** |  | **Coded By Initials** |  | **Modified On** |  |
|  | | | **Codes\\hope** | | | | | | | | | | | | | |  |
|  |  |  |  |  | No |  | 0.0277 |  | 1 |  | | | | | | |  |
|  | | |  |  |  |  |  |  |  |  | | | | | | | |
|  | | | | | | | | | | | | 1 |  | BV |  | 14/01/2020 1:01 PM |  |
|  | | | I hope to get some positive outcomes of it all. And that aspect hasn’t changed.  Q: Good, so you feel like it's getting towards that, or working towards that?  A: I hope so yes, I hope so. | | | | | | | | | | | | | |  |
|  | | |  | | | | | | | | | | | | | |  |
|  | | | **Codes\\hope\hoping treatment lasts** | | | | | | | | | | | | | |  |
|  |  |  |  |  | No |  | 0.0115 |  | 1 |  | | | | | | |  |
|  | | |  |  |  |  |  |  |  |  | | | | | | | |
|  | | | | | | | | | | | | 1 |  | BV |  | 15/01/2020 9:56 AM |  |
|  | | | I hope to get some positive outcomes of it all. And that aspect hasn’t changed. | | | | | | | | | | | | | |  |
|  | | |  | | | | | | | | | | | | | |  |
|  | | | **Codes\\initial motivation for participation** | | | | | | | | | | | | | |  |
|  |  |  |  |  | No |  | 0.0161 |  | 1 |  | | | | | | |  |
|  | | |  |  |  |  |  |  |  |  | | | | | | | |
|  | | | | | | | | | | | | 1 |  | BV |  | 14/01/2020 1:00 PM |  |
|  | | | No it's still about the same; I hope to get some positive outcomes of it all. And that aspect hasn’t changed. | | | | | | | | | | | | | |  |
|  | | |  | | | | | | | | | | | | | |  |
|  | | | **Codes\\initial motivation for participation\changes to initial motivation for participation** | | | | | | | | | | | | | |  |
|  |  |  |  |  | No |  | 0.0320 |  | 1 |  | | | | | | |  |
|  | | |  |  |  |  |  |  |  |  | | | | | | | |
|  | | | | | | | | | | | | 1 |  | BV |  | 14/01/2020 1:01 PM |  |
|  | | | No it's still about the same; I hope to get some positive outcomes of it all. And that aspect hasn’t changed.  Q: Good, so you feel like it's getting towards that, or working towards that?  A: I hope so yes, I hope so. | | | | | | | | | | | | | |  |
|  | | |  | | | | | | | | | | | | | |  |
|  | | | **Codes\\needing better communication from doctor** | | | | | | | | | | | | | |  |
|  |  |  |  |  | No |  | 0.1606 |  | 5 |  | | | | | | |  |
|  | | |  |  |  |  |  |  |  |  | | | | | | | |
|  | | | | | | | | | | | | 1 |  | BV |  | 14/01/2020 1:00 PM |  |
|  | | | Probably in some case a bit more explanation. | | | | | | | | | | | | | |  |
|  | | |  | | | | | | | | | | | | | |  |
|  | | | | | | | | | | | | 2 |  | BV |  | 14/01/2020 1:00 PM |  |
|  | | | With what was, you know what was happening and that. I just felt sometimes they you know sort of rushed the thing a little bit, not necessarily their fault but you know they had a certain amount to get through and they got through it you know. | | | | | | | | | | | | | |  |
|  | | |  | | | | | | | | | | | | | |  |
| Formatted Reports\\Coding Summary by File Formatted Report | | | | | | | | | | | Page 66 of 143 | | | | | | |
| 6/10/2022 8:14 AM | | | | | | | | | | | | | | | | | |
|  | | | **Classification** |  | **Aggregate** |  | **Coverage** |  | **Number Of Coding References** |  | | **Reference Number** |  | **Coded By Initials** |  | **Modified On** |  |
|  | | | | | | | | | | | | | | | | | |
|  | | | | | | | | | | | | 3 |  | BV |  | 14/01/2020 1:00 PM |  |
|  | | | Q: You would have liked them to talk it through with you a bit more?  A: Yes, yeah I mean it was done to a certain extent, and some people do it naturally, whereas others aren’t prone to do it naturally you know. Like you know you have to sort of nudge them a bit I think. | | | | | | | | | | | | | |  |
|  | | |  | | | | | | | | | | | | | |  |
|  | | | | | | | | | | | | 4 |  | BV |  | 14/01/2020 1:01 PM |  |
|  | | | If possible, if things change you know like if symptoms changed or your approach changed or something new was introduced you know, yeah it would, it would be handy to get sort of feedback on that sort of thing. It may not directly involve me I don't know.  Q: Okay. What kind of support do you think you might require?  A: Explanations and things you know and what's expected and so on if necessary. | | | | | | | | | | | | | |  |
|  | | |  | | | | | | | | | | | | | |  |
|  | | | | | | | | | | | | 5 |  | BV |  | 14/01/2020 1:01 PM |  |
|  | | | Q: Okay, so just someone to have a proper sit down with and answer questions and that sort of stuff?  A: Yeah and have a proper exit situation. | | | | | | | | | | | | | |  |
|  | | |  | | | | | | | | | | | | | |  |
|  | | | **Codes\\needing more support** | | | | | | | | | | | | | |  |
|  |  |  |  |  | No |  | 0.0989 |  | 2 |  | | | | | | |  |
|  | | |  |  |  |  |  |  |  |  | | | | | | | |
|  | | | | | | | | | | | | 1 |  | BV |  | 14/01/2020 1:01 PM |  |
|  | | | If possible, if things change you know like if symptoms changed or your approach changed or something new was introduced you know, yeah it would, it would be handy to get sort of feedback on that sort of thing. It may not directly involve me I don't know.  Q: Okay. What kind of support do you think you might require?  A: Explanations and things you know and what's expected and so on if necessary. | | | | | | | | | | | | | |  |
|  | | |  | | | | | | | | | | | | | |  |
|  | | | | | | | | | | | | 2 |  | BV |  | 14/01/2020 1:02 PM |  |
|  | | | Q: Okay, so just someone to have a proper sit down with and answer questions and that sort of stuff?  A: Yeah and have a proper exit situation.  Q: Oh yep that, I understand that definitely. So that's what you'd like going forward from this point, is that correct?  A: Yes. | | | | | | | | | | | | | |  |
|  |  |  |  |  |  |  |  |  |  |  |  |  |  |  |  |  |  |
|  | | |  | | | | | | | | | | | | | |  |
|  | | | **Codes\\quality of life changes** | | | | | | | | | | | | | |  |
|  |  |  |  |  | No |  | 0.0120 |  | 1 |  | | | | | | |  |
|  | | |  |  |  |  |  |  |  |  | | | | | | | |
|  | | | | | | | | | | | | 1 |  | BV |  | 25/03/2020 8:25 AM |  |
|  | | | Q: Mm, Hmm. Okay have you had any quality of life changes?  A: No not really, no. | | | | | | | | | | | | | |  |
|  | | |  | | | | | | | | | | | | | |  |
|  | | | | | | | | | | | | | | | | | |
| Formatted Reports\\Coding Summary by File Formatted Report | | | | | | | | | | | Page 67 of 143 | | | | | | |
| 6/10/2022 8:14 AM | | | | | | | | | | | | | | | | | |
|  | | | **Classification** |  | **Aggregate** |  | **Coverage** |  | **Number Of Coding References** |  | | **Reference Number** |  | **Coded By Initials** |  | **Modified On** |  |
|  | | | **Codes\\quality of life changes\quality of life changes - lutetium** | | | | | | | | | | | | | |  |
|  |  |  |  |  | No |  | 0.0120 |  | 1 |  | | | | | | |  |
|  | | |  |  |  |  |  |  |  |  | | | | | | | |
|  | | | | | | | | | | | | 1 |  | BV |  | 14/01/2020 12:54 PM |  |
|  | | | Q: Mm, Hmm. Okay have you had any quality of life changes?  A: No not really, no. | | | | | | | | | | | | | |  |
|  | | |  | | | | | | | | | | | | | |  |
|  | | | **Codes\\quality of trialists** | | | | | | | | | | | | | |  |
|  |  |  |  |  | No |  | 0.0219 |  | 1 |  | | | | | | |  |
|  | | |  |  |  |  |  |  |  |  | | | | | | | |
|  | | | | | | | | | | | | 1 |  | BV |  | 14/01/2020 12:52 PM |  |
|  | | | The knowledge of the people involved has been very good, and their ability to deal with different people and different situations I founds pretty good. | | | | | | | | | | | | | |  |
|  | | |  | | | | | | | | | | | | | |  |
|  | | | **Codes\\quality of trialists\not keen about hospitals** | | | | | | | | | | | | | |  |
|  |  |  |  |  | No |  | 0.0286 |  | 1 |  | | | | | | |  |
|  | | |  |  |  |  |  |  |  |  | | | | | | | |
|  | | | | | | | | | | | | 1 |  | BV |  | 14/01/2020 12:59 PM |  |
|  | | | Oh yeah I think sometimes we all get a bit of that on these things, because you know there's an … of anticipation and that, you know and anything to do with hospitals I'm never overly keen about so. | | | | | | | | | | | | | |  |
|  | | |  | | | | | | | | | | | | | |  |
|  | | | **Codes\\side effects** | | | | | | | | | | | | | |  |
|  |  |  |  |  | No |  | 0.0584 |  | 2 |  | | | | | | |  |
|  | | |  |  |  |  |  |  |  |  | | | | | | | |
|  | | | | | | | | | | | | 1 |  | BV |  | 25/03/2020 8:26 AM |  |
|  | | | Well I notice and I've made aware of this but it's one of the side effects of the meds that I'm on is that it makes my throat very dry and my lips very dry. They're aware of that and I understand that it's also … with other patients as well. | | | | | | | | | | | | | |  |
|  | | |  | | | | | | | | | | | | | |  |
|  | | | | | | | | | | | | 2 |  | BV |  | 25/03/2020 8:26 AM |  |
|  | | | Well I found that one, last week I had a session I sort of had 4 days where I had different things on each day and I found that pretty sort of very, made me tired. | | | | | | | | | | | | | |  |
|  | | |  | | | | | | | | | | | | | |  |
|  | | | **Codes\\side effects\side effects - lutetium** | | | | | | | | | | | | | |  |
|  |  |  |  |  | No |  | 0.0584 |  | 2 |  | | | | | | |  |
|  | | |  |  |  |  |  |  |  |  | | | | | | | |
|  | | | | | | | | | | | | 1 |  | BV |  | 14/01/2020 12:52 PM |  |
|  | | | Well I notice and I've made aware of this but it's one of the side effects of the meds that I'm on is that it makes my throat very dry and my lips very dry. They're aware of that and I understand that it's also … with other patients as well. | | | | | | | | | | | | | |  |
|  | | |  | | | | | | | | | | | | | |  |
|  | | | | | | | | | | | | | | | | | |
| Formatted Reports\\Coding Summary by File Formatted Report | | | | | | | | | | | Page 68 of 143 | | | | | | |
| 6/10/2022 8:14 AM | | | | | | | | | | | | | | | | | |
|  | | | **Classification** |  | **Aggregate** |  | **Coverage** |  | **Number Of Coding References** |  | | **Reference Number** |  | **Coded By Initials** |  | **Modified On** |  |
|  | | | | | | | | | | | | | | | | | |
|  | | | | | | | | | | | | 2 |  | BV |  | 14/01/2020 12:53 PM |  |
|  | | | Well I found that one, last week I had a session I sort of had 4 days where I had different things on each day and I found that pretty sort of very, made me tired. | | | | | | | | | | | | | |  |
|  | | |  | | | | | | | | | | | | | |  |
|  | | | **Codes\\struggling with treatment** | | | | | | | | | | | | | |  |
|  |  |  |  |  | No |  | 0.1013 |  | 2 |  | | | | | | |  |
|  | | |  |  |  |  |  |  |  |  | | | | | | | |
|  | | | | | | | | | | | | 1 |  | BV |  | 14/01/2020 12:53 PM |  |
|  | | | A: Well I found that one, last week I had a session I sort of had 4 days where I had different things on each day and I found that pretty sort of very, made me tired.  Q: Okay.  A: I should have probably, with hindsight it should have been perhaps spread out over 3 days and a single day or something you know. | | | | | | | | | | | | | |  |
|  | | |  | | | | | | | | | | | | | |  |
|  | | | | | | | | | | | | 2 |  | BV |  | 14/01/2020 12:54 PM |  |
|  | | | Because you know at some of the tests you have and that are fairly intensive in terms of like you know if you're going in to have scans and that you're on the machine for a fair while and pretty uncomfortable.  Q: Yeah I can imagine.  A: So that sort of practical thing you know can have an issue with you. Because often you know you get off and you're pretty cramped up for a while. | | | | | | | | | | | | | |  |
|  |  |  |  |  |  |  |  |  |  |  |  |  |  |  |  |  |  |
|  | | |  | | | | | | | | | | | | | |  |
|  | | | **Codes\\struggling with treatment\struggling with post treatment scans** | | | | | | | | | | | | | |  |
|  |  |  |  |  | No |  | 0.0562 |  | 1 |  | | | | | | |  |
|  | | |  |  |  |  |  |  |  |  | | | | | | | |
|  | | | | | | | | | | | | 1 |  | BV |  | 15/01/2020 5:30 AM |  |
|  | | | Because you know at some of the tests you have and that are fairly intensive in terms of like you know if you're going in to have scans and that you're on the machine for a fair while and pretty uncomfortable.  Q: Yeah I can imagine.  A: So that sort of practical thing you know can have an issue with you. Because often you know you get off and you're pretty cramped up for a while. | | | | | | | | | | | | | |  |
|  |  |  |  |  |  |  |  |  |  |  |  |  |  |  |  |  |  |
|  | | |  | | | | | | | | | | | | | |  |
|  | | | **Codes\\travelling to treatment** | | | | | | | | | | | | | |  |
|  |  |  |  |  | No |  | 0.0190 |  | 1 |  | | | | | | |  |
|  | | |  |  |  |  |  |  |  |  | | | | | | | |
|  | | | | | | | | | | | | 1 |  | BV |  | 14/01/2020 1:00 PM |  |
|  | | | Nah I've been driving a bit and getting public transport, depending on what was happening you know. The session I was on you know. | | | | | | | | | | | | | |  |
|  | | |  | | | | | | | | | | | | | |  |
|  | | | | | | | | | | | | | | | | | |
| Formatted Reports\\Coding Summary by File Formatted Report | | | | | | | | | | | Page 69 of 143 | | | | | | |
| 6/10/2022 8:14 AM | | | | | | | | | | | | | | | | | |
|  | | | **Classification** |  | **Aggregate** |  | **Coverage** |  | **Number Of Coding References** |  | | **Reference Number** |  | **Coded By Initials** |  | **Modified On** |  |
|  | | | **Codes\\treatments been good** | | | | | | | | | | | | | |  |
|  |  |  |  |  | No |  | 0.0028 |  | 1 |  | | | | | | |  |
|  | | |  |  |  |  |  |  |  |  | | | | | | | |
|  | | | | | | | | | | | | 1 |  | BV |  | 14/01/2020 12:51 PM |  |
|  | | | Yeah it's been good. | | | | | | | | | | | | | |  |
|  | | |  | | | | | | | | | | | | | |  |
|  | | | **Codes\\treatments left** | | | | | | | | | | | | | |  |
|  |  |  |  |  | No |  | 0.0183 |  | 2 |  | | | | | | |  |
|  | | |  |  |  |  |  |  |  |  | | | | | | | |
|  | | | | | | | | | | | | 1 |  | BV |  | 14/01/2020 12:51 PM |  |
|  | | | No I've still got another, oh about 6 weeks to go, so I've got 3 sessions to go yet. | | | | | | | | | | | | | |  |
|  | | |  | | | | | | | | | | | | | |  |
|  | | | | | | | | | | | | 2 |  | BV |  | 14/01/2020 12:51 PM |  |
|  | | | I'm about half way through it at the moment. | | | | | | | | | | | | | |  |
|  | | |  | | | | | | | | | | | | | |  |
|  | | | **Codes\\uncertainty of the future** | | | | | | | | | | | | | |  |
|  |  |  |  |  | No |  | 0.0463 |  | 3 |  | | | | | | |  |
|  | | |  |  |  |  |  |  |  |  | | | | | | | |
|  | | | | | | | | | | | | 1 |  | BV |  | 15/01/2020 4:58 AM |  |
|  | | | So you know there’s a bit of that you know, fear of the unknown. | | | | | | | | | | | | | |  |
|  | | |  | | | | | | | | | | | | | |  |
|  | | | | | | | | | | | | 2 |  | BV |  | 14/01/2020 1:02 PM |  |
|  | | | Yeah and have a proper exit situation.  Q: Oh yep that, I understand that definitely. So that's what you'd like going forward from this point, is that correct?  A: Yes. | | | | | | | | | | | | | |  |
|  | | |  | | | | | | | | | | | | | |  |
|  | | | | | | | | | | | | 3 |  | BV |  | 14/01/2020 1:02 PM |  |
|  | | | I'd like … it just depends on what transpires I think, … crystal ball goes in there. | | | | | | | | | | | | | |  |
|  | | |  | | | | | | | | | | | | | |  |
|  | | | | | | | | | | | | | | | | | |
|  | | | | | | | | | | | | | | | | | |
| Formatted Reports\\Coding Summary by File Formatted Report | | | | | | | | | | | Page 70 of 143 | | | | | | |
| 6/10/2022 8:14 AM | | | | | | | | | | | | | | | | | |
|  | | | **Classification** |  | **Aggregate** |  | **Coverage** |  | **Number Of Coding References** |  | | **Reference Number** |  | **Coded By Initials** |  | **Modified On** |  |
|  | **Files\\Transcription 2_014** | | | | | | | | | | | | | | | |  |
|  | | **Code** | | | | | | | | | | | | | | |  |
|  | | | **Codes\\coping strategies\coping okay** | | | | | | | | | | | | | |  |
|  |  |  |  |  | No |  | 0.0032 |  | 1 |  | | | | | | |  |
|  | | |  |  |  |  |  |  |  |  | | | | | | | |
|  | | | | | | | | | | | | 1 |  | BV |  | 15/01/2020 4:58 AM |  |
|  | | | From a coping point of view, no problems | | | | | | | | | | | | | |  |
|  | | |  | | | | | | | | | | | | | |  |
|  | | | **Codes\\doctor happy with progress** | | | | | | | | | | | | | |  |
|  |  |  |  |  | No |  | 0.0266 |  | 1 |  | | | | | | |  |
|  | | |  |  |  |  |  |  |  |  | | | | | | | |
|  | | | | | | | | | | | | 1 |  | BV |  | 15/01/2020 4:45 AM |  |
|  | | | yeah, no, I did, you know, in my consultation with the consultant …, you know, … the trials and … (Inaudible) if this proves to be. …, you know, it … ineffective going forward, you know, the treatment I’m on … that looks as though it’s going to be … that there are other things we can try but – so, no, he was, he was positive- | | | | | | | | | | | | | |  |
|  | | |  | | | | | | | | | | | | | |  |
|  | | | **Codes\\emotional impact** | | | | | | | | | | | | | |  |
|  |  |  |  |  | No |  | 0.1346 |  | 4 |  | | | | | | |  |
|  | | |  |  |  |  |  |  |  |  | | | | | | | |
|  | | | | | | | | | | | | 1 |  | BV |  | 15/01/2020 4:59 AM |  |
|  | | | From a coping point of view, no problems but the trial – the first few – couple of cycles at the trial, they – we did seem to be making in roads into bringing my PSA down, but the last couple of readings we’ve had the PSA seems to have levelled off. So, that – I was a bit – I have to say, I’ve been a bit disappointed about that, you know, I was, you know, I was hoping that my PSA would be coming right back down. | | | | | | | | | | | | | |  |
|  | | |  | | | | | | | | | | | | | |  |
|  | | | | | | | | | | | | 2 |  | BV |  | 15/01/2020 4:59 AM |  |
|  | | | I had, you know, I had, I had treatment with a drug called … and – many years ago; soon after I had my operation and, and that brought my PSA levels back down to a sort of almost rock bottom. But … hasn’t done that, so far. Now, I don’t know if things will change but at the moment – after my last consultation with the specialist, I felt as though we were perhaps at the point now where we were keeping the cancer in check but not bringing it down or, or, you know, eradicating it. And so, I came away from the last consultation, which was about nine or ten days ago, feeling a bit down about that and starting to think, well, you know, it’s – what else – what, what’s next sort of – what can we do next? | | | | | | | | | | | | | |  |
|  | | |  | | | | | | | | | | | | | |  |
|  | | | | | | | | | | | | 3 |  | BV |  | 15/01/2020 4:59 AM |  |
|  | | | so, yeah, so little bit of a knock back over the last week and a half, I would say. | | | | | | | | | | | | | |  |
|  | | |  | | | | | | | | | | | | | |  |
|  | | | | | | | | | | | | 4 |  | BV |  | 15/01/2020 4:59 AM |  |
|  | | | just to mention, yeah, I just … it wasn’t just the, the effects of the PSA levelling off, it was also I’d had … the mid, mid-trial scans or the eleven week scans and that actually showed that the, you know, the cancer had stopped spreading; largely stopped spreading but hadn’t reduced. So, that was also I think contributing to, you know, … my afterwards feeling a bit down about actually the, the effects of the treatment. You know, … whether it was- | | | | | | | | | | | | | |  |
|  | | |  | | | | | | | | | | | | | |  |
|  | | | | | | | | | | | | | | | | | |
| Formatted Reports\\Coding Summary by File Formatted Report | | | | | | | | | | | Page 71 of 143 | | | | | | |
| 6/10/2022 8:14 AM | | | | | | | | | | | | | | | | | |
|  | | | **Classification** |  | **Aggregate** |  | **Coverage** |  | **Number Of Coding References** |  | | **Reference Number** |  | **Coded By Initials** |  | **Modified On** |  |
|  | | | **Codes\\facing mortality** | | | | | | | | | | | | | |  |
|  |  |  |  |  | No |  | 0.0362 |  | 1 |  | | | | | | |  |
|  | | |  |  |  |  |  |  |  |  | | | | | | | |
|  | | | | | | | | | | | | 1 |  | BV |  | 14/01/2020 1:18 PM |  |
|  | | | And this has been a long journey for me. You know, I had my operation about – seven years ago and over that time, you know, I’ve had operation, I’ve had chemo, I’ve had hormone treatment, and, and – you know, I’ve been very positive about doing things, you know, lots of exercise. Doing lots of things to, you know, to, to challenge the cancer but I have to say, I came away last time thinking, … you know, is, you know, is the end stage looming? | | | | | | | | | | | | | |  |
|  | | |  | | | | | | | | | | | | | |  |
|  | | | **Codes\\feeling disappointed** | | | | | | | | | | | | | |  |
|  |  |  |  |  | No |  | 0.2049 |  | 6 |  | | | | | | |  |
|  | | |  |  |  |  |  |  |  |  | | | | | | | |
|  | | | | | | | | | | | | 1 |  | BV |  | 14/01/2020 1:18 PM |  |
|  | | | From a coping point of view, no problems but the trial – the first few – couple of cycles at the trial, they – we did seem to be making in roads into bringing my PSA down, but the last couple of readings we’ve had the PSA seems to have levelled off. So, that – I was a bit – I have to say, I’ve been a bit disappointed about that, you know, I was, you know, I was hoping that my PSA would be coming right back down. | | | | | | | | | | | | | |  |
|  | | |  | | | | | | | | | | | | | |  |
|  | | | | | | | | | | | | 2 |  | BV |  | 14/01/2020 1:17 PM |  |
|  | | | I had, you know, I had, I had treatment with a drug called … and – many years ago; soon after I had my operation and, and that brought my PSA levels back down to a sort of almost rock bottom. But … hasn’t done that, so far. Now, I don’t know if things will change but at the moment – after my last consultation with the specialist, I felt as though we were perhaps at the point now where we were keeping the cancer in check but not bringing it down or, or, you know, eradicating it. And so, I came away from the last consultation, which was about nine or ten days ago, feeling a bit down about that and starting to think, well, you know, it’s – what else – what, what’s next sort of – what can we do next? | | | | | | | | | | | | | |  |
|  | | |  | | | | | | | | | | | | | |  |
|  | | | | | | | | | | | | 3 |  | BV |  | 14/01/2020 1:18 PM |  |
|  | | | so, yeah, so little bit of a knock back over the last week and a half, I would say. | | | | | | | | | | | | | |  |
|  | | |  | | | | | | | | | | | | | |  |
|  | | | | | | | | | | | | 4 |  | BV |  | 14/01/2020 1:19 PM |  |
|  | | | And this has been a long journey for me. You know, I had my operation about – seven years ago and over that time, you know, I’ve had operation, I’ve had chemo, I’ve had hormone treatment, and, and – you know, I’ve been very positive about doing things, you know, lots of exercise. Doing lots of things to, you know, to, to challenge the cancer but I have to say, I came away last time thinking, … you know, is, you know, is the end stage looming? Now, I don’t mean to be alarmist. I mean I’m not expecting that to be, you know, any time quite soon, but, you know, am I getting to the - … starting to wonder whether I was getting to the stage in my treatment where, you know, maybe, maybe, …, you know, the finish line is, is out there and in the not too distant future. | | | | | | | | | | | | | |  |
|  | | |  | | | | | | | | | | | | | |  |
|  | | | | | | | | | | | | 5 |  | BV |  | 14/01/2020 1:33 PM |  |
|  | | | just to mention, yeah, I just … it wasn’t just the, the effects of the PSA levelling off, it was also I’d had … the mid, mid-trial scans or the eleven week scans and that actually showed that the, you know, the cancer had stopped spreading; largely stopped spreading but hadn’t reduced. So, that was also I think contributing to, you know, … my afterwards feeling a bit down about actually the, the effects of the treatment. You know, … whether it was- | | | | | | | | | | | | | |  |
|  | | |  | | | | | | | | | | | | | |  |
|  | | | | | | | | | | | | 6 |  | BV |  | 14/01/2020 1:33 PM |  |
|  | | | (Inaudible) hadn’t really done what I had hoped it might do in, in the early part of the trail. | | | | | | | | | | | | | |  |
|  | | |  | | | | | | | | | | | | | |  |
|  | | | | | | | | | | | | | | | | | |
| Formatted Reports\\Coding Summary by File Formatted Report | | | | | | | | | | | Page 72 of 143 | | | | | | |
| 6/10/2022 8:14 AM | | | | | | | | | | | | | | | | | |
|  | | | **Classification** |  | **Aggregate** |  | **Coverage** |  | **Number Of Coding References** |  | | **Reference Number** |  | **Coded By Initials** |  | **Modified On** |  |
|  | | | **Codes\\financial impact** | | | | | | | | | | | | | |  |
|  |  |  |  |  | No |  | 0.0180 |  | 1 |  | | | | | | |  |
|  | | |  |  |  |  |  |  |  |  | | | | | | | |
|  | | | | | | | | | | | | 1 |  | BV |  | 14/01/2020 1:21 PM |  |
|  | | | throughout the course of my illness I took the decision to stop work. So, I took probably retirement a little bit earlier than I had perhaps intended. But no, financially, so far no, no issues. No, we’re, we’re okay. | | | | | | | | | | | | | |  |
|  | | |  | | | | | | | | | | | | | |  |
|  | | | **Codes\\financial impact\hospital parking** | | | | | | | | | | | | | |  |
|  |  |  |  |  | No |  | 0.0025 |  | 1 |  | | | | | | |  |
|  | | |  |  |  |  |  |  |  |  | | | | | | | |
|  | | | | | | | | | | | | 1 |  | BV |  | 14/01/2020 1:20 PM |  |
|  | | | no free parking at the hospital | | | | | | | | | | | | | |  |
|  | | |  | | | | | | | | | | | | | |  |
|  | | | **Codes\\financial impact\no affect to finances** | | | | | | | | | | | | | |  |
|  |  |  |  |  | No |  | 0.0294 |  | 2 |  | | | | | | |  |
|  | | |  |  |  |  |  |  |  |  | | | | | | | |
|  | | | | | | | | | | | | 1 |  | BV |  | 14/01/2020 1:20 PM |  |
|  | | | No, - financially, no. No, I mean, you know, I’m on the trial. So, the chemotherapy is not being paid for. … the drugs have to be paid for | | | | | | | | | | | | | |  |
|  | | |  | | | | | | | | | | | | | |  |
|  | | | | | | | | | | | | 2 |  | BV |  | 14/01/2020 1:21 PM |  |
|  | | | throughout the course of my illness I took the decision to stop work. So, I took probably retirement a little bit earlier than I had perhaps intended. But no, financially, so far no, no issues. No, we’re, we’re okay. | | | | | | | | | | | | | |  |
|  | | |  | | | | | | | | | | | | | |  |
|  | | | **Codes\\happy about participating** | | | | | | | | | | | | | |  |
|  |  |  |  |  | No |  | 0.0171 |  | 2 |  | | | | | | |  |
|  | | |  |  |  |  |  |  |  |  | | | | | | | |
|  | | | | | | | | | | | | 1 |  | BV |  | 14/01/2020 1:24 PM |  |
|  | | | I mean I, I’m happy that I am. You know, I’m pleased that I am | | | | | | | | | | | | | |  |
|  | | |  | | | | | | | | | | | | | |  |
|  | | | | | | | | | | | | 2 |  | BV |  | 14/01/2020 1:25 PM |  |
|  | | | I feel as though, you know, I’m contributing a bit to, to medical science research …-  Q: Yeah.  A: And no, it’s pleasing to be able to do that. | | | | | | | | | | | | | |  |
|  | | |  | | | | | | | | | | | | | |  |
|  | | | | | | | | | | | | | | | | | |
| Formatted Reports\\Coding Summary by File Formatted Report | | | | | | | | | | | Page 73 of 143 | | | | | | |
| 6/10/2022 8:14 AM | | | | | | | | | | | | | | | | | |
|  | | | **Classification** |  | **Aggregate** |  | **Coverage** |  | **Number Of Coding References** |  | | **Reference Number** |  | **Coded By Initials** |  | **Modified On** |  |
|  | | | **Codes\\hope** | | | | | | | | | | | | | |  |
|  |  |  |  |  | No |  | 0.0365 |  | 1 |  | | | | | | |  |
|  | | |  |  |  |  |  |  |  |  | | | | | | | |
|  | | | | | | | | | | | | 1 |  | BV |  | 15/01/2020 5:21 AM |  |
|  | | | I mean, you know, my, my only wish is that, you know, is that next time I get to have my PSA checked, it might have dropped a bit further.  Q: Yeah, I’m hoping that for you as well.  A: Yeah, thank you.  Q: Definitely.  A: Otherwise, you know, it’s, you know, I think the time will come … even perhaps before the end of the trial when …, you know, in conjunction with the consultant, we have to think about maybe a plan B going forward. | | | | | | | | | | | | | |  |
|  |  |  |  |  |  |  |  |  |  |  |  |  |  |  |  |  |  |
|  | | |  | | | | | | | | | | | | | |  |
|  | | | **Codes\\hope\hoping PSA dropped further** | | | | | | | | | | | | | |  |
|  |  |  |  |  | No |  | 0.0364 |  | 1 |  | | | | | | |  |
|  | | |  |  |  |  |  |  |  |  | | | | | | | |
|  | | | | | | | | | | | | 1 |  | BV |  | 15/01/2020 9:54 AM |  |
|  | | | I mean, you know, my, my only wish is that, you know, is that next time I get to have my PSA checked, it might have dropped a bit further.  Q: Yeah, I’m hoping that for you as well.  A: Yeah, thank you.  Q: Definitely.  A: Otherwise, you know, it’s, you know, I think the time will come … even perhaps before the end of the trial when …, you know, in conjunction with the consultant, we have to think about maybe a plan B going forward. | | | | | | | | | | | | | |  |
|  |  |  |  |  |  |  |  |  |  |  |  |  |  |  |  |  |  |
|  | | |  | | | | | | | | | | | | | |  |
|  | | | **Codes\\initial motivation for participation\failure in previous treatment** | | | | | | | | | | | | | |  |
|  |  |  |  |  | No |  | 0.0362 |  | 1 |  | | | | | | |  |
|  | | |  |  |  |  |  |  |  |  | | | | | | | |
|  | | | | | | | | | | | | 1 |  | BV |  | 15/01/2020 4:54 AM |  |
|  | | | And this has been a long journey for me. You know, I had my operation about – seven years ago and over that time, you know, I’ve had operation, I’ve had chemo, I’ve had hormone treatment, and, and – you know, I’ve been very positive about doing things, you know, lots of exercise. Doing lots of things to, you know, to, to challenge the cancer but I have to say, I came away last time thinking, … you know, is, you know, is the end stage looming? | | | | | | | | | | | | | |  |
|  | | |  | | | | | | | | | | | | | |  |
|  | | | **Codes\\needing more support** | | | | | | | | | | | | | |  |
|  |  |  |  |  | No |  | 0.0220 |  | 1 |  | | | | | | |  |
|  | | |  |  |  |  |  |  |  |  | | | | | | | |
|  | | | | | | | | | | | | 1 |  | BV |  | 14/01/2020 1:28 PM |  |
|  | | | Not, not in terms of support, no. As I said, I, I think I’m pretty good so far. I mean, you know, things change of course. … (Inaudible) I’d have to say. But no, at the moment I don’t foresee anything that – where I – any … (Inaudible) sort of external support I need. | | | | | | | | | | | | | |  |
|  | | |  | | | | | | | | | | | | | |  |
|  | | | | | | | | | | | | | | | | | |
| Formatted Reports\\Coding Summary by File Formatted Report | | | | | | | | | | | Page 74 of 143 | | | | | | |
| 6/10/2022 8:14 AM | | | | | | | | | | | | | | | | | |
|  | | | **Classification** |  | **Aggregate** |  | **Coverage** |  | **Number Of Coding References** |  | | **Reference Number** |  | **Coded By Initials** |  | **Modified On** |  |
|  | | | **Codes\\needing more support\no additional support required** | | | | | | | | | | | | | |  |
|  |  |  |  |  | No |  | 0.0252 |  | 2 |  | | | | | | |  |
|  | | |  |  |  |  |  |  |  |  | | | | | | | |
|  | | | | | | | | | | | | 1 |  | BV |  | 14/01/2020 1:23 PM |  |
|  | | | I don’t think so, not so far. No. No. | | | | | | | | | | | | | |  |
|  | | |  | | | | | | | | | | | | | |  |
|  | | | | | | | | | | | | 2 |  | BV |  | 14/01/2020 1:28 PM |  |
|  | | | Not, not in terms of support, no. As I said, I, I think I’m pretty good so far. I mean, you know, things change of course. … (Inaudible) I’d have to say. But no, at the moment I don’t foresee anything that – where I – any … (Inaudible) sort of external support I need. | | | | | | | | | | | | | |  |
|  | | |  | | | | | | | | | | | | | |  |
|  | | | **Codes\\PSA numbers not going down** | | | | | | | | | | | | | |  |
|  |  |  |  |  | No |  | 0.1679 |  | 5 |  | | | | | | |  |
|  | | |  |  |  |  |  |  |  |  | | | | | | | |
|  | | | | | | | | | | | | 1 |  | BV |  | 15/01/2020 4:58 AM |  |
|  | | | From a coping point of view, no problems but the trial – the first few – couple of cycles at the trial, they – we did seem to be making in roads into bringing my PSA down, but the last couple of readings we’ve had the PSA seems to have levelled off. So, that – I was a bit – I have to say, I’ve been a bit disappointed about that, you know, I was, you know, I was hoping that my PSA would be coming right back down. | | | | | | | | | | | | | |  |
|  | | |  | | | | | | | | | | | | | |  |
|  | | | | | | | | | | | | 2 |  | BV |  | 14/01/2020 1:17 PM |  |
|  | | | I had, you know, I had, I had treatment with a drug called … and – many years ago; soon after I had my operation and, and that brought my PSA levels back down to a sort of almost rock bottom. But … hasn’t done that, so far. Now, I don’t know if things will change but at the moment – after my last consultation with the specialist, I felt as though we were perhaps at the point now where we were keeping the cancer in check but not bringing it down or, or, you know, eradicating it. And so, I came away from the last consultation, which was about nine or ten days ago, feeling a bit down about that and starting to think, well, you know, it’s – what else – what, what’s next sort of – what can we do next? | | | | | | | | | | | | | |  |
|  | | |  | | | | | | | | | | | | | |  |
|  | | | | | | | | | | | | 3 |  | BV |  | 15/01/2020 5:21 AM |  |
|  | | | I mean, you know, my, my only wish is that, you know, is that next time I get to have my PSA checked, it might have dropped a bit further.  Q: Yeah, I’m hoping that for you as well.  A: Yeah, thank you.  Q: Definitely.  A: Otherwise, you know, it’s, you know, I think the time will come … even perhaps before the end of the trial when …, you know, in conjunction with the consultant, we have to think about maybe a plan B going forward. | | | | | | | | | | | | | |  |
|  |  |  |  |  |  |  |  |  |  |  |  |  |  |  |  |  |  |
|  | | |  | | | | | | | | | | | | | |  |
|  | | | | | | | | | | | | 4 |  | BV |  | 14/01/2020 1:30 PM |  |
|  | | | just to mention, yeah, I just … it wasn’t just the, the effects of the PSA levelling off, it was also I’d had … the mid, mid-trial scans or the eleven week scans and that actually showed that the, you know, the cancer had stopped spreading; largely stopped spreading but hadn’t reduced. So, that was also I think contributing to, you know, … my afterwards feeling a bit down about actually the, the effects of the treatment. You know, … whether it was- | | | | | | | | | | | | | |  |
|  | | |  | | | | | | | | | | | | | |  |
|  | | | | | | | | | | | | 5 |  | BV |  | 14/01/2020 1:33 PM |  |
|  | | | Yeah, and I’ll keep my own fingers crossed | | | | | | | | | | | | | |  |
|  | | |  | | | | | | | | | | | | | |  |
|  | | | | | | | | | | | | | | | | | |
| Formatted Reports\\Coding Summary by File Formatted Report | | | | | | | | | | | Page 75 of 143 | | | | | | |
| 6/10/2022 8:14 AM | | | | | | | | | | | | | | | | | |
|  | | | **Classification** |  | **Aggregate** |  | **Coverage** |  | **Number Of Coding References** |  | | **Reference Number** |  | **Coded By Initials** |  | **Modified On** |  |
|  | | | **Codes\\PSA numbers not going down\PSA numbers not going down - cabazitaxel** | | | | | | | | | | | | | |  |
|  |  |  |  |  | No |  | 0.1679 |  | 5 |  | | | | | | |  |
|  | | |  |  |  |  |  |  |  |  | | | | | | | |
|  | | | | | | | | | | | | 1 |  | BV |  | 25/03/2020 8:21 AM |  |
|  | | | From a coping point of view, no problems but the trial – the first few – couple of cycles at the trial, they – we did seem to be making in roads into bringing my PSA down, but the last couple of readings we’ve had the PSA seems to have levelled off. So, that – I was a bit – I have to say, I’ve been a bit disappointed about that, you know, I was, you know, I was hoping that my PSA would be coming right back down. | | | | | | | | | | | | | |  |
|  | | |  | | | | | | | | | | | | | |  |
|  | | | | | | | | | | | | 2 |  | BV |  | 25/03/2020 8:21 AM |  |
|  | | | I had, you know, I had, I had treatment with a drug called … and – many years ago; soon after I had my operation and, and that brought my PSA levels back down to a sort of almost rock bottom. But … hasn’t done that, so far. Now, I don’t know if things will change but at the moment – after my last consultation with the specialist, I felt as though we were perhaps at the point now where we were keeping the cancer in check but not bringing it down or, or, you know, eradicating it. And so, I came away from the last consultation, which was about nine or ten days ago, feeling a bit down about that and starting to think, well, you know, it’s – what else – what, what’s next sort of – what can we do next? | | | | | | | | | | | | | |  |
|  | | |  | | | | | | | | | | | | | |  |
|  | | | | | | | | | | | | 3 |  | BV |  | 25/03/2020 8:21 AM |  |
|  | | | I mean, you know, my, my only wish is that, you know, is that next time I get to have my PSA checked, it might have dropped a bit further.  Q: Yeah, I’m hoping that for you as well.  A: Yeah, thank you.  Q: Definitely.  A: Otherwise, you know, it’s, you know, I think the time will come … even perhaps before the end of the trial when …, you know, in conjunction with the consultant, we have to think about maybe a plan B going forward. | | | | | | | | | | | | | |  |
|  |  |  |  |  |  |  |  |  |  |  |  |  |  |  |  |  |  |
|  | | |  | | | | | | | | | | | | | |  |
|  | | | | | | | | | | | | 4 |  | BV |  | 25/03/2020 8:21 AM |  |
|  | | | just to mention, yeah, I just … it wasn’t just the, the effects of the PSA levelling off, it was also I’d had … the mid, mid-trial scans or the eleven week scans and that actually showed that the, you know, the cancer had stopped spreading; largely stopped spreading but hadn’t reduced. So, that was also I think contributing to, you know, … my afterwards feeling a bit down about actually the, the effects of the treatment. You know, … whether it was- | | | | | | | | | | | | | |  |
|  | | |  | | | | | | | | | | | | | |  |
|  | | | | | | | | | | | | 5 |  | BV |  | 25/03/2020 8:21 AM |  |
|  | | | Yeah, and I’ll keep my own fingers crossed | | | | | | | | | | | | | |  |
|  | | |  | | | | | | | | | | | | | |  |
|  | | | **Codes\\quality of life changes** | | | | | | | | | | | | | |  |
|  |  |  |  |  | No |  | 0.0908 |  | 4 |  | | | | | | |  |
|  | | |  |  |  |  |  |  |  |  | | | | | | | |
|  | | | | | | | | | | | | 1 |  | BV |  | 25/03/2020 8:25 AM |  |
|  | | | no as – you know, I haven’t got as much energy as I had before I went on the trial, but, but – not stopping me doing things, it just means I, you know, maybe need to rest a little bit more than I used to but yeah, no, so that’s been going well. And in terms of the sickness and stuff like that, no, no real problem. I mean, taste has been a problem- | | | | | | | | | | | | | |  |
|  | | |  | | | | | | | | | | | | | |  |
|  | | | | | | | | | | | | 2 |  | BV |  | 25/03/2020 8:25 AM |  |
|  | | | Each cycle, probably for a good week at least, I’ve had, you know, a grotty taste in my mouth and some foods therefore have not tasted as, as enjoyable as I remember them being. But apart from that, fatigue and taste, that’s about it. | | | | | | | | | | | | | |  |
|  | | |  | | | | | | | | | | | | | |  |
|  | | | | | | | | | | | | 3 |  | BV |  | 25/03/2020 8:25 AM |  |
|  | | | Not really. No, I mean I was – because my illness had accelerated a bit, before I went on the trial, you know, I, I was sort of slowing down a bit before I went on the trial and, and it – certainly being on the trial hasn’t really had a huge – made a huge change to that – to the effect that that was having on my lifestyle. | | | | | | | | | | | | | |  |
|  | | |  | | | | | | | | | | | | | |  |
| Formatted Reports\\Coding Summary by File Formatted Report | | | | | | | | | | | Page 76 of 143 | | | | | | |
| 6/10/2022 8:14 AM | | | | | | | | | | | | | | | | | |
|  | | | **Classification** |  | **Aggregate** |  | **Coverage** |  | **Number Of Coding References** |  | | **Reference Number** |  | **Coded By Initials** |  | **Modified On** |  |
|  | | | | | | | | | | | | | | | | | |
|  | | | | | | | | | | | | 4 |  | BV |  | 25/03/2020 8:25 AM |  |
|  | | | I’m having a pretty regular lifestyle. I mean, apart from feeling a bit tired and a bit, bit yucky at times; taste wise and stomach wise. … no, I mean, you know, life’s pretty much, you know, fine really. | | | | | | | | | | | | | |  |
|  | | |  | | | | | | | | | | | | | |  |
|  | | | **Codes\\quality of life changes\quality of life changes - cabazitaxel** | | | | | | | | | | | | | |  |
|  |  |  |  |  | No |  | 0.0911 |  | 4 |  | | | | | | |  |
|  | | |  |  |  |  |  |  |  |  | | | | | | | |
|  | | | | | | | | | | | | 1 |  | BV |  | 14/01/2020 1:14 PM |  |
|  | | | no as – you know, I haven’t got as much energy as I had before I went on the trial, but, but – not stopping me doing things, it just means I, you know, maybe need to rest a little bit more than I used to but yeah, no, so that’s been going well. And in terms of the sickness and stuff like that, no, no real problem. I mean, taste has been a problem- | | | | | | | | | | | | | |  |
|  | | |  | | | | | | | | | | | | | |  |
|  | | | | | | | | | | | | 2 |  | BV |  | 14/01/2020 1:14 PM |  |
|  | | | Each cycle, probably for a good week at least, I’ve had, you know, a grotty taste in my mouth and some foods therefore have not tasted as, as enjoyable as I remember them being. But apart from that, fatigue and taste, that’s about it. | | | | | | | | | | | | | |  |
|  | | |  | | | | | | | | | | | | | |  |
|  | | | | | | | | | | | | 3 |  | BV |  | 14/01/2020 1:14 PM |  |
|  | | | Not really. No, I mean I was – because my illness had accelerated a bit, before I went on the trial, you know, I, I was sort of slowing down a bit before I went on the trial and, and it – certainly being on the trial hasn’t really had a huge – made a huge change to that – to the effect that that was having on my lifestyle. | | | | | | | | | | | | | |  |
|  | | |  | | | | | | | | | | | | | |  |
|  | | | | | | | | | | | | 4 |  | BV |  | 14/01/2020 1:23 PM |  |
|  | | | I’m having a pretty regular lifestyle. I mean, apart from feeling a bit tired and a bit, bit yucky at times; taste wise and stomach wise. … no, I mean, you know, life’s pretty much, you know, fine really. | | | | | | | | | | | | | |  |
|  | | |  | | | | | | | | | | | | | |  |
|  | | | **Codes\\quality of trialists** | | | | | | | | | | | | | |  |
|  |  |  |  |  | No |  | 0.0378 |  | 2 |  | | | | | | |  |
|  | | |  |  |  |  |  |  |  |  | | | | | | | |
|  | | | | | | | | | | | | 1 |  | BV |  | 14/01/2020 1:21 PM |  |
|  | | | A: I found all of the staff at the Royal Brisbane, really, really helpful. Enthusiastic. They’ve got plenty of time for the patient. Really, I – I can’t speak highly enough about the, you know, the, the people there. They do, you know, they’re all doing a great job. It doesn’t matter, you know, which nurse I get, on which particular day. You know, they’re all, they’re all very committed; very friendly and helpful and informative. | | | | | | | | | | | | | |  |
|  | | |  | | | | | | | | | | | | | |  |
|  | | | | | | | | | | | | 2 |  | BV |  | 14/01/2020 1:21 PM |  |
|  | | | - really good, really good. | | | | | | | | | | | | | |  |
|  | | |  | | | | | | | | | | | | | |  |
|  | | | **Codes\\side effects** | | | | | | | | | | | | | |  |
|  |  |  |  |  | No |  | 0.1211 |  | 5 |  | | | | | | |  |
|  | | |  |  |  |  |  |  |  |  | | | | | | | |
|  | | | | | | | | | | | | 1 |  | BV |  | 25/03/2020 8:26 AM |  |
|  | | | Yes. Yeah, it’s pretty good, actually, in terms of, of side effects. I haven’t really – you know, when I, when I first started off on the treatment, I was warned that there could be all sorts of side effects associated with it and the drugs. But I’ve so far; nearly halfway through, I’m – it’s, it’s been going quite well, you know, with the – yeah, no, no real problems. Fatigue has been a bit of a problem- | | | | | | | | | | | | | |  |
|  | | |  | | | | | | | | | | | | | |  |
| Formatted Reports\\Coding Summary by File Formatted Report | | | | | | | | | | | Page 77 of 143 | | | | | | |
| 6/10/2022 8:14 AM | | | | | | | | | | | | | | | | | |
|  | | | **Classification** |  | **Aggregate** |  | **Coverage** |  | **Number Of Coding References** |  | | **Reference Number** |  | **Coded By Initials** |  | **Modified On** |  |
|  | | | | | | | | | | | | | | | | | |
|  | | | | | | | | | | | | 2 |  | BV |  | 25/03/2020 8:26 AM |  |
|  | | | no as – you know, I haven’t got as much energy as I had before I went on the trial, but, but – not stopping me doing things, it just means I, you know, maybe need to rest a little bit more than I used to but yeah, no, so that’s been going well. And in terms of the sickness and stuff like that, no, no real problem. I mean, taste has been a problem- | | | | | | | | | | | | | |  |
|  | | |  | | | | | | | | | | | | | |  |
|  | | | | | | | | | | | | 3 |  | BV |  | 25/03/2020 8:26 AM |  |
|  | | | Each cycle, probably for a good week at least, I’ve had, you know, a grotty taste in my mouth and some foods therefore have not tasted as, as enjoyable as I remember them being. But apart from that, fatigue and taste, that’s about it. | | | | | | | | | | | | | |  |
|  | | |  | | | | | | | | | | | | | |  |
|  | | | | | | | | | | | | 4 |  | BV |  | 25/03/2020 8:26 AM |  |
|  | | | I’m having a pretty regular lifestyle. I mean, apart from feeling a bit tired and a bit, bit yucky at times; taste wise and stomach wise. … no, I mean, you know, life’s pretty much, you know, fine really. | | | | | | | | | | | | | |  |
|  | | |  | | | | | | | | | | | | | |  |
|  | | | | | | | | | | | | 5 |  | BV |  | 25/03/2020 8:26 AM |  |
|  | | | just to mention, yeah, I just … it wasn’t just the, the effects of the PSA levelling off, it was also I’d had … the mid, mid-trial scans or the eleven week scans and that actually showed that the, you know, the cancer had stopped spreading; largely stopped spreading but hadn’t reduced. | | | | | | | | | | | | | |  |
|  | | |  | | | | | | | | | | | | | |  |
|  | | | **Codes\\side effects\side effects - cabazitaxel** | | | | | | | | | | | | | |  |
|  |  |  |  |  | No |  | 0.1214 |  | 5 |  | | | | | | |  |
|  | | |  |  |  |  |  |  |  |  | | | | | | | |
|  | | | | | | | | | | | | 1 |  | BV |  | 14/01/2020 1:14 PM |  |
|  | | | Yes. Yeah, it’s pretty good, actually, in terms of, of side effects. I haven’t really – you know, when I, when I first started off on the treatment, I was warned that there could be all sorts of side effects associated with it and the drugs. But I’ve so far; nearly halfway through, I’m – it’s, it’s been going quite well, you know, with the – yeah, no, no real problems. Fatigue has been a bit of a problem- | | | | | | | | | | | | | |  |
|  | | |  | | | | | | | | | | | | | |  |
|  | | | | | | | | | | | | 2 |  | BV |  | 14/01/2020 1:14 PM |  |
|  | | | no as – you know, I haven’t got as much energy as I had before I went on the trial, but, but – not stopping me doing things, it just means I, you know, maybe need to rest a little bit more than I used to but yeah, no, so that’s been going well. And in terms of the sickness and stuff like that, no, no real problem. I mean, taste has been a problem- | | | | | | | | | | | | | |  |
|  | | |  | | | | | | | | | | | | | |  |
|  | | | | | | | | | | | | 3 |  | BV |  | 14/01/2020 1:14 PM |  |
|  | | | Each cycle, probably for a good week at least, I’ve had, you know, a grotty taste in my mouth and some foods therefore have not tasted as, as enjoyable as I remember them being. But apart from that, fatigue and taste, that’s about it. | | | | | | | | | | | | | |  |
|  | | |  | | | | | | | | | | | | | |  |
|  | | | | | | | | | | | | 4 |  | BV |  | 14/01/2020 1:23 PM |  |
|  | | | I’m having a pretty regular lifestyle. I mean, apart from feeling a bit tired and a bit, bit yucky at times; taste wise and stomach wise. … no, I mean, you know, life’s pretty much, you know, fine really. | | | | | | | | | | | | | |  |
|  | | |  | | | | | | | | | | | | | |  |
|  | | | | | | | | | | | | 5 |  | BV |  | 14/01/2020 1:30 PM |  |
|  | | | just to mention, yeah, I just … it wasn’t just the, the effects of the PSA levelling off, it was also I’d had … the mid, mid-trial scans or the eleven week scans and that actually showed that the, you know, the cancer had stopped spreading; largely stopped spreading but hadn’t reduced. | | | | | | | | | | | | | |  |
|  | | |  | | | | | | | | | | | | | |  |
|  | | | **Codes\\staying positive** | | | | | | | | | | | | | |  |
|  |  |  |  |  | No |  | 0.0629 |  | 2 |  | | | | | | |  |
|  | | |  |  |  |  |  |  |  |  | | | | | | | |
|  | | | | | | | | | | | | 1 |  | BV |  | 15/01/2020 4:56 AM |  |
|  | | | And this has been a long journey for me. You know, I had my operation about – seven years ago and over that time, you know, I’ve had operation, I’ve had chemo, I’ve had hormone treatment, and, and – you know, I’ve been very positive about doing things, you know, lots of exercise. Doing lots of things to, you know, to, to challenge the cancer but I have to say, I came away last time thinking, … you know, is, you know, is the end stage looming? | | | | | | | | | | | | | |  |
|  | | |  | | | | | | | | | | | | | |  |
| Formatted Reports\\Coding Summary by File Formatted Report | | | | | | | | | | | Page 78 of 143 | | | | | | |
| 6/10/2022 8:14 AM | | | | | | | | | | | | | | | | | |
|  | | | **Classification** |  | **Aggregate** |  | **Coverage** |  | **Number Of Coding References** |  | | **Reference Number** |  | **Coded By Initials** |  | **Modified On** |  |
|  | | | | | | | | | | | | | | | | | |
|  | | | | | | | | | | | | 2 |  | BV |  | 14/01/2020 1:20 PM |  |
|  | | | yeah, no, I did, you know, in my consultation with the consultant …, you know, … the trials and … (Inaudible) if this proves to be. …, you know, it … ineffective going forward, you know, the treatment I’m on … that looks as though it’s going to be … that there are other things we can try but – so, no, he was, he was positive- | | | | | | | | | | | | | |  |
|  | | |  | | | | | | | | | | | | | |  |
|  | | | **Codes\\support from family** | | | | | | | | | | | | | |  |
|  |  |  |  |  | No |  | 0.0067 |  | 1 |  | | | | | | |  |
|  | | |  |  |  |  |  |  |  |  | | | | | | | |
|  | | | | | | | | | | | | 1 |  | BV |  | 14/01/2020 1:29 PM |  |
|  | | | You know, I’ve got the support of my wife and my family and, and, and that’s fine. | | | | | | | | | | | | | |  |
|  | | |  | | | | | | | | | | | | | |  |
|  | | | **Codes\\support from partner** | | | | | | | | | | | | | |  |
|  |  |  |  |  | No |  | 0.0067 |  | 1 |  | | | | | | |  |
|  | | |  |  |  |  |  |  |  |  | | | | | | | |
|  | | | | | | | | | | | | 1 |  | BV |  | 15/01/2020 5:31 AM |  |
|  | | | You know, I’ve got the support of my wife and my family and, and, and that’s fine. | | | | | | | | | | | | | |  |
|  | | |  | | | | | | | | | | | | | |  |
|  | | | **Codes\\treatments left** | | | | | | | | | | | | | |  |
|  |  |  |  |  | No |  | 0.0052 |  | 1 |  | | | | | | |  |
|  | | |  |  |  |  |  |  |  |  | | | | | | | |
|  | | | | | | | | | | | | 1 |  | BV |  | 14/01/2020 1:18 PM |  |
|  | | | Q: How many treatments have you had?  A: I’ve had five now. | | | | | | | | | | | | | |  |
|  | | |  | | | | | | | | | | | | | |  |
|  | | | **Codes\\uncertainty of the future** | | | | | | | | | | | | | |  |
|  |  |  |  |  | No |  | 0.1590 |  | 4 |  | | | | | | |  |
|  | | |  |  |  |  |  |  |  |  | | | | | | | |
|  | | | | | | | | | | | | 1 |  | BV |  | 14/01/2020 1:19 PM |  |
|  | | | You know, I had my operation about – seven years ago and over that time, you know, I’ve had operation, I’ve had chemo, I’ve had hormone treatment, and, and – you know, I’ve been very positive about doing things, you know, lots of exercise. Doing lots of things to, you know, to, to challenge the cancer but I have to say, I came away last time thinking, … you know, is, you know, is the end stage looming? Now, I don’t mean to be alarmist. I mean I’m not expecting that to be, you know, any time quite soon, but, you know, am I getting to the - … starting to wonder whether I was getting to the stage in my treatment where, you know, maybe, maybe, …, you know, the finish line is, is out there and in the not too distant future. | | | | | | | | | | | | | |  |
|  | | |  | | | | | | | | | | | | | |  |
|  | | | | | | | | | | | | 2 |  | BV |  | 14/01/2020 1:20 PM |  |
|  | | | yeah, no, I did, you know, in my consultation with the consultant …, you know, … the trials and … (Inaudible) if this proves to be. …, you know, it … ineffective going forward, you know, the treatment I’m on … that looks as though it’s going to be … that there are other things we can try but – so, no, he was, he was positive- | | | | | | | | | | | | | |  |
|  | | |  | | | | | | | | | | | | | |  |
|  | | | | | | | | | | | | | | | | | |
| Formatted Reports\\Coding Summary by File Formatted Report | | | | | | | | | | | Page 79 of 143 | | | | | | |
| 6/10/2022 8:14 AM | | | | | | | | | | | | | | | | | |
|  | | | **Classification** |  | **Aggregate** |  | **Coverage** |  | **Number Of Coding References** |  | | **Reference Number** |  | **Coded By Initials** |  | **Modified On** |  |
|  | | | | | | | | | | | | | | | | | |
|  | | | | | | | | | | | | 3 |  | BV |  | 15/01/2020 5:21 AM |  |
|  | | | I mean, you know, my, my only wish is that, you know, is that next time I get to have my PSA checked, it might have dropped a bit further.  Q: Yeah, I’m hoping that for you as well.  A: Yeah, thank you.  Q: Definitely.  A: Otherwise, you know, it’s, you know, I think the time will come … even perhaps before the end of the trial when …, you know, in conjunction with the consultant, we have to think about maybe a plan B going forward. | | | | | | | | | | | | | |  |
|  |  |  |  |  |  |  |  |  |  |  |  |  |  |  |  |  |  |
|  | | |  | | | | | | | | | | | | | |  |
|  | | | | | | | | | | | | 4 |  | BV |  | 14/01/2020 1:30 PM |  |
|  | | | just to mention, yeah, I just … it wasn’t just the, the effects of the PSA levelling off, it was also I’d had … the mid, mid-trial scans or the eleven week scans and that actually showed that the, you know, the cancer had stopped spreading; largely stopped spreading but hadn’t reduced. So, that was also I think contributing to, you know, … my afterwards feeling a bit down about actually the, the effects of the treatment. You know, … whether it was- | | | | | | | | | | | | | |  |
|  | | |  | | | | | | | | | | | | | |  |
|  | | | **Codes\\waiting for doctors** | | | | | | | | | | | | | |  |
|  |  |  |  |  | No |  | 0.0538 |  | 2 |  | | | | | | |  |
|  | | |  |  |  |  |  |  |  |  | | | | | | | |
|  | | | | | | | | | | | | 1 |  | BV |  | 15/01/2020 5:32 AM |  |
|  | | | No, I think the only thing is; and it wouldn’t just be me saying this, I think it’s everybody going there, it can turn out to be a long day. You’re not sure how long things take. You’re not sure if people are going to react to drugs and therefore take longer to have their treatment, and therefore … you know, in the waiting list. | | | | | | | | | | | | | |  |
|  | | |  | | | | | | | | | | | | | |  |
|  | | | | | | | | | | | | 2 |  | BV |  | 15/01/2020 5:32 AM |  |
|  | | | So, it can be, you know, it’s, it’s pretty much the best part of a day taken up, having the treatment but, you know, we just – my wife and I – my wife always comes along with me and we just sort of, you know, go there thinking that, you know, it, it’ll finish when it finishes. And we go prepared with books and, and things to do. | | | | | | | | | | | | | |  |
|  | | |  | | | | | | | | | | | | | |  |
|  | | | **Codes\\wanting to contribute to research** | | | | | | | | | | | | | |  |
|  |  |  |  |  | No |  | 0.0263 |  | 2 |  | | | | | | |  |
|  | | |  |  |  |  |  |  |  |  | | | | | | | |
|  | | | | | | | | | | | | 1 |  | BV |  | 15/01/2020 5:23 AM |  |
|  | | | I think I mentioned to you last time that, you know, I, you know, I … I’m benefiting by medical research, and, and medical research needs guinea pigs, if you like. | | | | | | | | | | | | | |  |
|  | | |  | | | | | | | | | | | | | |  |
|  | | | | | | | | | | | | 2 |  | BV |  | 15/01/2020 5:11 AM |  |
|  | | | And you know, I feel as though, you know, I’m contributing a bit to, to medical science research …-  Q: Yeah.  A: And no, it’s pleasing to be able to do that. | | | | | | | | | | | | | |  |
|  | | |  | | | | | | | | | | | | | |  |
|  | | | | | | | | | | | | | | | | | |
| Formatted Reports\\Coding Summary by File Formatted Report | | | | | | | | | | | Page 80 of 143 | | | | | | |
| 6/10/2022 8:14 AM | | | | | | | | | | | | | | | | | |
|  | | | **Classification** |  | **Aggregate** |  | **Coverage** |  | **Number Of Coding References** |  | | **Reference Number** |  | **Coded By Initials** |  | **Modified On** |  |
|  | **Files\\Transcription 3_002** | | | | | | | | | | | | | | | |  |
|  | | **Code** | | | | | | | | | | | | | | |  |
|  | | | **Codes\\coping strategies\coping okay** | | | | | | | | | | | | | |  |
|  |  |  |  |  | No |  | 0.0161 |  | 1 |  | | | | | | |  |
|  | | |  |  |  |  |  |  |  |  | | | | | | | |
|  | | | | | | | | | | | | 1 |  | BV |  | 14/01/2020 1:45 PM |  |
|  | | | Has the trial affected you emotionally or from a coping point of view?  A: No. | | | | | | | | | | | | | |  |
|  | | |  | | | | | | | | | | | | | |  |
|  | | | **Codes\\doctor happy with progress** | | | | | | | | | | | | | |  |
|  |  |  |  |  | No |  | 0.0348 |  | 1 |  | | | | | | |  |
|  | | |  |  |  |  |  |  |  |  | | | | | | | |
|  | | | | | | | | | | | | 1 |  | BV |  | 14/01/2020 1:47 PM |  |
|  | | | Q: Great, well that’s excellent. I’m glad things seem – well, they sound like they’re progressing well.  A: Yeah, well they seem to think so and I seem to think so, so. | | | | | | | | | | | | | |  |
|  | | |  | | | | | | | | | | | | | |  |
|  | | | **Codes\\financial impact\no affect to finances** | | | | | | | | | | | | | |  |
|  |  |  |  |  | No |  | 0.0161 |  | 1 |  | | | | | | |  |
|  | | |  |  |  |  |  |  |  |  | | | | | | | |
|  | | | | | | | | | | | | 1 |  | BV |  | 14/01/2020 1:45 PM |  |
|  | | | Q: Okay, and any financial or practical implications of participating?  A: No. | | | | | | | | | | | | | |  |
|  | | |  | | | | | | | | | | | | | |  |
|  | | | **Codes\\happy about participating** | | | | | | | | | | | | | |  |
|  |  |  |  |  | No |  | 0.0569 |  | 1 |  | | | | | | |  |
|  | | |  |  |  |  |  |  |  |  | | | | | | | |
|  | | | | | | | | | | | | 1 |  | BV |  | 14/01/2020 1:44 PM |  |
|  | | | Well, I feel, it’s good. It’s – I don’t suppose it’s ever going to last forever but that’s what the trial’s all about, to see how long it’s going to last and what side effects and all that sort of thing, so I’m quite happy with it actually, it’s given me another option and, yeah. | | | | | | | | | | | | | |  |
|  | | |  | | | | | | | | | | | | | |  |
|  | | | **Codes\\hope** | | | | | | | | | | | | | |  |
|  |  |  |  |  | No |  | 0.0626 |  | 2 |  | | | | | | |  |
|  | | |  |  |  |  |  |  |  |  | | | | | | | |
|  | | | | | | | | | | | | 1 |  | BV |  | 14/01/2020 1:42 PM |  |
|  | | | Well, it’s – I can’t say it’s a good experience, it’s an experience and, what can I say, it seems to be doing what they hoped it would do, so yeah, experience …. | | | | | | | | | | | | | |  |
|  | | |  | | | | | | | | | | | | | |  |
|  | | | | | | | | | | | | 2 |  | BV |  | 14/01/2020 1:46 PM |  |
|  | | | I just hope that this lasts a long time and then there’s something else around the corner – might be something else around the corner, so who knows. | | | | | | | | | | | | | |  |
|  | | |  | | | | | | | | | | | | | |  |
| Formatted Reports\\Coding Summary by File Formatted Report | | | | | | | | | | | Page 81 of 143 | | | | | | |
| 6/10/2022 8:14 AM | | | | | | | | | | | | | | | | | |
|  | | | **Classification** |  | **Aggregate** |  | **Coverage** |  | **Number Of Coding References** |  | | **Reference Number** |  | **Coded By Initials** |  | **Modified On** |  |
|  | | | **Codes\\hope\hoping treatment lasts** | | | | | | | | | | | | | |  |
|  |  |  |  |  | No |  | 0.0297 |  | 1 |  | | | | | | |  |
|  | | |  |  |  |  |  |  |  |  | | | | | | | |
|  | | | | | | | | | | | | 1 |  | BV |  | 15/01/2020 9:56 AM |  |
|  | | | I just hope that this lasts a long time and then there’s something else around the corner – might be something else around the corner, so who knows. | | | | | | | | | | | | | |  |
|  | | |  | | | | | | | | | | | | | |  |
|  | | | **Codes\\initial motivation for participation** | | | | | | | | | | | | | |  |
|  |  |  |  |  | No |  | 0.0865 |  | 1 |  | | | | | | |  |
|  | | |  |  |  |  |  |  |  |  | | | | | | | |
|  | | | | | | | | | | | | 1 |  | BV |  | 14/01/2020 1:45 PM |  |
|  | | | Yeah, okay, my initial motivations, after chemo and after the next lot of medication when the PSA kept getting up, motivation was the oncologist said, well, maybe you can have more chemo which would be …, but I’d been looking on the internet and I found this, the trial, so I just asked him, and that was my motivation to – I’ll have another thing up my sleeve if, when this stops working … (Inaudible), that was the motivation. | | | | | | | | | | | | | |  |
|  | | |  | | | | | | | | | | | | | |  |
|  | | | **Codes\\initial motivation for participation\changes to initial motivation for participation** | | | | | | | | | | | | | |  |
|  |  |  |  |  | No |  | 0.0438 |  | 1 |  | | | | | | |  |
|  | | |  |  |  |  |  |  |  |  | | | | | | | |
|  | | | | | | | | | | | | 1 |  | BV |  | 14/01/2020 1:45 PM |  |
|  | | | Q: And I’m assuming it’s still the same motivation?  A: Of course. I just hope that this lasts a long time and then there’s something else around the corner – might be something else around the corner, so who knows. | | | | | | | | | | | | | |  |
|  | | |  | | | | | | | | | | | | | |  |
|  | | | **Codes\\initial motivation for participation\doctors recommendation** | | | | | | | | | | | | | |  |
|  |  |  |  |  | No |  | 0.0865 |  | 1 |  | | | | | | |  |
|  | | |  |  |  |  |  |  |  |  | | | | | | | |
|  | | | | | | | | | | | | 1 |  | BV |  | 14/01/2020 1:45 PM |  |
|  | | | Yeah, okay, my initial motivations, after chemo and after the next lot of medication when the PSA kept getting up, motivation was the oncologist said, well, maybe you can have more chemo which would be …, but I’d been looking on the internet and I found this, the trial, so I just asked him, and that was my motivation to – I’ll have another thing up my sleeve if, when this stops working … (Inaudible), that was the motivation. | | | | | | | | | | | | | |  |
|  | | |  | | | | | | | | | | | | | |  |
|  | | | **Codes\\lutetium provided another option** | | | | | | | | | | | | | |  |
|  |  |  |  |  | No |  | 0.0569 |  | 1 |  | | | | | | |  |
|  | | |  |  |  |  |  |  |  |  | | | | | | | |
|  | | | | | | | | | | | | 1 |  | BV |  | 14/01/2020 1:44 PM |  |
|  | | | Well, I feel, it’s good. It’s – I don’t suppose it’s ever going to last forever but that’s what the trial’s all about, to see how long it’s going to last and what side effects and all that sort of thing, so I’m quite happy with it actually, it’s given me another option and, yeah. | | | | | | | | | | | | | |  |
|  | | |  | | | | | | | | | | | | | |  |
|  | | | | | | | | | | | | | | | | | |
| Formatted Reports\\Coding Summary by File Formatted Report | | | | | | | | | | | Page 82 of 143 | | | | | | |
| 6/10/2022 8:14 AM | | | | | | | | | | | | | | | | | |
|  | | | **Classification** |  | **Aggregate** |  | **Coverage** |  | **Number Of Coding References** |  | | **Reference Number** |  | **Coded By Initials** |  | **Modified On** |  |
|  | | | **Codes\\needing more support\no additional support required** | | | | | | | | | | | | | |  |
|  |  |  |  |  | No |  | 0.0338 |  | 1 |  | | | | | | |  |
|  | | |  |  |  |  |  |  |  |  | | | | | | | |
|  | | | | | | | | | | | | 1 |  | BV |  | 14/01/2020 1:45 PM |  |
|  | | | Alright. Is there any situations where you would have liked more support or care during the trial?  A: No, I can’t think of any, no. No, there’s definitely not, no. | | | | | | | | | | | | | |  |
|  | | |  | | | | | | | | | | | | | |  |
|  | | | **Codes\\PSA numbers going down** | | | | | | | | | | | | | |  |
|  |  |  |  |  | No |  | 0.0418 |  | 1 |  | | | | | | |  |
|  | | |  |  |  |  |  |  |  |  | | | | | | | |
|  | | | | | | | | | | | | 1 |  | BV |  | 15/01/2020 4:47 AM |  |
|  | | | Oh, yes, well there’s an improvement in my PSA, it’s gone – it’s down below 3, and well, when they show me the x-rays and that, the …, it’s all reducing, the cancer is – you can’t even see it in places now. | | | | | | | | | | | | | |  |
|  | | |  | | | | | | | | | | | | | |  |
|  | | | **Codes\\PSA numbers going down\PSA numbers going down - lutetium** | | | | | | | | | | | | | |  |
|  |  |  |  |  | No |  | 0.0414 |  | 1 |  | | | | | | |  |
|  | | |  |  |  |  |  |  |  |  | | | | | | | |
|  | | | | | | | | | | | | 1 |  | BV |  | 25/03/2020 8:22 AM |  |
|  | | | Oh, yes, well there’s an improvement in my PSA, it’s gone – it’s down below 3, and well, when they show me the x-rays and that, the …, it’s all reducing, the cancer is – you can’t even see it in places now. | | | | | | | | | | | | | |  |
|  | | |  | | | | | | | | | | | | | |  |
|  | | | **Codes\\quality of life changes** | | | | | | | | | | | | | |  |
|  |  |  |  |  | No |  | 0.0038 |  | 1 |  | | | | | | |  |
|  | | |  |  |  |  |  |  |  |  | | | | | | | |
|  | | | | | | | | | | | | 1 |  | BV |  | 25/03/2020 8:25 AM |  |
|  | | | No, not really, no. | | | | | | | | | | | | | |  |
|  | | |  | | | | | | | | | | | | | |  |
|  | | | **Codes\\quality of life changes\quality of life changes - lutetium** | | | | | | | | | | | | | |  |
|  |  |  |  |  | No |  | 0.0038 |  | 1 |  | | | | | | |  |
|  | | |  |  |  |  |  |  |  |  | | | | | | | |
|  | | | | | | | | | | | | 1 |  | BV |  | 14/01/2020 1:43 PM |  |
|  | | | No, not really, no. | | | | | | | | | | | | | |  |
|  | | |  | | | | | | | | | | | | | |  |
|  | | | **Codes\\quality of trialists** | | | | | | | | | | | | | |  |
|  |  |  |  |  | No |  | 0.0614 |  | 3 |  | | | | | | |  |
|  | | |  |  |  |  |  |  |  |  | | | | | | | |
|  | | | | | | | | | | | | 1 |  | BV |  | 14/01/2020 1:44 PM |  |
|  | | | do their job and there’s no hassles with anything. | | | | | | | | | | | | | |  |
|  | | |  | | | | | | | | | | | | | |  |
| Formatted Reports\\Coding Summary by File Formatted Report | | | | | | | | | | | Page 83 of 143 | | | | | | |
| 6/10/2022 8:14 AM | | | | | | | | | | | | | | | | | |
|  | | | **Classification** |  | **Aggregate** |  | **Coverage** |  | **Number Of Coding References** |  | | **Reference Number** |  | **Coded By Initials** |  | **Modified On** |  |
|  | | | | | | | | | | | | | | | | | |
|  | | | | | | | | | | | | 2 |  | BV |  | 14/01/2020 1:44 PM |  |
|  | | | They really – they helped me all along the way and … smile and, yeah. | | | | | | | | | | | | | |  |
|  | | |  | | | | | | | | | | | | | |  |
|  | | | | | | | | | | | | 3 |  | BV |  | 14/01/2020 1:47 PM |  |
|  | | | The last things I want to say, well, I’d like to thank everybody at the hospital, which I will be doing once the trial is finished, but it’s not going to be finished for quite a while. | | | | | | | | | | | | | |  |
|  | | |  | | | | | | | | | | | | | |  |
|  | | | **Codes\\side effects** | | | | | | | | | | | | | |  |
|  |  |  |  |  | No |  | 0.0350 |  | 1 |  | | | | | | |  |
|  | | |  |  |  |  |  |  |  |  | | | | | | | |
|  | | | | | | | | | | | | 1 |  | BV |  | 25/03/2020 8:26 AM |  |
|  | | | Oh, just dry mouth, I get a bit tired if I do too much, that’s it. Yeah, I don’t have any other symptoms really – a bit of groin pain but they’ve figured that out, so, yeah. | | | | | | | | | | | | | |  |
|  | | |  | | | | | | | | | | | | | |  |
|  | | | **Codes\\side effects\side effects - lutetium** | | | | | | | | | | | | | |  |
|  |  |  |  |  | No |  | 0.0350 |  | 1 |  | | | | | | |  |
|  | | |  |  |  |  |  |  |  |  | | | | | | | |
|  | | | | | | | | | | | | 1 |  | BV |  | 14/01/2020 1:43 PM |  |
|  | | | Oh, just dry mouth, I get a bit tired if I do too much, that’s it. Yeah, I don’t have any other symptoms really – a bit of groin pain but they’ve figured that out, so, yeah. | | | | | | | | | | | | | |  |
|  | | |  | | | | | | | | | | | | | |  |
|  | | | **Codes\\treatments been good** | | | | | | | | | | | | | |  |
|  |  |  |  |  | No |  | 0.0463 |  | 3 |  | | | | | | |  |
|  | | |  |  |  |  |  |  |  |  | | | | | | | |
|  | | | | | | | | | | | | 1 |  | BV |  | 14/01/2020 1:42 PM |  |
|  | | | Yeah, I’m doing okay, yeah. I’m okay, yes. | | | | | | | | | | | | | |  |
|  | | |  | | | | | | | | | | | | | |  |
|  | | | | | | | | | | | | 2 |  | BV |  | 14/01/2020 1:42 PM |  |
|  | | | Well, it’s – I can’t say it’s a good experience, it’s an experience and, what can I say, it seems to be doing what they hoped it would do, so yeah, experience …. | | | | | | | | | | | | | |  |
|  | | |  | | | | | | | | | | | | | |  |
|  | | | | | | | | | | | | 3 |  | BV |  | 15/01/2020 5:22 AM |  |
|  | | | Well, I feel, it’s good. | | | | | | | | | | | | | |  |
|  | | |  | | | | | | | | | | | | | |  |
|  | | | | | | | | | | | | | | | | | |
| Formatted Reports\\Coding Summary by File Formatted Report | | | | | | | | | | | Page 84 of 143 | | | | | | |
| 6/10/2022 8:14 AM | | | | | | | | | | | | | | | | | |
|  | | | **Classification** |  | **Aggregate** |  | **Coverage** |  | **Number Of Coding References** |  | | **Reference Number** |  | **Coded By Initials** |  | **Modified On** |  |
|  | | | **Codes\\treatments left** | | | | | | | | | | | | | |  |
|  |  |  |  |  | No |  | 0.0259 |  | 1 |  | | | | | | |  |
|  | | |  |  |  |  |  |  |  |  | | | | | | | |
|  | | | | | | | | | | | | 1 |  | BV |  | 14/01/2020 1:42 PM |  |
|  | | | Q: So have you finished completely on the trial or do you still have a couple more-  A: No, I’ve still got one treatment to go. | | | | | | | | | | | | | |  |
|  | | |  | | | | | | | | | | | | | |  |
|  | | | **Codes\\uncertainty of the future** | | | | | | | | | | | | | |  |
|  |  |  |  |  | No |  | 0.0297 |  | 1 |  | | | | | | |  |
|  | | |  |  |  |  |  |  |  |  | | | | | | | |
|  | | | | | | | | | | | | 1 |  | BV |  | 14/01/2020 1:46 PM |  |
|  | | | I just hope that this lasts a long time and then there’s something else around the corner – might be something else around the corner, so who knows. | | | | | | | | | | | | | |  |
|  | | |  | | | | | | | | | | | | | |  |
|  | | | **Codes\\unsure of how long treatment effects will last** | | | | | | | | | | | | | |  |
|  |  |  |  |  | No |  | 0.0867 |  | 2 |  | | | | | | |  |
|  | | |  |  |  |  |  |  |  |  | | | | | | | |
|  | | | | | | | | | | | | 1 |  | BV |  | 15/01/2020 5:22 AM |  |
|  | | | Well, I feel, it’s good. It’s – I don’t suppose it’s ever going to last forever but that’s what the trial’s all about, to see how long it’s going to last and what side effects and all that sort of thing, so I’m quite happy with it actually, it’s given me another option and, yeah. | | | | | | | | | | | | | |  |
|  | | |  | | | | | | | | | | | | | |  |
|  | | | | | | | | | | | | 2 |  | BV |  | 14/01/2020 1:46 PM |  |
|  | | | I just hope that this lasts a long time and then there’s something else around the corner – might be something else around the corner, so who knows. | | | | | | | | | | | | | |  |
|  | | |  | | | | | | | | | | | | | |  |
|  | **Files\\Transcription 3_003** | | | | | | | | | | | | | | | |  |
|  | | **Code** | | | | | | | | | | | | | | |  |
|  | | | **Codes\\emotional impact** | | | | | | | | | | | | | |  |
|  |  |  |  |  | No |  | 0.0302 |  | 1 |  | | | | | | |  |
|  | | |  |  |  |  |  |  |  |  | | | | | | | |
|  | | | | | | | | | | | | 1 |  | BV |  | 14/01/2020 2:45 PM |  |
|  | | | Not from the treatment as such. I suppose it’s – with the PSA level rising, it’s quite draining emotionally because the PSA is a good gauge of what’s happening and mine continues to go up and in a pretty quick fashion. So, that does sort of drain you emotionally. | | | | | | | | | | | | | |  |
|  | | |  | | | | | | | | | | | | | |  |
|  | | | | | | | | | | | | | | | | | |
| Formatted Reports\\Coding Summary by File Formatted Report | | | | | | | | | | | Page 85 of 143 | | | | | | |
| 6/10/2022 8:14 AM | | | | | | | | | | | | | | | | | |
|  | | | **Classification** |  | **Aggregate** |  | **Coverage** |  | **Number Of Coding References** |  | | **Reference Number** |  | **Coded By Initials** |  | **Modified On** |  |
|  | | | **Codes\\feeling disappointed** | | | | | | | | | | | | | |  |
|  |  |  |  |  | No |  | 0.0229 |  | 1 |  | | | | | | |  |
|  | | |  |  |  |  |  |  |  |  | | | | | | | |
|  | | | | | | | | | | | | 1 |  | BV |  | 14/01/2020 2:42 PM |  |
|  | | | A: Yeah. So, my PSA came all the way to 15. So, it had a considerable drop. It was doing really well.  Q: Yeah.  A: Yeah and then all of a sudden it just started to rise and it continued to rise. | | | | | | | | | | | | | |  |
|  | | |  | | | | | | | | | | | | | |  |
|  | | | **Codes\\feeling lucky** | | | | | | | | | | | | | |  |
|  |  |  |  |  | No |  | 0.0482 |  | 1 |  | | | | | | |  |
|  | | |  |  |  |  |  |  |  |  | | | | | | | |
|  | | | | | | | | | | | | 1 |  | BV |  | 14/01/2020 2:47 PM |  |
|  | | | I suppose I was lucky. When I was having the Docetaxel, that type of particular chemo, I was lucky, I had very few side effects apart from losing my hair and after – sort of the first couple of days after, I was sort of down a fair bit, quite tired and that but it picked up. But I didn’t have symptoms. I never vomited. I didn’t feel sick. All those sort of things, I was very lucky I didn’t get those sort of things. | | | | | | | | | | | | | |  |
|  | | |  | | | | | | | | | | | | | |  |
|  | | | **Codes\\financial impact\no affect to finances** | | | | | | | | | | | | | |  |
|  |  |  |  |  | No |  | 0.0064 |  | 1 |  | | | | | | |  |
|  | | |  |  |  |  |  |  |  |  | | | | | | | |
|  | | | | | | | | | | | | 1 |  | BV |  | 14/01/2020 2:46 PM |  |
|  | | | No. Not at all. I’ve got a good support from work here. | | | | | | | | | | | | | |  |
|  | | |  | | | | | | | | | | | | | |  |
|  | | | **Codes\\happy about participating** | | | | | | | | | | | | | |  |
|  |  |  |  |  | No |  | 0.0256 |  | 1 |  | | | | | | |  |
|  | | |  |  |  |  |  |  |  |  | | | | | | | |
|  | | | | | | | | | | | | 1 |  | BV |  | 14/01/2020 2:49 PM |  |
|  | | | Q: Well, that’s what I was just going to ask, if you were still glad that you decided to participate.  A: Oh yeah, definitely. Yep, definitely. Just sort of bit – I’m not sure why it’s stopped all of a sudden so to speak. | | | | | | | | | | | | | |  |
|  | | |  | | | | | | | | | | | | | |  |
|  | | | | | | | | | | | | | | | | | |
|  | | | | | | | | | | | | | | | | | |
|  | | | | | | | | | | | | | | | | | |
| Formatted Reports\\Coding Summary by File Formatted Report | | | | | | | | | | | Page 86 of 143 | | | | | | |
| 6/10/2022 8:14 AM | | | | | | | | | | | | | | | | | |
|  | | | **Classification** |  | **Aggregate** |  | **Coverage** |  | **Number Of Coding References** |  | | **Reference Number** |  | **Coded By Initials** |  | **Modified On** |  |
|  | | | **Codes\\hope** | | | | | | | | | | | | | |  |
|  |  |  |  |  | No |  | 0.0548 |  | 1 |  | | | | | | |  |
|  | | |  |  |  |  |  |  |  |  | | | | | | | |
|  | | | | | | | | | | | | 1 |  | BV |  | 14/01/2020 2:43 PM |  |
|  | | | A: Yeah. So, my PSA came all the way to 15. So, it had a considerable drop. It was doing really well.  Q: Yeah.  A: Yeah and then all of a sudden it just started to rise and it continued to rise. So-  Q: Okay.  A: It’s a bit perplexing but you know.  Q: Yeah, well I guess you’ll get some more answers in the next week or so by the sounds of it.  A: Yeah.  Q: Hopefully that’ll give you some more direction for what’s going to happen next.  A: Certainly hope so. | | | | | | | | | | | | | |  |
|  |  |  |  |  |  |  |  |  |  |  |  |  |  |  |  |  |  |
|  |  |  |  |  |  |  |  |  |  |  |  |  |  |  |  |  |  |
|  | | |  | | | | | | | | | | | | | |  |
|  | | | **Codes\\hope\hoping PSA dropped further** | | | | | | | | | | | | | |  |
|  |  |  |  |  | No |  | 0.0548 |  | 1 |  | | | | | | |  |
|  | | |  |  |  |  |  |  |  |  | | | | | | | |
|  | | | | | | | | | | | | 1 |  | BV |  | 15/01/2020 9:56 AM |  |
|  | | | A: Yeah. So, my PSA came all the way to 15. So, it had a considerable drop. It was doing really well.  Q: Yeah.  A: Yeah and then all of a sudden it just started to rise and it continued to rise. So-  Q: Okay.  A: It’s a bit perplexing but you know.  Q: Yeah, well I guess you’ll get some more answers in the next week or so by the sounds of it.  A: Yeah.  Q: Hopefully that’ll give you some more direction for what’s going to happen next.  A: Certainly hope so. | | | | | | | | | | | | | |  |
|  |  |  |  |  |  |  |  |  |  |  |  |  |  |  |  |  |  |
|  |  |  |  |  |  |  |  |  |  |  |  |  |  |  |  |  |  |
|  | | |  | | | | | | | | | | | | | |  |
|  | | | **Codes\\initial motivation for participation** | | | | | | | | | | | | | |  |
|  |  |  |  |  | No |  | 0.0859 |  | 1 |  | | | | | | |  |
|  | | |  |  |  |  |  |  |  |  | | | | | | | |
|  | | | | | | | | | | | | 1 |  | BV |  | 14/01/2020 2:48 PM |  |
|  | | | I suppose when my – when I was at the PA hospital, my – I had the Docetaxel and it sort of knocked them down for about 3 years or so. And it started to rise again and I had some Docetaxel – some more Docetaxel that didn’t seem to work. So, rather than have the Cabazitaxel which I could’ve had, my doctor suggested that we wait until my PSA got up a bit to about the 20 mark and to try and get onto this trial because we believed there was some good result out of the trial. So, that was one of the motivations I suppose is to increase the amount of treatments I had because if I’d gone and had the Cabazitaxel, it would’ve excluded me from the trial. So, and look, I don’t regret having it because it certainly – first couple certainly did some good. | | | | | | | | | | | | | |  |
|  | | |  | | | | | | | | | | | | | |  |
| Formatted Reports\\Coding Summary by File Formatted Report | | | | | | | | | | | Page 87 of 143 | | | | | | |
| 6/10/2022 8:14 AM | | | | | | | | | | | | | | | | | |
|  | | | **Classification** |  | **Aggregate** |  | **Coverage** |  | **Number Of Coding References** |  | | **Reference Number** |  | **Coded By Initials** |  | **Modified On** |  |
|  | | | **Codes\\initial motivation for participation\failure in previous treatment** | | | | | | | | | | | | | |  |
|  |  |  |  |  | No |  | 0.0859 |  | 1 |  | | | | | | |  |
|  | | |  |  |  |  |  |  |  |  | | | | | | | |
|  | | | | | | | | | | | | 1 |  | BV |  | 14/01/2020 2:48 PM |  |
|  | | | I suppose when my – when I was at the PA hospital, my – I had the Docetaxel and it sort of knocked them down for about 3 years or so. And it started to rise again and I had some Docetaxel – some more Docetaxel that didn’t seem to work. So, rather than have the Cabazitaxel which I could’ve had, my doctor suggested that we wait until my PSA got up a bit to about the 20 mark and to try and get onto this trial because we believed there was some good result out of the trial. So, that was one of the motivations I suppose is to increase the amount of treatments I had because if I’d gone and had the Cabazitaxel, it would’ve excluded me from the trial. So, and look, I don’t regret having it because it certainly – first couple certainly did some good. | | | | | | | | | | | | | |  |
|  | | |  | | | | | | | | | | | | | |  |
|  | | | **Codes\\needing more support\no additional support required** | | | | | | | | | | | | | |  |
|  |  |  |  |  | No |  | 0.0187 |  | 1 |  | | | | | | |  |
|  | | |  |  |  |  |  |  |  |  | | | | | | | |
|  | | | | | | | | | | | | 1 |  | BV |  | 14/01/2020 2:48 PM |  |
|  | | | No. Look, I can’t complain anything about – I thought the care and the support’s been 100% from the guys. I couldn’t complain whatsoever. It’s always been spot on | | | | | | | | | | | | | |  |
|  | | |  | | | | | | | | | | | | | |  |
|  | | | **Codes\\not as intrusive as chemo** | | | | | | | | | | | | | |  |
|  |  |  |  |  | No |  | 0.1589 |  | 4 |  | | | | | | |  |
|  | | |  |  |  |  |  |  |  |  | | | | | | | |
|  | | | | | | | | | | | | 1 |  | BV |  | 14/01/2020 2:44 PM |  |
|  | | | No, no. It’s been quite good. It’s not very intrusive at all. They said there’s – I’ve had no sickness. No hair – all my hair’s – normally when I have the chemo, my hair falls out but I haven’t lost any of my hair, so – I haven’t felt sick. I’m really -it’s like you have the dose and you sort – they scan you to make sure you’re under a certain gauge and you walk out. And I feel the same as I leave versus when I come in. And even the next day I feel just as good as well. So, very little adjustment there. It’s quite good. | | | | | | | | | | | | | |  |
|  | | |  | | | | | | | | | | | | | |  |
|  | | | | | | | | | | | | 2 |  | BV |  | 14/01/2020 2:46 PM |  |
|  | | | Yeah, not knowing what’s happening sort of thing is a bit of a worry. So – but that would be the only – there’s no emotion from the treatment as such because like I said, it’s not intrusive at all. | | | | | | | | | | | | | |  |
|  | | |  | | | | | | | | | | | | | |  |
|  | | | | | | | | | | | | 3 |  | BV |  | 14/01/2020 2:47 PM |  |
|  | | | Not really. No. I really can’t think of anything because having the actual Lu PMSA compared to say, having something like chemo, it’s so much quicker. So, you really can’t even complain about that …. It’s a pretty quick process. So- | | | | | | | | | | | | | |  |
|  | | |  | | | | | | | | | | | | | |  |
|  | | | | | | | | | | | | 4 |  | BV |  | 14/01/2020 2:48 PM |  |
|  | | | I suppose I was lucky. When I was having the Docetaxel, that type of particular chemo, I was lucky, I had very few side effects apart from losing my hair and after – sort of the first couple of days after, I was sort of down a fair bit, quite tired and that but it picked up. But I didn’t have symptoms. I never vomited. I didn’t feel sick. All those sort of things, I was very lucky I didn’t get those sort of things. | | | | | | | | | | | | | |  |
|  | | |  | | | | | | | | | | | | | |  |
|  | | | | | | | | | | | | | | | | | |
|  | | | | | | | | | | | | | | | | | |
| Formatted Reports\\Coding Summary by File Formatted Report | | | | | | | | | | | Page 88 of 143 | | | | | | |
| 6/10/2022 8:14 AM | | | | | | | | | | | | | | | | | |
|  | | | **Classification** |  | **Aggregate** |  | **Coverage** |  | **Number Of Coding References** |  | | **Reference Number** |  | **Coded By Initials** |  | **Modified On** |  |
|  | | | **Codes\\PSA numbers going down** | | | | | | | | | | | | | |  |
|  |  |  |  |  | No |  | 0.0600 |  | 2 |  | | | | | | |  |
|  | | |  |  |  |  |  |  |  |  | | | | | | | |
|  | | | | | | | | | | | | 1 |  | BV |  | 14/01/2020 2:42 PM |  |
|  | | | A: Yeah. So, my PSA came all the way to 15. So, it had a considerable drop. It was doing really well.  Q: Yeah.  A: Yeah and then all of a sudden it just started to rise and it continued to rise. So-  Q: Okay.  A: It’s a bit perplexing but you know. | | | | | | | | | | | | | |  |
|  |  |  |  |  |  |  |  |  |  |  |  |  |  |  |  |  |  |
|  | | |  | | | | | | | | | | | | | |  |
|  | | | | | | | | | | | | 2 |  | BV |  | 14/01/2020 2:45 PM |  |
|  | | | Not from the treatment as such. I suppose it’s – with the PSA level rising, it’s quite draining emotionally because the PSA is a good gauge of what’s happening and mine continues to go up and in a pretty quick fashion. So, that does sort of drain you emotionally. | | | | | | | | | | | | | |  |
|  | | |  | | | | | | | | | | | | | |  |
|  | | | **Codes\\PSA numbers going down\PSA numbers going down - lutetium** | | | | | | | | | | | | | |  |
|  |  |  |  |  | No |  | 0.0600 |  | 2 |  | | | | | | |  |
|  | | |  |  |  |  |  |  |  |  | | | | | | | |
|  | | | | | | | | | | | | 1 |  | BV |  | 25/03/2020 8:22 AM |  |
|  | | | A: Yeah. So, my PSA came all the way to 15. So, it had a considerable drop. It was doing really well.  Q: Yeah.  A: Yeah and then all of a sudden it just started to rise and it continued to rise. So-  Q: Okay.  A: It’s a bit perplexing but you know. | | | | | | | | | | | | | |  |
|  |  |  |  |  |  |  |  |  |  |  |  |  |  |  |  |  |  |
|  | | |  | | | | | | | | | | | | | |  |
|  | | | | | | | | | | | | 2 |  | BV |  | 25/03/2020 8:22 AM |  |
|  | | | Not from the treatment as such. I suppose it’s – with the PSA level rising, it’s quite draining emotionally because the PSA is a good gauge of what’s happening and mine continues to go up and in a pretty quick fashion. So, that does sort of drain you emotionally. | | | | | | | | | | | | | |  |
|  | | |  | | | | | | | | | | | | | |  |
|  | | | **Codes\\PSA numbers not going down** | | | | | | | | | | | | | |  |
|  |  |  |  |  | No |  | 0.0822 |  | 3 |  | | | | | | |  |
|  | | |  |  |  |  |  |  |  |  | | | | | | | |
|  | | | | | | | | | | | | 1 |  | BV |  | 14/01/2020 2:38 PM |  |
|  | | | Yeah, I’ve seen better days. My PSA has jumped. It’s gone from - I suppose it’s doubled in the last 3 weeks. So, it’s gone from 48 to 110. But they’re a bit perplexed because the scans seem to show that the cancer’s sort of stable. It’s not growing. | | | | | | | | | | | | | |  |
|  | | |  | | | | | | | | | | | | | |  |
|  | | | | | | | | | | | | 2 |  | BV |  | 15/01/2020 5:00 AM |  |
|  | | | A: Yeah. So, my PSA came all the way to 15. So, it had a considerable drop. It was doing really well.  Q: Yeah.  A: Yeah and then all of a sudden it just started to rise and it continued to rise. | | | | | | | | | | | | | |  |
|  | | |  | | | | | | | | | | | | | |  |
| Formatted Reports\\Coding Summary by File Formatted Report | | | | | | | | | | | Page 89 of 143 | | | | | | |
| 6/10/2022 8:14 AM | | | | | | | | | | | | | | | | | |
|  | | | **Classification** |  | **Aggregate** |  | **Coverage** |  | **Number Of Coding References** |  | | **Reference Number** |  | **Coded By Initials** |  | **Modified On** |  |
|  | | | | | | | | | | | | | | | | | |
|  | | | | | | | | | | | | 3 |  | BV |  | 14/01/2020 2:45 PM |  |
|  | | | Not from the treatment as such. I suppose it’s – with the PSA level rising, it’s quite draining emotionally because the PSA is a good gauge of what’s happening and mine continues to go up and in a pretty quick fashion. So, that does sort of drain you emotionally. | | | | | | | | | | | | | |  |
|  | | |  | | | | | | | | | | | | | |  |
|  | | | **Codes\\PSA numbers not going down\PSA numbers not going down - lutetium** | | | | | | | | | | | | | |  |
|  |  |  |  |  | No |  | 0.0822 |  | 3 |  | | | | | | |  |
|  | | |  |  |  |  |  |  |  |  | | | | | | | |
|  | | | | | | | | | | | | 1 |  | BV |  | 25/03/2020 8:21 AM |  |
|  | | | Yeah, I’ve seen better days. My PSA has jumped. It’s gone from - I suppose it’s doubled in the last 3 weeks. So, it’s gone from 48 to 110. But they’re a bit perplexed because the scans seem to show that the cancer’s sort of stable. It’s not growing. | | | | | | | | | | | | | |  |
|  | | |  | | | | | | | | | | | | | |  |
|  | | | | | | | | | | | | 2 |  | BV |  | 25/03/2020 8:21 AM |  |
|  | | | A: Yeah. So, my PSA came all the way to 15. So, it had a considerable drop. It was doing really well.  Q: Yeah.  A: Yeah and then all of a sudden it just started to rise and it continued to rise. | | | | | | | | | | | | | |  |
|  | | |  | | | | | | | | | | | | | |  |
|  | | | | | | | | | | | | 3 |  | BV |  | 25/03/2020 8:21 AM |  |
|  | | | Not from the treatment as such. I suppose it’s – with the PSA level rising, it’s quite draining emotionally because the PSA is a good gauge of what’s happening and mine continues to go up and in a pretty quick fashion. So, that does sort of drain you emotionally. | | | | | | | | | | | | | |  |
|  | | |  | | | | | | | | | | | | | |  |
|  | | | **Codes\\quality of trialists** | | | | | | | | | | | | | |  |
|  |  |  |  |  | No |  | 0.1185 |  | 3 |  | | | | | | |  |
|  | | |  |  |  |  |  |  |  |  | | | | | | | |
|  | | | | | | | | | | | | 1 |  | BV |  | 14/01/2020 2:47 PM |  |
|  | | | I’d say that the whole crew from Jenny then nurse, she’s the coordinator of it all, right through to Dr Goh and they’ve always been there and they’re very supporting. Yeah, ask - they sort of ask a lot of questions; how do you feel? What’s going on? So, that they’re really involved int hat part of it, which is good. You – it’s good to see them really taking an interest and want to know how you physically feel and stuff like that. So, they’re good in that way. | | | | | | | | | | | | | |  |
|  | | |  | | | | | | | | | | | | | |  |
|  | | | | | | | | | | | | 2 |  | BV |  | 14/01/2020 2:48 PM |  |
|  | | | No. Look, I can’t complain anything about – I thought the care and the support’s been 100% from the guys. I couldn’t complain whatsoever. It’s always been spot on | | | | | | | | | | | | | |  |
|  | | |  | | | | | | | | | | | | | |  |
|  | | | | | | | | | | | | 3 |  | BV |  | 14/01/2020 2:49 PM |  |
|  | | | Look, I said – I wouldn’t say it’s a pleasant experience but like I said, you got sort of top notch care from all the people there, right from the – even when you walk in and sort of greet the ladies on the desk there. They’re straight on the ball on those sort of things. So, I can’t complain about anything from that. I think this was excellent, yep. On the ball all the time, so it was really good. | | | | | | | | | | | | | |  |
|  | | |  | | | | | | | | | | | | | |  |
|  | | | **Codes\\seen better days** | | | | | | | | | | | | | |  |
|  |  |  |  |  | No |  | 0.0031 |  | 1 |  | | | | | | |  |
|  | | |  |  |  |  |  |  |  |  | | | | | | | |
|  | | | | | | | | | | | | 1 |  | BV |  | 14/01/2020 2:34 PM |  |
|  | | | Yeah, I’ve seen better days. | | | | | | | | | | | | | |  |
|  | | |  | | | | | | | | | | | | | |  |
| Formatted Reports\\Coding Summary by File Formatted Report | | | | | | | | | | | Page 90 of 143 | | | | | | |
| 6/10/2022 8:14 AM | | | | | | | | | | | | | | | | | |
|  | | | **Classification** |  | **Aggregate** |  | **Coverage** |  | **Number Of Coding References** |  | | **Reference Number** |  | **Coded By Initials** |  | **Modified On** |  |
|  | | | **Codes\\side effects** | | | | | | | | | | | | | |  |
|  |  |  |  |  | No |  | 0.0666 |  | 2 |  | | | | | | |  |
|  | | |  |  |  |  |  |  |  |  | | | | | | | |
|  | | | | | | | | | | | | 1 |  | BV |  | 25/03/2020 8:26 AM |  |
|  | | | No, no. It’s been quite good. It’s not very intrusive at all. They said there’s – I’ve had no sickness. No hair – all my hair’s – normally when I have the chemo, my hair falls out but I haven’t lost any of my hair, so – I haven’t felt sick. I’m really -it’s like you have the dose and you sort – they scan you to make sure you’re under a certain gauge and you walk out. And I feel the same as I leave versus when I come in. And even the next day I feel just as good as well. So, very little adjustment there. It’s quite good. | | | | | | | | | | | | | |  |
|  | | |  | | | | | | | | | | | | | |  |
|  | | | | | | | | | | | | 2 |  | BV |  | 25/03/2020 8:26 AM |  |
|  | | | Q: So, no side effects?  A: No. Not whatsoever. | | | | | | | | | | | | | |  |
|  | | |  | | | | | | | | | | | | | |  |
|  | | | **Codes\\side effects\side effects - lutetium** | | | | | | | | | | | | | |  |
|  |  |  |  |  | No |  | 0.0666 |  | 2 |  | | | | | | |  |
|  | | |  |  |  |  |  |  |  |  | | | | | | | |
|  | | | | | | | | | | | | 1 |  | BV |  | 14/01/2020 2:44 PM |  |
|  | | | No, no. It’s been quite good. It’s not very intrusive at all. They said there’s – I’ve had no sickness. No hair – all my hair’s – normally when I have the chemo, my hair falls out but I haven’t lost any of my hair, so – I haven’t felt sick. I’m really -it’s like you have the dose and you sort – they scan you to make sure you’re under a certain gauge and you walk out. And I feel the same as I leave versus when I come in. And even the next day I feel just as good as well. So, very little adjustment there. It’s quite good. | | | | | | | | | | | | | |  |
|  | | |  | | | | | | | | | | | | | |  |
|  | | | | | | | | | | | | 2 |  | BV |  | 14/01/2020 2:44 PM |  |
|  | | | Q: So, no side effects?  A: No. Not whatsoever. | | | | | | | | | | | | | |  |
|  | | |  | | | | | | | | | | | | | |  |
|  | | | **Codes\\struggling with treatment\symptom fluctuations** | | | | | | | | | | | | | |  |
|  |  |  |  |  | No |  | 0.0669 |  | 2 |  | | | | | | |  |
|  | | |  |  |  |  |  |  |  |  | | | | | | | |
|  | | | | | | | | | | | | 1 |  | BV |  | 14/01/2020 2:41 PM |  |
|  | | | A: Yeah. So, my PSA came all the way to 15. So, it had a considerable drop. It was doing really well.  Q: Yeah.  A: Yeah and then all of a sudden it just started to rise and it continued to rise. | | | | | | | | | | | | | |  |
|  | | |  | | | | | | | | | | | | | |  |
|  | | | | | | | | | | | | 2 |  | BV |  | 14/01/2020 2:44 PM |  |
|  | | | Q: Okay. Do you feel an improvement or not? Or just the same?  A: Yeah. It’s hard to say. So, yeah, some days I feel a little bit off colour than other days. But that mightn’t be towards the Lu PMSA, it could anything. It just could be – the day before could be strenuous and just … or whatever. So, it’s been pretty – I really haven’t noticed much of a difference at all. So- | | | | | | | | | | | | | |  |
|  | | |  | | | | | | | | | | | | | |  |
|  | | | **Codes\\support from work** | | | | | | | | | | | | | |  |
|  |  |  |  |  | No |  | 0.0392 |  | 2 |  | | | | | | |  |
|  | | |  |  |  |  |  |  |  |  | | | | | | | |
|  | | | | | | | | | | | | 1 |  | BV |  | 14/01/2020 2:46 PM |  |
|  | | | No. Not at all. I’ve got a good support from work here. Got a good crew we work with. And that’s – they’ve always – if I need to have time off, they’ve always covered and so, there’s never been that at all. So, I’m lucky I’ve got a lot of sick leave up my sleeve at the moment. So, that’s what- | | | | | | | | | | | | | |  |
|  | | |  | | | | | | | | | | | | | |  |
| Formatted Reports\\Coding Summary by File Formatted Report | | | | | | | | | | | Page 91 of 143 | | | | | | |
| 6/10/2022 8:14 AM | | | | | | | | | | | | | | | | | |
|  | | | **Classification** |  | **Aggregate** |  | **Coverage** |  | **Number Of Coding References** |  | | **Reference Number** |  | **Coded By Initials** |  | **Modified On** |  |
|  | | | | | | | | | | | | | | | | | |
|  | | | | | | | | | | | | 2 |  | BV |  | 14/01/2020 2:46 PM |  |
|  | | | Yeah, good mob. Very god mob to work with. | | | | | | | | | | | | | |  |
|  | | |  | | | | | | | | | | | | | |  |
|  | | | **Codes\\treatments been good** | | | | | | | | | | | | | |  |
|  |  |  |  |  | No |  | 0.1072 |  | 2 |  | | | | | | |  |
|  | | |  |  |  |  |  |  |  |  | | | | | | | |
|  | | | | | | | | | | | | 1 |  | BV |  | 14/01/2020 2:44 PM |  |
|  | | | No, no. It’s been quite good. It’s not very intrusive at all. They said there’s – I’ve had no sickness. No hair – all my hair’s – normally when I have the chemo, my hair falls out but I haven’t lost any of my hair, so – I haven’t felt sick. I’m really -it’s like you have the dose and you sort – they scan you to make sure you’re under a certain gauge and you walk out. And I feel the same as I leave versus when I come in. And even the next day I feel just as good as well. So, very little adjustment there. It’s quite good. | | | | | | | | | | | | | |  |
|  | | |  | | | | | | | | | | | | | |  |
|  | | | | | | | | | | | | 2 |  | BV |  | 14/01/2020 2:49 PM |  |
|  | | | Look, I said – I wouldn’t say it’s a pleasant experience but like I said, you got sort of top notch care from all the people there, right from the – even when you walk in and sort of greet the ladies on the desk there. They’re straight on the ball on those sort of things. So, I can’t complain about anything from that. I think this was excellent, yep. On the ball all the time, so it was really good. | | | | | | | | | | | | | |  |
|  | | |  | | | | | | | | | | | | | |  |
|  | | | **Codes\\treatments left** | | | | | | | | | | | | | |  |
|  |  |  |  |  | No |  | 0.0077 |  | 1 |  | | | | | | |  |
|  | | |  |  |  |  |  |  |  |  | | | | | | | |
|  | | | | | | | | | | | | 1 |  | BV |  | 14/01/2020 2:41 PM |  |
|  | | | Q: Okay. How many treatments of the Lutetium have you had?  A: 5. | | | | | | | | | | | | | |  |
|  | | |  | | | | | | | | | | | | | |  |
|  | | | **Codes\\uncertainty of the future** | | | | | | | | | | | | | |  |
|  |  |  |  |  | No |  | 0.1288 |  | 3 |  | | | | | | |  |
|  | | |  |  |  |  |  |  |  |  | | | | | | | |
|  | | | | | | | | | | | | 1 |  | BV |  | 14/01/2020 2:38 PM |  |
|  | | | Yeah, I’ve seen better days. My PSA has jumped. It’s gone from - I suppose it’s doubled in the last 3 weeks. So, it’s gone from 48 to 110. But they’re a bit perplexed because the scans seem to show that the cancer’s sort of stable. It’s not growing. So, I’m in the process of seeing Dr – so, I’ve just seen Dr Goh on Tuesday. I’ll have a blood on Wednesday and I go back to see Dr Goh again next Friday to discuss a course of action. Whether we go – continue to go with the … or I go off and have some Lu PMSA - no, no, Cabazitaxel. So, I might go back to the chemo. So- | | | | | | | | | | | | | |  |
|  | | |  | | | | | | | | | | | | | |  |
|  | | | | | | | | | | | | 2 |  | BV |  | 14/01/2020 2:43 PM |  |
|  | | | A: Yeah. So, my PSA came all the way to 15. So, it had a considerable drop. It was doing really well.  Q: Yeah.  A: Yeah and then all of a sudden it just started to rise and it continued to rise. So-  Q: Okay.  A: It’s a bit perplexing but you know.  Q: Yeah, well I guess you’ll get some more answers in the next week or so by the sounds of it.  A: Yeah.  Q: Hopefully that’ll give you some more direction for what’s going to happen next.  A: Certainly hope so. | | | | | | | | | | | | | |  |
|  |  |  |  |  |  |  |  |  |  |  |  |  |  |  |  |  |  |
|  |  |  |  |  |  |  |  |  |  |  |  |  |  |  |  |  |  |
|  | | |  | | | | | | | | | | | | | |  |
| Formatted Reports\\Coding Summary by File Formatted Report | | | | | | | | | | | Page 92 of 143 | | | | | | |
| 6/10/2022 8:14 AM | | | | | | | | | | | | | | | | | |
|  | | | **Classification** |  | **Aggregate** |  | **Coverage** |  | **Number Of Coding References** |  | | **Reference Number** |  | **Coded By Initials** |  | **Modified On** |  |
|  | | | | | | | | | | | | | | | | | |
|  | | | | | | | | | | | | 3 |  | BV |  | 14/01/2020 2:46 PM |  |
|  | | | Yeah, not knowing what’s happening sort of thing is a bit of a worry. | | | | | | | | | | | | | |  |
|  | | |  | | | | | | | | | | | | | |  |
|  | | | **Codes\\worry** | | | | | | | | | | | | | |  |
|  |  |  |  |  | No |  | 0.0079 |  | 1 |  | | | | | | |  |
|  | | |  |  |  |  |  |  |  |  | | | | | | | |
|  | | | | | | | | | | | | 1 |  | BV |  | 14/01/2020 2:46 PM |  |
|  | | | Yeah, not knowing what’s happening sort of thing is a bit of a worry. | | | | | | | | | | | | | |  |
|  | | |  | | | | | | | | | | | | | |  |
|  | **Files\\Transcription 3_006** | | | | | | | | | | | | | | | |  |
|  | | **Code** | | | | | | | | | | | | | | |  |
|  | | | **Codes\\coping strategies\coping okay** | | | | | | | | | | | | | |  |
|  |  |  |  |  | No |  | 0.0071 |  | 1 |  | | | | | | |  |
|  | | |  |  |  |  |  |  |  |  | | | | | | | |
|  | | | | | | | | | | | | 1 |  | BV |  | 14/01/2020 2:57 PM |  |
|  | | | Oh no more than – no more than normal, no. | | | | | | | | | | | | | |  |
|  | | |  | | | | | | | | | | | | | |  |
|  | | | **Codes\\feeling disappointed** | | | | | | | | | | | | | |  |
|  |  |  |  |  | No |  | 0.0551 |  | 1 |  | | | | | | |  |
|  | | |  |  |  |  |  |  |  |  | | | | | | | |
|  | | | | | | | | | | | | 1 |  | BV |  | 14/01/2020 2:55 PM |  |
|  | | | The personal experience has been good. It’s been – it’s been very good, very positive. Although it hasn’t been working all that well. It works for a little bit less time, every time I have it. But it’s – it’s - they’re all …, and – and released from all the symptoms – for cancer symptoms. Which is pain mainly, has been good. | | | | | | | | | | | | | |  |
|  | | |  | | | | | | | | | | | | | |  |
|  | | | **Codes\\financial impact** | | | | | | | | | | | | | |  |
|  |  |  |  |  | No |  | 0.0291 |  | 1 |  | | | | | | |  |
|  | | |  |  |  |  |  |  |  |  | | | | | | | |
|  | | | | | | | | | | | | 1 |  | BV |  | 14/01/2020 2:57 PM |  |
|  | | | Oh it’s always a little bit of financial problem with parking and things for – it’s – the parking is fairly expensive. And – but other than that no, not – not really, no. | | | | | | | | | | | | | |  |
|  | | |  | | | | | | | | | | | | | |  |
|  | | | | | | | | | | | | | | | | | |
| Formatted Reports\\Coding Summary by File Formatted Report | | | | | | | | | | | Page 93 of 143 | | | | | | |
| 6/10/2022 8:14 AM | | | | | | | | | | | | | | | | | |
|  | | | **Classification** |  | **Aggregate** |  | **Coverage** |  | **Number Of Coding References** |  | | **Reference Number** |  | **Coded By Initials** |  | **Modified On** |  |
|  | | | **Codes\\financial impact\hospital parking** | | | | | | | | | | | | | |  |
|  |  |  |  |  | No |  | 0.0291 |  | 1 |  | | | | | | |  |
|  | | |  |  |  |  |  |  |  |  | | | | | | | |
|  | | | | | | | | | | | | 1 |  | BV |  | 15/01/2020 5:09 AM |  |
|  | | | Oh it’s always a little bit of financial problem with parking and things for – it’s – the parking is fairly expensive. And – but other than that no, not – not really, no. | | | | | | | | | | | | | |  |
|  | | |  | | | | | | | | | | | | | |  |
|  | | | **Codes\\going back and forth to the hospital** | | | | | | | | | | | | | |  |
|  |  |  |  |  | No |  | 0.0524 |  | 1 |  | | | | | | |  |
|  | | |  |  |  |  |  |  |  |  | | | | | | | |
|  | | | | | | | | | | | | 1 |  | BV |  | 14/01/2020 2:56 PM |  |
|  | | | It’s a bit inconvenient sometimes to sort of get into from where we are into … to northern – the northern – northern suburbs. Which is where the Royal Brisbane is. Had to – having to travel through peak hour traffic. Can be a little bit – a little bit hit and miss. But other than that it hasn’t been too bad. | | | | | | | | | | | | | |  |
|  | | |  | | | | | | | | | | | | | |  |
|  | | | **Codes\\hope** | | | | | | | | | | | | | |  |
|  |  |  |  |  | No |  | 0.0431 |  | 1 |  | | | | | | |  |
|  | | |  |  |  |  |  |  |  |  | | | | | | | |
|  | | | | | | | | | | | | 1 |  | BV |  | 14/01/2020 2:59 PM |  |
|  | | | Q: No that’s fine. Thank you for answering my questions. I really hope that things with your tests improve. And that you can return to receiving treatment-  A: Yes thank you for that. I – I hope so too. Just got to keep our fingers crossed haven’t we? | | | | | | | | | | | | | |  |
|  | | |  | | | | | | | | | | | | | |  |
|  | | | **Codes\\initial motivation for participation** | | | | | | | | | | | | | |  |
|  |  |  |  |  | No |  | 0.0166 |  | 1 |  | | | | | | |  |
|  | | |  |  |  |  |  |  |  |  | | | | | | | |
|  | | | | | | | | | | | | 1 |  | BV |  | 14/01/2020 2:57 PM |  |
|  | | | Just something that – that works. That there’s something that lessens the – the effects of cancer. | | | | | | | | | | | | | |  |
|  | | |  | | | | | | | | | | | | | |  |
|  | | | **Codes\\initial motivation for participation\changes to initial motivation for participation** | | | | | | | | | | | | | |  |
|  |  |  |  |  | No |  | 0.0351 |  | 1 |  | | | | | | |  |
|  | | |  |  |  |  |  |  |  |  | | | | | | | |
|  | | | | | | | | | | | | 1 |  | BV |  | 14/01/2020 2:57 PM |  |
|  | | | Well I don’t think it’s changed. I mean the – the – the results have not been as good as we like. But no it’s – it’s – we’d like the results to be better. But apart from that no it’s nothing I can think of. | | | | | | | | | | | | | |  |
|  | | |  | | | | | | | | | | | | | |  |
|  | | | **Codes\\low blood count** | | | | | | | | | | | | | |  |
|  |  |  |  |  | No |  | 0.2091 |  | 4 |  | | | | | | |  |
|  | | |  |  |  |  |  |  |  |  | | | | | | | |
|  | | | | | | | | | | | | 1 |  | BV |  | 14/01/2020 2:53 PM |  |
|  | | | Well I couldn’t have the last infusion of lutetium, because my blood count was down too low. So I’ve had no – no treatment at all. And that’s made things a little hard, because I’m just on …, that’s all I’m on. And I’ve had a fair bit of pain. | | | | | | | | | | | | | |  |
|  | | |  | | | | | | | | | | | | | |  |
| Formatted Reports\\Coding Summary by File Formatted Report | | | | | | | | | | | Page 94 of 143 | | | | | | |
| 6/10/2022 8:14 AM | | | | | | | | | | | | | | | | | |
|  | | | **Classification** |  | **Aggregate** |  | **Coverage** |  | **Number Of Coding References** |  | | **Reference Number** |  | **Coded By Initials** |  | **Modified On** |  |
|  | | | | | | | | | | | | | | | | | |
|  | | | | | | | | | | | | 2 |  | BV |  | 14/01/2020 2:53 PM |  |
|  | | | I had a treatment – just trying to work backwards. I was supposed to get a treatment last Tuesday. What’s today? Todays Thursday. So not – like a week ago on Tuesday I was supposed to get a treatment. And I didn’t, and I couldn’t. And the one before that – 6 weeks before that was when I had my last treatment. So you have to work backwards. | | | | | | | | | | | | | |  |
|  | | |  | | | | | | | | | | | | | |  |
|  | | | | | | | | | | | | 3 |  | BV |  | 14/01/2020 2:53 PM |  |
|  | | | Well it all depends – so it all depends on the blood count – my white blood cells. That’s too low to give me any treatment at all. So I had a blood test on Tuesday. And it was still low. So I’ve got to wait another week. Then I’ll have another blood test. And depending on the results of that blood test they will either schedule me for another lutetium dose, or not. Depending on the blood count. | | | | | | | | | | | | | |  |
|  | | |  | | | | | | | | | | | | | |  |
|  | | | | | | | | | | | | 4 |  | BV |  | 14/01/2020 2:57 PM |  |
|  | | | Yeah well not being able to control the pain has affected my quality of life. But as I say hopefully now we’re getting a bit on top of that. And hopefully I’ll be – white blood count will be a little higher. And I’ll be able to continue the treatment. | | | | | | | | | | | | | |  |
|  | | |  | | | | | | | | | | | | | |  |
|  | | | **Codes\\needing more support\no additional support required** | | | | | | | | | | | | | |  |
|  |  |  |  |  | No |  | 0.0184 |  | 1 |  | | | | | | |  |
|  | | |  |  |  |  |  |  |  |  | | | | | | | |
|  | | | | | | | | | | | | 1 |  | BV |  | 14/01/2020 2:56 PM |  |
|  | | | No I think it’s been pretty good. I think the support and care has been excellent really. No it’s been good. | | | | | | | | | | | | | |  |
|  | | |  | | | | | | | | | | | | | |  |
|  | | | **Codes\\needing to see better results** | | | | | | | | | | | | | |  |
|  |  |  |  |  | No |  | 0.0722 |  | 2 |  | | | | | | |  |
|  | | |  |  |  |  |  |  |  |  | | | | | | | |
|  | | | | | | | | | | | | 1 |  | BV |  | 14/01/2020 2:58 PM |  |
|  | | | I mean the – the – the results have not been as good as we like. But no it’s – it’s – we’d like the results to be better. But apart from that no it’s nothing I can think of. | | | | | | | | | | | | | |  |
|  | | |  | | | | | | | | | | | | | |  |
|  | | | | | | | | | | | | 2 |  | BV |  | 14/01/2020 2:59 PM |  |
|  | | | Q: No that’s fine. Thank you for answering my questions. I really hope that things with your tests improve. And that you can return to receiving treatment-  A: Yes thank you for that. I – I hope so too. Just got to keep our fingers crossed haven’t we? | | | | | | | | | | | | | |  |
|  | | |  | | | | | | | | | | | | | |  |
|  | | | **Codes\\quality of life changes** | | | | | | | | | | | | | |  |
|  |  |  |  |  | No |  | 0.0424 |  | 1 |  | | | | | | |  |
|  | | |  |  |  |  |  |  |  |  | | | | | | | |
|  | | | | | | | | | | | | 1 |  | BV |  | 25/03/2020 8:25 AM |  |
|  | | | Yeah well not being able to control the pain has affected my quality of life. But as I say hopefully now we’re getting a bit on top of that. And hopefully I’ll be – white blood count will be a little higher. And I’ll be able to continue the treatment. | | | | | | | | | | | | | |  |
|  | | |  | | | | | | | | | | | | | |  |
|  | | | | | | | | | | | | | | | | | |
| Formatted Reports\\Coding Summary by File Formatted Report | | | | | | | | | | | Page 95 of 143 | | | | | | |
| 6/10/2022 8:14 AM | | | | | | | | | | | | | | | | | |
|  | | | **Classification** |  | **Aggregate** |  | **Coverage** |  | **Number Of Coding References** |  | | **Reference Number** |  | **Coded By Initials** |  | **Modified On** |  |
|  | | | **Codes\\quality of life changes\quality of life changes - lutetium** | | | | | | | | | | | | | |  |
|  |  |  |  |  | No |  | 0.0424 |  | 1 |  | | | | | | |  |
|  | | |  |  |  |  |  |  |  |  | | | | | | | |
|  | | | | | | | | | | | | 1 |  | BV |  | 14/01/2020 2:56 PM |  |
|  | | | Yeah well not being able to control the pain has affected my quality of life. But as I say hopefully now we’re getting a bit on top of that. And hopefully I’ll be – white blood count will be a little higher. And I’ll be able to continue the treatment. | | | | | | | | | | | | | |  |
|  | | |  | | | | | | | | | | | | | |  |
|  | | | **Codes\\quality of trialists** | | | | | | | | | | | | | |  |
|  |  |  |  |  | No |  | 0.0274 |  | 2 |  | | | | | | |  |
|  | | |  |  |  |  |  |  |  |  | | | | | | | |
|  | | | | | | | | | | | | 1 |  | BV |  | 14/01/2020 2:55 PM |  |
|  | | | It’s – it’s been good. It’s – I’ve got no complaints. | | | | | | | | | | | | | |  |
|  | | |  | | | | | | | | | | | | | |  |
|  | | | | | | | | | | | | 2 |  | BV |  | 14/01/2020 2:56 PM |  |
|  | | | No I think it’s been pretty good. I think the support and care has been excellent really. No it’s been good. | | | | | | | | | | | | | |  |
|  | | |  | | | | | | | | | | | | | |  |
|  | | | **Codes\\side effects** | | | | | | | | | | | | | |  |
|  |  |  |  |  | No |  | 0.1522 |  | 4 |  | | | | | | |  |
|  | | |  |  |  |  |  |  |  |  | | | | | | | |
|  | | | | | | | | | | | | 1 |  | BV |  | 25/03/2020 8:26 AM |  |
|  | | | Well I couldn’t have the last infusion of lutetium, because my blood count was down too low. So I’ve had no – no treatment at all. And that’s made things a little hard, because I’m just on …, that’s all I’m on. And I’ve had a fair bit of pain. | | | | | | | | | | | | | |  |
|  | | |  | | | | | | | | | | | | | |  |
|  | | | | | | | | | | | | 2 |  | BV |  | 25/03/2020 8:26 AM |  |
|  | | | Although it hasn’t been working all that well. It works for a little bit less time, every time I have it. But it’s – it’s - they’re all …, and – and released from all the symptoms – for cancer symptoms. Which is pain mainly, has been good. | | | | | | | | | | | | | |  |
|  | | |  | | | | | | | | | | | | | |  |
|  | | | | | | | | | | | | 3 |  | BV |  | 25/03/2020 8:26 AM |  |
|  | | | It’s - just had a few side effects like dry mouth, and things like that. But apart from that it’s been reasonability good. | | | | | | | | | | | | | |  |
|  | | |  | | | | | | | | | | | | | |  |
|  | | | | | | | | | | | | 4 |  | BV |  | 25/03/2020 8:26 AM |  |
|  | | | As I say keeping the pain under control has been a big problem the last few weeks. But apart from that I think we might be on top of that now hopefully. But apart from that it’s – it’s all been fairly good. But yeah getting on top of the – the pain control has been probably the biggest thing. | | | | | | | | | | | | | |  |
|  | | |  | | | | | | | | | | | | | |  |
|  | | | **Codes\\side effects\side effects - lutetium** | | | | | | | | | | | | | |  |
|  |  |  |  |  | No |  | 0.1525 |  | 4 |  | | | | | | |  |
|  | | |  |  |  |  |  |  |  |  | | | | | | | |
|  | | | | | | | | | | | | 1 |  | BV |  | 15/01/2020 5:21 AM |  |
|  | | | Well I couldn’t have the last infusion of lutetium, because my blood count was down too low. So I’ve had no – no treatment at all. And that’s made things a little hard, because I’m just on …, that’s all I’m on. And I’ve had a fair bit of pain. | | | | | | | | | | | | | |  |
|  | | |  | | | | | | | | | | | | | |  |
| Formatted Reports\\Coding Summary by File Formatted Report | | | | | | | | | | | Page 96 of 143 | | | | | | |
| 6/10/2022 8:14 AM | | | | | | | | | | | | | | | | | |
|  | | | **Classification** |  | **Aggregate** |  | **Coverage** |  | **Number Of Coding References** |  | | **Reference Number** |  | **Coded By Initials** |  | **Modified On** |  |
|  | | | | | | | | | | | | | | | | | |
|  | | | | | | | | | | | | 2 |  | BV |  | 15/01/2020 5:25 AM |  |
|  | | | Although it hasn’t been working all that well. It works for a little bit less time, every time I have it. But it’s – it’s - they’re all …, and – and released from all the symptoms – for cancer symptoms. Which is pain mainly, has been good. | | | | | | | | | | | | | |  |
|  | | |  | | | | | | | | | | | | | |  |
|  | | | | | | | | | | | | 3 |  | BV |  | 14/01/2020 2:55 PM |  |
|  | | | It’s - just had a few side effects like dry mouth, and things like that. But apart from that it’s been reasonability good. | | | | | | | | | | | | | |  |
|  | | |  | | | | | | | | | | | | | |  |
|  | | | | | | | | | | | | 4 |  | BV |  | 15/01/2020 5:25 AM |  |
|  | | | As I say keeping the pain under control has been a big problem the last few weeks. But apart from that I think we might be on top of that now hopefully. But apart from that it’s – it’s all been fairly good. But yeah getting on top of the – the pain control has been probably the biggest thing. | | | | | | | | | | | | | |  |
|  | | |  | | | | | | | | | | | | | |  |
|  | | | **Codes\\side effects\side effects - lutetium\reduction in pain** | | | | | | | | | | | | | |  |
|  |  |  |  |  | No |  | 0.0899 |  | 2 |  | | | | | | |  |
|  | | |  |  |  |  |  |  |  |  | | | | | | | |
|  | | | | | | | | | | | | 1 |  | BV |  | 14/01/2020 2:54 PM |  |
|  | | | Although it hasn’t been working all that well. It works for a little bit less time, every time I have it. But it’s – it’s - they’re all …, and – and released from all the symptoms – for cancer symptoms. Which is pain mainly, has been good. | | | | | | | | | | | | | |  |
|  | | |  | | | | | | | | | | | | | |  |
|  | | | | | | | | | | | | 2 |  | BV |  | 14/01/2020 2:56 PM |  |
|  | | | As I say keeping the pain under control has been a big problem the last few weeks. But apart from that I think we might be on top of that now hopefully. But apart from that it’s – it’s all been fairly good. But yeah getting on top of the – the pain control has been probably the biggest thing. | | | | | | | | | | | | | |  |
|  | | |  | | | | | | | | | | | | | |  |
|  | | | **Codes\\staying positive** | | | | | | | | | | | | | |  |
|  |  |  |  |  | No |  | 0.0551 |  | 1 |  | | | | | | |  |
|  | | |  |  |  |  |  |  |  |  | | | | | | | |
|  | | | | | | | | | | | | 1 |  | BV |  | 15/01/2020 5:00 AM |  |
|  | | | The personal experience has been good. It’s been – it’s been very good, very positive. Although it hasn’t been working all that well. It works for a little bit less time, every time I have it. But it’s – it’s - they’re all …, and – and released from all the symptoms – for cancer symptoms. Which is pain mainly, has been good. | | | | | | | | | | | | | |  |
|  | | |  | | | | | | | | | | | | | |  |
|  | | | **Codes\\struggling with treatment** | | | | | | | | | | | | | |  |
|  |  |  |  |  | No |  | 0.0404 |  | 1 |  | | | | | | |  |
|  | | |  |  |  |  |  |  |  |  | | | | | | | |
|  | | | | | | | | | | | | 1 |  | BV |  | 14/01/2020 2:55 PM |  |
|  | | | Although it hasn’t been working all that well. It works for a little bit less time, every time I have it. But it’s – it’s - they’re all …, and – and released from all the symptoms – for cancer symptoms. Which is pain mainly, has been good. | | | | | | | | | | | | | |  |
|  | | |  | | | | | | | | | | | | | |  |
|  | | | | | | | | | | | | | | | | | |
| Formatted Reports\\Coding Summary by File Formatted Report | | | | | | | | | | | Page 97 of 143 | | | | | | |
| 6/10/2022 8:14 AM | | | | | | | | | | | | | | | | | |
|  | | | **Classification** |  | **Aggregate** |  | **Coverage** |  | **Number Of Coding References** |  | | **Reference Number** |  | **Coded By Initials** |  | **Modified On** |  |
|  | | | **Codes\\struggling with treatment\struggling with additional health issues** | | | | | | | | | | | | | |  |
|  |  |  |  |  | No |  | 0.0411 |  | 1 |  | | | | | | |  |
|  | | |  |  |  |  |  |  |  |  | | | | | | | |
|  | | | | | | | | | | | | 1 |  | BV |  | 15/01/2020 5:21 AM |  |
|  | | | Well I couldn’t have the last infusion of lutetium, because my blood count was down too low. So I’ve had no – no treatment at all. And that’s made things a little hard, because I’m just on …, that’s all I’m on. And I’ve had a fair bit of pain. | | | | | | | | | | | | | |  |
|  | | |  | | | | | | | | | | | | | |  |
|  | | | **Codes\\travelling to treatment\travelling to treatment challenging** | | | | | | | | | | | | | |  |
|  |  |  |  |  | No |  | 0.0524 |  | 1 |  | | | | | | |  |
|  | | |  |  |  |  |  |  |  |  | | | | | | | |
|  | | | | | | | | | | | | 1 |  | BV |  | 15/01/2020 5:10 AM |  |
|  | | | It’s a bit inconvenient sometimes to sort of get into from where we are into … to northern – the northern – northern suburbs. Which is where the Royal Brisbane is. Had to – having to travel through peak hour traffic. Can be a little bit – a little bit hit and miss. But other than that it hasn’t been too bad. | | | | | | | | | | | | | |  |
|  | | |  | | | | | | | | | | | | | |  |
|  | | | **Codes\\treatments been good** | | | | | | | | | | | | | |  |
|  |  |  |  |  | No |  | 0.0224 |  | 1 |  | | | | | | |  |
|  | | |  |  |  |  |  |  |  |  | | | | | | | |
|  | | | | | | | | | | | | 1 |  | BV |  | 14/01/2020 2:54 PM |  |
|  | | | The personal experience has been good. It’s been – it’s been very good, very positive. Although it hasn’t been working all that well. | | | | | | | | | | | | | |  |
|  | | |  | | | | | | | | | | | | | |  |
|  | **Files\\Transcription 3_008** | | | | | | | | | | | | | | | |  |
|  | | **Code** | | | | | | | | | | | | | | |  |
|  | | | **Codes\\coping strategies\coping okay** | | | | | | | | | | | | | |  |
|  |  |  |  |  | No |  | 0.0078 |  | 1 |  | | | | | | |  |
|  | | |  |  |  |  |  |  |  |  | | | | | | | |
|  | | | | | | | | | | | | 1 |  | BV |  | 14/01/2020 1:52 PM |  |
|  | | | Q: Any concerns emotionally or from a … (Unable to understand) view?  A: No. | | | | | | | | | | | | | |  |
|  | | |  | | | | | | | | | | | | | |  |
|  | | | **Codes\\emotional impact** | | | | | | | | | | | | | |  |
|  |  |  |  |  | No |  | 0.0385 |  | 1 |  | | | | | | |  |
|  | | |  |  |  |  |  |  |  |  | | | | | | | |
|  | | | | | | | | | | | | 1 |  | BV |  | 15/01/2020 5:30 AM |  |
|  | | | I was feeling depressed early because I was, there was talk about my, bringing a walking frame home, having physios come out to visit me, a social worker, home help and all these sorts of things. And the way my leg was feeling after some days, I thought I'm, will I ever get better? Because the tests had started and nothing was coming up, so naturally I was feeling very, very low. | | | | | | | | | | | | | |  |
|  | | |  | | | | | | | | | | | | | |  |
| Formatted Reports\\Coding Summary by File Formatted Report | | | | | | | | | | | Page 98 of 143 | | | | | | |
| 6/10/2022 8:14 AM | | | | | | | | | | | | | | | | | |
|  | | | **Classification** |  | **Aggregate** |  | **Coverage** |  | **Number Of Coding References** |  | | **Reference Number** |  | **Coded By Initials** |  | **Modified On** |  |
|  | | | **Codes\\financial impact\hospital parking** | | | | | | | | | | | | | |  |
|  |  |  |  |  | No |  | 0.0163 |  | 1 |  | | | | | | |  |
|  | | |  |  |  |  |  |  |  |  | | | | | | | |
|  | | | | | | | | | | | | 1 |  | BV |  | 14/01/2020 1:54 PM |  |
|  | | | Simple, and I don’t take the car because parking is so difficult at Peter Mac, and then I can get tangled up in traffic, and public transport for me is the option. | | | | | | | | | | | | | |  |
|  | | |  | | | | | | | | | | | | | |  |
|  | | | **Codes\\financial impact\no affect to finances** | | | | | | | | | | | | | |  |
|  |  |  |  |  | No |  | 0.0164 |  | 2 |  | | | | | | |  |
|  | | |  |  |  |  |  |  |  |  | | | | | | | |
|  | | | | | | | | | | | | 1 |  | BV |  | 14/01/2020 1:52 PM |  |
|  | | | Q: Alright, any financial or practical implications?  A: No. | | | | | | | | | | | | | |  |
|  | | |  | | | | | | | | | | | | | |  |
|  | | | | | | | | | | | | 2 |  | BV |  | 14/01/2020 1:54 PM |  |
|  | | | Q: Great, and you don’t have any financial implications using public transport?  A: No not at all. | | | | | | | | | | | | | |  |
|  | | |  | | | | | | | | | | | | | |  |
|  | | | **Codes\\happy about participating** | | | | | | | | | | | | | |  |
|  |  |  |  |  | No |  | 0.0192 |  | 1 |  | | | | | | |  |
|  | | |  |  |  |  |  |  |  |  | | | | | | | |
|  | | | | | | | | | | | | 1 |  | BV |  | 14/01/2020 1:57 PM |  |
|  | | | Q: Okay so what would you like to ultimately get out of this trial?  A: Well in two words – good health.  Q: Okay and do you feel like that’s happening at the moment?  A: Yes I do, yeah. | | | | | | | | | | | | | |  |
|  |  |  |  |  |  |  |  |  |  |  |  |  |  |  |  |  |  |
|  | | |  | | | | | | | | | | | | | |  |
|  | | | **Codes\\initial motivation for participation** | | | | | | | | | | | | | |  |
|  |  |  |  |  | No |  | 0.0106 |  | 1 |  | | | | | | |  |
|  | | |  |  |  |  |  |  |  |  | | | | | | | |
|  | | | | | | | | | | | | 1 |  | BV |  | 15/01/2020 5:10 AM |  |
|  | | | Q: Okay so what would you like to ultimately get out of this trial?  A: Well in two words – good health. | | | | | | | | | | | | | |  |
|  | | |  | | | | | | | | | | | | | |  |
|  | | | | | | | | | | | | | | | | | |
|  | | | | | | | | | | | | | | | | | |
| Formatted Reports\\Coding Summary by File Formatted Report | | | | | | | | | | | Page 99 of 143 | | | | | | |
| 6/10/2022 8:14 AM | | | | | | | | | | | | | | | | | |
|  | | | **Classification** |  | **Aggregate** |  | **Coverage** |  | **Number Of Coding References** |  | | **Reference Number** |  | **Coded By Initials** |  | **Modified On** |  |
|  | | | **Codes\\initial motivation for participation\changes to initial motivation for participation** | | | | | | | | | | | | | |  |
|  |  |  |  |  | No |  | 0.0194 |  | 1 |  | | | | | | |  |
|  | | |  |  |  |  |  |  |  |  | | | | | | | |
|  | | | | | | | | | | | | 1 |  | BV |  | 14/01/2020 1:56 PM |  |
|  | | | Q: I just, my last question for you is X?, looking back on your original motivations for enrolling in this trial, do you feel like these motivations have changed in any way?  A: No not at all. | | | | | | | | | | | | | |  |
|  | | |  | | | | | | | | | | | | | |  |
|  | | | **Codes\\needing more support** | | | | | | | | | | | | | |  |
|  |  |  |  |  | No |  | 0.0296 |  | 1 |  | | | | | | |  |
|  | | |  |  |  |  |  |  |  |  | | | | | | | |
|  | | | | | | | | | | | | 1 |  | BV |  | 14/01/2020 1:55 PM |  |
|  | | | Q: Okay, alright. Any support at home that you require or …-?  A: No I can't, there could be some home help for cleaning.  Q: Okay.  A: So I can do, I'm sure I can do my own.  Q: If that’s something, you know that was an option to you, you'd be accepting it?  A: Yes I probably would. | | | | | | | | | | | | | |  |
|  |  |  |  |  |  |  |  |  |  |  |  |  |  |  |  |  |  |
|  | | |  | | | | | | | | | | | | | |  |
|  | | | **Codes\\PSA numbers going down** | | | | | | | | | | | | | |  |
|  |  |  |  |  | No |  | 0.0806 |  | 2 |  | | | | | | |  |
|  | | |  |  |  |  |  |  |  |  | | | | | | | |
|  | | | | | | | | | | | | 1 |  | BV |  | 14/01/2020 1:55 PM |  |
|  | | | No because my PSA has gone down and down. | | | | | | | | | | | | | |  |
|  | | |  | | | | | | | | | | | | | |  |
|  | | | | | | | | | | | | | | | | | |
|  | | | | | | | | | | | | | | | | | |
|  | | | | | | | | | | | | | | | | | |
| Formatted Reports\\Coding Summary by File Formatted Report | | | | | | | | | | | Page 100 of 143 | | | | | | |
| 6/10/2022 8:14 AM | | | | | | | | | | | | | | | | | |
|  | | | **Classification** |  | **Aggregate** |  | **Coverage** |  | **Number Of Coding References** |  | | **Reference Number** |  | **Coded By Initials** |  | **Modified On** |  |
|  | | | | | | | | | | | | | | | | | |
|  | | | | | | | | | | | | 2 |  | BV |  | 14/01/2020 1:56 PM |  |
|  | | | A: Have I told you about my PSA?  Q: You told me a little bit about it but how’s it doing at the moment?  A: It was 10.5 the last time I had a blood test.  Q: Wow, and what was it when you started?  A: Oh it got up to 2000.  Q: Wow what a big difference.  A: And then it went, I can tell you off the top of my head, it went down to 1000, then it went down to 400, then it went to 90, then to 50, then to 20, and then the last time was 10.5.  Q: Wow, so it's been a progressive change, a drop.  A: Yeah.  Q: How does that make you feel?  A: It makes me feel good, although to be quite honest I didn’t feel better physically when it was 10.5 as compared with say 2000, because when it was 2000 I can't say that I was feeling sick or not well. | | | | | | | | | | | | | |  |
|  |  |  |  |  |  |  |  |  |  |  |  |  |  |  |  |  |  |
|  |  |  |  |  |  |  |  |  |  |  |  |  |  |  |  |  |  |
|  |  |  |  |  |  |  |  |  |  |  |  |  |  |  |  |  |  |
|  | | |  | | | | | | | | | | | | | |  |
|  | | | **Codes\\PSA numbers going down\PSA numbers going down - cabazitaxel** | | | | | | | | | | | | | |  |
|  |  |  |  |  | No |  | 0.0804 |  | 2 |  | | | | | | |  |
|  | | |  |  |  |  |  |  |  |  | | | | | | | |
|  | | | | | | | | | | | | 1 |  | BV |  | 25/03/2020 8:23 AM |  |
|  | | | No because my PSA has gone down and down. | | | | | | | | | | | | | |  |
|  | | |  | | | | | | | | | | | | | |  |
|  | | | | | | | | | | | | 2 |  | BV |  | 25/03/2020 8:23 AM |  |
|  | | | A: Have I told you about my PSA?  Q: You told me a little bit about it but how’s it doing at the moment?  A: It was 10.5 the last time I had a blood test.  Q: Wow, and what was it when you started?  A: Oh it got up to 2000.  Q: Wow what a big difference.  A: And then it went, I can tell you off the top of my head, it went down to 1000, then it went down to 400, then it went to 90, then to 50, then to 20, and then the last time was 10.5.  Q: Wow, so it's been a progressive change, a drop.  A: Yeah.  Q: How does that make you feel?  A: It makes me feel good, although to be quite honest I didn’t feel better physically when it was 10.5 as compared with say 2000, because when it was 2000 I can't say that I was feeling sick or not well. | | | | | | | | | | | | | |  |
|  |  |  |  |  |  |  |  |  |  |  |  |  |  |  |  |  |  |
|  |  |  |  |  |  |  |  |  |  |  |  |  |  |  |  |  |  |
|  |  |  |  |  |  |  |  |  |  |  |  |  |  |  |  |  |  |
|  | | |  | | | | | | | | | | | | | |  |
| Formatted Reports\\Coding Summary by File Formatted Report | | | | | | | | | | | Page 101 of 143 | | | | | | |
| 6/10/2022 8:14 AM | | | | | | | | | | | | | | | | | |
|  | | | **Classification** |  | **Aggregate** |  | **Coverage** |  | **Number Of Coding References** |  | | **Reference Number** |  | **Coded By Initials** |  | **Modified On** |  |
|  | | | **Codes\\quality of life changes** | | | | | | | | | | | | | |  |
|  |  |  |  |  | No |  | 0.0038 |  | 1 |  | | | | | | |  |
|  | | |  |  |  |  |  |  |  |  | | | | | | | |
|  | | | | | | | | | | | | 1 |  | BV |  | 25/03/2020 8:25 AM |  |
|  | | | Any quality of life changes?  A: No. | | | | | | | | | | | | | |  |
|  | | |  | | | | | | | | | | | | | |  |
|  | | | **Codes\\quality of life changes\quality of life changes - cabazitaxel** | | | | | | | | | | | | | |  |
|  |  |  |  |  | No |  | 0.0038 |  | 1 |  | | | | | | |  |
|  | | |  |  |  |  |  |  |  |  | | | | | | | |
|  | | | | | | | | | | | | 1 |  | BV |  | 14/01/2020 1:52 PM |  |
|  | | | Any quality of life changes?  A: No. | | | | | | | | | | | | | |  |
|  | | |  | | | | | | | | | | | | | |  |
|  | | | **Codes\\quality of trialists** | | | | | | | | | | | | | |  |
|  |  |  |  |  | No |  | 0.0066 |  | 1 |  | | | | | | |  |
|  | | |  |  |  |  |  |  |  |  | | | | | | | |
|  | | | | | | | | | | | | 1 |  | BV |  | 14/01/2020 1:53 PM |  |
|  | | | they were good, all the people were good. The meals were terrific | | | | | | | | | | | | | |  |
|  | | |  | | | | | | | | | | | | | |  |
|  | | | **Codes\\side effects** | | | | | | | | | | | | | |  |
|  |  |  |  |  | No |  | 0.0864 |  | 2 |  | | | | | | |  |
|  | | |  |  |  |  |  |  |  |  | | | | | | | |
|  | | | | | | | | | | | | 1 |  | BV |  | 25/03/2020 8:26 AM |  |
|  | | | No it hasn’t. Well could I say not at all.  Q: Okay so no side effects?  A: No side effects, no. | | | | | | | | | | | | | |  |
|  | | |  | | | | | | | | | | | | | |  |
|  | | | | | | | | | | | | | | | | | |
|  | | | | | | | | | | | | | | | | | |
|  | | | | | | | | | | | | | | | | | |
[truncated: 161,805 more chars]
