# Supplementary material for: Experiences of participants in a clinical trial of a novel radioactive treatment for advanced prostate cancer: A nested, qualitative longitudinal study
Source: PLoS One. 2022 Nov 9;17(11):e0276063. doi: 10.1371/journal.pone.0276063 (PMC9645653; doi:10.1371/journal.pone.0276063)
Supplement: S2 File — (DOCX) [file pone.0276063.s002.docx]

| 6/10/2022 8:17 AM | | | | | | | | | | | | | | | | | |
| --- | --- | --- | --- | --- | --- | --- | --- | --- | --- | --- | --- | --- | --- | --- | --- | --- | --- |
| Coding Summary by File | | | | | | | | | | | | | | | | | |
| Coding Follow Up Interviews | | | | | | | | | | | | | | | | | |
| 6/10/2022 8:17 AM | | | | | | | | | | | | | | | | | |
|  | | | **Classification** |  | **Aggregate** |  | **Coverage** |  | **Number Of Coding References** |  | | **Reference Number** |  | **Coded By Initials** |  | **Modified On** |  |
| **Document** | | | | | | | | | | | | | | | | |  |
|  | **Files\\ID002.4** | | | | | | | | | | | | | | | |  |
|  | | **Code** | | | | | | | | | | | | | | |  |
|  | | | **Codes\\currenting receiving other treatment\currently receiving no treatment** | | | | | | | | | | | | | |  |
|  |  |  |  |  | No |  | 0.0206 |  | 1 |  | | | | | | |  |
|  | | |  |  |  |  |  |  |  |  | | | | | | | |
|  | | | | | | | | | | | | 1 |  | BV |  | 25/03/2020 9:28 AM |  |
|  | | | Well there’s nothing happening in, happening in the meantime, but if it doesn’t double well I’ll just be monitored I guess and just in 4 weeks. | | | | | | | | | | | | | |  |
|  | | |  | | | | | | | | | | | | | |  |
|  | | | **Codes\\currenting receiving other treatment\monitoring disease** | | | | | | | | | | | | | |  |
|  |  |  |  |  | No |  | 0.0277 |  | 2 |  | | | | | | |  |
|  | | |  |  |  |  |  |  |  |  | | | | | | | |
|  | | | | | | | | | | | | 1 |  | BV |  | 25/03/2020 9:28 AM |  |
|  | | | Well there’s nothing happening in, happening in the meantime, but if it doesn’t double well I’ll just be monitored I guess and just in 4 weeks. | | | | | | | | | | | | | |  |
|  | | |  | | | | | | | | | | | | | |  |
|  | | | | | | | | | | | | 2 |  | BV |  | 25/03/2020 9:32 AM |  |
|  | | | you’re currently just being monitored?  A: Yes. | | | | | | | | | | | | | |  |
|  | | |  | | | | | | | | | | | | | |  |
|  | | | **Codes\\no additional support required** | | | | | | | | | | | | | |  |
|  |  |  |  |  | No |  | 0.0031 |  | 1 |  | | | | | | |  |
|  | | |  |  |  |  |  |  |  |  | | | | | | | |
|  | | | | | | | | | | | | 1 |  | BV |  | 25/03/2020 9:28 AM |  |
|  | | | No, no I’m … I’m okay. | | | | | | | | | | | | | |  |
|  | | |  | | | | | | | | | | | | | |  |
|  | | | | | | | | | | | | | | | | | |
| Formatted Reports\\Coding Summary by File Formatted Report | | | | | | | | | | | Page 1 of 39 | | | | | | |
| 6/10/2022 8:17 AM | | | | | | | | | | | | | | | | | |
|  | | | **Classification** |  | **Aggregate** |  | **Coverage** |  | **Number Of Coding References** |  | | **Reference Number** |  | **Coded By Initials** |  | **Modified On** |  |
|  | | | **Codes\\not happy about participating\seeking alternative treatment** | | | | | | | | | | | | | |  |
|  |  |  |  |  | No |  | 0.0706 |  | 2 |  | | | | | | |  |
|  | | |  |  |  |  |  |  |  |  | | | | | | | |
|  | | | | | | | | | | | | 1 |  | BV |  | 25/03/2020 9:27 AM |  |
|  | | | A: Well I feel alright, but my PSAs gone up in, the Oncog said if it, if it doubles each time, they’re going to offer me, no so.  Q: They’re going to offer you what sorry?  A: Chemo.  Q: Oh okay.  A: Cabazitaxel. | | | | | | | | | | | | | |  |
|  | | |  |  |  |  |  |  |  |  |  |  |  |  |  |  |  |
|  | | |  | | | | | | | | | | | | | |  |
|  | | | | | | | | | | | | 2 |  | BV |  | 25/03/2020 9:27 AM |  |
|  | | | Q: Oh Cabazitaxel. So when will you be receiving that?  A: Well we’re not sure yet, but I’ve got to go back in 2 weeks’ time and he made the, he’s made the appointment for it. But that’s, if the PSA doesn’t double well, well stays the same I guess, it won’t happen. | | | | | | | | | | | | | |  |
|  | | |  | | | | | | | | | | | | | |  |
|  | | | **Codes\\PSA post treatment\PSA numbers not staying down - lutetium** | | | | | | | | | | | | | |  |
|  |  |  |  |  | No |  | 0.1439 |  | 4 |  | | | | | | |  |
|  | | |  |  |  |  |  |  |  |  | | | | | | | |
|  | | | | | | | | | | | | 1 |  | BV |  | 25/03/2020 9:27 AM |  |
|  | | | A: Well I feel alright, but my PSAs gone up in, the Oncog said if it, if it doubles each time, they’re going to offer me, no so.  Q: They’re going to offer you what sorry?  A: Chemo.  Q: Oh okay.  A: Cabazitaxel. | | | | | | | | | | | | | |  |
|  | | |  |  |  |  |  |  |  |  |  |  |  |  |  |  |  |
|  | | |  | | | | | | | | | | | | | |  |
|  | | | | | | | | | | | | 2 |  | BV |  | 25/03/2020 9:27 AM |  |
|  | | | Q: Oh Cabazitaxel. So when will you be receiving that?  A: Well we’re not sure yet, but I’ve got to go back in 2 weeks’ time and he made the, he’s made the appointment for it. But that’s, if the PSA doesn’t double well, well stays the same I guess, it won’t happen. | | | | | | | | | | | | | |  |
|  | | |  | | | | | | | | | | | | | |  |
|  | | | | | | | | | | | | 3 |  | BV |  | 25/03/2020 9:28 AM |  |
|  | | | Well there’s nothing happening in, happening in the meantime, but if it doesn’t double well I’ll just be monitored I guess and just in 4 weeks. | | | | | | | | | | | | | |  |
|  | | |  | | | | | | | | | | | | | |  |
|  | | | | | | | | | | | | 4 |  | BV |  | 25/03/2020 9:32 AM |  |
|  | | | A: … (Talking over each other). It was yes, but it went down with the trial, it went right down to 2.5.  Q: Oh that was even better isn’t it?  A: Yeah then it went up to 3, and in the next 4 weeks it doubled to 6. And that’s what is, what’s saying it looks like it’s on the way up again and if it doubles again he said, well they’ll offer me the Cabazitaxel. | | | | | | | | | | | | | |  |
|  | | |  | | | | | | | | | | | | | |  |
|  | | | | | | | | | | | | | | | | | |
| Formatted Reports\\Coding Summary by File Formatted Report | | | | | | | | | | | Page 2 of 39 | | | | | | |
| 6/10/2022 8:17 AM | | | | | | | | | | | | | | | | | |
|  | | | **Classification** |  | **Aggregate** |  | **Coverage** |  | **Number Of Coding References** |  | | **Reference Number** |  | **Coded By Initials** |  | **Modified On** |  |
|  | | | **Codes\\x - sociodemographic data\children** | | | | | | | | | | | | | |  |
|  |  |  |  |  | No |  | 0.0010 |  | 1 |  | | | | | | |  |
|  | | |  |  |  |  |  |  |  |  | | | | | | | |
|  | | | | | | | | | | | | 1 |  | BV |  | 25/03/2020 9:30 AM |  |
|  | | | 5 sons. | | | | | | | | | | | | | |  |
|  | | |  | | | | | | | | | | | | | |  |
|  | | | **Codes\\x - sociodemographic data\country born** | | | | | | | | | | | | | |  |
|  |  |  |  |  | No |  | 0.0017 |  | 1 |  | | | | | | |  |
|  | | |  |  |  |  |  |  |  |  | | | | | | | |
|  | | | | | | | | | | | | 1 |  | BV |  | 25/03/2020 9:30 AM |  |
|  | | | Australia. | | | | | | | | | | | | | |  |
|  | | |  | | | | | | | | | | | | | |  |
|  | | | **Codes\\x - sociodemographic data\current PSA** | | | | | | | | | | | | | |  |
|  |  |  |  |  | No |  | 0.0602 |  | 2 |  | | | | | | |  |
|  | | |  |  |  |  |  |  |  |  | | | | | | | |
|  | | | | | | | | | | | | 1 |  | BV |  | 25/03/2020 9:32 AM |  |
|  | | | Well it was up to at 6 last time, that’s 2 weeks ago. | | | | | | | | | | | | | |  |
|  | | |  | | | | | | | | | | | | | |  |
|  | | | | | | | | | | | | 2 |  | BV |  | 25/03/2020 9:32 AM |  |
|  | | | A: … (Talking over each other). It was yes, but it went down with the trial, it went right down to 2.5.  Q: Oh that was even better isn’t it?  A: Yeah then it went up to 3, and in the next 4 weeks it doubled to 6. And that’s what is, what’s saying it looks like it’s on the way up again and if it doubles again he said, well they’ll offer me the Cabazitaxel. | | | | | | | | | | | | | |  |
|  | | |  | | | | | | | | | | | | | |  |
|  | | | **Codes\\x - sociodemographic data\DOB** | | | | | | | | | | | | | |  |
|  |  |  |  |  | No |  | 0.0021 |  | 1 |  | | | | | | |  |
|  | | |  |  |  |  |  |  |  |  | | | | | | | |
|  | | | | | | | | | | | | 1 |  | BV |  | 25/03/2020 9:29 AM |  |
|  | | | 29th Feb, 1945. | | | | | | | | | | | | | |  |
|  | | |  | | | | | | | | | | | | | |  |
|  | | | **Codes\\x - sociodemographic data\education** | | | | | | | | | | | | | |  |
|  |  |  |  |  | No |  | 0.0011 |  | 1 |  | | | | | | |  |
|  | | |  |  |  |  |  |  |  |  | | | | | | | |
|  | | | | | | | | | | | | 1 |  | BV |  | 25/03/2020 9:30 AM |  |
|  | | | Diploma. | | | | | | | | | | | | | |  |
|  | | |  | | | | | | | | | | | | | |  |
| Formatted Reports\\Coding Summary by File Formatted Report | | | | | | | | | | | Page 3 of 39 | | | | | | |
| 6/10/2022 8:17 AM | | | | | | | | | | | | | | | | | |
|  | | | **Classification** |  | **Aggregate** |  | **Coverage** |  | **Number Of Coding References** |  | | **Reference Number** |  | **Coded By Initials** |  | **Modified On** |  |
|  | | | **Codes\\x - sociodemographic data\first diagnosed** | | | | | | | | | | | | | |  |
|  |  |  |  |  | No |  | 0.0060 |  | 2 |  | | | | | | |  |
|  | | |  |  |  |  |  |  |  |  | | | | | | | |
|  | | | | | | | | | | | | 1 |  | BV |  | 25/03/2020 9:31 AM |  |
|  | | | October 2011. | | | | | | | | | | | | | |  |
|  | | |  | | | | | | | | | | | | | |  |
|  | | | | | | | | | | | | 2 |  | BV |  | 25/03/2020 9:31 AM |  |
|  | | | advanced cancer straightaway. | | | | | | | | | | | | | |  |
|  | | |  | | | | | | | | | | | | | |  |
|  | | | **Codes\\x - sociodemographic data\gleason score** | | | | | | | | | | | | | |  |
|  |  |  |  |  | No |  | 0.0033 |  | 1 |  | | | | | | |  |
|  | | |  |  |  |  |  |  |  |  | | | | | | | |
|  | | | | | | | | | | | | 1 |  | BV |  | 25/03/2020 9:31 AM |  |
|  | | | I think it was 3 and 4. | | | | | | | | | | | | | |  |
|  | | |  | | | | | | | | | | | | | |  |
|  | | | **Codes\\x - sociodemographic data\income** | | | | | | | | | | | | | |  |
|  |  |  |  |  | No |  | 0.0031 |  | 1 |  | | | | | | |  |
|  | | |  |  |  |  |  |  |  |  | | | | | | | |
|  | | | | | | | | | | | | 1 |  | BV |  | 25/03/2020 9:30 AM |  |
|  | | | Oh I’m a TTI pensioner | | | | | | | | | | | | | |  |
|  | | |  | | | | | | | | | | | | | |  |
|  | | | **Codes\\x - sociodemographic data\marital status** | | | | | | | | | | | | | |  |
|  |  |  |  |  | No |  | 0.0049 |  | 2 |  | | | | | | |  |
|  | | |  |  |  |  |  |  |  |  | | | | | | | |
|  | | | | | | | | | | | | 1 |  | BV |  | 25/03/2020 9:29 AM |  |
|  | | | Married | | | | | | | | | | | | | |  |
|  | | |  | | | | | | | | | | | | | |  |
|  | | | | | | | | | | | | 2 |  | BV |  | 25/03/2020 9:30 AM |  |
|  | | | No my wife has Alzheimer’s. | | | | | | | | | | | | | |  |
|  | | |  | | | | | | | | | | | | | |  |
|  | | | **Codes\\x - sociodemographic data\previous treatment** | | | | | | | | | | | | | |  |
|  |  |  |  |  | No |  | 0.0141 |  | 5 |  | | | | | | |  |
|  | | |  |  |  |  |  |  |  |  | | | | | | | |
|  | | | | | | | | | | | | 1 |  | BV |  | 25/03/2020 9:31 AM |  |
|  | | | Zoladex and I’m still on Zoladex that started in 2012. | | | | | | | | | | | | | |  |
|  | | |  | | | | | | | | | | | | | |  |
| Formatted Reports\\Coding Summary by File Formatted Report | | | | | | | | | | | Page 4 of 39 | | | | | | |
| 6/10/2022 8:17 AM | | | | | | | | | | | | | | | | | |
|  | | | **Classification** |  | **Aggregate** |  | **Coverage** |  | **Number Of Coding References** |  | | **Reference Number** |  | **Coded By Initials** |  | **Modified On** |  |
|  | | | | | | | | | | | | | | | | | |
|  | | | | | | | | | | | | 2 |  | BV |  | 25/03/2020 9:31 AM |  |
|  | | | I had chemotherapy. | | | | | | | | | | | | | |  |
|  | | |  | | | | | | | | | | | | | |  |
|  | | | | | | | | | | | | 3 |  | BV |  | 25/03/2020 9:31 AM |  |
|  | | | Docetaxel. | | | | | | | | | | | | | |  |
|  | | |  | | | | | | | | | | | | | |  |
|  | | | | | | | | | | | | 4 |  | BV |  | 25/03/2020 9:31 AM |  |
|  | | | Casodex- | | | | | | | | | | | | | |  |
|  | | |  | | | | | | | | | | | | | |  |
|  | | | | | | | | | | | | 5 |  | BV |  | 25/03/2020 9:32 AM |  |
|  | | | Zytiga | | | | | | | | | | | | | |  |
|  | | |  | | | | | | | | | | | | | |  |
|  | | | **Codes\\x - sociodemographic data\residency** | | | | | | | | | | | | | |  |
|  |  |  |  |  | No |  | 0.0117 |  | 2 |  | | | | | | |  |
|  | | |  |  |  |  |  |  |  |  | | | | | | | |
|  | | | | | | | | | | | | 1 |  | BV |  | 25/03/2020 9:29 AM |  |
|  | | | We’ve actually, we’ve actually moved up to my son’s place at Maroochy River. | | | | | | | | | | | | | |  |
|  | | |  | | | | | | | | | | | | | |  |
|  | | | | | | | | | | | | 2 |  | BV |  | 25/03/2020 9:29 AM |  |
|  | | | 4162. | | | | | | | | | | | | | |  |
|  | | |  | | | | | | | | | | | | | |  |
|  | | | **Codes\\x - sociodemographic data\work status** | | | | | | | | | | | | | |  |
|  |  |  |  |  | No |  | 0.0017 |  | 1 |  | | | | | | |  |
|  | | |  |  |  |  |  |  |  |  | | | | | | | |
|  | | | | | | | | | | | | 1 |  | BV |  | 25/03/2020 9:30 AM |  |
|  | | | I’m retired. | | | | | | | | | | | | | |  |
|  | | |  | | | | | | | | | | | | | |  |
|  | | | | | | | | | | | | | | | | | |
|  | | | | | | | | | | | | | | | | | |
| Formatted Reports\\Coding Summary by File Formatted Report | | | | | | | | | | | Page 5 of 39 | | | | | | |
| 6/10/2022 8:17 AM | | | | | | | | | | | | | | | | | |
|  | | | **Classification** |  | **Aggregate** |  | **Coverage** |  | **Number Of Coding References** |  | | **Reference Number** |  | **Coded By Initials** |  | **Modified On** |  |
|  | | | **Codes\\x - sociodemographic data\years married** | | | | | | | | | | | | | |  |
|  |  |  |  |  | No |  | 0.0015 |  | 1 |  | | | | | | |  |
|  | | |  |  |  |  |  |  |  |  | | | | | | | |
|  | | | | | | | | | | | | 1 |  | BV |  | 25/03/2020 9:30 AM |  |
|  | | | 51 years. | | | | | | | | | | | | | |  |
|  | | |  | | | | | | | | | | | | | |  |
|  | **Files\\ID008.4** | | | | | | | | | | | | | | | |  |
|  | | **Code** | | | | | | | | | | | | | | |  |
|  | | | **Codes\\new additional health concerns** | | | | | | | | | | | | | |  |
|  |  |  |  |  | No |  | 0.1338 |  | 2 |  | | | | | | |  |
|  | | |  |  |  |  |  |  |  |  | | | | | | | |
|  | | | | | | | | | | | | 1 |  | BV |  | 25/03/2020 9:42 AM |  |
|  | | | A: Well, first of all, the PSA has started to go up again. It went down to 12, now it’s 151 and going up. And there’s nothing that I can be offered. They were the words of the oncologist. But as well as that, I’ve got a melanoma-  Q: Oh, goodness.  A: The liver – the liver, in one of the lymph nodes. It’s aggressive, and they can’t treat one, the melanoma or the prostate cancer, because the other one will grow. And I was told that the oncologist was uncertain as to whether the melanoma treatment would work. There’s only a 10-15 chance of severe – there is a 10-15 chance of severe side effects. That’s all I can tell you. And the timeframe for me would be about 6-9 months. | | | | | | | | | | | | | |  |
|  | | |  |  |  |  |  |  |  |  |  |  |  |  |  |  |  |
|  | | |  | | | | | | | | | | | | | |  |
|  | | | | | | | | | | | | 2 |  | BV |  | 25/03/2020 9:43 AM |  |
|  | | | you can’t treat one, like the prostate cancer, because the other one will grow. | | | | | | | | | | | | | |  |
|  | | |  | | | | | | | | | | | | | |  |
|  | | | **Codes\\palliative care** | | | | | | | | | | | | | |  |
|  |  |  |  |  | No |  | 0.0100 |  | 1 |  | | | | | | |  |
|  | | |  |  |  |  |  |  |  |  | | | | | | | |
|  | | | | | | | | | | | | 1 |  | BV |  | 25/03/2020 9:43 AM |  |
|  | | | But the palliative care people have been in touch with me. | | | | | | | | | | | | | |  |
|  | | |  | | | | | | | | | | | | | |  |
|  | | | **Codes\\PSA post treatment\PSA numbers not staying down - cabazitaxel** | | | | | | | | | | | | | |  |
|  |  |  |  |  | No |  | 0.0650 |  | 2 |  | | | | | | |  |
|  | | |  |  |  |  |  |  |  |  | | | | | | | |
|  | | | | | | | | | | | | 1 |  | BV |  | 25/03/2020 9:41 AM |  |
|  | | | Well, first of all, the PSA has started to go up again. It went down to 12, now it’s 151 and going up. And there’s nothing that I can be offered. They were the words of the oncologist. But as well as that, I’ve got a melanoma- | | | | | | | | | | | | | |  |
|  | | |  | | | | | | | | | | | | | |  |
| Formatted Reports\\Coding Summary by File Formatted Report | | | | | | | | | | | Page 6 of 39 | | | | | | |
| 6/10/2022 8:17 AM | | | | | | | | | | | | | | | | | |
|  | | | **Classification** |  | **Aggregate** |  | **Coverage** |  | **Number Of Coding References** |  | | **Reference Number** |  | **Coded By Initials** |  | **Modified On** |  |
|  | | | | | | | | | | | | | | | | | |
|  | | | | | | | | | | | | 2 |  | BV |  | 25/03/2020 9:42 AM |  |
|  | | | 151 - it was down to 12, now it’s on the up, and there’s nothing to offer me in the way of that, because it would react against the melanoma, and- | | | | | | | | | | | | | |  |
|  | | |  | | | | | | | | | | | | | |  |
|  | | | **Codes\\x - sociodemographic data\country born** | | | | | | | | | | | | | |  |
|  |  |  |  |  | No |  | 0.0100 |  | 1 |  | | | | | | |  |
|  | | |  |  |  |  |  |  |  |  | | | | | | | |
|  | | | | | | | | | | | | 1 |  | BV |  | 25/03/2020 9:44 AM |  |
|  | | | Q: Year 11. Were you born in Australia?  A: Yes, I was. | | | | | | | | | | | | | |  |
|  | | |  | | | | | | | | | | | | | |  |
|  | | | **Codes\\x - sociodemographic data\current PSA** | | | | | | | | | | | | | |  |
|  |  |  |  |  | No |  | 0.0252 |  | 1 |  | | | | | | |  |
|  | | |  |  |  |  |  |  |  |  | | | | | | | |
|  | | | | | | | | | | | | 1 |  | BV |  | 25/03/2020 9:43 AM |  |
|  | | | 151 - it was down to 12, now it’s on the up, and there’s nothing to offer me in the way of that, because it would react against the melanoma, and- | | | | | | | | | | | | | |  |
|  | | |  | | | | | | | | | | | | | |  |
|  | | | **Codes\\x - sociodemographic data\DOB** | | | | | | | | | | | | | |  |
|  |  |  |  |  | No |  | 0.0034 |  | 1 |  | | | | | | |  |
|  | | |  |  |  |  |  |  |  |  | | | | | | | |
|  | | | | | | | | | | | | 1 |  | BV |  | 25/03/2020 9:44 AM |  |
|  | | | 16-8 – August, 1935. | | | | | | | | | | | | | |  |
|  | | |  | | | | | | | | | | | | | |  |
|  | | | **Codes\\x - sociodemographic data\education** | | | | | | | | | | | | | |  |
|  |  |  |  |  | No |  | 0.0013 |  | 1 |  | | | | | | |  |
|  | | |  |  |  |  |  |  |  |  | | | | | | | |
|  | | | | | | | | | | | | 1 |  | BV |  | 25/03/2020 9:44 AM |  |
|  | | | Year 11. | | | | | | | | | | | | | |  |
|  | | |  | | | | | | | | | | | | | |  |
|  | | | **Codes\\x - sociodemographic data\first diagnosed** | | | | | | | | | | | | | |  |
|  |  |  |  |  | No |  | 0.0195 |  | 1 |  | | | | | | |  |
|  | | |  |  |  |  |  |  |  |  | | | | | | | |
|  | | | | | | | | | | | | 1 |  | BV |  | 25/03/2020 9:44 AM |  |
|  | | | Q: That’s okay. Do you remember what the date was when you were first diagnosed?  A: No, no, I can’t remember. | | | | | | | | | | | | | |  |
|  | | |  | | | | | | | | | | | | | |  |
| Formatted Reports\\Coding Summary by File Formatted Report | | | | | | | | | | | Page 7 of 39 | | | | | | |
| 6/10/2022 8:17 AM | | | | | | | | | | | | | | | | | |
|  | | | **Classification** |  | **Aggregate** |  | **Coverage** |  | **Number Of Coding References** |  | | **Reference Number** |  | **Coded By Initials** |  | **Modified On** |  |
|  | | | **Codes\\x - sociodemographic data\gleason score** | | | | | | | | | | | | | |  |
|  |  |  |  |  | No |  | 0.0086 |  | 1 |  | | | | | | |  |
|  | | |  |  |  |  |  |  |  |  | | | | | | | |
|  | | | | | | | | | | | | 1 |  | BV |  | 25/03/2020 9:45 AM |  |
|  | | | Q: Or your Gleeson score, sorry.  A: No, no, no. | | | | | | | | | | | | | |  |
|  | | |  | | | | | | | | | | | | | |  |
|  | | | **Codes\\x - sociodemographic data\marital status** | | | | | | | | | | | | | |  |
|  |  |  |  |  | No |  | 0.0012 |  | 1 |  | | | | | | |  |
|  | | |  |  |  |  |  |  |  |  | | | | | | | |
|  | | | | | | | | | | | | 1 |  | BV |  | 25/03/2020 9:44 AM |  |
|  | | | Single. | | | | | | | | | | | | | |  |
|  | | |  | | | | | | | | | | | | | |  |
|  | | | **Codes\\x - sociodemographic data\previous treatment** | | | | | | | | | | | | | |  |
|  |  |  |  |  | No |  | 0.1206 |  | 2 |  | | | | | | |  |
|  | | |  |  |  |  |  |  |  |  | | | | | | | |
|  | | | | | | | | | | | | 1 |  | BV |  | 25/03/2020 9:45 AM |  |
|  | | | No, I can’t answer that. I don’t know. | | | | | | | | | | | | | |  |
|  | | |  | | | | | | | | | | | | | |  |
|  | | | | | | | | | | | | 2 |  | BV |  | 25/03/2020 9:45 AM |  |
|  | | | A: And as well as that, I had an operation for the right neck dissection. There was a growth in the right side of the neck.  Q: Okay. Have you had your prostate gland removed?  A: Yes.  Q: Yes. Do you remember what type of surgery that was? Did they make an open incision below your belly-button, or-?  A: No, no, it was an operation.  Q: Yeah, so did they cut-?  A: It was 2010.  Q: In 2010, yes. Did they do it with a camera, or did they make an incision on your belly-?  A: No, no, no – no, camera, no.  Q: Is there an incision in your belly, or bottom?  A: There is, but it’s not from that, it’s from the anti-reflux operation. | | | | | | | | | | | | | |  |
|  | | |  |  |  |  |  |  |  |  |  |  |  |  |  |  |  |
|  | | |  |  |  |  |  |  |  |  |  |  |  |  |  |  |  |
|  | | |  |  |  |  |  |  |  |  |  |  |  |  |  |  |  |
|  | | |  | | | | | | | | | | | | | |  |
|  | | | | | | | | | | | | | | | | | |
| Formatted Reports\\Coding Summary by File Formatted Report | | | | | | | | | | | Page 8 of 39 | | | | | | |
| 6/10/2022 8:17 AM | | | | | | | | | | | | | | | | | |
|  | | | **Classification** |  | **Aggregate** |  | **Coverage** |  | **Number Of Coding References** |  | | **Reference Number** |  | **Coded By Initials** |  | **Modified On** |  |
|  | | | **Codes\\x - sociodemographic data\residency** | | | | | | | | | | | | | |  |
|  |  |  |  |  | No |  | 0.0015 |  | 1 |  | | | | | | |  |
|  | | |  |  |  |  |  |  |  |  | | | | | | | |
|  | | | | | | | | | | | | 1 |  | BV |  | 25/03/2020 9:44 AM |  |
|  | | | Victoria. | | | | | | | | | | | | | |  |
|  | | |  | | | | | | | | | | | | | |  |
|  | | | **Codes\\x - sociodemographic data\work status** | | | | | | | | | | | | | |  |
|  |  |  |  |  | No |  | 0.0102 |  | 1 |  | | | | | | |  |
|  | | |  |  |  |  |  |  |  |  | | | | | | | |
|  | | | | | | | | | | | | 1 |  | BV |  | 25/03/2020 9:44 AM |  |
|  | | | I’m – I’ve retired from teaching, but I’m still tutoring. | | | | | | | | | | | | | |  |
|  | | |  | | | | | | | | | | | | | |  |
|  | **Files\\ID009.4** | | | | | | | | | | | | | | | |  |
|  | | **Code** | | | | | | | | | | | | | | |  |
|  | | | **Codes\\currenting receiving other treatment** | | | | | | | | | | | | | |  |
|  |  |  |  |  | No |  | 0.0049 |  | 3 |  | | | | | | |  |
|  | | |  |  |  |  |  |  |  |  | | | | | | | |
|  | | | | | | | | | | | | 1 |  | BV |  | 25/03/2020 9:55 AM |  |
|  | | | Only Zoladex | | | | | | | | | | | | | |  |
|  | | |  | | | | | | | | | | | | | |  |
|  | | | | | | | | | | | | 2 |  | BV |  | 25/03/2020 9:55 AM |  |
|  | | | I think the Zoladex is enough I think. | | | | | | | | | | | | | |  |
|  | | |  | | | | | | | | | | | | | |  |
|  | | | | | | | | | | | | 3 |  | BV |  | 25/03/2020 9:55 AM |  |
|  | | | That’s my opinion, that’s chemical castration right? | | | | | | | | | | | | | |  |
|  | | |  | | | | | | | | | | | | | |  |
|  | | | **Codes\\emotional impact** | | | | | | | | | | | | | |  |
|  |  |  |  |  | No |  | 0.0434 |  | 2 |  | | | | | | |  |
|  | | |  |  |  |  |  |  |  |  | | | | | | | |
|  | | | | | | | | | | | | 1 |  | BV |  | 25/03/2020 9:53 AM |  |
|  | | | Oh good, but I’m – I’ve never been, I’ve never really been down. I got a bit down, I think I got a bit down with (wife) at the start you know, I thought she could of tried a little harder at the really the beginning, but she probably couldn’t because she was in, she was … (Unable to understand). So, I was and I probably got a bit yeah, I guess I got a bit, maybe a bit stressed, you know? Yeah, so yeah. | | | | | | | | | | | | | |  |
|  | | |  | | | | | | | | | | | | | |  |
| Formatted Reports\\Coding Summary by File Formatted Report | | | | | | | | | | | Page 9 of 39 | | | | | | |
| 6/10/2022 8:17 AM | | | | | | | | | | | | | | | | | |
|  | | | **Classification** |  | **Aggregate** |  | **Coverage** |  | **Number Of Coding References** |  | | **Reference Number** |  | **Coded By Initials** |  | **Modified On** |  |
|  | | | | | | | | | | | | | | | | | |
|  | | | | | | | | | | | | 2 |  | BV |  | 25/03/2020 9:53 AM |  |
|  | | | No, well I’m not really worried, I look I expected it to go up which it was, would have to be the last so I had 3 weeks ago and then 6 weeks so, 9 weeks, 9 yeah, … (Unable to understand) when I take it. So 9 or 12 weeks ago when my PSA only went down a little bit to 22, I knew then that even though they – I … (Unable to understand) anyway because they – no, they’re doctors and fair enough, I’ve got no, I have no medical experience or and nothing, but I just know me you know, so. | | | | | | | | | | | | | |  |
|  | | |  | | | | | | | | | | | | | |  |
|  | | | **Codes\\emotional impact\coping strategies** | | | | | | | | | | | | | |  |
|  |  |  |  |  | No |  | 0.0198 |  | 1 |  | | | | | | |  |
|  | | |  |  |  |  |  |  |  |  | | | | | | | |
|  | | | | | | | | | | | | 1 |  | BV |  | 25/03/2020 9:53 AM |  |
|  | | | Oh good, but I’m – I’ve never been, I’ve never really been down. I got a bit down, I think I got a bit down with (wife) at the start you know, I thought she could of tried a little harder at the really the beginning, but she probably couldn’t because she was in, she was … (Unable to understand). So, I was and I probably got a bit yeah, I guess I got a bit, maybe a bit stressed, you know? Yeah, so yeah. | | | | | | | | | | | | | |  |
|  | | |  | | | | | | | | | | | | | |  |
|  | | | **Codes\\facing mortality** | | | | | | | | | | | | | |  |
|  |  |  |  |  | No |  | 0.0052 |  | 1 |  | | | | | | |  |
|  | | |  |  |  |  |  |  |  |  | | | | | | | |
|  | | | | | | | | | | | | 1 |  | BV |  | 25/03/2020 9:55 AM |  |
|  | | | Q: Alrighty. If you don’t get the last treatment, do you know roughly what will happen?  A: Probably die. | | | | | | | | | | | | | |  |
|  | | |  | | | | | | | | | | | | | |  |
|  | | | **Codes\\hope\staying positive** | | | | | | | | | | | | | |  |
|  |  |  |  |  | No |  | 0.0140 |  | 2 |  | | | | | | |  |
|  | | |  |  |  |  |  |  |  |  | | | | | | | |
|  | | | | | | | | | | | | 1 |  | BV |  | 25/03/2020 9:59 AM |  |
|  | | | Yeah, I - well I obviously do because I’ve always tried my best and I’m always looked on the bright side and all that you know, I’ve never been – I never walked around the house saying, (wife), yeah I’m buggered you know, blah-blah. | | | | | | | | | | | | | |  |
|  | | |  | | | | | | | | | | | | | |  |
|  | | | | | | | | | | | | 2 |  | BV |  | 25/03/2020 10:00 AM |  |
|  | | | I’ve always been positive, always been positive yeah, so. | | | | | | | | | | | | | |  |
|  | | |  | | | | | | | | | | | | | |  |
|  | | | **Codes\\needing to support partner** | | | | | | | | | | | | | |  |
|  |  |  |  |  | No |  | 0.0528 |  | 3 |  | | | | | | |  |
|  | | |  |  |  |  |  |  |  |  | | | | | | | |
|  | | | | | | | | | | | | 1 |  | BV |  | 25/03/2020 9:52 AM |  |
|  | | | Q: You’ve had to support one another over the past-  A: Well, yes I-  Q: For a long time.  A: We did, I had to sort of take over everything, but I’m a lousy cook, thank god she’s cooking again otherwise we’d all die of starvation. But I’m lousy, I’m a … – I have sort of – if I don’t have any interest in it, I just can’t sort of put your heart and soul behind it you know? | | | | | | | | | | | | | |  |
|  | | |  |  |  |  |  |  |  |  |  |  |  |  |  |  |  |
|  | | |  | | | | | | | | | | | | | |  |
| Formatted Reports\\Coding Summary by File Formatted Report | | | | | | | | | | | Page 10 of 39 | | | | | | |
| 6/10/2022 8:17 AM | | | | | | | | | | | | | | | | | |
|  | | | **Classification** |  | **Aggregate** |  | **Coverage** |  | **Number Of Coding References** |  | | **Reference Number** |  | **Coded By Initials** |  | **Modified On** |  |
|  | | | | | | | | | | | | | | | | | |
|  | | | | | | | | | | | | 2 |  | BV |  | 25/03/2020 9:53 AM |  |
|  | | | Oh good, but I’m – I’ve never been, I’ve never really been down. I got a bit down, I think I got a bit down with (wife) at the start you know, I thought she could of tried a little harder at the really the beginning, but she probably couldn’t because she was in, she was … (Unable to understand). So, I was and I probably got a bit yeah, I guess I got a bit, maybe a bit stressed, you know? Yeah, so yeah. | | | | | | | | | | | | | |  |
|  | | |  | | | | | | | | | | | | | |  |
|  | | | | | | | | | | | | 3 |  | BV |  | 25/03/2020 9:53 AM |  |
|  | | | No, I’m alright, no. I always look after myself, I don’t – if you have other people sort of, you’ve got to rely on other people, instead of just, doing your thing you know, willy-nilly you’ve got to sort of give in without that. At the moment we’re going to handle everything ourselves so, yeah. | | | | | | | | | | | | | |  |
|  | | |  | | | | | | | | | | | | | |  |
|  | | | **Codes\\needing to support partner\partner unwell** | | | | | | | | | | | | | |  |
|  |  |  |  |  | No |  | 0.0435 |  | 2 |  | | | | | | |  |
|  | | |  |  |  |  |  |  |  |  | | | | | | | |
|  | | | | | | | | | | | | 1 |  | BV |  | 25/03/2020 9:52 AM |  |
|  | | | well the whole thing is a change of life, is you can’t plan on nothing and it’s been a total I don’t know, whatever you call it. The early part was not too bad because we were caravanners'. We can’t go away like we used to you know, for a month a long time. Yeah and I’ve actually sold it; I’ve sold my van because I thought because (wife) actually caught – got to, I don’t know whether you catch it or not, but she got melanoma and that was back about 6 months ago and we thought or Judy did anyway, that she was going to die. And I dare say, and I actually had faith that I thought she would survive and but most people thought well that’s it for Jude. But she’s now on nanotherapy and it is sort of working really well. | | | | | | | | | | | | | |  |
|  | | |  | | | | | | | | | | | | | |  |
|  | | | | | | | | | | | | 2 |  | BV |  | 25/03/2020 9:52 AM |  |
|  | | | So she’s improved, she lost a lot of weight, but she hasn’t gained all her weight back because she was always a small person anyway; she used to weigh about 50 kilos. | | | | | | | | | | | | | |  |
|  | | |  | | | | | | | | | | | | | |  |
|  | | | **Codes\\no additional support required** | | | | | | | | | | | | | |  |
|  |  |  |  |  | No |  | 0.0144 |  | 1 |  | | | | | | |  |
|  | | |  |  |  |  |  |  |  |  | | | | | | | |
|  | | | | | | | | | | | | 1 |  | BV |  | 25/03/2020 9:53 AM |  |
|  | | | No, I’m alright, no. I always look after myself, I don’t – if you have other people sort of, you’ve got to rely on other people, instead of just, doing your thing you know, willy-nilly you’ve got to sort of give in without that. At the moment we’re going to handle everything ourselves so, yeah. | | | | | | | | | | | | | |  |
|  | | |  | | | | | | | | | | | | | |  |
|  | | | **Codes\\no cure** | | | | | | | | | | | | | |  |
|  |  |  |  |  | No |  | 0.0139 |  | 1 |  | | | | | | |  |
|  | | |  |  |  |  |  |  |  |  | | | | | | | |
|  | | | | | | | | | | | | 1 |  | BV |  | 25/03/2020 9:59 AM |  |
|  | | | Yeah, well I hope something – I have a cure you see, there’s no cure; no cure for prostate cancer, no matter what they do to you, that doesn’t cure it you know, so. Now I think with cancer it gets stronger and stronger you know, and that’s when the drugs can’t kill a lot you know, so. | | | | | | | | | | | | | |  |
|  | | |  | | | | | | | | | | | | | |  |
|  | | | **Codes\\not happy about participating** | | | | | | | | | | | | | |  |
|  |  |  |  |  | No |  | 0.0059 |  | 1 |  | | | | | | |  |
|  | | |  |  |  |  |  |  |  |  | | | | | | | |
|  | | | | | | | | | | | | 1 |  | BV |  | 25/03/2020 9:58 AM |  |
|  | | | Well, no I’m not sad, I’m not thing, but the only thing is, I’m probably disappointed it never lasted longer that’s all. | | | | | | | | | | | | | |  |
|  | | |  | | | | | | | | | | | | | |  |
| Formatted Reports\\Coding Summary by File Formatted Report | | | | | | | | | | | Page 11 of 39 | | | | | | |
| 6/10/2022 8:17 AM | | | | | | | | | | | | | | | | | |
|  | | | **Classification** |  | **Aggregate** |  | **Coverage** |  | **Number Of Coding References** |  | | **Reference Number** |  | **Coded By Initials** |  | **Modified On** |  |
|  | | | **Codes\\not happy about participating\dissapointed** | | | | | | | | | | | | | |  |
|  |  |  |  |  | No |  | 0.0416 |  | 2 |  | | | | | | |  |
|  | | |  |  |  |  |  |  |  |  | | | | | | | |
|  | | | | | | | | | | | | 1 |  | BV |  | 25/03/2020 9:49 AM |  |
|  | | | Yeah, so it’s gone up 3 times. When it went to 22, I – normally I know how I operate here because I’ve had that many things and when you start up a new thing it goes down very quickly, boom-boom-boom-boom. When it got to 22, I’m not too sure what the figure was prior to going to 22, but I’d said to him, I said that’s no good. I said I’m – this drug is finished you know, it’s not going to do anymore. Oh he said it come down, I said yeah, but it didn’t come down enough so, when it sort of comes down sort of slows up, plateaus and then goes back up, you know. And they keep saying oh, we look at more things than blood, but-  Q: Okay.  A: It’s PSA to me is what is you know, where the cancer is, that’s my opinion anyway. | | | | | | | | | | | | | |  |
|  | | |  |  |  |  |  |  |  |  |  |  |  |  |  |  |  |
|  | | |  | | | | | | | | | | | | | |  |
|  | | | | | | | | | | | | 2 |  | BV |  | 25/03/2020 9:58 AM |  |
|  | | | Well, no I’m not sad, I’m not thing, but the only thing is, I’m probably disappointed it never lasted longer that’s all. | | | | | | | | | | | | | |  |
|  | | |  | | | | | | | | | | | | | |  |
|  | | | **Codes\\PSA post treatment\PSA numbers not staying down - lutetium** | | | | | | | | | | | | | |  |
|  |  |  |  |  | No |  | 0.0701 |  | 3 |  | | | | | | |  |
|  | | |  |  |  |  |  |  |  |  | | | | | | | |
|  | | | | | | | | | | | | 1 |  | BV |  | 25/03/2020 9:49 AM |  |
|  | | | A: My PSA come down, down, down, got down to 22, then the next month or 6 weeks, about to 31 or something like that.  Q: Okay.  A: And then it went from a 31 to 48 and the other day, I went in last Tuesday and it’s 70. | | | | | | | | | | | | | |  |
|  | | |  | | | | | | | | | | | | | |  |
|  | | | | | | | | | | | | 2 |  | BV |  | 25/03/2020 9:49 AM |  |
|  | | | Yeah, so it’s gone up 3 times. When it went to 22, I – normally I know how I operate here because I’ve had that many things and when you start up a new thing it goes down very quickly, boom-boom-boom-boom. When it got to 22, I’m not too sure what the figure was prior to going to 22, but I’d said to him, I said that’s no good. I said I’m – this drug is finished you know, it’s not going to do anymore. Oh he said it come down, I said yeah, but it didn’t come down enough so, when it sort of comes down sort of slows up, plateaus and then goes back up, you know. And they keep saying oh, we look at more things than blood, but-  Q: Okay.  A: It’s PSA to me is what is you know, where the cancer is, that’s my opinion anyway. | | | | | | | | | | | | | |  |
|  | | |  |  |  |  |  |  |  |  |  |  |  |  |  |  |  |
|  | | |  | | | | | | | | | | | | | |  |
|  | | | | | | | | | | | | 3 |  | BV |  | 25/03/2020 9:53 AM |  |
|  | | | No, well I’m not really worried, I look I expected it to go up which it was, would have to be the last so I had 3 weeks ago and then 6 weeks so, 9 weeks, 9 yeah, … (Unable to understand) when I take it. So 9 or 12 weeks ago when my PSA only went down a little bit to 22, I knew then that even though they – I … (Unable to understand) anyway because they – no, they’re doctors and fair enough, I’ve got no, I have no medical experience or and nothing, but I just know me you know, so. | | | | | | | | | | | | | |  |
|  | | |  | | | | | | | | | | | | | |  |
|  | | | | | | | | | | | | | | | | | |
|  | | | | | | | | | | | | | | | | | |
| Formatted Reports\\Coding Summary by File Formatted Report | | | | | | | | | | | Page 12 of 39 | | | | | | |
| 6/10/2022 8:17 AM | | | | | | | | | | | | | | | | | |
|  | | | **Classification** |  | **Aggregate** |  | **Coverage** |  | **Number Of Coding References** |  | | **Reference Number** |  | **Coded By Initials** |  | **Modified On** |  |
|  | | | **Codes\\quality of life changes\quality of life changes - lutetium** | | | | | | | | | | | | | |  |
|  |  |  |  |  | No |  | 0.0354 |  | 1 |  | | | | | | |  |
|  | | |  |  |  |  |  |  |  |  | | | | | | | |
|  | | | | | | | | | | | | 1 |  | BV |  | 25/03/2020 9:52 AM |  |
|  | | | well the whole thing is a change of life, is you can’t plan on nothing and it’s been a total I don’t know, whatever you call it. The early part was not too bad because we were caravanners'. We can’t go away like we used to you know, for a month a long time. Yeah and I’ve actually sold it; I’ve sold my van because I thought because (wife) actually caught – got to, I don’t know whether you catch it or not, but she got melanoma and that was back about 6 months ago and we thought or Judy did anyway, that she was going to die. And I dare say, and I actually had faith that I thought she would survive and but most people thought well that’s it for Jude. But she’s now on nanotherapy and it is sort of working really well. | | | | | | | | | | | | | |  |
|  | | |  | | | | | | | | | | | | | |  |
|  | | | **Codes\\side effects\side effects - lutetium** | | | | | | | | | | | | | |  |
|  |  |  |  |  | No |  | 0.1061 |  | 4 |  | | | | | | |  |
|  | | |  |  |  |  |  |  |  |  | | | | | | | |
|  | | | | | | | | | | | | 1 |  | BV |  | 25/03/2020 9:50 AM |  |
|  | | | I’m feeling alright, but I’ve started taking pain killers on the 17th on last Friday, I don’t know whether that was the 17th on last Friday, well it was definitely the 17th anyway, 17th of January. I had aches and pains you know, all over the place, I felt – I must admit I did do some hard sort of hard work prior to that and I don’t really know whether that was you know, had some influence on the pain or not, I don’t know. But I had the - it felt like the cancer in my … (Unable to understand) aches and shooting sometimes they’re just shooting pain and most times they’re just sort of like a dull ache type thing you know. I really can’t control them too much. But anyway now I’m on, I take 2 Panadol - well then when on from Friday, I used to take Panadol every 6 hours and I’m also on Lyrica. | | | | | | | | | | | | | |  |
|  | | |  | | | | | | | | | | | | | |  |
|  | | | | | | | | | | | | 2 |  | BV |  | 25/03/2020 9:50 AM |  |
|  | | | I take 1 of those in the morning and 1 at night and I now only take 1 Lyrica in the morning, 2 Panadol and 1 Lyrica at night and 2 Panadol and that’s it and I’m pretty well good. | | | | | | | | | | | | | |  |
|  | | |  | | | | | | | | | | | | | |  |
|  | | | | | | | | | | | | 3 |  | BV |  | 25/03/2020 9:54 AM |  |
|  | | | the side effects are just not worth it you know, I get – the last 2 times I had, I thought I was getting a sty in the eye right, that was on my right eye and it was in the corner you know, in the you know, the nose side you know? And it was very sore and it swelled up underneath and I’m thinking god you never see people - because I thought it was a sty at the time, I know it’s an old – people used to get it when we were kids. And I thought oh well, that’s it. But anyway I got, I just got – I had like urine type stuff and I just used to squirt that into my eye every now and again and that fixed it up. And it took a while; it took about 8 days or something and after I had the last thing, blow me, that’s why I now realise it’s the, the Lutetium was doing it, because it was in the right eye; it was on the outside corner, you know? | | | | | | | | | | | | | |  |
|  | | |  | | | | | | | | | | | | | |  |
|  | | | | | | | | | | | | 4 |  | BV |  | 25/03/2020 9:54 AM |  |
|  | | | And I also reckon even though they say it doesn’t wreck your feet, but I had whatever that you know, and my feet got numb and no feeling in my fingers. My fingers and everything have come good but my feet have never really got back to where they are and I reckon sometimes that this stuff makes them even you know, makes them bad as well so, I don’t know. | | | | | | | | | | | | | |  |
|  | | |  | | | | | | | | | | | | | |  |
|  | | | **Codes\\struggling with treatment** | | | | | | | | | | | | | |  |
|  |  |  |  |  | No |  | 0.0199 |  | 2 |  | | | | | | |  |
|  | | |  |  |  |  |  |  |  |  | | | | | | | |
|  | | | | | | | | | | | | 1 |  | BV |  | 25/03/2020 9:55 AM |  |
|  | | | Because the side effects are just not worth it you know | | | | | | | | | | | | | |  |
|  | | |  | | | | | | | | | | | | | |  |
|  | | | | | | | | | | | | 2 |  | BV |  | 25/03/2020 9:54 AM |  |
|  | | | And I also reckon even though they say it doesn’t wreck your feet, but I had whatever that you know, and my feet got numb and no feeling in my fingers. My fingers and everything have come good but my feet have never really got back to where they are and I reckon sometimes that this stuff makes them even you know, makes them bad as well so, I don’t know. | | | | | | | | | | | | | |  |
|  | | |  | | | | | | | | | | | | | |  |
| Formatted Reports\\Coding Summary by File Formatted Report | | | | | | | | | | | Page 13 of 39 | | | | | | |
| 6/10/2022 8:17 AM | | | | | | | | | | | | | | | | | |
|  | | | **Classification** |  | **Aggregate** |  | **Coverage** |  | **Number Of Coding References** |  | | **Reference Number** |  | **Coded By Initials** |  | **Modified On** |  |
|  | | | **Codes\\uncertainty of the future** | | | | | | | | | | | | | |  |
|  |  |  |  |  | No |  | 0.0541 |  | 4 |  | | | | | | |  |
|  | | |  |  |  |  |  |  |  |  | | | | | | | |
|  | | | | | | | | | | | | 1 |  | BV |  | 25/03/2020 9:53 AM |  |
|  | | | No, well I’m not really worried, I look I expected it to go up which it was, would have to be the last so I had 3 weeks ago and then 6 weeks so, 9 weeks, 9 yeah, … (Unable to understand) when I take it. So 9 or 12 weeks ago when my PSA only went down a little bit to 22, I knew then that even though they – I … (Unable to understand) anyway because they – no, they’re doctors and fair enough, I’ve got no, I have no medical experience or and nothing, but I just know me you know, so. | | | | | | | | | | | | | |  |
|  | | |  | | | | | | | | | | | | | |  |
|  | | | | | | | | | | | | 2 |  | BV |  | 25/03/2020 9:55 AM |  |
|  | | | Q: Alrighty. If you don’t get the last treatment, do you know roughly what will happen?  A: Probably die. | | | | | | | | | | | | | |  |
|  | | |  | | | | | | | | | | | | | |  |
|  | | | | | | | | | | | | 3 |  | BV |  | 25/03/2020 9:59 AM |  |
|  | | | Yeah, well I hope something – I have a cure you see, there’s no cure; no cure for prostate cancer, no matter what they do to you, that doesn’t cure it you know, so. Now I think with cancer it gets stronger and stronger you know, and that’s when the drugs can’t kill a lot you know, so. | | | | | | | | | | | | | |  |
|  | | |  | | | | | | | | | | | | | |  |
|  | | | | | | | | | | | | 4 |  | BV |  | 25/03/2020 9:59 AM |  |
|  | | | Yeah, I - well I obviously do because I’ve always tried my best and I’m always looked on the bright side and all that you know, I’ve never been – I never walked around the house saying, (wife), yeah I’m buggered you know, blah-blah. | | | | | | | | | | | | | |  |
|  | | |  | | | | | | | | | | | | | |  |
|  | | | **Codes\\uncertainty of the future\paying for lutetium outside of the trial** | | | | | | | | | | | | | |  |
|  |  |  |  |  | No |  | 0.0165 |  | 1 |  | | | | | | |  |
|  | | |  |  |  |  |  |  |  |  | | | | | | | |
|  | | | | | | | | | | | | 1 |  | BV |  | 25/03/2020 9:58 AM |  |
|  | | | Well, I was actually thinking of paying for it if we never got it and I you know, if I had paid for it and I would be very shitty now because I thought that this was the beginning and end. I’m not saying it was going to cure me, but I thought I was going to be had relief for cancer for after I’d even finished you know, a year or 2 years. | | | | | | | | | | | | | |  |
|  | | |  | | | | | | | | | | | | | |  |
|  | | | **Codes\\uncertainty of the future\treatments left on TheraP** | | | | | | | | | | | | | |  |
|  |  |  |  |  | No |  | 0.0234 |  | 3 |  | | | | | | |  |
|  | | |  |  |  |  |  |  |  |  | | | | | | | |
|  | | | | | | | | | | | | 1 |  | BV |  | 25/03/2020 9:48 AM |  |
|  | | | No, I’ve got one more to go. | | | | | | | | | | | | | |  |
|  | | |  | | | | | | | | | | | | | |  |
|  | | | | | | | | | | | | 2 |  | BV |  | 25/03/2020 9:49 AM |  |
|  | | | I do have one more treatment and whether I get it or not, I’m not 100% sure, they’re, we’re going to be talking about it I think I get, I think I’m due for in about 3 weeks’ time or 2½ weeks or whatever it may be. | | | | | | | | | | | | | |  |
|  | | |  | | | | | | | | | | | | | |  |
|  | | | | | | | | | | | | 3 |  | BV |  | 25/03/2020 9:54 AM |  |
|  | | | Q: How do you feel about the future when the trial’s finished?  A: Well, I actually – really I don’t really want it – I mean, I’ll take it if they give it to me.  Q: The last treatment?  A: I’ll do it, but I do - really don’t want it. | | | | | | | | | | | | | |  |
|  | | |  |  |  |  |  |  |  |  |  |  |  |  |  |  |  |
|  | | |  | | | | | | | | | | | | | |  |
| Formatted Reports\\Coding Summary by File Formatted Report | | | | | | | | | | | Page 14 of 39 | | | | | | |
| 6/10/2022 8:17 AM | | | | | | | | | | | | | | | | | |
|  | | | **Classification** |  | **Aggregate** |  | **Coverage** |  | **Number Of Coding References** |  | | **Reference Number** |  | **Coded By Initials** |  | **Modified On** |  |
|  | | | **Codes\\uncertainty of the future\wanting a break from treatment** | | | | | | | | | | | | | |  |
|  |  |  |  |  | No |  | 0.0165 |  | 1 |  | | | | | | |  |
|  | | |  |  |  |  |  |  |  |  | | | | | | | |
|  | | | | | | | | | | | | 1 |  | BV |  | 25/03/2020 9:58 AM |  |
|  | | | Well, I was actually thinking of paying for it if we never got it and I you know, if I had paid for it and I would be very shitty now because I thought that this was the beginning and end. I’m not saying it was going to cure me, but I thought I was going to be had relief for cancer for after I’d even finished you know, a year or 2 years. | | | | | | | | | | | | | |  |
|  | | |  | | | | | | | | | | | | | |  |
|  | | | **Codes\\x - sociodemographic data\current PSA** | | | | | | | | | | | | | |  |
|  |  |  |  |  | No |  | 0.0032 |  | 1 |  | | | | | | |  |
|  | | |  |  |  |  |  |  |  |  | | | | | | | |
|  | | | | | | | | | | | | 1 |  | BV |  | 25/03/2020 9:49 AM |  |
|  | | | Q: So you’re PSA’s 70 at the moment?  A: 70 at the moment, yeah. | | | | | | | | | | | | | |  |
|  | | |  | | | | | | | | | | | | | |  |
|  | | | **Codes\\x - sociodemographic data\DOB** | | | | | | | | | | | | | |  |
|  |  |  |  |  | No |  | 0.0009 |  | 1 |  | | | | | | |  |
|  | | |  |  |  |  |  |  |  |  | | | | | | | |
|  | | | | | | | | | | | | 1 |  | BV |  | 25/03/2020 9:56 AM |  |
|  | | | 8th of April, 1947. | | | | | | | | | | | | | |  |
|  | | |  | | | | | | | | | | | | | |  |
|  | | | **Codes\\x - sociodemographic data\first diagnosed** | | | | | | | | | | | | | |  |
|  |  |  |  |  | No |  | 0.0883 |  | 3 |  | | | | | | |  |
|  | | |  |  |  |  |  |  |  |  | | | | | | | |
|  | | | | | | | | | | | | 1 |  | BV |  | 25/03/2020 9:56 AM |  |
|  | | | 2006, probably I would say look October or - I think I was operated in November and it probably took a month so, most likely – my … because where I used to work, we used to do blood tests you know? | | | | | | | | | | | | | |  |
|  | | |  | | | | | | | | | | | | | |  |
|  | | | | | | | | | | | | 2 |  | BV |  | 25/03/2020 9:56 AM |  |
|  | | | And as far back as 1996 my PSA and that started to move and my – we used to do a blood test you know, because we worked with Benzyl all that type of that, a lot of different chemicals. And it was – it started to move up and then in October 96-98 and then 2000 and went 2002, 2004 and 2006. And my doctor; work doctor, I had – I only should of listened to him but I didn’t do it because I’m always a she’ll be right bloke. It was going up and even in like 2000 he said, “Look, it’s within the range, get it checked out”, I said “Oh yeah, fair enough.” And of course I went back there in 2002 and a guy up at … he said, “Look, get it checked, get it checked”, I said ”Yeah alright,” and never did. 2004 I think it was 3.9 and I still did nothing and then of course 2006 it was 9 point something, 9 – let’s just say 9.8. It was high and he said, “If you don’t,” he said to me, “If you don’t do any, something I will do it right”, and I said, “Alright, I’ll go and get it checked.” So, I went to my doctor and you know, … (Unable to understand) they all thought oh no, that’s all good, … (Unable to understand) they sent me off to a biopsy at Peter Mac you know, and I went in there for a biopsy and when I was coming out I thought well, I’ll never be back here again. And not that I realised it in November or something like that, at the end of December anyway, yeah the end of 2006 anyway it was probably November, could’ve been early - no I think it was November sometime and I went back and had my prostate removed so, yeah. | | | | | | | | | | | | | |  |
|  | | |  |  |  |  |  |  |  |  |  |  |  |  |  |  |  |
|  | | |  | | | | | | | | | | | | | |  |
|  | | | | | | | | | | | | 3 |  | BV |  | 25/03/2020 9:58 AM |  |
|  | | | Yes, I was. I was diagnosed and I tell you it was in a month I had my prostate gone. | | | | | | | | | | | | | |  |
|  | | |  | | | | | | | | | | | | | |  |
| Formatted Reports\\Coding Summary by File Formatted Report | | | | | | | | | | | Page 15 of 39 | | | | | | |
| 6/10/2022 8:17 AM | | | | | | | | | | | | | | | | | |
|  | | | **Classification** |  | **Aggregate** |  | **Coverage** |  | **Number Of Coding References** |  | | **Reference Number** |  | **Coded By Initials** |  | **Modified On** |  |
|  | | | **Codes\\x - sociodemographic data\gleason score** | | | | | | | | | | | | | |  |
|  |  |  |  |  | No |  | 0.0113 |  | 1 |  | | | | | | |  |
|  | | |  |  |  |  |  |  |  |  | | | | | | | |
|  | | | | | | | | | | | | 1 |  | BV |  | 25/03/2020 9:58 AM |  |
|  | | | Yes, well when I was first diagnosed I had 2 shocks that day because they said you’ve got cancer which I didn’t, I thought that was a shock and then the Gleason Score was before 3 and 10 or something like that and mine was bloody 9. | | | | | | | | | | | | | |  |
|  | | |  | | | | | | | | | | | | | |  |
|  | | | **Codes\\x - sociodemographic data\previous treatment** | | | | | | | | | | | | | |  |
|  |  |  |  |  | No |  | 0.1639 |  | 7 |  | | | | | | |  |
|  | | |  |  |  |  |  |  |  |  | | | | | | | |
|  | | | | | | | | | | | | 1 |  | BV |  | 25/03/2020 9:56 AM |  |
|  | | | Q: Did you have a radical prostatectomy?  A: I did, yep.  Q: Do you remember what type it was? So-  A: What’s that again?  Q: Do you remember what type of surgery it was? So open or surgical-  A: Yeah, open. | | | | | | | | | | | | | |  |
|  | | |  |  |  |  |  |  |  |  |  |  |  |  |  |  |  |
|  | | |  | | | | | | | | | | | | | |  |
|  | | | | | | | | | | | | 2 |  | BV |  | 25/03/2020 9:56 AM |  |
|  | | | And they – he said we’ll have to do the open because he wanted to have a good look because I think it was my, was it urethra tube or something you know- | | | | | | | | | | | | | |  |
|  | | |  | | | | | | | | | | | | | |  |
|  | | | | | | | | | | | | 3 |  | BV |  | 25/03/2020 9:56 AM |  |
|  | | | You know, of course that’s the prostate got cancer and they removed some of the lymph glands or whatever those things are. | | | | | | | | | | | | | |  |
|  | | |  | | | | | | | | | | | | | |  |
|  | | | | | | | | | | | | 4 |  | BV |  | 25/03/2020 9:57 AM |  |
|  | | | And they – then god only knows what else they removed because I don’t really take any notice; if they tell me you know, take a pill, I take a pill. I don’t bother you know saying well that’s a load of crap, I believe I should take this one because as I said you got to take notice of them you know so, yeah. | | | | | | | | | | | | | |  |
|  | | |  | | | | | | | | | | | | | |  |
|  | | | | | | | | | | | | | | | | | |
|  | | | | | | | | | | | | | | | | | |
|  | | | | | | | | | | | | | | | | | |
| Formatted Reports\\Coding Summary by File Formatted Report | | | | | | | | | | | Page 16 of 39 | | | | | | |
| 6/10/2022 8:17 AM | | | | | | | | | | | | | | | | | |
|  | | | **Classification** |  | **Aggregate** |  | **Coverage** |  | **Number Of Coding References** |  | | **Reference Number** |  | **Coded By Initials** |  | **Modified On** |  |
|  | | | | | | | | | | | | | | | | | |
|  | | | | | | | | | | | | 5 |  | BV |  | 25/03/2020 9:57 AM |  |
|  | | | Q: That’s alright. So you said you’ve had some chemotherapy as well?  A: I did, well that was later, I had chemo – well I’ve had this trial, I had a trial before that; that was the, they had a trial with the … you know ever, have you heard of that one?  Q: Yep.  A: Right, so we have the, and so I was tested and I had the, it was the ATM Gene I had right.  Q: Oh yeah.  A: And so yeah, so I was eligible to have a go at this stuff and really and for about the first 2 or 3 months it did nothing then it started bringing my PSA down only for a short period of time and then it started going back up. The side effects of that drug were ridiculous; I could not even go out in the sun, even for a second.  Q: Oh wow.  A: As soon as the sun shined on me my skin must’ve been that sensitive or something you know, and believe you me if I only knew how long that was going to last. As soon as I stopped, soon as – when it started going up, he stopped it and really I really had no relief at all because when it did go down I was so sensitive to the sun and I love the sun, I like going out, going outside, so and … (Talking over each other)-  Q: When – what year was that?  A: What year?  Q: Yeah.  A: Well that was before I started and I started this stuff probably half way through last year or maybe April or whatever so, that would’ve be - I would’ve been having that, that was, was that 2019 last year. So probably 2018 I might’ve started it, something like that you know, late 2019.  Q: Okay.  A: … 2018 I should say. And prior to that I had the chemo, don’t ask me what chemo it was-  Q: That’s alright.  A: I have no idea, but I had chemo.  Q: Did you have any radiation therapy?  A: Yes, when I had my prostrate removed and after I don’t know, 1, 2, 5 weeks, I don’t really know, I can’t remember now anyway.  Q: That’s okay. | | | | | | | | | | | | | |  |
|  | | |  |  |  |  |  |  |  |  |  |  |  |  |  |  |  |
|  | | |  |  |  |  |  |  |  |  |  |  |  |  |  |  |  |
|  | | |  |  |  |  |  |  |  |  |  |  |  |  |  |  |  |
|  | | |  |  |  |  |  |  |  |  |  |  |  |  |  |  |  |
|  | | |  |  |  |  |  |  |  |  |  |  |  |  |  |  |  |
|  | | |  |  |  |  |  |  |  |  |  |  |  |  |  |  |  |
|  | | |  | | | | | | | | | | | | | |  |
|  | | | | | | | | | | | | 6 |  | BV |  | 25/03/2020 9:57 AM |  |
|  | | | A: Yeah, so I had radiation not long, I couldn’t tell you whether it sort of been 5 months, 4 months, 6 months. Wasn’t very long after I had my prostate removed that I had in the groin area or whatever they want to call that area, I had the full radiation; I had 35 yeah, 35, 7 weeks it took – 7, 5’s yeah. So I did it for 7 weeks, 5 times a week, yeah.  Q: Yeah, was it the – do you remember if it was the external beams so you kind of go through-?  A: Yes, they – I just got on the machine and they-  Q: Yep, so it wasn’t internal?  A: Blasted you then shocked you with whatever they give you, you know. | | | | | | | | | | | | | |  |
|  | | |  |  |  |  |  |  |  |  |  |  |  |  |  |  |  |
|  | | |  | | | | | | | | | | | | | |  |
| Formatted Reports\\Coding Summary by File Formatted Report | | | | | | | | | | | Page 17 of 39 | | | | | | |
| 6/10/2022 8:17 AM | | | | | | | | | | | | | | | | | |
|  | | | **Classification** |  | **Aggregate** |  | **Coverage** |  | **Number Of Coding References** |  | | **Reference Number** |  | **Coded By Initials** |  | **Modified On** |  |
|  | | | | | | | | | | | | | | | | | |
|  | | | | | | | | | | | | 7 |  | BV |  | 25/03/2020 9:58 AM |  |
|  | | | Yes, I was. I was diagnosed and I tell you it was in a month I had my prostate gone. | | | | | | | | | | | | | |  |
|  | | |  | | | | | | | | | | | | | |  |
|  | **Files\\ID011.4** | | | | | | | | | | | | | | | |  |
|  | | **Code** | | | | | | | | | | | | | | |  |
|  | | | **Codes\\currently stable** | | | | | | | | | | | | | |  |
|  |  |  |  |  | No |  | 0.0053 |  | 2 |  | | | | | | |  |
|  | | |  |  |  |  |  |  |  |  | | | | | | | |
|  | | | | | | | | | | | | 1 |  | BV |  | 25/03/2020 10:30 AM |  |
|  | | | I’m going okay, yeah. Everything seems to be pretty stable. | | | | | | | | | | | | | |  |
|  | | |  | | | | | | | | | | | | | |  |
|  | | | | | | | | | | | | 2 |  | BV |  | 25/03/2020 10:31 AM |  |
|  | | | Yeah I’m stable, seem to be stable. | | | | | | | | | | | | | |  |
|  | | |  | | | | | | | | | | | | | |  |
|  | | | **Codes\\currently stable\functioning well** | | | | | | | | | | | | | |  |
|  |  |  |  |  | No |  | 0.0110 |  | 1 |  | | | | | | |  |
|  | | |  |  |  |  |  |  |  |  | | | | | | | |
|  | | | | | | | | | | | | 1 |  | BV |  | 25/03/2020 10:40 AM |  |
|  | | | Well – well yeah we don’t know. It’s just a funny one, that’s the thing, that’s what I’m saying. The numbers are high, but I’m actually functioning as good as – as I am when they were down at .2. | | | | | | | | | | | | | |  |
|  | | |  | | | | | | | | | | | | | |  |
|  | | | **Codes\\emotional impact** | | | | | | | | | | | | | |  |
|  |  |  |  |  | No |  | 0.0707 |  | 4 |  | | | | | | |  |
|  | | |  |  |  |  |  |  |  |  | | | | | | | |
|  | | | | | | | | | | | | 1 |  | BV |  | 25/03/2020 10:36 AM |  |
|  | | | Yeah I’m … about the whole thing. It’s definitely not as big, because you’re not worried about the treatment knocking you around. So you don’t tend to get a bit anxious when the time comes. And you’re like here we go, times are – time to get hit again. Whereas on – on this particular drug yeah it’s – you just go and do it because it’s – it’s very easy to – to deal with. See as far as anxiety and that, you don’t seem to have any when it comes to this drug. Whereas the other one you think, you just sort of get yourself ready for the – the hit that it’s going to give you. | | | | | | | | | | | | | |  |
|  | | |  | | | | | | | | | | | | | |  |
|  | | | | | | | | | | | | 2 |  | BV |  | 25/03/2020 10:37 AM |  |
|  | | | Yeah, yeah, yeah. And that – that in itself makes you feel better, because you just – you – you haven’t got all those problems with – that come with the chemo. | | | | | | | | | | | | | |  |
|  | | |  | | | | | | | | | | | | | |  |
|  | | | | | | | | | | | | 3 |  | BV |  | 25/03/2020 10:37 AM |  |
|  | | | So yeah as far as – you just function a lot better, you seem to be happier. And because yeah you – you have – don’t have those hard times. | | | | | | | | | | | | | |  |
|  | | |  | | | | | | | | | | | | | |  |
| Formatted Reports\\Coding Summary by File Formatted Report | | | | | | | | | | | Page 18 of 39 | | | | | | |
| 6/10/2022 8:17 AM | | | | | | | | | | | | | | | | | |
|  | | | **Classification** |  | **Aggregate** |  | **Coverage** |  | **Number Of Coding References** |  | | **Reference Number** |  | **Coded By Initials** |  | **Modified On** |  |
|  | | | | | | | | | | | | | | | | | |
|  | | | | | | | | | | | | 4 |  | BV |  | 25/03/2020 10:41 AM |  |
|  | | | Oh I think it’d be brilliant. I think I’ve been so lucky to have truly because the side effects aren’t there like on chemo. And I’d love other people to experience what I’ve experienced. So if anything it’s justified. Well my thinking that – if I could make – be apart of making a drug available to a lot of other people that wouldn’t have been without the trial. Then it’s worth it. | | | | | | | | | | | | | |  |
|  | | |  | | | | | | | | | | | | | |  |
|  | | | **Codes\\emotional impact\coping strategies** | | | | | | | | | | | | | |  |
|  |  |  |  |  | No |  | 0.0111 |  | 2 |  | | | | | | |  |
|  | | |  |  |  |  |  |  |  |  | | | | | | | |
|  | | | | | | | | | | | | 1 |  | BV |  | 25/03/2020 10:30 AM |  |
|  | | | I’m going okay, yeah. Everything seems to be pretty stable. | | | | | | | | | | | | | |  |
|  | | |  | | | | | | | | | | | | | |  |
|  | | | | | | | | | | | | 2 |  | BV |  | 25/03/2020 10:37 AM |  |
|  | | | So yeah as far as – you just function a lot better, you seem to be happier. And because yeah you – you have – don’t have those hard times. | | | | | | | | | | | | | |  |
|  | | |  | | | | | | | | | | | | | |  |
|  | | | **Codes\\financial impact** | | | | | | | | | | | | | |  |
|  |  |  |  |  | No |  | 0.0342 |  | 2 |  | | | | | | |  |
|  | | |  |  |  |  |  |  |  |  | | | | | | | |
|  | | | | | | | | | | | | 1 |  | BV |  | 25/03/2020 10:34 AM |  |
|  | | | No just – well I suppose just the – the travel after a while. You think it’s just – it’s a long way. And it costs money to travel up and down. That’s – that’s probably the hardest thing is the financial … that type of work. And I’m not working. And like you said – yeah so it gets a little bit – a little bit straining that way. But you just make it work, because you have to. | | | | | | | | | | | | | |  |
|  | | |  | | | | | | | | | | | | | |  |
|  | | | | | | | | | | | | 2 |  | BV |  | 25/03/2020 10:38 AM |  |
|  | | | You’ve – just – just the cost of getting there. And because we’ve stayed during the treatment cycle, you’ve got to pay for accommodation and stuff too. So it’s – it’s very … noticeable during - that’s the downside is the money. | | | | | | | | | | | | | |  |
|  | | |  | | | | | | | | | | | | | |  |
|  | | | **Codes\\happy about participating** | | | | | | | | | | | | | |  |
|  |  |  |  |  | No |  | 0.0418 |  | 5 |  | | | | | | |  |
|  | | |  |  |  |  |  |  |  |  | | | | | | | |
|  | | | | | | | | | | | | 1 |  | BV |  | 25/03/2020 10:38 AM |  |
|  | | | Q: So you feel like it’s worth it?  A: Oh absolutely, absolutely. | | | | | | | | | | | | | |  |
|  | | |  | | | | | | | | | | | | | |  |
|  | | | | | | | | | | | | 2 |  | BV |  | 25/03/2020 10:38 AM |  |
|  | | | Yeah, yeah and chemo is – by this stage you would be absolutely dying to get off it. Whereas this one, it didn’t matter. I wouldn’t worry me if I had to keep going. | | | | | | | | | | | | | |  |
|  | | |  | | | | | | | | | | | | | |  |
|  | | | | | | | | | | | | 3 |  | BV |  | 25/03/2020 10:39 AM |  |
|  | | | Yeah it’s just such tolerable treatment, it really is. | | | | | | | | | | | | | |  |
|  | | |  | | | | | | | | | | | | | |  |
|  | | | | | | | | | | | | 4 |  | BV |  | 25/03/2020 10:41 AM |  |
|  | | | Oh I think it’d be brilliant. I think I’ve been so lucky to have truly because the side effects aren’t there like on chemo. And I’d love other people to experience what I’ve experienced. So if anything it’s justified. Well my thinking that – if I could make – be apart of making a drug available to a lot of other people that wouldn’t have been without the trial. Then it’s worth it. | | | | | | | | | | | | | |  |
|  | | |  | | | | | | | | | | | | | |  |
| Formatted Reports\\Coding Summary by File Formatted Report | | | | | | | | | | | Page 19 of 39 | | | | | | |
| 6/10/2022 8:17 AM | | | | | | | | | | | | | | | | | |
|  | | | **Classification** |  | **Aggregate** |  | **Coverage** |  | **Number Of Coding References** |  | | **Reference Number** |  | **Coded By Initials** |  | **Modified On** |  |
|  | | | | | | | | | | | | | | | | | |
|  | | | | | | | | | | | | 5 |  | BV |  | 25/03/2020 10:42 AM |  |
|  | | | Yeah, yeah, yeah I think so. I definitely think it’s working quite well. | | | | | | | | | | | | | |  |
|  | | |  | | | | | | | | | | | | | |  |
|  | | | **Codes\\happy about participating\worth it** | | | | | | | | | | | | | |  |
|  |  |  |  |  | No |  | 0.0257 |  | 2 |  | | | | | | |  |
|  | | |  |  |  |  |  |  |  |  | | | | | | | |
|  | | | | | | | | | | | | 1 |  | BV |  | 25/03/2020 10:42 AM |  |
|  | | | Oh I think it’d be brilliant. I think I’ve been so lucky to have truly because the side effects aren’t there like on chemo. And I’d love other people to experience what I’ve experienced. So if anything it’s justified. Well my thinking that – if I could make – be apart of making a drug available to a lot of other people that wouldn’t have been without the trial. Then it’s worth it. | | | | | | | | | | | | | |  |
|  | | |  | | | | | | | | | | | | | |  |
|  | | | | | | | | | | | | 2 |  | BV |  | 25/03/2020 10:42 AM |  |
|  | | | Yeah, yeah, yeah I think so. I definitely think it’s working quite well. | | | | | | | | | | | | | |  |
|  | | |  | | | | | | | | | | | | | |  |
|  | | | **Codes\\hope** | | | | | | | | | | | | | |  |
|  |  |  |  |  | No |  | 0.0221 |  | 1 |  | | | | | | |  |
|  | | |  |  |  |  |  |  |  |  | | | | | | | |
|  | | | | | | | | | | | | 1 |  | BV |  | 25/03/2020 10:39 AM |  |
|  | | | I don’t know – well I mean I’m go be – I’m hoping that we get window of no treatment. Which means we’ll just have no – no commitments to doctors, and stuff like we’ve had, up until this point, over the last 4 months, or whatever it’s been – 5 months. Yeah I’m hoping that we’re a bit independent of medical people for a little while. We just hope we get a bit of a plateau of – of nothingness. | | | | | | | | | | | | | |  |
|  | | |  | | | | | | | | | | | | | |  |
|  | | | **Codes\\inital motivations for erolment** | | | | | | | | | | | | | |  |
|  |  |  |  |  | No |  | 0.0303 |  | 1 |  | | | | | | |  |
|  | | |  |  |  |  |  |  |  |  | | | | | | | |
|  | | | | | | | | | | | | 1 |  | BV |  | 25/03/2020 10:41 AM |  |
|  | | | I wanted to pay it forward like people had … for me. And that was – that was a big driving one. I mean what – the other one was to get a different treatment. But the main one was that I’d said right - all the way through that if the trials became available, I would do them. If – if the – if I was in the right cycle for it, because people have done it for the past 20 years. And given us better drugs than we – than what they had. And – and I heard that they’re – this Lutetium is one that they’re looking at taking as a mainstream drug- | | | | | | | | | | | | | |  |
|  | | |  | | | | | | | | | | | | | |  |
|  | | | **Codes\\no additional support required** | | | | | | | | | | | | | |  |
|  |  |  |  |  | No |  | 0.0076 |  | 1 |  | | | | | | |  |
|  | | |  |  |  |  |  |  |  |  | | | | | | | |
|  | | | | | | | | | | | | 1 |  | BV |  | 25/03/2020 10:39 AM |  |
|  | | | Q: Do you feel like you required any kind – any more support or care during the trial, in any shape or form?  A: No, no probably less. | | | | | | | | | | | | | |  |
|  | | |  | | | | | | | | | | | | | |  |
|  | | | | | | | | | | | | | | | | | |
| Formatted Reports\\Coding Summary by File Formatted Report | | | | | | | | | | | Page 20 of 39 | | | | | | |
| 6/10/2022 8:17 AM | | | | | | | | | | | | | | | | | |
|  | | | **Classification** |  | **Aggregate** |  | **Coverage** |  | **Number Of Coding References** |  | | **Reference Number** |  | **Coded By Initials** |  | **Modified On** |  |
|  | | | **Codes\\PSA post treatment\comparing own PSA to others** | | | | | | | | | | | | | |  |
|  |  |  |  |  | No |  | 0.0091 |  | 1 |  | | | | | | |  |
|  | | |  |  |  |  |  |  |  |  | | | | | | | |
|  | | | | | | | | | | | | 1 |  | BV |  | 25/03/2020 10:40 AM |  |
|  | | | Yeah it’s – it’s quite bizarre. And I felt other fellas - I’m probably the lowest of all the guys that I talk to on the trial. And there numbers are up in the 30s. | | | | | | | | | | | | | |  |
|  | | |  | | | | | | | | | | | | | |  |
|  | | | **Codes\\PSA post treatment\PSA numbers not staying down - lutetium** | | | | | | | | | | | | | |  |
|  |  |  |  |  | No |  | 0.0324 |  | 3 |  | | | | | | |  |
|  | | |  |  |  |  |  |  |  |  | | | | | | | |
|  | | | | | | | | | | | | 1 |  | BV |  | 25/03/2020 10:32 AM |  |
|  | | | Yeah I’m stable, seem to be stable. Number’s aren’t as good as what we’d – what – what I had, and what they’ve been on chemo. But they’re not too – they’re up a bit high. But I’m functioning yeah no worries at all. | | | | | | | | | | | | | |  |
|  | | |  | | | | | | | | | | | | | |  |
|  | | | | | | | | | | | | 2 |  | BV |  | 25/03/2020 10:39 AM |  |
|  | | | A: Last time it was 18.8.  Q: And that’s – has it been decreasing?  A: Yeah it’s come down from 30. So yeah when it got down to 14, then it’s come up again. | | | | | | | | | | | | | |  |
|  | | |  | | | | | | | | | | | | | |  |
|  | | | | | | | | | | | | 3 |  | BV |  | 25/03/2020 10:40 AM |  |
|  | | | Well – well yeah we don’t know. It’s just a funny one, that’s the thing, that’s what I’m saying. The numbers are high, but I’m actually functioning as good as – as I am when they were down at .2. | | | | | | | | | | | | | |  |
|  | | |  | | | | | | | | | | | | | |  |
|  | | | **Codes\\quality of life changes\quality of life changes - lutetium** | | | | | | | | | | | | | |  |
|  |  |  |  |  | No |  | 0.0626 |  | 6 |  | | | | | | |  |
|  | | |  |  |  |  |  |  |  |  | | | | | | | |
|  | | | | | | | | | | | | 1 |  | BV |  | 25/03/2020 10:35 AM |  |
|  | | | Well what during treatment, yeah it – it just – there’s not been many side effects what the – the trial drug is much, much better for quality of life than chemo. | | | | | | | | | | | | | |  |
|  | | |  | | | | | | | | | | | | | |  |
|  | | | | | | | | | | | | 2 |  | BV |  | 25/03/2020 10:35 AM |  |
|  | | | So as far as quality of life. This is so much easier to tolerate than chemo. | | | | | | | | | | | | | |  |
|  | | |  | | | | | | | | | | | | | |  |
|  | | | | | | | | | | | | 3 |  | BV |  | 25/03/2020 10:38 AM |  |
|  | | | Yeah, yeah and chemo is – by this stage you would be absolutely dying to get off it. Whereas this one, it didn’t matter. I wouldn’t worry me if I had to keep going. | | | | | | | | | | | | | |  |
|  | | |  | | | | | | | | | | | | | |  |
|  | | | | | | | | | | | | 4 |  | BV |  | 25/03/2020 10:39 AM |  |
|  | | | Yeah it’s just such tolerable treatment, it really is. | | | | | | | | | | | | | |  |
|  | | |  | | | | | | | | | | | | | |  |
|  | | | | | | | | | | | | 5 |  | BV |  | 25/03/2020 10:41 AM |  |
|  | | | Oh I think it’d be brilliant. I think I’ve been so lucky to have truly because the side effects aren’t there like on chemo. And I’d love other people to experience what I’ve experienced. So if anything it’s justified. Well my thinking that – if I could make – be apart of making a drug available to a lot of other people that wouldn’t have been without the trial. Then it’s worth it. | | | | | | | | | | | | | |  |
|  | | |  | | | | | | | | | | | | | |  |
| Formatted Reports\\Coding Summary by File Formatted Report | | | | | | | | | | | Page 21 of 39 | | | | | | |
| 6/10/2022 8:17 AM | | | | | | | | | | | | | | | | | |
|  | | | **Classification** |  | **Aggregate** |  | **Coverage** |  | **Number Of Coding References** |  | | **Reference Number** |  | **Coded By Initials** |  | **Modified On** |  |
|  | | | | | | | | | | | | | | | | | |
|  | | | | | | | | | | | | 6 |  | BV |  | 25/03/2020 10:44 AM |  |
|  | | | Just – just can’t get work around the house and that. You just can’t work too hard, because if it let’s you, you just – I’m still – still after 4 years learning that I just can’t do what I used to do. I can’t do anything near it. But my brain still tells me I can at times. | | | | | | | | | | | | | |  |
|  | | |  | | | | | | | | | | | | | |  |
|  | | | **Codes\\quality of trialists** | | | | | | | | | | | | | |  |
|  |  |  |  |  | No |  | 0.0310 |  | 2 |  | | | | | | |  |
|  | | |  |  |  |  |  |  |  |  | | | | | | | |
|  | | | | | | | | | | | | 1 |  | BV |  | 25/03/2020 10:33 AM |  |
|  | | | Oh look they’ve been incredibly helpful – everyone, because we’ve travelled that 2 and a half hours. And that’s on a good day. Yeah they’re – they’re very accommodating in that. They try and give you that – that sort of mid-morning appointment. So it gives you a chance to get through the traffic. And it also gives a chance to get home before the traffic – well get out of Melbourne before the traffic builds up too much. | | | | | | | | | | | | | |  |
|  | | |  | | | | | | | | | | | | | |  |
|  | | | | | | | | | | | | 2 |  | BV |  | 25/03/2020 10:33 AM |  |
|  | | | So no they’ve been really, really helpful in that regards. Everyone’s wonderful down there. We haven’t had any bad experiences. | | | | | | | | | | | | | |  |
|  | | |  | | | | | | | | | | | | | |  |
|  | | | **Codes\\side effects\comparing treatment to chemotherapy** | | | | | | | | | | | | | |  |
|  |  |  |  |  | No |  | 0.1331 |  | 11 |  | | | | | | |  |
|  | | |  |  |  |  |  |  |  |  | | | | | | | |
|  | | | | | | | | | | | | 1 |  | BV |  | 25/03/2020 10:31 AM |  |
|  | | | Yeah I’m stable, seem to be stable. Number’s aren’t as good as what we’d – what – what I had, and what they’ve been on chemo. But they’re not too – they’re up a bit high. But I’m functioning yeah no worries at all. | | | | | | | | | | | | | |  |
|  | | |  | | | | | | | | | | | | | |  |
|  | | | | | | | | | | | | 2 |  | BV |  | 25/03/2020 10:32 AM |  |
|  | | | And we’ve got a lot less side effects. | | | | | | | | | | | | | |  |
|  | | |  | | | | | | | | | | | | | |  |
|  | | | | | | | | | | | | 3 |  | BV |  | 25/03/2020 10:35 AM |  |
|  | | | Well what during treatment, yeah it – it just – there’s not been many side effects what the – the trial drug is much, much better for quality of life than chemo. | | | | | | | | | | | | | |  |
|  | | |  | | | | | | | | | | | | | |  |
|  | | | | | | | | | | | | 4 |  | BV |  | 25/03/2020 10:35 AM |  |
|  | | | You still get a bit tired. But nothing compared to chemo, no. And you haven’t got those cycles where you seem to follow on chemo, where you have a couple of good days after you have it. And then you have a week of really ordinary. Then you – and another week getting better. And you get a couple of days, good, then you get hit again. Whereas this one you’re good for 3 months, all of it. | | | | | | | | | | | | | |  |
|  | | |  | | | | | | | | | | | | | |  |
|  | | | | | | | | | | | | 5 |  | BV |  | 25/03/2020 10:35 AM |  |
|  | | | So as far as quality of life. This is so much easier to tolerate than chemo. | | | | | | | | | | | | | |  |
|  | | |  | | | | | | | | | | | | | |  |
|  | | | | | | | | | | | | 6 |  | BV |  | 25/03/2020 10:36 AM |  |
|  | | | Yeah I’m … about the whole thing. It’s definitely not as big, because you’re not worried about the treatment knocking you around. So you don’t tend to get a bit anxious when the time comes. And you’re like here we go, times are – time to get hit again. Whereas on – on this particular drug yeah it’s – you just go and do it because it’s – it’s very easy to – to deal with. See as far as anxiety and that, you don’t seem to have any when it comes to this drug. Whereas the other one you think, you just sort of get yourself ready for the – the hit that it’s going to give you. | | | | | | | | | | | | | |  |
|  | | |  | | | | | | | | | | | | | |  |
| Formatted Reports\\Coding Summary by File Formatted Report | | | | | | | | | | | Page 22 of 39 | | | | | | |
| 6/10/2022 8:17 AM | | | | | | | | | | | | | | | | | |
|  | | | **Classification** |  | **Aggregate** |  | **Coverage** |  | **Number Of Coding References** |  | | **Reference Number** |  | **Coded By Initials** |  | **Modified On** |  |
|  | | | | | | | | | | | | | | | | | |
|  | | | | | | | | | | | | 7 |  | BV |  | 25/03/2020 10:37 AM |  |
|  | | | Yeah, yeah, yeah. And that – that in itself makes you feel better, because you just – you – you haven’t got all those problems with – that come with the chemo. | | | | | | | | | | | | | |  |
|  | | |  | | | | | | | | | | | | | |  |
|  | | | | | | | | | | | | 8 |  | BV |  | 25/03/2020 10:37 AM |  |
|  | | | So yeah as far as – you just function a lot better, you seem to be happier. And because yeah you – you have – don’t have those hard times. | | | | | | | | | | | | | |  |
|  | | |  | | | | | | | | | | | | | |  |
|  | | | | | | | | | | | | 9 |  | BV |  | 25/03/2020 10:38 AM |  |
|  | | | Yeah, yeah and chemo is – by this stage you would be absolutely dying to get off it. Whereas this one, it didn’t matter. I wouldn’t worry me if I had to keep going. | | | | | | | | | | | | | |  |
|  | | |  | | | | | | | | | | | | | |  |
|  | | | | | | | | | | | | 10 |  | BV |  | 25/03/2020 10:39 AM |  |
|  | | | Yeah it’s just such tolerable treatment, it really is. | | | | | | | | | | | | | |  |
|  | | |  | | | | | | | | | | | | | |  |
|  | | | | | | | | | | | | 11 |  | BV |  | 25/03/2020 10:41 AM |  |
|  | | | Oh I think it’d be brilliant. I think I’ve been so lucky to have truly because the side effects aren’t there like on chemo. And I’d love other people to experience what I’ve experienced. So if anything it’s justified. Well my thinking that – if I could make – be apart of making a drug available to a lot of other people that wouldn’t have been without the trial. Then it’s worth it. | | | | | | | | | | | | | |  |
|  | | |  | | | | | | | | | | | | | |  |
|  | | | **Codes\\side effects\side effects - lutetium** | | | | | | | | | | | | | |  |
|  |  |  |  |  | No |  | 0.0522 |  | 6 |  | | | | | | |  |
|  | | |  |  |  |  |  |  |  |  | | | | | | | |
|  | | | | | | | | | | | | 1 |  | BV |  | 25/03/2020 10:32 AM |  |
|  | | | And we’ve got a lot less side effects. | | | | | | | | | | | | | |  |
|  | | |  | | | | | | | | | | | | | |  |
|  | | | | | | | | | | | | 2 |  | BV |  | 25/03/2020 10:32 AM |  |
|  | | | Q: Good, what side effects are you experiencing at the moment?  A: No not a lot, not a lot. That’s the beauty of it. | | | | | | | | | | | | | |  |
|  | | |  | | | | | | | | | | | | | |  |
|  | | | | | | | | | | | | 3 |  | BV |  | 25/03/2020 10:32 AM |  |
|  | | | Q: That’s great. Not even a dry mouth or anything like that?  A: No even that hasn’t kicked in. Which has been amazing. | | | | | | | | | | | | | |  |
|  | | |  | | | | | | | | | | | | | |  |
|  | | | | | | | | | | | | 4 |  | BV |  | 25/03/2020 10:35 AM |  |
|  | | | You still get a bit tired. But nothing compared to chemo, no. And you haven’t got those cycles where you seem to follow on chemo, where you have a couple of good days after you have it. And then you have a week of really ordinary. Then you – and another week getting better. And you get a couple of days, good, then you get hit again. Whereas this one you’re good for 3 months, all of it. | | | | | | | | | | | | | |  |
|  | | |  | | | | | | | | | | | | | |  |
|  | | | | | | | | | | | | 5 |  | BV |  | 25/03/2020 10:39 AM |  |
|  | | | Yeah it’s just such tolerable treatment, it really is. | | | | | | | | | | | | | |  |
|  | | |  | | | | | | | | | | | | | |  |
| Formatted Reports\\Coding Summary by File Formatted Report | | | | | | | | | | | Page 23 of 39 | | | | | | |
| 6/10/2022 8:17 AM | | | | | | | | | | | | | | | | | |
|  | | | **Classification** |  | **Aggregate** |  | **Coverage** |  | **Number Of Coding References** |  | | **Reference Number** |  | **Coded By Initials** |  | **Modified On** |  |
|  | | | | | | | | | | | | | | | | | |
|  | | | | | | | | | | | | 6 |  | BV |  | 25/03/2020 10:40 AM |  |
|  | | | Well – well yeah we don’t know. It’s just a funny one, that’s the thing, that’s what I’m saying. The numbers are high, but I’m actually functioning as good as – as I am when they were down at .2. | | | | | | | | | | | | | |  |
|  | | |  | | | | | | | | | | | | | |  |
|  | | | **Codes\\travelling to treatment** | | | | | | | | | | | | | |  |
|  |  |  |  |  | No |  | 0.0883 |  | 5 |  | | | | | | |  |
|  | | |  |  |  |  |  |  |  |  | | | | | | | |
|  | | | | | | | | | | | | 1 |  | BV |  | 25/03/2020 10:33 AM |  |
|  | | | Oh look they’ve been incredibly helpful – everyone, because we’ve travelled that 2 and a half hours. And that’s on a good day. Yeah they’re – they’re very accommodating in that. They try and give you that – that sort of mid-morning appointment. So it gives you a chance to get through the traffic. And it also gives a chance to get home before the traffic – well get out of Melbourne before the traffic builds up too much. | | | | | | | | | | | | | |  |
|  | | |  | | | | | | | | | | | | | |  |
|  | | | | | | | | | | | | 2 |  | BV |  | 25/03/2020 10:34 AM |  |
|  | | | No just – well I suppose just the – the travel after a while. You think it’s just – it’s a long way. And it costs money to travel up and down. That’s – that’s probably the hardest thing is the financial … that type of work. And I’m not working. And like you said – yeah so it gets a little bit – a little bit straining that way. But you just make it work, because you have to. | | | | | | | | | | | | | |  |
|  | | |  | | | | | | | | | | | | | |  |
|  | | | | | | | | | | | | 3 |  | BV |  | 25/03/2020 10:37 AM |  |
|  | | | Yeah the travelling stuff yeah it’s – because it’s just – you’re such a distance for anything. And then you travel to Melbourne in an hour and a half, or hour and three quarters. And then takes you another hour or so just to get the next 15 k’s to get to the hospital. And it’s just – it’s once you get close to Melbourne it’s just a bugger of a place. | | | | | | | | | | | | | |  |
|  | | |  | | | | | | | | | | | | | |  |
|  | | | | | | | | | | | | 4 |  | BV |  | 25/03/2020 10:37 AM |  |
|  | | | Well, and they’re doing all these road works, and everything. And now we’ve got strikes today on – on the rail line. So it’s just – yeah it’s just – at the moment it’s just really tough. | | | | | | | | | | | | | |  |
|  | | |  | | | | | | | | | | | | | |  |
|  | | | | | | | | | | | | 5 |  | BV |  | 25/03/2020 10:38 AM |  |
|  | | | You’ve – just – just the cost of getting there. And because we’ve stayed during the treatment cycle, you’ve got to pay for accommodation and stuff too. So it’s – it’s very … noticeable during - that’s the downside is the money. | | | | | | | | | | | | | |  |
|  | | |  | | | | | | | | | | | | | |  |
|  | | | **Codes\\uncertainty of the future\treatments left on TheraP** | | | | | | | | | | | | | |  |
|  |  |  |  |  | No |  | 0.0057 |  | 1 |  | | | | | | |  |
|  | | |  |  |  |  |  |  |  |  | | | | | | | |
|  | | | | | | | | | | | | 1 |  | BV |  | 25/03/2020 10:31 AM |  |
|  | | | Q: Okay, I thought you might have finished by now.  A: No, no got the last treatment in 3 weeks time. | | | | | | | | | | | | | |  |
|  | | |  | | | | | | | | | | | | | |  |
|  | | | **Codes\\uncertainty of the future\wanting a break from treatment** | | | | | | | | | | | | | |  |
|  |  |  |  |  | No |  | 0.0326 |  | 2 |  | | | | | | |  |
|  | | |  |  |  |  |  |  |  |  | | | | | | | |
|  | | | | | | | | | | | | 1 |  | BV |  | 25/03/2020 10:39 AM |  |
|  | | | I don’t know – well I mean I’m go be – I’m hoping that we get window of no treatment. Which means we’ll just have no – no commitments to doctors, and stuff like we’ve had, up until this point, over the last 4 months, or whatever it’s been – 5 months. Yeah I’m hoping that we’re a bit independent of medical people for a little while. We just hope we get a bit of a plateau of – of nothingness. | | | | | | | | | | | | | |  |
|  | | |  | | | | | | | | | | | | | |  |
| Formatted Reports\\Coding Summary by File Formatted Report | | | | | | | | | | | Page 24 of 39 | | | | | | |
| 6/10/2022 8:17 AM | | | | | | | | | | | | | | | | | |
|  | | | **Classification** |  | **Aggregate** |  | **Coverage** |  | **Number Of Coding References** |  | | **Reference Number** |  | **Coded By Initials** |  | **Modified On** |  |
|  | | | | | | | | | | | | | | | | | |
|  | | | | | | | | | | | | 2 |  | BV |  | 25/03/2020 10:39 AM |  |
|  | | | Q: And take a bit of a break from treatment?  A: Yeah that would be absolutely – and yeah we’re hoping we can maybe slip in a little holiday somewhere, or something. It would be lovely. | | | | | | | | | | | | | |  |
|  | | |  | | | | | | | | | | | | | |  |
|  | | | **Codes\\x - sociodemographic data\children** | | | | | | | | | | | | | |  |
|  |  |  |  |  | No |  | 0.0008 |  | 1 |  | | | | | | |  |
|  | | |  |  |  |  |  |  |  |  | | | | | | | |
|  | | | | | | | | | | | | 1 |  | BV |  | 25/03/2020 10:42 AM |  |
|  | | | Yeah we’ve got 3 | | | | | | | | | | | | | |  |
|  | | |  | | | | | | | | | | | | | |  |
|  | | | **Codes\\x - sociodemographic data\country born** | | | | | | | | | | | | | |  |
|  |  |  |  |  | No |  | 0.0022 |  | 1 |  | | | | | | |  |
|  | | |  |  |  |  |  |  |  |  | | | | | | | |
|  | | | | | | | | | | | | 1 |  | BV |  | 25/03/2020 10:42 AM |  |
|  | | | Were you born in Australia?  A: Yes. | | | | | | | | | | | | | |  |
|  | | |  | | | | | | | | | | | | | |  |
|  | | | **Codes\\x - sociodemographic data\current PSA** | | | | | | | | | | | | | |  |
|  |  |  |  |  | No |  | 0.0043 |  | 1 |  | | | | | | |  |
|  | | |  |  |  |  |  |  |  |  | | | | | | | |
|  | | | | | | | | | | | | 1 |  | BV |  | 25/03/2020 10:39 AM |  |
|  | | | Q: Yeah, do you know what your PSA is currently?  A: Last time it was 18.8. | | | | | | | | | | | | | |  |
|  | | |  | | | | | | | | | | | | | |  |
|  | | | **Codes\\x - sociodemographic data\DOB** | | | | | | | | | | | | | |  |
|  |  |  |  |  | No |  | 0.0012 |  | 1 |  | | | | | | |  |
|  | | |  |  |  |  |  |  |  |  | | | | | | | |
|  | | | | | | | | | | | | 1 |  | BV |  | 25/03/2020 10:42 AM |  |
|  | | | 28th of the second, 67. | | | | | | | | | | | | | |  |
|  | | |  | | | | | | | | | | | | | |  |
|  | | | **Codes\\x - sociodemographic data\education** | | | | | | | | | | | | | |  |
|  |  |  |  |  | No |  | 0.0004 |  | 1 |  | | | | | | |  |
|  | | |  |  |  |  |  |  |  |  | | | | | | | |
|  | | | | | | | | | | | | 1 |  | BV |  | 25/03/2020 10:42 AM |  |
|  | | | Year 12. | | | | | | | | | | | | | |  |
|  | | |  | | | | | | | | | | | | | |  |
| Formatted Reports\\Coding Summary by File Formatted Report | | | | | | | | | | | Page 25 of 39 | | | | | | |
| 6/10/2022 8:17 AM | | | | | | | | | | | | | | | | | |
|  | | | **Classification** |  | **Aggregate** |  | **Coverage** |  | **Number Of Coding References** |  | | **Reference Number** |  | **Coded By Initials** |  | **Modified On** |  |
|  | | | **Codes\\x - sociodemographic data\first diagnosed** | | | | | | | | | | | | | |  |
|  |  |  |  |  | No |  | 0.0052 |  | 1 |  | | | | | | |  |
|  | | |  |  |  |  |  |  |  |  | | | | | | | |
|  | | | | | | | | | | | | 1 |  | BV |  | 25/03/2020 10:43 AM |  |
|  | | | Q: 2016, were you diagnosed straight away with advanced cancer, or-?  A: Yes, yep, yep, yep. | | | | | | | | | | | | | |  |
|  | | |  | | | | | | | | | | | | | |  |
|  | | | **Codes\\x - sociodemographic data\income** | | | | | | | | | | | | | |  |
|  |  |  |  |  | No |  | 0.0004 |  | 1 |  | | | | | | |  |
|  | | |  |  |  |  |  |  |  |  | | | | | | | |
|  | | | | | | | | | | | | 1 |  | BV |  | 25/03/2020 10:43 AM |  |
|  | | | $50,000. | | | | | | | | | | | | | |  |
|  | | |  | | | | | | | | | | | | | |  |
|  | | | **Codes\\x - sociodemographic data\language** | | | | | | | | | | | | | |  |
|  |  |  |  |  | No |  | 0.0013 |  | 1 |  | | | | | | |  |
|  | | |  |  |  |  |  |  |  |  | | | | | | | |
|  | | | | | | | | | | | | 1 |  | BV |  | 25/03/2020 10:43 AM |  |
|  | | | A little bit of Italian. | | | | | | | | | | | | | |  |
|  | | |  | | | | | | | | | | | | | |  |
|  | | | **Codes\\x - sociodemographic data\marital status** | | | | | | | | | | | | | |  |
|  |  |  |  |  | No |  | 0.0003 |  | 1 |  | | | | | | |  |
|  | | |  |  |  |  |  |  |  |  | | | | | | | |
|  | | | | | | | | | | | | 1 |  | BV |  | 25/03/2020 10:42 AM |  |
|  | | | married | | | | | | | | | | | | | |  |
|  | | |  | | | | | | | | | | | | | |  |
|  | | | | | | | | | | | | | | | | | |
|  | | | | | | | | | | | | | | | | | |
|  | | | | | | | | | | | | | | | | | |
| Formatted Reports\\Coding Summary by File Formatted Report | | | | | | | | | | | Page 26 of 39 | | | | | | |
| 6/10/2022 8:17 AM | | | | | | | | | | | | | | | | | |
|  | | | **Classification** |  | **Aggregate** |  | **Coverage** |  | **Number Of Coding References** |  | | **Reference Number** |  | **Coded By Initials** |  | **Modified On** |  |
|  | | | **Codes\\x - sociodemographic data\previous treatment** | | | | | | | | | | | | | |  |
|  |  |  |  |  | No |  | 0.1178 |  | 1 |  | | | | | | |  |
|  | | |  |  |  |  |  |  |  |  | | | | | | | |
|  | | | | | | | | | | | | 1 |  | BV |  | 25/03/2020 10:44 AM |  |
|  | | | Q: No wait – no apologies, my apologies for it. No you had chemotherapy.  A: Correct.  Q: Do you remember what type of chemo that was?  A: Oh crikey, now you’re going to test me. No I … (Unable to understand) them at moment, I strengthen it. No I can’t remember.  Q: That’s okay. Do you remember what year it was that you had it in?  A: 2016.  Q: 2016 great. Have you had surgery at all?  A: No, not yet.  Q: No that’s okay. And any …-  A: Too advanced.  Q: Oh sorry, yeah too advanced-  A: Yeah it was too – it was too advanced to have any surgery.  Q: And any radiation?  A: Yeah I’ve had a couple of rounds, of the … I think a week, or a couple of weeks, one time, and a week the next time.  Q: Do you by any chance remember if it was – so there’s 2 types. We’ve got an – the external beam. So they kind of point straight you. Or was it internal?  A: No it’s external.  Q: External, great so that-  A: Yeah the machine just moves around you, and zaps you.  Q: Yep, do you roughly remember when that was? I’m testing you today.  A: I reckon – jeepers, I can’t remember if it was 2016, after, because he’s spine was – the cancer were eating my spine a bit. I can’t remember if it was 16, or 17.  Q: That’s okay.  A: Yeah and that was the first one. And then I had another one on a couple of spots that were really troublesome. But probably 12 months later. That’s why I can’t remember if it was 17, 18 or 16, and 17. It might have been late 16, and – or early 17 and – and middle of 18, or something.  Q: Alrighty we’ll put 17 and 18, that’s okay. Have you had any-  A: Yeah, yeah, yeah I think-  Q: Sorry, sorry you go.  A: No, no it’s alright.  Q: Have you had any hormone therapy?  A: Oh well I’m on the Zoladex implant. I’ve been on that pretty much right from the very start. Well after the first couple of injections they gave me to lower my testosterone. And then I’ve been on Zoladex pretty much from probably middle of 2016. And I’m currently | | | | | | | | | | | | | |  |
|  | | |  |  |  |  |  |  |  |  |  |  |  |  |  |  |  |
|  | | |  |  |  |  |  |  |  |  |  |  |  |  |  |  |  |
|  | | |  |  |  |  |  |  |  |  |  |  |  |  |  |  |  |
|  | | |  |  |  |  |  |  |  |  |  |  |  |  |  |  |  |
|  | | |  |  |  |  |  |  |  |  |  |  |  |  |  |  |  |
|  | | |  |  |  |  |  |  |  |  |  |  |  |  |  |  |  |
|  | | |  |  |  |  |  |  |  |  |  |  |  |  |  |  |  |
|  | | |  |  |  |  |  |  |  |  |  |  |  |  |  |  |  |
| Formatted Reports\\Coding Summary by File Formatted Report | | | | | | | | | | | Page 27 of 39 | | | | | | |
| 6/10/2022 8:17 AM | | | | | | | | | | | | | | | | | |
|  | | | **Classification** |  | **Aggregate** |  | **Coverage** |  | **Number Of Coding References** |  | | **Reference Number** |  | **Coded By Initials** |  | **Modified On** |  |
|  | | | still on it.  Q: Any other hormone treatments?  A: No. | | | | | | | | | | | | | |  |
|  | | |  | | | | | | | | | | | | | |  |
|  | | | **Codes\\x - sociodemographic data\residency** | | | | | | | | | | | | | |  |
|  |  |  |  |  | No |  | 0.0013 |  | 2 |  | | | | | | |  |
|  | | |  |  |  |  |  |  |  |  | | | | | | | |
|  | | | | | | | | | | | | 1 |  | BV |  | 25/03/2020 10:35 AM |  |
|  | | | Colac. | | | | | | | | | | | | | |  |
|  | | |  | | | | | | | | | | | | | |  |
|  | | | | | | | | | | | | 2 |  | BV |  | 25/03/2020 10:42 AM |  |
|  | | | Colac in Victoria | | | | | | | | | | | | | |  |
|  | | |  | | | | | | | | | | | | | |  |
|  | | | **Codes\\x - sociodemographic data\work status** | | | | | | | | | | | | | |  |
|  |  |  |  |  | No |  | 0.0102 |  | 1 |  | | | | | | |  |
|  | | |  |  |  |  |  |  |  |  | | | | | | | |
|  | | | | | | | | | | | | 1 |  | BV |  | 25/03/2020 10:43 AM |  |
|  | | | Unemployed. Yeah well I haven’t worked for – since I’ve been diagnosed pretty much. I tried to work – I worked for probably 18 months after I was diagnosed. But it just got too hard. | | | | | | | | | | | | | |  |
|  | | |  | | | | | | | | | | | | | |  |
|  | | | **Codes\\x - sociodemographic data\years married** | | | | | | | | | | | | | |  |
|  |  |  |  |  | No |  | 0.0011 |  | 1 |  | | | | | | |  |
|  | | |  |  |  |  |  |  |  |  | | | | | | | |
|  | | | | | | | | | | | | 1 |  | BV |  | 25/03/2020 10:42 AM |  |
|  | | | Coming up 30 years. | | | | | | | | | | | | | |  |
|  | | |  | | | | | | | | | | | | | |  |
|  | **Files\\ID012.4 + ID012P.4** | | | | | | | | | | | | | | | |  |
|  | | **Code** | | | | | | | | | | | | | | |  |
|  | | | **Codes\\currently stable** | | | | | | | | | | | | | |  |
|  |  |  |  |  | No |  | 0.0195 |  | 1 |  | | | | | | |  |
|  | | |  |  |  |  |  |  |  |  | | | | | | | |
|  | | | | | | | | | | | | 1 |  | BV |  | 25/03/2020 11:00 AM |  |
|  | | | I'd like to see … (Inaudible) he’s going to have another consultation in about 6 months’ time, and hopefully if things have all settled down and the cancer’s gone, I don’t think I’ll need to go back, you know maybe every couple of years or whatever. | | | | | | | | | | | | | |  |
|  | | |  | | | | | | | | | | | | | |  |
| Formatted Reports\\Coding Summary by File Formatted Report | | | | | | | | | | | Page 28 of 39 | | | | | | |
| 6/10/2022 8:17 AM | | | | | | | | | | | | | | | | | |
|  | | | **Classification** |  | **Aggregate** |  | **Coverage** |  | **Number Of Coding References** |  | | **Reference Number** |  | **Coded By Initials** |  | **Modified On** |  |
|  | | | **Codes\\facing mortality** | | | | | | | | | | | | | |  |
|  |  |  |  |  | No |  | 0.0106 |  | 1 |  | | | | | | |  |
|  | | |  |  |  |  |  |  |  |  | | | | | | | |
|  | | | | | | | | | | | | 1 |  | BV |  | 25/03/2020 11:01 AM |  |
|  | | | Q: Do you feel like you're happy that you enrolled in the trial?  M: Yes, oh look I could have been 6 feet under by now if I hadn't. | | | | | | | | | | | | | |  |
|  | | |  | | | | | | | | | | | | | |  |
|  | | | **Codes\\happy about participating** | | | | | | | | | | | | | |  |
|  |  |  |  |  | No |  | 0.0106 |  | 1 |  | | | | | | |  |
|  | | |  |  |  |  |  |  |  |  | | | | | | | |
|  | | | | | | | | | | | | 1 |  | BV |  | 25/03/2020 11:01 AM |  |
|  | | | Q: Do you feel like you're happy that you enrolled in the trial?  M: Yes, oh look I could have been 6 feet under by now if I hadn't. | | | | | | | | | | | | | |  |
|  | | |  | | | | | | | | | | | | | |  |
|  | | | **Codes\\happy about participating\worth it** | | | | | | | | | | | | | |  |
|  |  |  |  |  | No |  | 0.0030 |  | 1 |  | | | | | | |  |
|  | | |  |  |  |  |  |  |  |  | | | | | | | |
|  | | | | | | | | | | | | 1 |  | BV |  | 25/03/2020 11:01 AM |  |
|  | | | Been supported, and it's been worth it. | | | | | | | | | | | | | |  |
|  | | |  | | | | | | | | | | | | | |  |
|  | | | **Codes\\no additional support required** | | | | | | | | | | | | | |  |
|  |  |  |  |  | No |  | 0.0180 |  | 2 |  | | | | | | |  |
|  | | |  |  |  |  |  |  |  |  | | | | | | | |
|  | | | | | | | | | | | | 1 |  | BV |  | 25/03/2020 11:00 AM |  |
|  | | | Q: Alright. Have you felt like you needed any support closer to the end of this trial?  M: Not really, not really. | | | | | | | | | | | | | |  |
|  | | |  | | | | | | | | | | | | | |  |
|  | | | | | | | | | | | | 2 |  | BV |  | 25/03/2020 11:01 AM |  |
|  | | | Q: So do you feel like you would need any support or care once the trial is complete?  M: I don’t think so, no. | | | | | | | | | | | | | |  |
|  | | |  | | | | | | | | | | | | | |  |
|  | | | **Codes\\PSA post treatment\PSA gone down - lutetium** | | | | | | | | | | | | | |  |
|  |  |  |  |  | No |  | 0.0067 |  | 1 |  | | | | | | |  |
|  | | |  |  |  |  |  |  |  |  | | | | | | | |
|  | | | | | | | | | | | | 1 |  | BV |  | 25/03/2020 11:00 AM |  |
|  | | | Q: 3.3, well that’s not bad.  M: No it's come down a lot; it's come down a lot yeah. | | | | | | | | | | | | | |  |
|  | | |  | | | | | | | | | | | | | |  |
|  | | | | | | | | | | | | | | | | | |
| Formatted Reports\\Coding Summary by File Formatted Report | | | | | | | | | | | Page 29 of 39 | | | | | | |
| 6/10/2022 8:17 AM | | | | | | | | | | | | | | | | | |
|  | | | **Classification** |  | **Aggregate** |  | **Coverage** |  | **Number Of Coding References** |  | | **Reference Number** |  | **Coded By Initials** |  | **Modified On** |  |
|  | | | **Codes\\quality of life changes\quality of life changes - lutetium** | | | | | | | | | | | | | |  |
|  |  |  |  |  | No |  | 0.0017 |  | 1 |  | | | | | | |  |
|  | | |  |  |  |  |  |  |  |  | | | | | | | |
|  | | | | | | | | | | | | 1 |  | BV |  | 25/03/2020 10:58 AM |  |
|  | | | Just being a bit tired | | | | | | | | | | | | | |  |
|  | | |  | | | | | | | | | | | | | |  |
|  | | | **Codes\\quality of trialists** | | | | | | | | | | | | | |  |
|  |  |  |  |  | No |  | 0.0202 |  | 1 |  | | | | | | |  |
|  | | |  |  |  |  |  |  |  |  | | | | | | | |
|  | | | | | | | | | | | | 1 |  | BV |  | 25/03/2020 11:01 AM |  |
|  | | | I'm not a … I don’t enjoy going …. I mean there's some lovely people up there at the hospital yeah, meet different people, have lots of yarns and tell a few jokes and whatever, but no it's not the sort of place that you'd want to go if you didn’t have to. | | | | | | | | | | | | | |  |
|  | | |  | | | | | | | | | | | | | |  |
|  | | | **Codes\\side effects\side effects - lutetium** | | | | | | | | | | | | | |  |
|  |  |  |  |  | No |  | 0.0219 |  | 2 |  | | | | | | |  |
|  | | |  |  |  |  |  |  |  |  | | | | | | | |
|  | | | | | | | | | | | | 1 |  | BV |  | 25/03/2020 10:57 AM |  |
|  | | | Yeah not too bad, I still get a very dry mouth. | | | | | | | | | | | | | |  |
|  | | |  | | | | | | | | | | | | | |  |
|  | | | | | | | | | | | | 2 |  | BV |  | 25/03/2020 10:57 AM |  |
|  | | | I don’t have much of an appetite these days, the tastebuds, that’s probably part of the … nothing tastes … (Inaudible), I can't tell much difference between one thing and another these days, I just put it down the gob and away we go | | | | | | | | | | | | | |  |
|  | | |  | | | | | | | | | | | | | |  |
|  | | | **Codes\\uncertainty of the future\wanting a break from treatment** | | | | | | | | | | | | | |  |
|  |  |  |  |  | No |  | 0.0195 |  | 1 |  | | | | | | |  |
|  | | |  |  |  |  |  |  |  |  | | | | | | | |
|  | | | | | | | | | | | | 1 |  | BV |  | 25/03/2020 11:00 AM |  |
|  | | | I'd like to see … (Inaudible) he’s going to have another consultation in about 6 months’ time, and hopefully if things have all settled down and the cancer’s gone, I don’t think I’ll need to go back, you know maybe every couple of years or whatever. | | | | | | | | | | | | | |  |
|  | | |  | | | | | | | | | | | | | |  |
|  | | | **Codes\\x - sociodemographic data\country born** | | | | | | | | | | | | | |  |
|  |  |  |  |  | No |  | 0.0015 |  | 1 |  | | | | | | |  |
|  | | |  |  |  |  |  |  |  |  | | | | | | | |
|  | | | | | | | | | | | | 1 |  | BV |  | 25/03/2020 10:58 AM |  |
|  | | | Born in … Queensland | | | | | | | | | | | | | |  |
|  | | |  | | | | | | | | | | | | | |  |
|  | | | | | | | | | | | | | | | | | |
| Formatted Reports\\Coding Summary by File Formatted Report | | | | | | | | | | | Page 30 of 39 | | | | | | |
| 6/10/2022 8:17 AM | | | | | | | | | | | | | | | | | |
|  | | | **Classification** |  | **Aggregate** |  | **Coverage** |  | **Number Of Coding References** |  | | **Reference Number** |  | **Coded By Initials** |  | **Modified On** |  |
|  | | | **Codes\\x - sociodemographic data\current PSA** | | | | | | | | | | | | | |  |
|  |  |  |  |  | No |  | 0.0054 |  | 1 |  | | | | | | |  |
|  | | |  |  |  |  |  |  |  |  | | | | | | | |
|  | | | | | | | | | | | | 1 |  | BV |  | 25/03/2020 10:59 AM |  |
|  | | | Q: Yeah, do you know what your current psa level is?  M: About 3.3. | | | | | | | | | | | | | |  |
|  | | |  | | | | | | | | | | | | | |  |
|  | | | **Codes\\x - sociodemographic data\DOB** | | | | | | | | | | | | | |  |
|  |  |  |  |  | No |  | 0.0017 |  | 1 |  | | | | | | |  |
|  | | |  |  |  |  |  |  |  |  | | | | | | | |
|  | | | | | | | | | | | | 1 |  | BV |  | 25/03/2020 10:58 AM |  |
|  | | | 13th of February 1941. | | | | | | | | | | | | | |  |
|  | | |  | | | | | | | | | | | | | |  |
|  | | | **Codes\\x - sociodemographic data\education** | | | | | | | | | | | | | |  |
|  |  |  |  |  | No |  | 0.0025 |  | 1 |  | | | | | | |  |
|  | | |  |  |  |  |  |  |  |  | | | | | | | |
|  | | | | | | | | | | | | 1 |  | BV |  | 25/03/2020 10:58 AM |  |
|  | | | I went to matric at high school. | | | | | | | | | | | | | |  |
|  | | |  | | | | | | | | | | | | | |  |
|  | | | **Codes\\x - sociodemographic data\first diagnosed** | | | | | | | | | | | | | |  |
|  |  |  |  |  | No |  | 0.0077 |  | 2 |  | | | | | | |  |
|  | | |  |  |  |  |  |  |  |  | | | | | | | |
|  | | | | | | | | | | | | 1 |  | BV |  | 25/03/2020 10:59 AM |  |
|  | | | I don’t, but it was in 2012. | | | | | | | | | | | | | |  |
|  | | |  | | | | | | | | | | | | | |  |
|  | | | | | | | | | | | | 2 |  | BV |  | 25/03/2020 10:59 AM |  |
|  | | | Yeah I scored 9 out of 10 and I nearly died there and then on the spot | | | | | | | | | | | | | |  |
|  | | |  | | | | | | | | | | | | | |  |
|  | | | **Codes\\x - sociodemographic data\gleason score** | | | | | | | | | | | | | |  |
|  |  |  |  |  | No |  | 0.0036 |  | 1 |  | | | | | | |  |
|  | | |  |  |  |  |  |  |  |  | | | | | | | |
|  | | | | | | | | | | | | 1 |  | BV |  | 25/03/2020 10:59 AM |  |
|  | | | I scored 9 out of the 10 on the Gleason scale. | | | | | | | | | | | | | |  |
|  | | |  | | | | | | | | | | | | | |  |
|  | | | | | | | | | | | | | | | | | |
| Formatted Reports\\Coding Summary by File Formatted Report | | | | | | | | | | | Page 31 of 39 | | | | | | |
| 6/10/2022 8:17 AM | | | | | | | | | | | | | | | | | |
|  | | | **Classification** |  | **Aggregate** |  | **Coverage** |  | **Number Of Coding References** |  | | **Reference Number** |  | **Coded By Initials** |  | **Modified On** |  |
|  | | | **Codes\\x - sociodemographic data\previous treatment** | | | | | | | | | | | | | |  |
|  |  |  |  |  | No |  | 0.1359 |  | 1 |  | | | | | | |  |
|  | | |  |  |  |  |  |  |  |  | | | | | | | |
|  | | | | | | | | | | | | 1 |  | BV |  | 25/03/2020 11:00 AM |  |
|  | | | : Have you had any surgery in the past?  M: So I had my appendix out when I was about 19.  Q: So not a … or anything like that?  M: No they didn’t do any surgery, no. No as I said they didn’t do any because of those aneurisms.  Q: Yes. Have you had any radiation therapy?  M: Yeah I did have that, yeah that took them 37 days.  Q: Do you remember any chance, if that was an external, did something rotate around you, or was it internal?  M: No it was me lying on the bed and the bed goes in and out of the … tunnel ….  Q: Okay so the external beam, beautiful.  M: … anything like a reverse … and … (Inaudible) and each … that goes on has one of these, and when they … they pull it out …, put them on the table … (Inaudible) in the right spot for the radiation, as I said about 6 minutes was all it took per day.  Q: Alright, and your hormone therapy, you said you had zoladex …, did you by any chance receive zytiga?  M: Zytiga bloody nearly crippled me, yeah.  Q: Any other hormone therapy that you can remember?  M: No but they gave me, now what are those things they call, starts with …, now just let me think, steroids.  Q: Steroids, yeah.  M: Steroids, that’s what made me 110 kilograms.  Q: Do you remember the name of the steroids?  M: No I don’t, it’ll be on my …, feel free, my number, … got a pencil there?  Q: Yeah you’ve given it to me before, so it's ….  M: Yeah … have a look on the file and they’ll be able to tell you what it was. They’ve given me anti-nausea pills if I need them, and … something else … (Inaudible) but I haven't needed them at all.  Q: Alright. Have you felt like you needed any support closer to the end of this trial?  M: Not really, not really. | | | | | | | | | | | | | |  |
|  | | |  |  |  |  |  |  |  |  |  |  |  |  |  |  |  |
|  | | |  |  |  |  |  |  |  |  |  |  |  |  |  |  |  |
|  | | |  |  |  |  |  |  |  |  |  |  |  |  |  |  |  |
|  | | |  |  |  |  |  |  |  |  |  |  |  |  |  |  |  |
|  | | |  |  |  |  |  |  |  |  |  |  |  |  |  |  |  |
|  | | |  |  |  |  |  |  |  |  |  |  |  |  |  |  |  |
|  | | |  | | | | | | | | | | | | | |  |
|  | | | **Codes\\x - sociodemographic data\work status** | | | | | | | | | | | | | |  |
|  |  |  |  |  | No |  | 0.0058 |  | 1 |  | | | | | | |  |
|  | | |  |  |  |  |  |  |  |  | | | | | | | |
|  | | | | | | | | | | | | 1 |  | BV |  | 25/03/2020 10:58 AM |  |
|  | | | Oh I've been well and truly retired yes, the only work I do is housework. | | | | | | | | | | | | | |  |
|  | | |  | | | | | | | | | | | | | |  |
| Formatted Reports\\Coding Summary by File Formatted Report | | | | | | | | | | | Page 32 of 39 | | | | | | |
| 6/10/2022 8:17 AM | | | | | | | | | | | | | | | | | |
|  | | | **Classification** |  | **Aggregate** |  | **Coverage** |  | **Number Of Coding References** |  | | **Reference Number** |  | **Coded By Initials** |  | **Modified On** |  |
|  | | | **Codes\\y - partner sociodemorgraphic data\children** | | | | | | | | | | | | | |  |
|  |  |  |  |  | No |  | 0.0015 |  | 1 |  | | | | | | |  |
|  | | |  |  |  |  |  |  |  |  | | | | | | | |
|  | | | | | | | | | | | | 1 |  | BV |  | 25/03/2020 10:56 AM |  |
|  | | | No neither of us do. | | | | | | | | | | | | | |  |
|  | | |  | | | | | | | | | | | | | |  |
|  | | | **Codes\\y - partner sociodemorgraphic data\country born** | | | | | | | | | | | | | |  |
|  |  |  |  |  | No |  | 0.0013 |  | 1 |  | | | | | | |  |
|  | | |  |  |  |  |  |  |  |  | | | | | | | |
|  | | | | | | | | | | | | 1 |  | BV |  | 25/03/2020 10:56 AM |  |
|  | | | born in Brisbane, | | | | | | | | | | | | | |  |
|  | | |  | | | | | | | | | | | | | |  |
|  | | | **Codes\\y - partner sociodemorgraphic data\DOB** | | | | | | | | | | | | | |  |
|  |  |  |  |  | No |  | 0.0014 |  | 1 |  | | | | | | |  |
|  | | |  |  |  |  |  |  |  |  | | | | | | | |
|  | | | | | | | | | | | | 1 |  | BV |  | 25/03/2020 10:56 AM |  |
|  | | | 20th the third, 42 | | | | | | | | | | | | | |  |
|  | | |  | | | | | | | | | | | | | |  |
|  | | | **Codes\\y - partner sociodemorgraphic data\education** | | | | | | | | | | | | | |  |
|  |  |  |  |  | No |  | 0.0017 |  | 1 |  | | | | | | |  |
|  | | |  |  |  |  |  |  |  |  | | | | | | | |
|  | | | | | | | | | | | | 1 |  | BV |  | 25/03/2020 10:56 AM |  |
|  | | | I went to high school. | | | | | | | | | | | | | |  |
|  | | |  | | | | | | | | | | | | | |  |
|  | | | **Codes\\y - partner sociodemorgraphic data\residency** | | | | | | | | | | | | | |  |
|  |  |  |  |  | No |  | 0.0006 |  | 1 |  | | | | | | |  |
|  | | |  |  |  |  |  |  |  |  | | | | | | | |
|  | | | | | | | | | | | | 1 |  | BV |  | 25/03/2020 10:56 AM |  |
|  | | | Brisbane | | | | | | | | | | | | | |  |
|  | | |  | | | | | | | | | | | | | |  |
|  | | | | | | | | | | | | | | | | | |
| Formatted Reports\\Coding Summary by File Formatted Report | | | | | | | | | | | Page 33 of 39 | | | | | | |
| 6/10/2022 8:17 AM | | | | | | | | | | | | | | | | | |
|  | | | **Classification** |  | **Aggregate** |  | **Coverage** |  | **Number Of Coding References** |  | | **Reference Number** |  | **Coded By Initials** |  | **Modified On** |  |
|  | | | **Codes\\y - partner sociodemorgraphic data\work status** | | | | | | | | | | | | | |  |
|  |  |  |  |  | No |  | 0.0018 |  | 1 |  | | | | | | |  |
|  | | |  |  |  |  |  |  |  |  | | | | | | | |
|  | | | | | | | | | | | | 1 |  | BV |  | 25/03/2020 10:57 AM |  |
|  | | | Yes I would be retired. | | | | | | | | | | | | | |  |
|  | | |  | | | | | | | | | | | | | |  |
|  | | | **Codes\\y - partner sociodemorgraphic data\years married** | | | | | | | | | | | | | |  |
|  |  |  |  |  | No |  | 0.0011 |  | 1 |  | | | | | | |  |
|  | | |  |  |  |  |  |  |  |  | | | | | | | |
|  | | | | | | | | | | | | 1 |  | BV |  | 25/03/2020 10:56 AM |  |
|  | | | About 27 years | | | | | | | | | | | | | |  |
|  | | |  | | | | | | | | | | | | | |  |
|  | **Files\\ID014.4** | | | | | | | | | | | | | | | |  |
|  | | **Code** | | | | | | | | | | | | | | |  |
|  | | | **Codes\\currenting receiving other treatment** | | | | | | | | | | | | | |  |
|  |  |  |  |  | No |  | 0.0774 |  | 1 |  | | | | | | |  |
|  | | |  |  |  |  |  |  |  |  | | | | | | | |
|  | | | | | | | | | | | | 1 |  | BV |  | 25/03/2020 11:26 AM |  |
|  | | | Yeah I am, yeah but as I say I’m feeling very anxious as to what we do next. The oncologist has suggested it would appropriate to me to continue with the Cabazitaxel but obviously outside of the trial now.  Q: How do you feel about that?  A: Umm yeah I think, while it’s continuing to have some benefit I’d probably be in favour of doing that unless there’s something else he might mention that could be perhaps more beneficial but my understanding is that at this stage of my illness it’s good to continue with things that are working you know, rather than just jumping from one to another so exhaust one option before going on to another | | | | | | | | | | | | | |  |
|  | | |  |  |  |  |  |  |  |  |  |  |  |  |  |  |  |
|  | | |  | | | | | | | | | | | | | |  |
|  | | | **Codes\\emotional impact** | | | | | | | | | | | | | |  |
|  |  |  |  |  | No |  | 0.0238 |  | 1 |  | | | | | | |  |
|  | | |  |  |  |  |  |  |  |  | | | | | | | |
|  | | | | | | | | | | | | 1 |  | BV |  | 25/03/2020 11:28 AM |  |
|  | | | I’m still feeling pretty positive but things are, you know, my illness is stable which is encouraging. Obviously if it starts to take a decline again than maybe, mentally, I might feel a bit different | | | | | | | | | | | | | |  |
|  | | |  | | | | | | | | | | | | | |  |
|  | | | | | | | | | | | | | | | | | |
| Formatted Reports\\Coding Summary by File Formatted Report | | | | | | | | | | | Page 34 of 39 | | | | | | |
| 6/10/2022 8:17 AM | | | | | | | | | | | | | | | | | |
|  | | | **Classification** |  | **Aggregate** |  | **Coverage** |  | **Number Of Coding References** |  | | **Reference Number** |  | **Coded By Initials** |  | **Modified On** |  |
|  | | | **Codes\\emotional impact\coping strategies** | | | | | | | | | | | | | |  |
|  |  |  |  |  | No |  | 0.0238 |  | 1 |  | | | | | | |  |
|  | | |  |  |  |  |  |  |  |  | | | | | | | |
|  | | | | | | | | | | | | 1 |  | BV |  | 25/03/2020 11:28 AM |  |
|  | | | I’m still feeling pretty positive but things are, you know, my illness is stable which is encouraging. Obviously if it starts to take a decline again than maybe, mentally, I might feel a bit different | | | | | | | | | | | | | |  |
|  | | |  | | | | | | | | | | | | | |  |
|  | | | **Codes\\happy about participating** | | | | | | | | | | | | | |  |
|  |  |  |  |  | No |  | 0.0816 |  | 3 |  | | | | | | |  |
|  | | |  |  |  |  |  |  |  |  | | | | | | | |
|  | | | | | | | | | | | | 1 |  | BV |  | 25/03/2020 11:22 AM |  |
|  | | | it’s been successful overall. I’m pleased, I’ve done, did the trial and I think I’ve had very close umm, scrutiny of my health over the time I’ve been on the trial which has been reassuring me but obviously now I get a little bit anxious now about you know, what the next phase of treatment will be for me, now that trial has completely finished. | | | | | | | | | | | | | |  |
|  | | |  | | | | | | | | | | | | | |  |
|  | | | | | | | | | | | | 2 |  | BV |  | 25/03/2020 11:29 AM |  |
|  | | | Wouldn’t it just. I mean you’ve got to keep positive and you know, I’m very grateful for all the treatment that I’ve received which I’ve said to you before. It’s just great. You know, the advances there are in medical science at the moment. | | | | | | | | | | | | | |  |
|  | | |  | | | | | | | | | | | | | |  |
|  | | | | | | | | | | | | 3 |  | BV |  | 25/03/2020 11:30 AM |  |
|  | | | I’m only too happy to. I myself have benefited from people who have done these trials before. | | | | | | | | | | | | | |  |
|  | | |  | | | | | | | | | | | | | |  |
|  | | | **Codes\\happy about participating\grateful for treatment** | | | | | | | | | | | | | |  |
|  |  |  |  |  | No |  | 0.0286 |  | 1 |  | | | | | | |  |
|  | | |  |  |  |  |  |  |  |  | | | | | | | |
|  | | | | | | | | | | | | 1 |  | BV |  | 25/03/2020 11:29 AM |  |
|  | | | Wouldn’t it just. I mean you’ve got to keep positive and you know, I’m very grateful for all the treatment that I’ve received which I’ve said to you before. It’s just great. You know, the advances there are in medical science at the moment. | | | | | | | | | | | | | |  |
|  | | |  | | | | | | | | | | | | | |  |
|  | | | **Codes\\hope** | | | | | | | | | | | | | |  |
|  |  |  |  |  | No |  | 0.1024 |  | 3 |  | | | | | | |  |
|  | | |  |  |  |  |  |  |  |  | | | | | | | |
|  | | | | | | | | | | | | 1 |  | BV |  | 25/03/2020 11:29 AM |  |
|  | | | I think that’s been the problem with this illness, since I had, since I was first diagnosed back in 2012, is not knowing how long it’s going to go on for, if there’s going to be any eventual cure, which I’m not confident on. I mean, there’s no cure for prostate cancer at the moment but you always live in hope that you’re suddenly going to hear some medical breakthrough. | | | | | | | | | | | | | |  |
|  | | |  | | | | | | | | | | | | | |  |
|  | | | | | | | | | | | | 2 |  | BV |  | 25/03/2020 11:29 AM |  |
|  | | | Wouldn’t it just. I mean you’ve got to keep positive and you know, I’m very grateful for all the treatment that I’ve received which I’ve said to you before. It’s just great. You know, the advances there are in medical science at the moment. | | | | | | | | | | | | | |  |
|  | | |  | | | | | | | | | | | | | |  |
|  | | | | | | | | | | | | 3 |  | BV |  | 25/03/2020 11:30 AM |  |
|  | | | On the top of my head I can’t think of anything at the moment. As I say I remain optimistic about things and I’m hoping that my oncologist, I mean, my oncologists in the past always said that they’ve got various tricks in their toolboxes so I … | | | | | | | | | | | | | |  |
|  | | |  | | | | | | | | | | | | | |  |
| Formatted Reports\\Coding Summary by File Formatted Report | | | | | | | | | | | Page 35 of 39 | | | | | | |
| 6/10/2022 8:17 AM | | | | | | | | | | | | | | | | | |
|  | | | **Classification** |  | **Aggregate** |  | **Coverage** |  | **Number Of Coding References** |  | | **Reference Number** |  | **Coded By Initials** |  | **Modified On** |  |
|  | | | **Codes\\hope\staying positive** | | | | | | | | | | | | | |  |
|  |  |  |  |  | No |  | 0.0531 |  | 2 |  | | | | | | |  |
|  | | |  |  |  |  |  |  |  |  | | | | | | | |
|  | | | | | | | | | | | | 1 |  | BV |  | 25/03/2020 11:28 AM |  |
|  | | | I’m still feeling pretty positive but things are, you know, my illness is stable which is encouraging. Obviously if it starts to take a decline again than maybe, mentally, I might feel a bit different | | | | | | | | | | | | | |  |
|  | | |  | | | | | | | | | | | | | |  |
|  | | | | | | | | | | | | 2 |  | BV |  | 25/03/2020 11:30 AM |  |
|  | | | On the top of my head I can’t think of anything at the moment. As I say I remain optimistic about things and I’m hoping that my oncologist, I mean, my oncologists in the past always said that they’ve got various tricks in their toolboxes so I … | | | | | | | | | | | | | |  |
|  | | |  | | | | | | | | | | | | | |  |
|  | | | **Codes\\no additional support required** | | | | | | | | | | | | | |  |
|  |  |  |  |  | No |  | 0.0571 |  | 2 |  | | | | | | |  |
|  | | |  |  |  |  |  |  |  |  | | | | | | | |
|  | | | | | | | | | | | | 1 |  | BV |  | 25/03/2020 11:28 AM |  |
|  | | | I don’t think so at the moment. I’m still feeling pretty positive but things are, you know, my illness is stable which is encouraging. Obviously if it starts to take a decline again than maybe, mentally, I might feel a bit different. | | | | | | | | | | | | | |  |
|  | | |  | | | | | | | | | | | | | |  |
|  | | | | | | | | | | | | 2 |  | BV |  | 25/03/2020 11:30 AM |  |
|  | | | On the top of my head I can’t think of anything at the moment. As I say I remain optimistic about things and I’m hoping that my oncologist, I mean, my oncologists in the past always said that they’ve got various tricks in their toolboxes so I … | | | | | | | | | | | | | |  |
|  | | |  | | | | | | | | | | | | | |  |
|  | | | **Codes\\no cure** | | | | | | | | | | | | | |  |
|  |  |  |  |  | No |  | 0.0731 |  | 2 |  | | | | | | |  |
|  | | |  |  |  |  |  |  |  |  | | | | | | | |
|  | | | | | | | | | | | | 1 |  | BV |  | 25/03/2020 11:29 AM |  |
|  | | | I think that’s been the problem with this illness, since I had, since I was first diagnosed back in 2012, is not knowing how long it’s going to go on for, if there’s going to be any eventual cure, which I’m not confident on. I mean, there’s no cure for prostate cancer at the moment but you always live in hope that you’re suddenly going to hear some medical breakthrough. | | | | | | | | | | | | | |  |
|  | | |  | | | | | | | | | | | | | |  |
|  | | | | | | | | | | | | 2 |  | BV |  | 25/03/2020 11:29 AM |  |
|  | | | Wouldn’t it just. I mean you’ve got to keep positive and you know, I’m very grateful for all the treatment that I’ve received which I’ve said to you before. It’s just great. You know, the advances there are in medical science at the moment. | | | | | | | | | | | | | |  |
|  | | |  | | | | | | | | | | | | | |  |
|  | | | **Codes\\PSA post treatment\PSA numbers not staying down - cabazitaxel** | | | | | | | | | | | | | |  |
|  |  |  |  |  | No |  | 0.1148 |  | 2 |  | | | | | | |  |
|  | | |  |  |  |  |  |  |  |  | | | | | | | |
|  | | | | | | | | | | | | 1 |  | BV |  | 25/03/2020 11:22 AM |  |
|  | | | Yeah pretty good since the last call, yeah. I mean you know just same old same old. I’ve been continuing with the treatment. I think I’ve got round ten which I think is the last one on the trial, next week. Umm and then, then you know, we just have to see where I go after that but yeah I think you know, the trial or the medication I’ve been on, the chemotherapy, has obviously had some success but not umm you know, it’s not got rid of the problem. It’s brought my PSA level down so you know, there is still a battle ahead, clearly. | | | | | | | | | | | | | |  |
|  | | |  | | | | | | | | | | | | | |  |
|  | | | | | | | | | | | | | | | | | |
| Formatted Reports\\Coding Summary by File Formatted Report | | | | | | | | | | | Page 36 of 39 | | | | | | |
| 6/10/2022 8:17 AM | | | | | | | | | | | | | | | | | |
|  | | | **Classification** |  | **Aggregate** |  | **Coverage** |  | **Number Of Coding References** |  | | **Reference Number** |  | **Coded By Initials** |  | **Modified On** |  |
|  | | | | | | | | | | | | | | | | | |
|  | | | | | | | | | | | | 2 |  | BV |  | 25/03/2020 11:31 AM |  |
|  | | | Do you know what you’re PSA is at the moment?  A: It was 16 I think, last time.  Q: Ok.  A: It’s never been that high. I’m one of these prostate cancer people who have had fairly low PSA throughout but ended up with a very high grade tumour. So I understand I’m in the unusual bracket. You know, low PSA high grade tumour. So it’s never gone that high but it’s you know, certainly been lower than it is currently. | | | | | | | | | | | | | |  |
|  | | |  |  |  |  |  |  |  |  |  |  |  |  |  |  |  |
|  | | |  | | | | | | | | | | | | | |  |
|  | | | **Codes\\quality of life changes\quality of life changes - cabazitaxel** | | | | | | | | | | | | | |  |
|  |  |  |  |  | No |  | 0.0657 |  | 2 |  | | | | | | |  |
|  | | |  |  |  |  |  |  |  |  | | | | | | | |
|  | | | | | | | | | | | | 1 |  | BV |  | 25/03/2020 11:27 AM |  |
|  | | | Umm, I would have to say it’s not really. I can still do everything I want you know. I’m a keen runner but my running has taken a bit of a knock since I’ve been on the trial, since I’ve been on the chemotherapy. Certainly I’m not able to run like I was able to previously but I think that’s a combination of the growth of my illness, tumour wise and probably also the effects of the chemotherapy. | | | | | | | | | | | | | |  |
|  | | |  | | | | | | | | | | | | | |  |
|  | | | | | | | | | | | | 2 |  | BV |  | 25/03/2020 11:28 AM |  |
|  | | | I’m keeping up my exercise.  Q: That’s good.  A: I’d be interested to see what, you know, how I bounce back if I do, once I finish this treatment. | | | | | | | | | | | | | |  |
|  | | |  | | | | | | | | | | | | | |  |
|  | | | **Codes\\quality of trialists** | | | | | | | | | | | | | |  |
|  |  |  |  |  | No |  | 0.0627 |  | 2 |  | | | | | | |  |
|  | | |  |  |  |  |  |  |  |  | | | | | | | |
|  | | | | | | | | | | | | 1 |  | BV |  | 25/03/2020 11:22 AM |  |
|  | | | it’s been successful overall. I’m pleased, I’ve done, did the trial and I think I’ve had very close umm, scrutiny of my health over the time I’ve been on the trial which has been reassuring me but obviously now I get a little bit anxious now about you know, what the next phase of treatment will be for me, now that trial has completely finished. | | | | | | | | | | | | | |  |
|  | | |  | | | | | | | | | | | | | |  |
|  | | | | | | | | | | | | 2 |  | BV |  | 25/03/2020 11:30 AM |  |
|  | | | I mean I’ve been interacting with the same people and they’ve been supportive. So yeah I’ve been quite happy with the interest been shown in me by my family and professionals. | | | | | | | | | | | | | |  |
|  | | |  | | | | | | | | | | | | | |  |
|  | | | **Codes\\receiving cabazitaxel outside of the trial** | | | | | | | | | | | | | |  |
|  |  |  |  |  | No |  | 0.0774 |  | 1 |  | | | | | | |  |
|  | | |  |  |  |  |  |  |  |  | | | | | | | |
|  | | | | | | | | | | | | 1 |  | BV |  | 25/03/2020 11:26 AM |  |
|  | | | Yeah I am, yeah but as I say I’m feeling very anxious as to what we do next. The oncologist has suggested it would appropriate to me to continue with the Cabazitaxel but obviously outside of the trial now.  Q: How do you feel about that?  A: Umm yeah I think, while it’s continuing to have some benefit I’d probably be in favour of doing that unless there’s something else he might mention that could be perhaps more beneficial but my understanding is that at this stage of my illness it’s good to continue with things that are working you know, rather than just jumping from one to another so exhaust one option before going on to another | | | | | | | | | | | | | |  |
|  | | |  |  |  |  |  |  |  |  |  |  |  |  |  |  |  |
|  | | |  | | | | | | | | | | | | | |  |
| Formatted Reports\\Coding Summary by File Formatted Report | | | | | | | | | | | Page 37 of 39 | | | | | | |
| 6/10/2022 8:17 AM | | | | | | | | | | | | | | | | | |
|  | | | **Classification** |  | **Aggregate** |  | **Coverage** |  | **Number Of Coding References** |  | | **Reference Number** |  | **Coded By Initials** |  | **Modified On** |  |
|  | | | **Codes\\side effects\comparing treatment to chemotherapy** | | | | | | | | | | | | | |  |
|  |  |  |  |  | No |  | 0.0296 |  | 1 |  | | | | | | |  |
|  | | |  |  |  |  |  |  |  |  | | | | | | | |
|  | | | | | | | | | | | | 1 |  | BV |  | 25/03/2020 11:32 AM |  |
|  | | | Obviously looking forward to hearing the results from the trial because as you know, it was a comparison trial between Cabazitaxel and Lutetium so I’ll be very keen to know how other people have got on and certainly how the Lutetium people got on. | | | | | | | | | | | | | |  |
|  | | |  | | | | | | | | | | | | | |  |
|  | | | **Codes\\side effects\side effects - cabazitaxel** | | | | | | | | | | | | | |  |
|  |  |  |  |  | No |  | 0.1191 |  | 2 |  | | | | | | |  |
|  | | |  |  |  |  |  |  |  |  | | | | | | | |
|  | | | | | | | | | | | | 1 |  | BV |  | 25/03/2020 11:21 AM |  |
|  | | | Yeah pretty good since the last call, yeah. I mean you know just same old same old. I’ve been continuing with the treatment. I think I’ve got round ten which I think is the last one on the trial, next week. Umm and then, then you know, we just have to see where I go after that but yeah I think you know, the trial or the medication I’ve been on, the chemotherapy, has obviously had some success but not umm you know, it’s not got rid of the problem. It’s brought my PSA level down so you know, there is still a battle ahead, clearly. | | | | | | | | | | | | | |  |
|  | | |  | | | | | | | | | | | | | |  |
|  | | | | | | | | | | | | 2 |  | BV |  | 25/03/2020 11:27 AM |  |
|  | | | Physically umm, probably a little bit yuckier than before, a bit more tired perhaps, hair loss, hair thinning a bit. I’ve been lucky with regards to hair loss whilst I’ve been on the trial but I’ve noticed a bit of thinning these last few weeks. So, yeah, you know and a bit tired but I‘ve had that throughout. I would say in the last couple of weeks, since I had the last round of chemo, I’ve felt a little bit yuckier than I had before and got a bit tireder. | | | | | | | | | | | | | |  |
|  | | |  | | | | | | | | | | | | | |  |
|  | | | **Codes\\still a battle ahead** | | | | | | | | | | | | | |  |
|  |  |  |  |  | No |  | 0.0641 |  | 1 |  | | | | | | |  |
|  | | |  |  |  |  |  |  |  |  | | | | | | | |
|  | | | | | | | | | | | | 1 |  | BV |  | 25/03/2020 11:22 AM |  |
|  | | | Yeah pretty good since the last call, yeah. I mean you know just same old same old. I’ve been continuing with the treatment. I think I’ve got round ten which I think is the last one on the trial, next week. Umm and then, then you know, we just have to see where I go after that but yeah I think you know, the trial or the medication I’ve been on, the chemotherapy, has obviously had some success but not umm you know, it’s not got rid of the problem. It’s brought my PSA level down so you know, there is still a battle ahead, clearly. | | | | | | | | | | | | | |  |
|  | | |  | | | | | | | | | | | | | |  |
|  | | | **Codes\\uncertainty of the future** | | | | | | | | | | | | | |  |
|  |  |  |  |  | No |  | 0.2246 |  | 4 |  | | | | | | |  |
|  | | |  |  |  |  |  |  |  |  | | | | | | | |
|  | | | | | | | | | | | | 1 |  | BV |  | 25/03/2020 11:22 AM |  |
|  | | | Yeah pretty good since the last call, yeah. I mean you know just same old same old. I’ve been continuing with the treatment. I think I’ve got round ten which I think is the last one on the trial, next week. Umm and then, then you know, we just have to see where I go after that but yeah I think you know, the trial or the medication I’ve been on, the chemotherapy, has obviously had some success but not umm you know, it’s not got rid of the problem. It’s brought my PSA level down so you know, there is still a battle ahead, clearly. | | | | | | | | | | | | | |  |
|  | | |  | | | | | | | | | | | | | |  |
|  | | | | | | | | | | | | 2 |  | BV |  | 25/03/2020 11:26 AM |  |
|  | | | Yeah I am, yeah but as I say I’m feeling very anxious as to what we do next. The oncologist has suggested it would appropriate to me to continue with the Cabazitaxel but obviously outside of the trial now.  Q: How do you feel about that?  A: Umm yeah I think, while it’s continuing to have some benefit I’d probably be in favour of doing that unless there’s something else he might mention that could be perhaps more beneficial but my understanding is that at this stage of my illness it’s good to continue with things that are working you know, rather than just jumping from one to another so exhaust one option before going on to another | | | | | | | | | | | | | |  |
|  | | |  |  |  |  |  |  |  |  |  |  |  |  |  |  |  |
|  | | |  | | | | | | | | | | | | | |  |
| Formatted Reports\\Coding Summary by File Formatted Report | | | | | | | | | | | Page 38 of 39 | | | | | | |
| 6/10/2022 8:17 AM | | | | | | | | | | | | | | | | | |
|  | | | **Classification** |  | **Aggregate** |  | **Coverage** |  | **Number Of Coding References** |  | | **Reference Number** |  | **Coded By Initials** |  | **Modified On** |  |
|  | | | | | | | | | | | | | | | | | |
|  | | | | | | | | | | | | 3 |  | BV |  | 25/03/2020 11:29 AM |  |
|  | | | I think that’s been the problem with this illness, since I had, since I was first diagnosed back in 2012, is not knowing how long it’s going to go on for, if there’s going to be any eventual cure, which I’m not confident on. I mean, there’s no cure for prostate cancer at the moment but you always live in hope that you’re suddenly going to hear some medical breakthrough. | | | | | | | | | | | | | |  |
|  | | |  | | | | | | | | | | | | | |  |
|  | | | | | | | | | | | | 4 |  | BV |  | 25/03/2020 11:30 AM |  |
|  | | | Yeah. I’m not sure if it will be at the next consultation or whether we will be pencilling one for when the treatment is actually finished. When I have my treatment it’s a three week cycle so he might say let’s come back in three weeks once the chemo has done its stuff for this cycle. I remain to be guided by him really. | | | | | | | | | | | | | |  |
|  | | |  | | | | | | | | | | | | | |  |
|  | | | **Codes\\uncertainty of the future\treatments left on TheraP** | | | | | | | | | | | | | |  |
|  |  |  |  |  | No |  | 0.0088 |  | 1 |  | | | | | | |  |
|  | | |  |  |  |  |  |  |  |  | | | | | | | |
|  | | | | | | | | | | | | 1 |  | BV |  | 25/03/2020 11:24 AM |  |
|  | | | My final treatment I think, from the top of my head, a week from Tuesday. | | | | | | | | | | | | | |  |
|  | | |  | | | | | | | | | | | | | |  |
|  | | | **Codes\\x - sociodemographic data\current PSA** | | | | | | | | | | | | | |  |
|  |  |  |  |  | No |  | 0.0181 |  | 1 |  | | | | | | |  |
|  | | |  |  |  |  |  |  |  |  | | | | | | | |
|  | | | | | | | | | | | | 1 |  | BV |  | 25/03/2020 11:31 AM |  |
|  | | | I mean, so far so good with the treatment which is excellent to hear. Do you know what you’re PSA is at the moment?  A: It was 16 I think, last time. | | | | | | | | | | | | | |  |
|  | | |  | | | | | | | | | | | | | |  |
|  | | | | | | | | | | | | | | | | | |
|  | | | | | | | | | | | | | | | | | |
|  | | | | | | | | | | | | | | | | | |
|  | | | | | | | | | | | | | | | | | |
| Formatted Reports\\Coding Summary by File Formatted Report | | | | | | | | | | | Page 39 of 39 | | | | | | |
