# Supplementary material for: Experiences of participants in a clinical trial of a novel radioactive treatment for advanced prostate cancer: A nested, qualitative longitudinal study
Source: PLoS One. 2022 Nov 9;17(11):e0276063. doi: 10.1371/journal.pone.0276063 (PMC9645653; doi:10.1371/journal.pone.0276063)
Supplement: Attachment — Submitted filename: Response to Reviewers.docx [file pone.0276063.s003.docx]

Academic Editor

*PLOS ONE*

Date: 8th September 2022

Subject: Submission of revised manuscript

Viljoen B et al: Experiences of participants in a clinical trial of a novel radioactive treatment for advanced prostate cancer: A nested, qualitative longitudinal study

Manuscript number: PONE-D-22-10351

Dear Randall J. Kimple,

We would like to thank the reviewers for their comments.

We have carefully reviewed the comments and have revised the manuscript accordingly. Our responses are provided in a point-by-point manner below.

Please note that the reference list has been amended. Reference 2 and 3 have been removed and do not require replacement.

The authors have declared that no competing interests exist.

We hope the revised version is now suitable for publication and look forward to hearing from you in due course.

Sincerely,

Bianca Viljoen

Lead Author

**Comments to the Author**

We have answered each of these comments below:

| **Comments** | **Authors Response** |
| --- | --- |
| **3. Has the statistical analysis been performed appropriately and rigorously?**  **Reviewer #4: No** | We are unsure why there is a comment about the rigour of statistical analyses in a qualitative paper. An interpretative phenomenological approach was used, and interviews analysed using thematic analysis. The current manuscript reports the results from the mid-point, conclusion and follow up interviews, focusing specifically on participants’ experiences of trial participation. |

| **Comments** | **Authors Response** |
| --- | --- |
| **4. Have the authors made all data underlying the findings in their manuscript fully available. The PLOS Data policy requires authors to make all data underlying the findings described in their manuscript fully available without restriction, with rare exception. The data should be provided as part of the manuscript or its supporting information or deposited to a public repository.**  **Reviewer #3: No**  **Reviewer #4: No** | All data underlying the findings described in the manuscript have been made available to PLOS ONE as supplementary files. Participants information has been kept confidential. |

**Response to Reviewer 1**

Thank you for your review of our paper. We have answered your comment below:

| **Reviewer Comments** | **Authors Response** |
| --- | --- |
| **Minor: Page 5, line 118: Did authors mean “deprivation” instead of “detection”.** | The word has been amended to state ‘deprivation’. |

**Response to Reviewer 2**

Thank you for your review of our paper. We have answered your comment below:

| **Reviewer Comments** | **Authors Response** |
| --- | --- |
| **Small editorial correction: Introduction, page 4: “androgen detection therapy” should be “androgen deprivation therapy”.** | The word has been amended to state ‘deprivation’. |

**Response to Reviewer 3**

Thank you for your review of our paper. We have answered your comment below:

| **Reviewer Comments** | **Authors Response** |
| --- | --- |
| **I previously suggested that the authors ‘include in the results some comparison of characteristics of these 15 patients to all patients on the randomized trial. Are they similar age, …’. The authors have responded that ‘Specific data from participants in the larger trial can unfortunately not be included in the paper for comparison’. It is not clear to me why this is? Are you not allowed to give the mean age of all patients enrolled on the larger trial? Is this not already published?** | The mean (SD) age of participants in the larger TheraP trial have been included in Table 2. Furthermore, the total participants allocated to the LuPSMA and Cabazitaxel arms have also been included, as published in the Lancet: <https://www.thelancet.com/journals/lancet/article/PIIS0140-6736(21)00237-3/fulltext>  We wish to make the distinction that the language used to draw conclusions does not necessarily relate to the broader population of trial patients in the TheraP trial. |

**Response to Reviewer 4**

Thank you for your review of our paper. We have answered your comment below:

| **Reviewer Comments** | **Authors Response** |
| --- | --- |
| **This qualitative study describes the lived experiences of men with advanced prostate cancer participating in the TheraP trial; an open-label randomised trial of 177Lu-PSMA-617 compared with cabazitaxel chemotherapy. An interpretative phenomenological approach was used, and interviews analysed using thematic analysis. The current manuscript reports the results from the mid-point, conclusion and follow up interviews, focusing specifically on participants’ experiences of trial participation.  1) The current manuscript identifies three representative themes from the participants. As an open-label trial, it is disappointing the authors didn’t try to identify the differences, if there is any, of the participants between the two arms, which can be very interesting. Since all the materials are available, please dig into it more, and present the findings, even if it’s null.** | The results of the study identify the overall lived experiences of men with advanced prostate cancer participating in the TheraP trial. The authors believe that the similarities and differences in experiences are shown in the results through the inclusion of participants perspectives and selected quotations from participants in both the LuPSMA and Cabazitaxel treatment arms.  Some changes have been made to the results to try and emphasise the comparison even more (page 14, line 210; page 15 line 232 and 250). |
